# Relationship between craniofacial skeletal patterns and anatomic characteristics of masticatory muscles: a systematic review and meta-analysis

**DOI:** 10.1186/s40510-024-00534-2

**Published:** 2024-09-09

**Authors:** David Togninalli, Gregory S. Antonarakis, Alexandra K. Papadopoulou

**Affiliations:** 1 Division of Orthodontics, University Clinics of Dental Medicine, Faculty of Medicine, University of Geneva, Geneva, Switzerland; 2 Discipline of Orthodontics and Paediatric Dentistry, Sydney Dental School, Faculty of Medicine and Health, The University of Sydney, Sydney, Australia

**Keywords:** Masticatory muscles, Masseter, Craniofacial pattern, Skeletal pattern, Systematic review, Meta-analysis

## Abstract

**Background:**

The anatomic characteristics of the masticatory muscles differ across craniofacial skeletal patterns.

**Objective:**

To identify differences in the anatomic characteristics of masticatory muscles across different sagittal and vertical craniofacial skeletal patterns.

**Eligibility criteria:**

Studies measuring the thickness, width, cross-sectional area (CSA), volume and orientation of masticatory muscles in healthy patients of different sagittal (Class I, Class II, and Class III) and/or vertical (normodivergent, hypodivergent, and hyperdivergent) patterns.

**Information sources:**

Unrestricted literature searches in 8 electronic databases/registers until December 2023.

**Risk of bias and synthesis of results:**

Study selection, data extraction, and risk of bias assessment with a customised tool were performed independently in duplicate. Random-effects meta-analysis and assessment of the certainty of clinical recommendations with the GRADE approach were conducted.

**Results:**

34 studies (37 publications) were selected with a total of 2047 participants and data from 16 studies were pulled in the meta-analysis. Masseter muscle thickness in relaxation was significantly greater by 1.14 mm (95% CI 0.74–1.53 mm) in hypodivergent compared to normodivergent patients while it was significantly decreased in hyperdivergent patients by − 1.14 mm (95% CI − 1.56 to − 0.73 mm) and − 2.28 mm (95% CI − 2.71 to − 1.85 mm) compared to normodivergent and hypodivergent patients respectively. Similar significant differences were seen between these groups in masseter muscle thickness during contraction as well as masseter muscle CSA and volume. Meta-analyses could not be performed for sagittal categorizations due to insufficient number of studies.

**Conclusions:**

Considerable differences in masseter muscle thickness, CSA and volume were found across vertical skeletal configurations being significantly reduced in hyperdivergent patients; however, results should be interpreted with caution due to the high risk of bias of the included studies. These variations in the anatomic characteristics of masticatory muscles among different craniofacial patterns could be part of the orthodontic diagnosis and treatment planning process.

**Registration**: PROSPERO CRD42022371187.

**Supplementary Information:**

The online version contains supplementary material available at 10.1186/s40510-024-00534-2.

## Introduction

### Rationale

Craniofacial growth begins in-utero during embryogenesis with the migration and differentiation of neural crest cells. Tissue organization and growth patterns are highly dictated by the expression of genes involved in these processes. However, environmental factors can potentially alter this pre- or post-natal growth [1]. It has been shown that muscles and the stresses they exert on the bone can have an important influence on its remodelling and morphology [2–4]. Several studies have investigated the relationship between muscle thickness and craniofacial dimensions. Van Spronsen et al. [5] highlighted a significant correlation between the cross-sectional area of the anterior temporal muscle and facial width. Kitai et al. [6] suggested that the muscular variables are significantly correlated with the bizygomatic arch width and temporal fossa but are not correlated with the cranial width. Chan et al. [7] found that growing subjects with thicker masticatory muscles are more likely to have a greater bizygomatic arch width.

Differences in muscle fibre orientation and insertion are also related to different dentofacial morphologies with the masticatory muscles of individuals with a skeletal hypodivergent pattern being more uprightly oriented and anteriorly inserted compared to those with skeletal hyperdivergent patterns [8]. Takada et al. [9] suggest an association between vertically oriented masseter muscles that are anteriorly attached on the mandible and a long posterior face height accompanied with a flat mandibular plane and an acute gonial angle in hypodivergent children. On the other hand, Proctor and De Vincenzo report more horizontally oriented masseter muscles relative to the cranial base, Frankfort horizontal and the palatal plane in hyperdivergent individuals [10].

When masticatory forces were evaluated in adult individuals, patients with less powerful and thinner masticatory muscles were dolichocephalic. Conversely, subjects with strong masticatory muscles have a rather brachycephalic facial pattern [11, 12]. This difference in masticatory muscle strength between normodivergent and hyperdivergent individuals is not evident in children aged 6–11 years, indicating a possible inability of the mandibular elevator muscles to gain strength in the hyperdivergent group with growth [13]. Additionally, these differences in muscular force capacity are reflected in muscle thickness as patients affected by degenerative neuromuscular diseases have thinner muscles of lower strength that directly affect craniofacial morphology [14].

The function of the masticatory muscles is linked to increased stress on the jaws and the formation of bone, potentially influencing areas such as the gonial angle [11]. This part of the mandible serves as a point of attachment for the masseter and medial pterygoid muscles, thus variations in their size and activity could impact mandibular shape and, consequently, overall dentofacial morphology. According to Wolff’s law (1870), bone structure is influenced by muscle thickness, implying a connection between muscle function and the internal skeletal structure and form [15, 16]. An inverse correlation has been reported between masseter muscle thickness and anterior face height, mandibular plane angle and the gonial angle, while masseter muscle thickness was positively correlated with mandibular ramus height and posterior facial height [17–27].

Diverse methods have been used for the assessments of masticatory muscle attributes. Muscle biopsies have been employed to analyse characteristics such as muscle fibre type, composition and thickness, but due to its invasive nature is not used routinely [28]. Alternatively, muscular functional activity and force generation can be assessed with electromyography and bite force measurements respectively while muscular dimensions are measured using computer tomography, magnetic resonance imaging and ultrasonography. From the imaging techniques, ultrasonography (US) is the most widespread method for the analysis of the thickness of masticatory muscles for its advantages such as the absence of ionizing radiation, convenience, and rapidity [27, 29–32].

### Objectives

The aim of this systematic review was to assess in a systematic manner the available evidence regarding the potential relationships between craniofacial patterns (sagittal and vertical) and masticatory muscle macroscopic anatomic characteristics, such as thickness (depth), width, cross-sectional area, volume and angle orientation assessed with any 2D or 3D imaging method.

## Methods

### Protocol registration, research question and eligibility criteria

The reporting of the present systematic review and meta-analysis is based on the PRISMA guidelines [33].

The study protocol was registered with the international prospective register of systematic reviews (PROSPERO CRD42022371187).

The research question was whether any differences exist between the size and/or orientation characteristics of the muscles of mastication in healthy humans and different craniofacial patterns (sagittal or vertical). The eligibility criteria were based on the PECO framework (population, exposure, comparator, outcomes) and are described in detail in Supplementary Table 1.

### Information sources and search strategy

The databases, registers, websites, organizations, and other sources searched to identify potentially eligible studies as well as all search strategies are described in Supplementary Table 2. The last electronic literature search was performed on December 2023 and no limitations regarding publication year, language, status, or type were imposed (apart from filters for studies on humans, where they existed). Additionally, the reference lists of all included studies and all relevant systematic reviews were checked for additional studies. The literature search was carried out by two reviewers independently (DT and AKP).

### Study selection

The selection of studies was performed by two independent reviewers (DT, AKP) based on screening titles and abstracts. The full-text versions of the pre-selected articles were accessed to assess their eligibility. Any disagreements were resolved through discussion while in the absence of consensus, a third reviewer (GSA) was consulted until consensus was reached. All relevant citations were imported to a reference manager software (EndNote® 20, Thomson Reuters, Philadelphia, PA) for de-duplication. Researchers were not blinded to the authors of included studies.

### Data collection and data items

The data collection procedure was carried out by two independent reviewers (DT and AKP) using pre-designed and pre-piloted forms (Supplementary Table 3) and extracted data were imported in digital spreadsheets. Discrepancies were resolved in the same way as above by consulting another author (GSA). Researchers were not blinded to the authors of included studies.

### Study risk of bias assessment risk of bias within individual studies

A customized risk of bias tool was used for assessment of internal validity / reporting quality of each individual study independently and in duplicate by two investigators (DT and AKP). This tool was tailored to the scope of this review’s eligible studies and based on items from The Joanna Briggs Institute's critical appraisal checklists for cross-sectional studies and the Appraisal tool for Cross-Sectional Studies (AXIS) [34, 35]. The specific domains / questions of the customized tool can be seen in Supplementary Table 4.

### Summary measures, data synthesis and certainty assessment

The main objective of our systematic study and meta-analysis was to investigate the differences, if these exist, between the muscles of mastication and the sagittal or vertical craniofacial patterns in healthy humans. The Mean Difference (MD) with its 95% Confidence Interval (CI) was used to estimate anatomic characteristics in masticatory muscle differences among Class I, II and III or among normodivergent, hypodivergent and hyperdivergent patient groups.

As anatomic characteristics in masticatory muscles were expected to vary according to patient-related (chronological age, developmental growth stage, sex) or measurement-related factors (radiographical technical characteristics, method error, cut-off values used for categorization), a random-effects model was a priori deemed (using clinical / methodological justification [36]) most appropriate to incorporate this variability and estimate the average distribution of effects across studies.

The heterogeneity of the studies was determined using I2 statistics as well as Tau^2^ statistics [37, 38]. Pooled mean differences between groups (normo-, hypo- and hyperdivergent) were obtained with multivariate mixed-effects linear models for meta-analyses. Leave-one-out sensitivity analyses were conducted to check the robustness of the findings. The trim and fill approach was used to correct the pooled mean differences for a potential publication bias. Due to the low number of studies, the latest was used only on the most frequently reported outcome (masseter thickness). All statistical tests were two-sided with a significance level of 0.05. Statistical analyses were carried out with the package Metafor v3.8-1 for R v4.0.2 (R Core Team (2020). R: A language and environment for statistical computing. R Foundation for Statistical Computing, Vienna, Austria. URL https://www.R-project.org/ (https://www.r-project.org/)). The certainty of evidence (confidence in effect estimates) for both the primary and secondary outcomes as per the PECO table (Supplementary Table 1) was assessed using the GRADE approach [39].

## Results

### Study selection

**Fig. 1:**
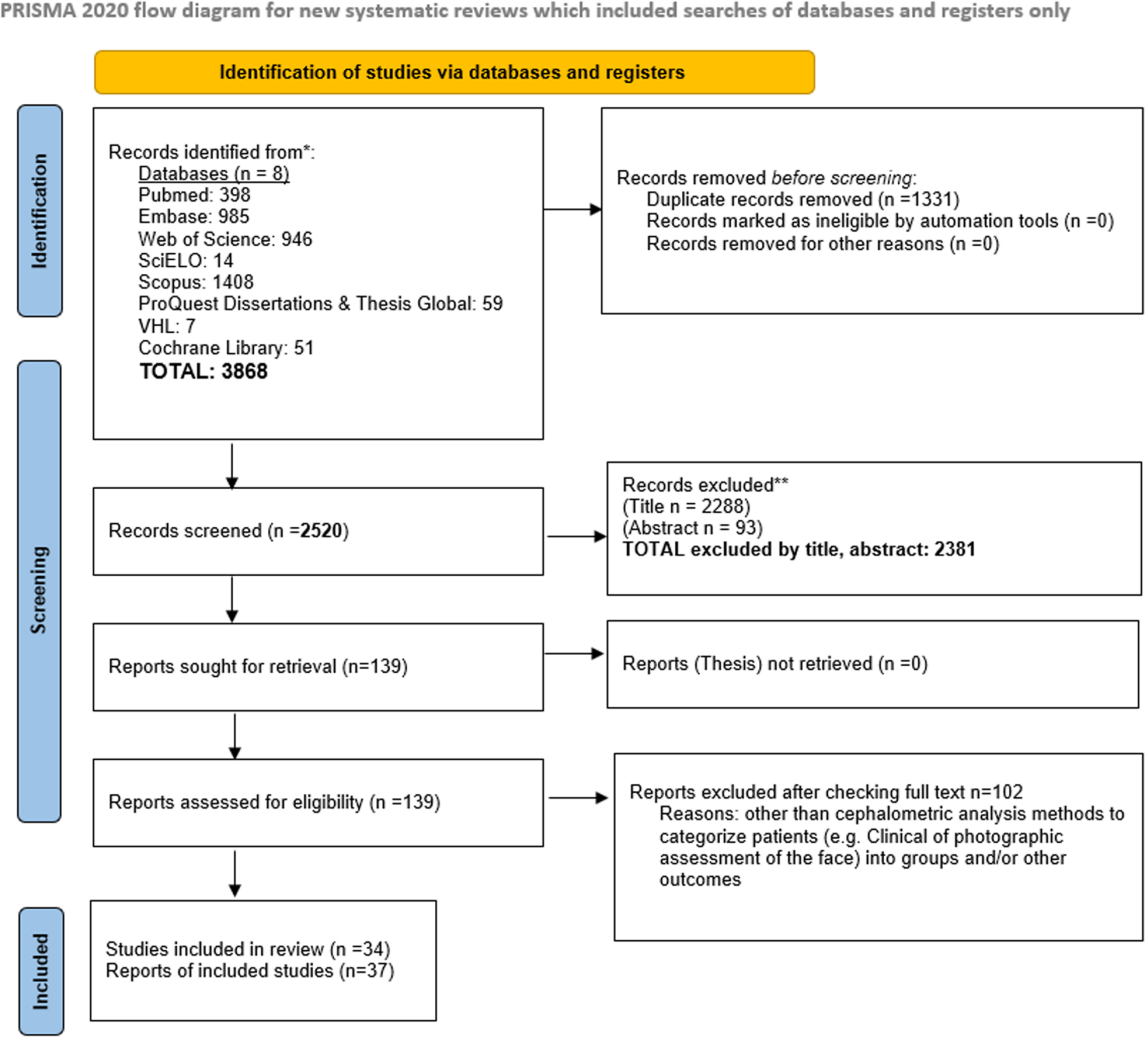
PRISMA flowchart diagram

A total of 3868 studies were retrieved from the databases, which after deduplication, selection according to title, abstract and full text were eliminated to 37 studies included in the present review. Studies were excluded in full text if they assessed outcomes different to the ones of interest or did not include at least two sagittal or vertical groups for comparison. The flow diagram of the studies retrieved from the databases is described in detail in the PRISMA diagram in Fig. 1, Supplementary Table 5. For the quantitative analysis, only 6 studies were included for the cross-sectional area (CSA) of the masseter, 7 for the volume of the masseter, 10 for the thickness of the masseter under relaxation, and 8 for the thickness of the masseter under contraction (during biting). The quantitative analysis focuses solely on patient divergence (hypodivergent vs. normodivergent vs. hyperdivergent), while for the sagittal relationship (Class I vs. Class II vs. Class III), an insufficient number of studies (or subjects) have been published, preventing a meta-analysis from being conducted.

### Study characteristics

Thirty-four (34) studies (37 publications) were included in the present review (28 prospective, 6 retrospective and 1 was of unclear study design). Muscular anatomic characteristics were measured using ultrasonography in 17 studies, computed tomography (CT) in 10 studies and magnetic resonance imaging (MRI) in 8 studies. Fourteen studies were conducted in Europe, 13 in Asia, 4 in Oceania, 3 in Africa and one in the Americas. Four studies classified the patients in sagittal skeletal patterns (Class I, II, III), twenty studies classified the patients in vertical skeletal patterns (hypodivergent, normodivergent, hyperdivergent) and ten studies classified them both in sagittal and vertical skeletal patterns. Seven studies reported on children, twenty studies on adults, four studies had mixed groups and three studies did not report on the age of their sample. The detailed characteristics of the included studies are presented in detail in Supplementary Tables 6–8.

### Risk of bias in individual studies

The risk of bias of the included studies is presented in Supplementary Table 9. In general, the included studies exhibited high risk of bias. Even though the domain of stating aims and objectives was clear, there were several domains that were generally unclear such as the inclusion and exclusion criteria of the samples and the cut-off points of the cephalometric values used to categorize the subjects in the sagittal and vertical skeletal patterns. The domains that were the most problematic were related to the domains of sample size calculation and justification, identification and accounting of confounding factors (age, sex, sagittal classification for studies that grouped per vertical and vice-versa, functional shifts, asymmetries, linear or curved probe used) in the analyses, incomplete reporting of demographics and results (12 studies with vertical categorization and 5 studies with sagittal categorization reported only correlations between cephalometric and muscular parameters), calibration and experience of the assessors of the muscles. Additionally, most of the studies were unclear with regard to if the assessor of the muscles was blinded to the cephalometric variables.

### Results of individual studies and data synthesis

Due to the lack of eligible similar studies from which data could be pulled according to sagittal skeletal categorization, quantitative analysis was feasible only for patients categorized in the vertical dimension.

Descriptive statistics for the cross-sectional area of the masseter muscle (at relaxation and contraction), lateral pterygoid muscle, medial pterygoid muscle, and temporalis muscle are described in Table 1. Descriptive statistics for the volume of the masseter muscle (at relaxation and contraction), lateral pterygoid muscle and medial pterygoid muscle are described in Table 2. Descriptive statistics for the angle of insertion of the masseter muscle relative to the Frankfort horizontal plane are described in Table 3. Descriptive statistics for the width and thickness of the masseter muscle (at relaxation and contraction) are described in Table 4.

**Table 1:** Descriptive statistics for the muscle CSAs per vertical group

Outcomes	Norm		Hypo		Hyper	
	N, n (min–max)	Pooled mean (95% CI); I^2^	N, n (min–max)	Pooled mean (95% CI); I^2^	N, n (min–max)	Pooled mean (95% CI); I^2^
Masseter CSA (cm^2^)	6; 115 [4–35]	3.8 (3.1–4.5); I^2^ = 0.95	4; 62 [10–22]	4.1 (3.2–5.1); I^2^ = 0.98	6; 75 [5–25]	3.3 (2.8–3.8); I^2^ = 0.92
Lateral pterygoid CSA (cm^2^)	1; 35 [35–35]	4.2 (4.0–4.4)	0; 0		1; 13 [13–13]	3.7 (3.4–4.0)
Medial pterygoid CSA (cm^2^)	3; 79 [11–35]	2.9 (2.6–3.2); I^2^ = 0.85	2; 30 [11–19]	2.9 (2.6–3.2); I^2^ = 0.57	3; 46 [8–25]	2.5 (2.2–2.9); I^2^ = 0.77
Temporalis CSA (cm^2^)	1; 35 [35–35]	5.1 (4.9–5.4)	0; 0		1; 13 [13–13]	4.4 (4.1–4.8)
Masseter CSA_bite (cm^2^)	1; 10 [10–10]	3.3 (2.7–3.9)	1; 10 [10–10]	4.1 (3.8–4.4)	1; 10 [10–10]	3.1 (2.7–3.5)

*N* number of studies reporting the outcome in more than 1 patient; *n* total number of patients in the studies; *min–max* number of patients in the smallest and the largest study; *I*^*2*^ statistic for assessing the between-study heterogeneity (from 0 (no heterogeneity) to 1 (extreme heterogeneity))

**Table 2:** Descriptive statistics for the muscle volumes per vertical group

Outcomes	Norm		Hypo		Hyper	
	N, n (min–max)	Pooled mean (95% CI); I^2^	N, n (min–max)	Pooled mean (95% CI); I^2^	N, n (min–max)	Pooled mean (95% CI); I^2^
Masseter volume (cm^3^)	6; 99 [4–33]	17.2 (14.7–19.7); I^2^ = 0.96	6; 88 [5–22]	20.8 (15.7–25.9); I^2^ = 0.98	7; 89 [5–25]	15.7 (12.9–18.4); I^2^ = 0.95
Lateral pterygoid volume (cm^3^)	1; 9 [9–9]	7.0 (6.3–7.8)	1; 5 [5–5]	6.57 (6.1–7.0)	1; 6 [6–6]	5.7 (5.1–6.4)
Medial pterygoid volume (cm^3^)	3; 53 [9–33]	8.1 (6.3–10.0); I^2^ = 0.92	3; 35 [5–19]	8.2 (5.3–11.2); I^2^ = 0.92	3; 39 [6–25]	7.2 (4.4–10.0); I^2^ = 0.91
Masseter volume_bite (cm^3^)	1; 20 [20–20]	14.9 (14.3–15.5)	1; 20 [20–20]	16.7 (16.2–17.2)	1; 20 [20–20]	12.7 (12.2–13.2)

*N* number of studies reporting the outcome in more than 1 patient; *n* total number of patients in the studies; *min–max* number of patients in the smallest and the largest study; *I*^*2*^ statistic for assessing the between-study heterogeneity (from 0 (no heterogeneity) to 1 (extreme heterogeneity))

**Table 3:** Descriptive statistics for the angle of insertion of the masseter muscle relative to Frankfort horizontal plane per vertical group

Outcomes	Norm		Hypo		Hyper	
	N, n (min–max)	Pooled mean (95% CI); I^2^	N, n (min–max)	Pooled mean (95% CI); I^2^	N, n (min–max)	Pooled mean (95% CI); I^2^
Masseter angle (°)	3; 103 [9–83]	69.3 (64.2–74.4); I^2^ = 0.95	3; 81 [5–65]	71.4 (69.2–73.5); I^2^ = 0.66	3; 48 [6–34]	65.2 (59.3–71.1); I^2^ = 0.86

*N* number of studies reporting the outcome in more than 1 patient; *n* total number of patients in the studies; *min–max* number of patients in the smallest and the largest study; *I*^*2*^ statistic for assessing the between-study heterogeneity (from 0 (no heterogeneity) to 1 (extreme heterogeneity))

**Table 4:** Descriptive statistics for the width and thickness of the masseter muscle per vertical group

Outcomes	Norm		Hypo		Hyper	
	N, n (min–max)	Pooled mean (95% CI); I^2^	N, n (min–max)	Pooled mean (95% CI); I^2^	N, n (min–max)	Pooled mean (95% CI); I^2^
*All patients*						
Masseter thickness (mm)	9; 202 [4–55]	11.6 (10.4–12.7); I^2^ = 0.98	9; 152 [10–28]	12.9 (11.6–14.1); I^2^ = 0.98	9; 165 [5–32]	10.5 (9.1–12.0); I^2^ = 0.98
Masseter width (mm)	2; 24 [4–20]	41.6 (33.5–49.7); I^2^ = 0.79	1; 20 [20–20]	46.0 (44.2–47.7)	2; 25 [5–20]	39.3 (36.8–41.7); I^2^ = 0.3
Masseter thickness _bite (mm)	7; 183 [10–55]	12.9 (11.6–14.2); I^2^ = 0.97	8; 137 [10–28]	14.7 (13.4–15.9); I^2^ = 0.97	8; 160 [10–32]	12.2 (10.8–13.7); I^2^ = 0.98
Masseter width _bite (mm)	1; 20 [20–20]	40.0 (38.2–41.7)	1; 20 [20–20]	42.0 (40.7–43.3)	1; 20 [20–20]	37.0 (35.7–38.3)
*Males*						
Masseter thickness (mm)	4; 50 [6–26]	13.2 (11.7–14.6); I^2^ = 0.84	4; 38 [4–17]	14.7 (14.0–15.3); I^2^ = 0.39	4; 38 [8–11]	12.2 (9.9–14.5); I^2^ = 0.94
Masseter width (mm)	0; 0		0; 0		0; 0	
Masseter thickness _bite (mm)	4; 50 [6–26]	14.7 (13.7–15.7); I^2^ = 0.63	4; 38 [4–17]	16.1 (15.6–16.7); I^2^ = 0	4; 38 [8–11]	13.7 (11.2–16.3); I^2^ = 0.95
Masseter width _bite	0; 0		0; 0		0; 0	
*Females*						
Masseter thickness (mm)	4; 66 [8–29]	12.1 (11.7–12.6); I^2^ = 0	4; 35 [7–11]	12.8 (11.6–13.9); I^2^ = 0.72	4; 61 [8–24]	11.5 (10.5–12.5); I^2^ = 0.71
Masseter width (mm)	0; 0		0; 0		0; 0	
Masseter thickness _bite (mm)	4; 66 [8–29]	13.5 (13.0–13.9); I^2^ = 0	4; 35 [7–11]	14.5 (14.1–15.0); I^2^ = 0.4	4; 61 [8–24]	12.9 (12.1–13.7); I^2^ = 0.62
Masseter width _bite (mm)	0; 0		0; 0		0; 0	

N: number of studies reporting the outcome in more than 1 patient; n: total number of patients in the studies; min–max: number of patients in the smallest and the largest study; I^2^: statistic for assessing the between-study heterogeneity (from 0 (no heterogeneity) to 1 (extreme heterogeneity))

**Fig. 2:**
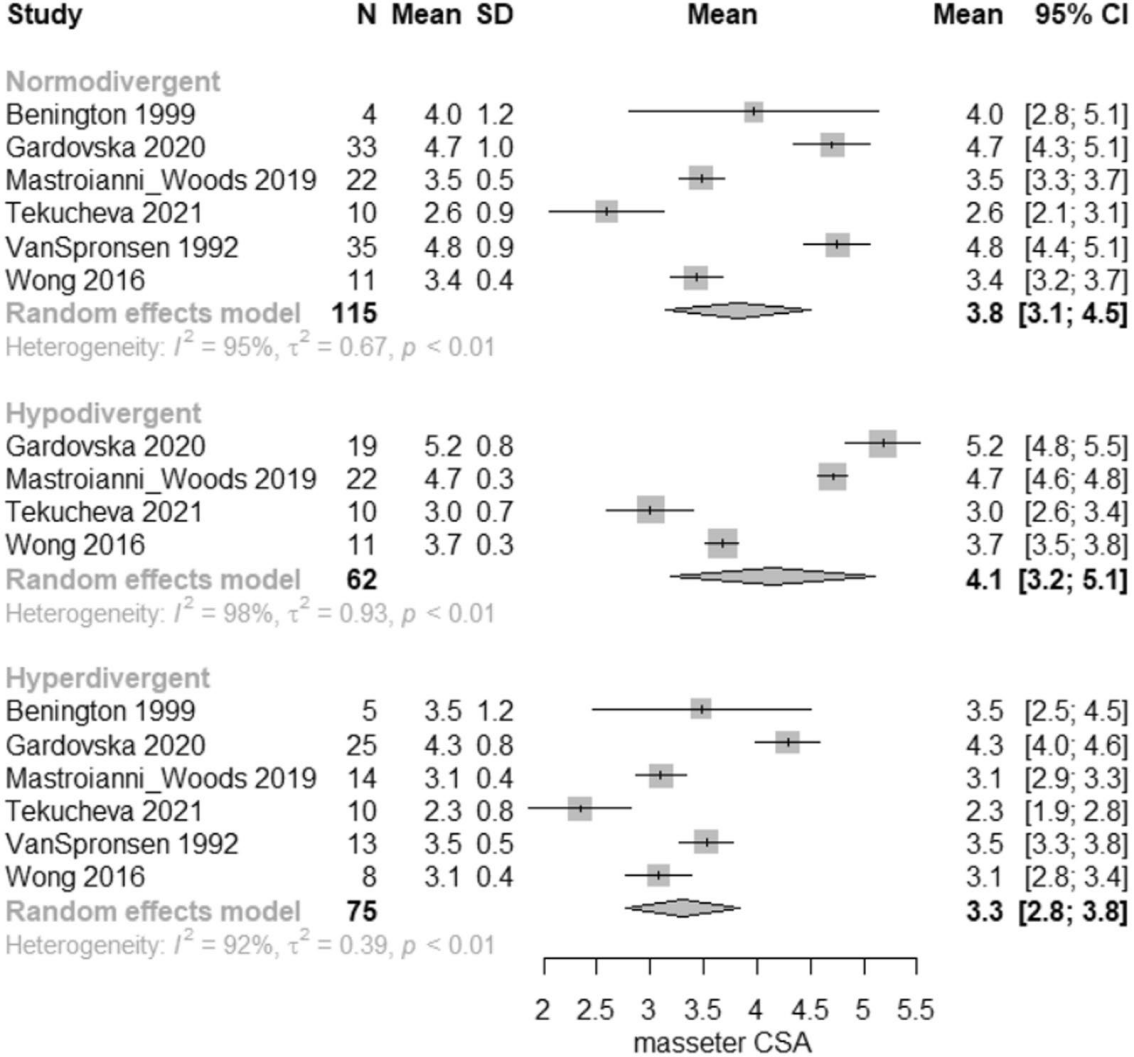
Forest plot of masseter cross-sectional area (CSA) in normodivergent, hypodivergent and hyperdivergent patients

**Fig. 3:**
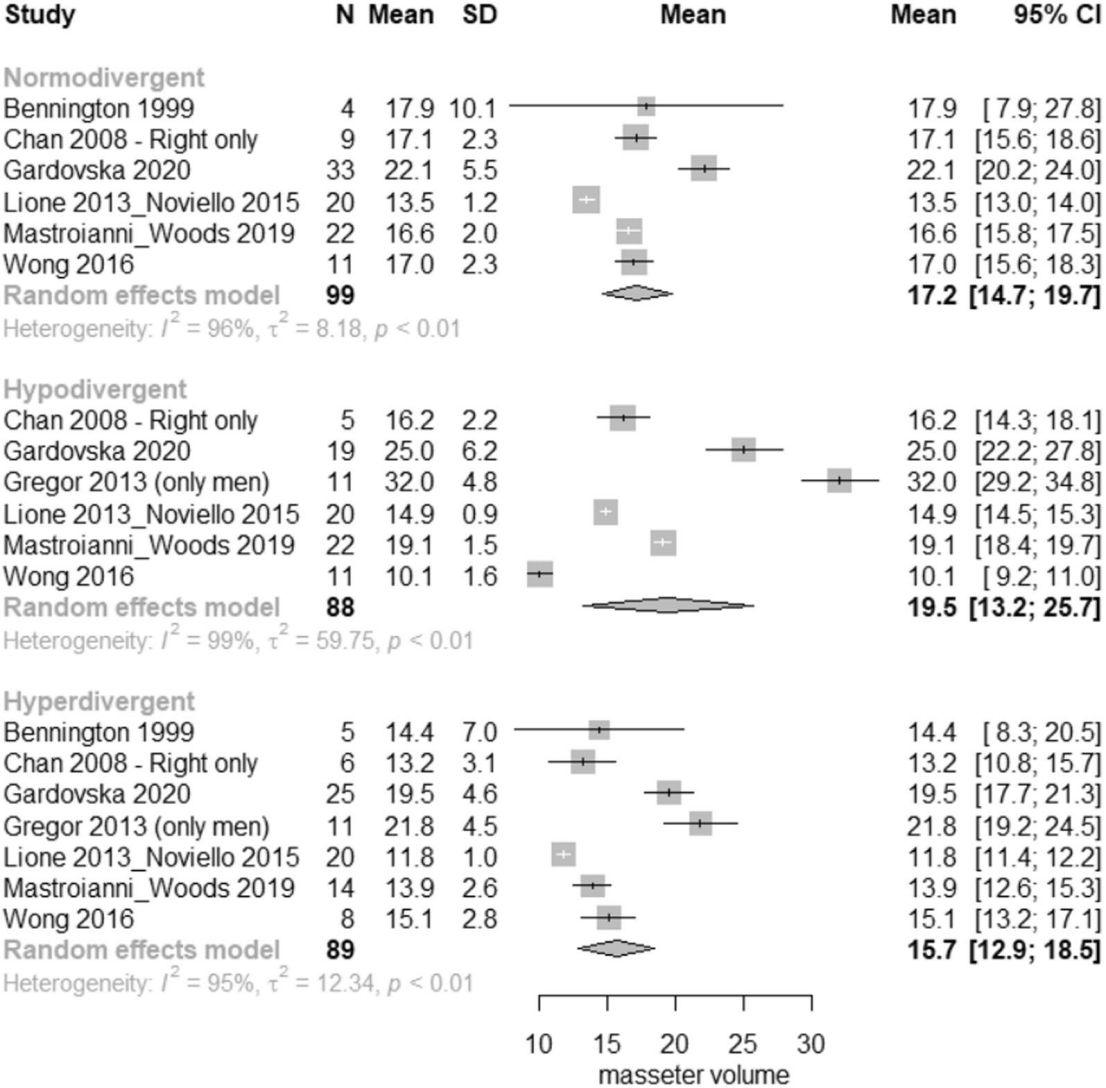
Forest plot of masseter volume in normodivergent, hypodivergent and hyperdivergent patients

**Fig. 4:**
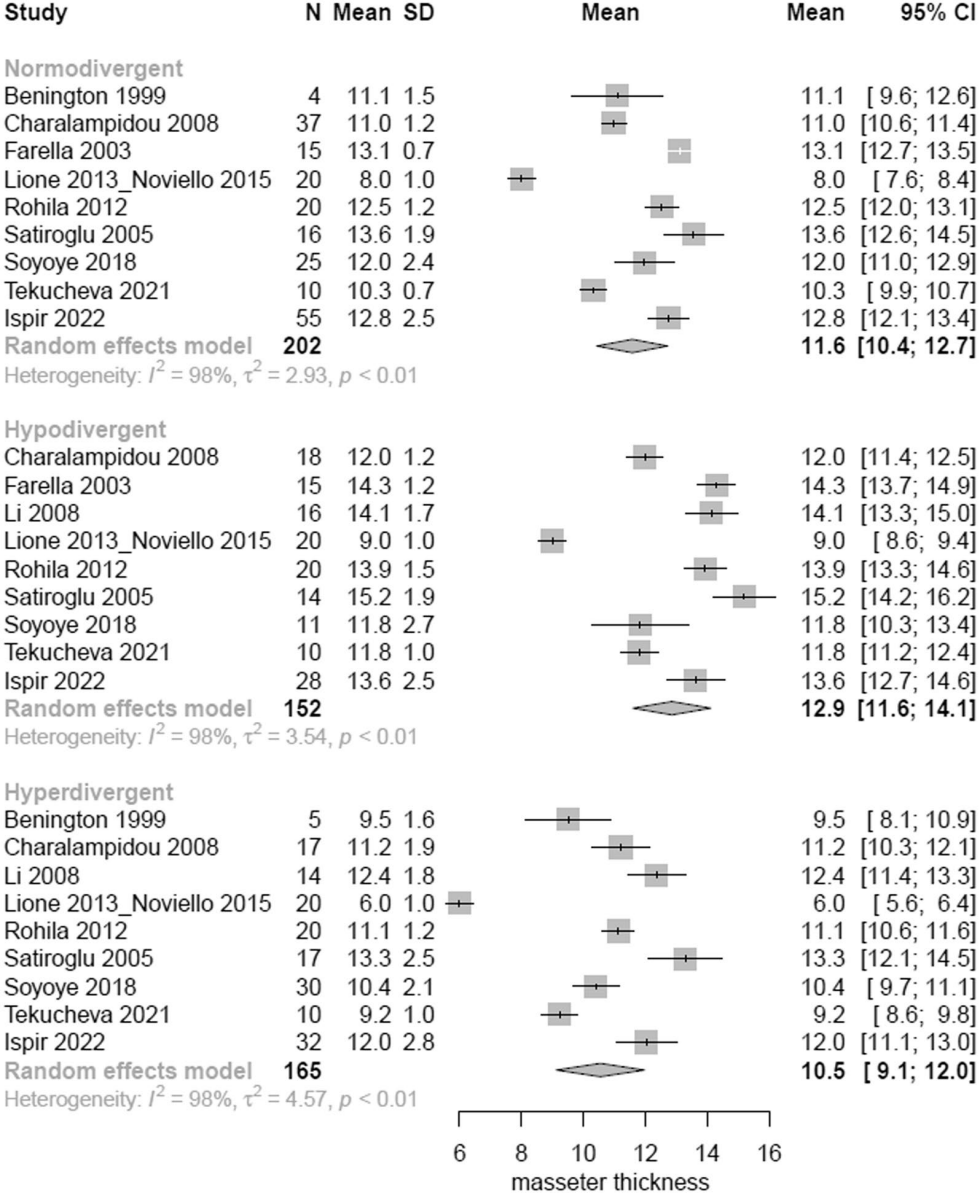
Forest plot of masseter thickness at relaxation in normodivergent, hypodivergent and hyperdivergent patients

**Fig. 5:**
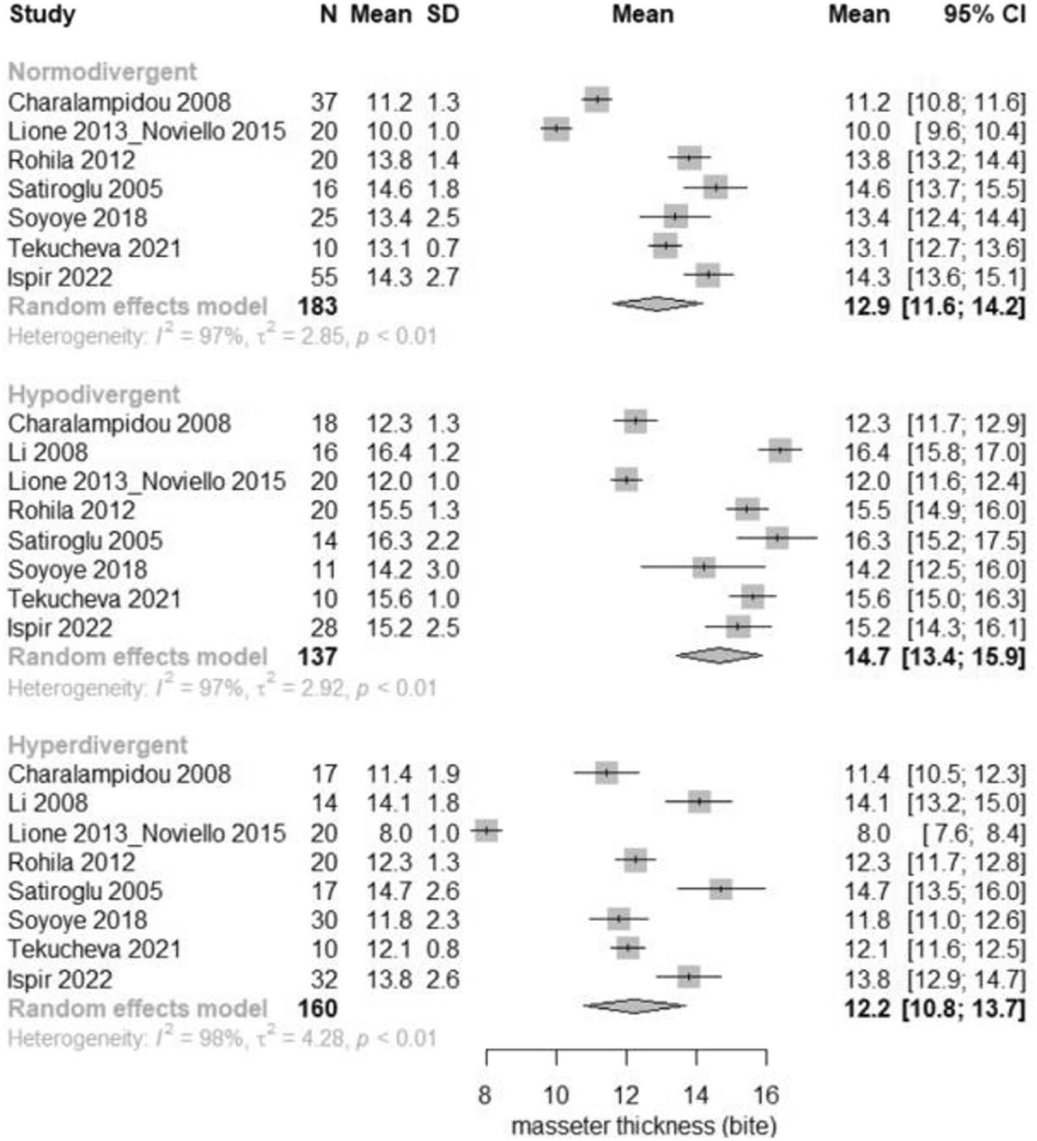
Forest plot of masseter thickness at bite in normodivergent, hypodivergent and hyperdivergent patients

According to the meta-analysis, considerable differences were seen in the masseter muscle cross-sectional area (CSA), volume, thickness in relaxation and thickness in contraction among the three vertical groups. Regarding the comparisons between hypodivergent and normodivergent patients, hypodivergent patients had significantly greater masseter CSA by 0.50 cm^2^ (95% CI 0.05–0.95 cm^2^); volume by 1.65 cm^3^ (95% CI 0.45–2.85 cm^3^); masseter thickness at relaxation by 1.14 mm (95% CI 0.74–1.53 mm); and masseter thickness at contraction by 1.61 mm (95% CI 0.96–2.27 mm). Regarding the comparisons between hyperdivergent and normodivergent patients, hyperdivergent patients had significantly decreased masseter CSA by − 0.54 cm^2^ (95% CI − 0.95 to − 0.12 cm^2^); volume by − 2.64 cm^3^ (95% CI − 3.90 to − 1.38 cm^3^); masseter thickness at relaxation by − 1.14 mm (95% CI − 1.56 to − 0.73 mm); and masseter thickness at contraction by − 1.00 mm (95% CI − 1.65 to − 0.35 mm). Regarding the comparisons between hyperdivergent and hypodivergent patients, hyperdivergent patients had significantly decreased masseter CSA by − 1.04 cm^2^ (95% CI − 1.49 to − 0.59 cm^2^); volume by − 4.29 cm^3^ (95% CI − 5.52 to − 3.06 cm^3^); masseter thickness at relaxation by − 2.28 mm (95% CI − 2.71 to − 1.85 mm); and masseter thickness at contraction by − 2.61 mm (95% CI − 3.26 to − 1.97 mm) (Table 5, Figs. 2, 3, 4, and 5).

**Table 5:** Pairwise comparisons of masseter muscle cross-sectional area (CSA), volume, thickness at relaxation and at maximal bite per vertical categorization

Outcome	Hypo versus Norm*		Hyper versus Norm**		Hyper versus Hypo***		Heterogeneity**** (*p* value)	Tau^2^	Overall *p* value*****
	Pooled MD (95%CI)	*p* value	Pooled MD (95%CI)	*p* value	Pooled MD (95%CI)	*p* value			
Masseter CSA (cm^2^)	0.50 (0.05–0.95)	0.0289	− 0.54 (− 0.95 to − 0.12)	0.0110	− 1.04 (− 1.49 to − 0.59)	< 0.0001	< 0.0001	0.61	0.0002
Masseter volume (cm^3^)	1.65 (0.45–2.85)	0.0070	− 2.64 (− 3.90 to − 1.38)	< 0.0001	− 4.29 (− 5.52 to − 3.06)	< 0.0001	< 0.0001	21.32	< 0.0001
Masseter thickness (mm)	1.14 (0.74–1.53)	< 0.0001	− 1.14 (− 1.56 to − 0.73)	0.0001	− 2.28 (− 2.71 to − 1.85)	< 0.0001	< 0.0001	3.38	< 0.0001
Masseter thickness_bite (mm)	1.61 (0.96–2.27)	< 0.0001	− 1.00 (− 1.65 to − 0.35)	0.0026	− 2.61 (− 3.26 to − 1.97)	< 0.0001	< 0.0001	3.36	< 0.0001

Statistically significant results at the 5% level

*MD* mean difference, *CI* confidence interval

*Positive mean difference = mean higher in hypo than in norm

**Positive mean difference = mean higher in hyper than in norm

***Positive mean difference = mean higher in hyper than in hypo

****Residual heterogeneity

*****For testing the equality of the mean in the three groups

A leave-one-out sensitivity analysis was conducted for each measured dimension. When the study by Tekucheva et al. [40] was removed from the analysis of CSA, the between-study variability (Tau^2^) decreased from 0.61 to 0.37. However, the heterogeneity was still statistically significant (*p* < 0.0001), and the difference between groups remained significant (*p* < 0.0001). The same occurred when excluding the study by Gregor et al. [41] (which comprises a pool of male patients only); the between-study variability (Tau^2^) decreased from 21.32 to 8.43, but the heterogeneity was still significant (*p* < 0.0001), and the difference between groups remained statistically significant (*p* < 0.0001). Regarding muscle thickness, the studies by Lione et al. [42] and Noviello et al. [43] (composed of the same sample of patients) reported lower values than other studies. If these are excluded from the statistical analysis (both for the resting and contraction conditions), Tau^2^ decreased from 3.36 to 1.69. However, the heterogeneity and difference between groups remained statistically significant (*p* < 0.0001).

Fourteen studies categorized the included patients according to their sagittal skeletal relationships as Class I, II and III. Due to heterogeneity and incomplete reporting of the outcomes, meta-analysis could not be performed. Ariji et al. [44] compared the masseter muscle angle of insertion and CSA between Class I and Class III patients and found that the inclination was significantly decreased, and the CSA significantly increased in the Class I group. Another study [45] reported the anatomical characteristics (length and angle) of the masseter muscle in the three different classes of patients (Class I; II; III). Their results revealed that the most acute orientation angle (67.2 ± 6.6°) was found in Class II subjects, while the most obtuse orientation angle (81.6 ± 6.8°) was observed in Class III group; however, no significant differences were found in muscle length among the three groups. Kim et al. [46] reported masseter, medial pterygoid, lateral pterygoid and temporalis muscle thickness separately in males and females in class I and class III patients. There was a significant negative correlation only between master muscle thickness in Class III patients and the ANB angle. Kim et al. [47] compared masseter muscle volume/length ratio between Class I and Class III patients and this was significantly greater in Class I patients. Rani and Ravi [32] report the masseter thickness of class I patients and class II patients, making a distinction between patients with maxillary excess and patients with mandibular growth deficiency. The masseter thickness of Class I patients was similar to Class II patients with maxillary excess but class II patients with mandibular deficiency showed thinner masseter thicknesses. Zepa et al. [48] compared the anatomical characteristics (CSA; thickness; volume; length; and width) of the masseter and medial pterygoid muscles between Class II and Class III patients. In Class III patients, there was a tendency for all masseter variables to be higher; however, they did not reach statistical significance. On the contrary, the volume and the thickness of the medial pterygoid muscles were significantly greater in the Class III patients compared to Class II patients.

Five studies reported only correlations between muscular anatomic characteristics and sagittal skeletal classification with mainly insignificant findings (Supplementary Table 7). The certainty of the evidence according to the GRADE rating was judged as being very low, and the reasons for downgrading were study design (observational, cross-sectional), individual study limitations due to high risk of bias, inconsistency of the results due to great heterogeneity, and imprecision due to small sample sizes and wide CIs (Supplementary Tables 10–12).

## Discussion

### General interpretation of the results in the context of other evidence

The results of the present systematic review and meta-analysis show significant differences in the parameters related to masseter muscle volume, CSA, width and thickness across different groups of patients categorized by their facial vertical divergence. In general, it was shown that hyperdivergent patients had smaller muscles for all analysed outcomes compared to normodivergent and hypodivergent patients while normodivergent patients had smaller muscles compared to hypodivergent patients. These results underpin a possible association between masticatory muscular characteristics and vertical craniofacial morphology; however, the magnitude of the differences was small and the certainty of the evidence very low.

Alternative approaches for assessing the functional capacity of masticatory muscles have been explored concerning vertical craniofacial morphology, yielding largely consistent findings. Studies have demonstrated a negative association between vertical facial dimensions and maximal bite force as well as electromyographic activity of masticatory muscles [49, 50]. Similarly, findings from computer tomography (CT) investigations align with these results, indicating a negative correlation between the mandibular plane angle and parameters such as masseter muscle thickness and length [51].

It has been previously reported that muscular anatomic characteristics are also reflected in the forces they are capable to exert with thicker muscles being able to deliver greater mechanical stresses on the underlying skeletal bone structures [52, 53]. Similarly, the masticatory muscles and their constraints exerted on facial bone structures considerably influence the face in general and mandibular shape [54, 55]. Additionally, it has been found that women with thinner muscles have longer faces while subjects with muscular dystrophies and subsequently weaker muscles attain a hyperdivergent growth pattern, which in turn indicates a relationship between aberrations in muscular characteristics and deviations in craniofacial morphology [20, 56].

The distribution of muscle fibres and their molecular structure also differs between vertical and horizontal growers. Studies have shown that the more hyperdivergent the patients, the more type I muscle fibres (slow fibres) are found, while in hypodivergent patients type II fibres (fast fibres) are more numerous [57, 58]. The correlation between sagittal jaw relationships and mean fibre area was found to be less evident; however, within Class III subjects, those with a deep bite exhibited a notable rise in type I and I/II hybrid fibres while polymorphism in the MYO1H gene was linked to an elevated susceptibility to mandibular prognathism and horizontal maxillomandibular discrepancies irrespective to the ethnic background [57, 59]. Associations between fibre-type distribution and biochemical composition of the masticatory muscles and how these can relate to skeletal craniofacial patterns however warrant further investigation.

### Limitations of the evidence included in the review and the review process

Even though the present protocol was pre-registered, and an exhaustive literature search was performed, this systematic review also comes with some limitations relevant mainly to its results and the high risk of bias of the included studies. Issues related to bias are pertinent to study size (sample size), inconsistencies in the cut-off values of the cephalometric parameters used to categorize the patients into sagittal and/or vertical groups, unclear information on whether the assessor of the muscles was trained and blinded to the cephalometric variables and incomplete reporting of the samples and results.

Various cofounding factors were also not taken into consideration and accounted in the analyses in the included studies such as patients’ age and sex. Several studies included only males in their sample, naturally presenting larger muscle dimensions than those of women [24, 26, 41, 60]. Given that outcomes related to muscular characteristics differ between vertical groups, such subcategorization should be accounted for when grouping the patients per sagittal group. Additionally, age also varied greatly between the samples of different studies. Knowing that muscular bite force differences are not evident between hyperdivergent and normodivergent patients during childhood but only after adulthood, carrying out analyses without accounting for age can bias the results, however, such sub-group analyses were not possible due to incomplete reporting of study samples and results [13].

It could be advocated that differences in image acquisition could impart the accuracy of the measurements; however, the studies that contributed to the meta-analyses assessed muscular characteristics primarily by using ultrasonography. Moreover, studies show no significant differences between different methods such as Cone-Beam CT, MRI scans and ultrasonography for measuring and analysing muscle characteristics [61, 62].

When considering masticatory muscle size assessments, those made under relaxed conditions are known to be less reproducible due to the fact that the relaxed muscles are more susceptible to the pressure with which the transducer is positioned against the cheek, and thus is very technique sensitive [31, 63–65]. Measurements made under contracted conditions (with the patient biting) are thus preferred when assessing masticatory muscle thickness characteristics.

Finally, the overall high risk of bias of the included studies precludes us from drawing robust conclusions based on the current available evidence.

### Implications of the results for practice, policy, and future research

These results highlight the importance of masticatory muscles in shaping the skeletal structures of the face. Even though in the past orthodontic treatment was basically focused on dental relationships, the current trends are more towards face-oriented orthodontic treatments [66]. Additionally, muscular anatomic characteristics have been reported to influence response to orthodontic treatment [67]. More specifically, the initial thickness of the masseter muscles in patients treated with functional appliances has been shown to influence treatment outcome. There was greater posterior displacement of the cephalometric point A in patients with thinner masseter muscles but also greater mandibular incisor proclination [68]. After undergoing functional appliance therapy, children exhibiting more pronounced dentoalveolar changes (thinner masseter muscles) may also demonstrate an increased likelihood of sagittal relapse post-treatment [69].

Although the current scientific evidence on the role of masticatory muscles on craniofacial patterns is weak, it could highlight an additional approach to patient care by incorporating factors related to muscular characteristics in baseline diagnosis. This approach considers the physiological aspects of a malocclusion, establishing the biological limits within which a practitioner can work. This applies to all treatment methods, whether conventional, combined with orthognathic surgery, using various appliances and biomechanics, or involving retention strategies for maintaining long-term stability.

With the present systematic review and meta-analysis, an attempt was made to gather the available evidence regarding the differences in anatomic masticatory muscle characteristics and craniofacial patterns as categorized in the sagittal or vertical dimensions using cephalometry. Given the uncertainty of the evidence though regarding the differences found in the present review, it is recommended that further high-quality prospective studies are conducted to expand the available evidence in this field.

## Conclusions

Based on the studies included in our systematic review and meta-analysis, masseter muscle volume, cross-sectional area, width and thickness (under both relaxation and contraction) were significantly decreased in hyperdivergent patients compared to normodivergent and hypodivergent while the same parameters were significantly increased in hypodivergent patients compared to normodivergent patients. These results should be interpreted with caution because the scientific evidence from primary studies is weak with a high risk of bias.

## Supplementary Information

Additional file1 (DOCX 1154 KB)

Additional file2 (DOCX 464 KB)
