# Correction to: Relationship between craniofacial skeletal patterns and anatomic characteristics of masticatory muscles: a systematic review and meta‑analysis

**DOI:** 10.1186/s40510-025-00593-z

**Published:** 2025-11-13

**Authors:** David Togninalli, Gregory S. Antonarakis, Alexandra K. Papadopoulou

**Affiliations:** 1 Division of Orthodontics, University Clinics of Dental Medicine, Faculty of Medicine, University of Geneva, Geneva, Switzerland; 2 Discipline of Orthodontics and Paediatric Dentistry, Sydney Dental School, Faculty of Medicine and Health, The University of Sydney, Sydney, Australia

**Correction to: Progress in Orthodontics (2024) 25:36**

10.1186/s40510-024-00534-2.

Following publication of the original article [1], the authors identified an error in the units that were used to present the cross-sectional areas (CSAs) of the muscles.

Wherever in Tables 1 and 5 and in the Results section the CSAs of the muscles are presented, these should read as cm^2^ and not as mm^2^.

It earlier read:

- Tables 1 and 5 earlier read:

- Table 1, CSAs of the muscles (masseter, lateral pterygoid, medial pterygoid, temporalis, masseter_bite) are presented in mm^2^.
- Table 5, CSA of the masseter muscle is presented in mm^2^.

- The “Results: Results of individual studies and data synthesis” earlier read:

Regarding the comparisons between hypodivergent and normodivergent patients, hypodivergent patients had significantly greater masseter CSA by 0.50 mm^2^ (95% CI 0.05 to 0.95 mm^2^); volume by 1.65 cm^3^ (95% CI 0.45 to 2.85 cm^3^); masseter thickness at relaxation by 1.14 mm (95% CI 0.74 to 1.53 mm); and masseter thickness at contraction by 1.61 mm (95% CI 0.96 to 2.27 mm). Regarding the comparisons between hyperdivergent and normodivergent patients, hyperdivergent patients had significantly decreased masseter CSA by − 0.54 mm^2^ (95% CI − 0.95 to − 0.12 mm^2^); volume by − 2.64 cm^3^ (95% CI − 3.90 to − 1.38 cm^3^); masseter thickness at relaxation by − 1.14 mm (95% CI − 1.56 to − 0.73 mm); and masseter thickness at contraction by − 1.00 mm (95% CI − 1.65 to − 0.35 mm). Regarding the comparisons between hyperdivergent and hypodivergent patients, hyperdivergent patients had significantly decreased masseter CSA by − 1.04 mm^2^ (95% CI − 1.49 to − 0.59 mm^2^); volume by − 4.29 cm^3^ (95% CI − 5.52 to − 3.06 cm^3^); masseter thickness at relaxation by − 2.28 mm (95% CI − 2.71 to − 1.85 mm); and masseter thickness at contraction by − 2.61 mm (95% CI − 3.26 to − 1.97 mm) (Table 5; Figs. 2, 3, 4 and 5).

It currently reads:

- Tables 1 and 5 currently reads:

- Table 1, CSAs of the muscles (masseter, lateral pterygoid, medial pterygoid, temporalis, masseter_bite) should read in cm^2^.
- Table 5, CSA of the masseter muscle should read in cm^2^.

- The “Results: Results of individual studies and data synthesis” currently read:

Regarding the comparisons between hypodivergent and normodivergent patients, hypodivergent patients had significantly greater masseter CSA by 0.50 cm^2^ (95% CI 0.05 to 0.95 cm^2^); volume by 1.65 cm^3^ (95% CI 0.45 to 2.85 cm^3^); masseter thickness at relaxation by 1.14 mm (95% CI 0.74 to 1.53 mm); and masseter thickness at contraction by 1.61 mm (95% CI 0.96 to 2.27 mm). Regarding the comparisons between hyperdivergent and normodivergent patients, hyperdivergent patients had significantly decreased masseter CSA by − 0.54 cm^2^ (95% CI − 0.95 to − 0.12 cm^2^); volume by − 2.64 cm^3^ (95% CI − 3.90 to − 1.38 cm^3^); masseter thickness at relaxation by − 1.14 mm (95% CI − 1.56 to − 0.73 mm); and masseter thickness at contraction by − 1.00 mm (95% CI − 1.65 to − 0.35 mm). Regarding the comparisons between hyperdivergent and hypodivergent patients, hyperdivergent patients had significantly decreased masseter CSA by − 1.04 cm^2^ (95% CI − 1.49 to − 0.59 cm^2^); volume by − 4.29 cm^3^ (95% CI − 5.52 to − 3.06 cm^3^); masseter thickness at relaxation by − 2.28 mm (95% CI − 2.71 to − 1.85 mm); and masseter thickness at contraction by − 2.61 mm (95% CI − 3.26 to − 1.97 mm) (Table 5; Figs. 2, 3, 4 and 5).

The Tables 1 and 5 and the Results section have been updated above and the original article [1] has been corrected.
