# Supplementary material for: Relationship between craniofacial skeletal patterns and anatomic characteristics of masticatory muscles: a systematic review and meta-analysis
Source: Prog Orthod. 2024 Sep 9;25:36. doi: 10.1186/s40510-024-00534-2 (PMC11381490; doi:10.1186/s40510-024-00534-2)

**Title:** RELATIONSHIP BETWEEN CRANIOFACIAL SKELETAL PATTERNS AND ANATOMIC CHARACTERISTICS OF MASTICATORY MUSCLES: A SYSTEMATIC REVIEW AND META-ANALYS.

**Supplementary Table 1.** Eligibility criteria used for the study selection.

| **Category** | **Inclusion criteria** | **Exclusion criteria** |
| --- | --- | --- |
| Participant characteristics | Humans (healthy). | Animal studies.  Humans with genetic or other acquired pathology (clefts, syndromes, craniofacial trauma). |
| Exposure | Assessment of the face muscles using three-dimensional imaging technology.  This would include magnetic resonance imaging (MRI), Computed tomography (CT), Cone- Beam Computed Tomography (CBCT) and ultrasonography (US) for the evaluation of muscle anatomic parameters/characteristics.  Craniofacial pattern categorization in anteroposterior-sagittal (Class I, II, III) and vertical (normodivergent, hypodivergent hyperdivergent) groups based on lateral cephalometry, either conventional 2D or reconstructed from 3D data. | Assessment of the face muscles using methods other than the ones mentioned in the inclusion criteria.  Assessment of facial craniofacial pattern with methods other than lateral cephalometry such as clinical examination, photographs, dental occlusion. |
| Comparison | Class I (controls) craniofacial pattern versus Cl II/III and normodivergent (controls) craniofacial patterns versus hyper/hypodivergent patients. Comparison of the face muscle anatomic characteristics amongst groups with different sagittal and vertical growth patterns. | Comparison of treatment effects on face muscles |
| Outcome | Primary outcome: muscle thickness (depth)  Secondary outcomes: muscle width, height (length), cross-sectional area, volume, inclination (angle of insertion: the angle formed between the masseter muscle long axis and FH plane indicating its orientation. | Studies not providing the outcomes of interest |
| Study design | Randomized and non-randomized prospective or retrospective clinical studies  Cross-sectional prospective or retrospective studies | Case reports  Review articles (references checked),  Unsupported opinions  Interviews & newsletters  Commentaries  Conference abstracts  Replies to the editor/author |

**Supplementary Table 2.** Search Strategy.

| **Database (all of them in “advanced search”)** |  | **Extend, Limits, Sum** |
| --- | --- | --- |
| **Pubmed**  <https://pubmed.ncbi.nlm.nih.gov/> | ((((masseter OR mastic*) AND muscle*) AND (((("cone beam" OR digital OR ct) AND (tomograph* or radio* OR scan* OR imag*)) OR (CBCT OR CBVT OR "computed tomography" OR "computerized tomography" OR "magnetic resonance imag*" OR DVT OR MRI OR 3D OR three-dimens* OR ultraso* OR Cephalometr* OR Photogrammetr* OR Stereogrammetr* OR Stereophotogrammetr* OR Elecromyograph*)) OR ((bite OR biting OR occlusal) AND (force* OR load*)))) AND (Craniofac* OR Dentocraniofac* OR Dentofac* OR Maxillofac* OR Skelet* OR Malocclusion* OR "Angle Class*" OR Grow* OR Prognath* OR Retrognath* OR Hypodivergen* OR Hyperdivergen* OR Normodivergen* OR Divergen* OR Brachyfac* OR Dolychofac* OR "short face" OR "long face")) NOT (syndrom* OR cleft*) | **In: Title, abstract**  **Limit: humans**  Results: 398 |
| Embase  [www.embase.com](http://www.embase.com) | Same as Pubmed | In: All fields  Quick limits: human  Limit Embase  Results: 985 |
| Web of Science  <https://www.webofscience.com/>  Web of Science Core Collection | Same as Pubmed | In: Topic  Results: 946 |
| ScielLo (via Web of Science)  <https://www.webofscience.com/wos/scielo/advanced-search> | Same as Pubmed | **In: Topic**  **Results: 14** |
| Scopus  [www.scopus.com](http://www.scopus.com) | Same as Pubmed | In: Title, Abstract, Key words  Limit: Human, Humans  Results: 1408 |
| Proquest (Dissertations and Theses)  (via Web of Science)  <https://www.webofscience.com/wos/pqdt/advanced-search> | Same as Pubmed | In: Topic  Results: 59 |
| Cochrane Library  [www.cochranelibrary.com](http://www.cochranelibrary.com) | Same as Pubmed | In: Title Abstract Keyword  (Word variations searched)  Results: 51 |
| Virtual Health Library  <http://pesquisa.bvsalud.org/portal/advanced/?lang=en> | Same as Pubmed | In: Title, Abstract, Subject  **LILACS: 5**  **BBO-Dentistry: 4**  **IBECS: 1**  **Deduplicated results: 7** |
|  | **Total prior to deduplication** | **3868** |
|  | **Total after deduplication** | **2520** |

Supplementary Table 3. Data extraction form.

| **Item** |
| --- |
| Study Name (author, year, clinical setting): |
| Study design:  Purpose of study (if stated):  Ethics approval (Yes/No/Unclear)  Funding (if stated): |
| **PATIENTS GROUPS inclusion & exclusion criteria, demographics (age, sex), cephalometric cut-off points for sagittal and vertical groups.**  **Inclusion Criteria**  **Exclusion criteria**  Class I  Class II  Class III  Normodivergent  Hypodivergent  Hyperdivergent |
| **INTERVENTIONS (treatment & comparison group)**  **Type of 3D analysis (**magnetic resonance imaging (MRI), Computed tomography (CT), Cone- Beam Computed Tomography (CBCT) and ultrasonography (US) for the evaluation of muscle anatomic parameters/characteristics.)  Patient positioning during muscle assessment. STANDING/SUPINE  Instructions during muscle assessment: relaxed / at maximal biting  Patient positioning during image acquisition. STANDING/SUPINE  Instructions during image acquisition. YES/NO  Equipment type used for muscle assessment. |
| **OUTCOMES**  **Measurements**  **Muscle: any type of primary mastication muscles**  muscle thickness (depth)  muscle width  muscle height (length)  muscle cross-sectional area  muscle volume  muscle inclination |
| **Other Measurements:** |
| **Statistical methods** (appropriateness; if relevant) |
| **Error Assessment:** |
| **Other comments:** |

**Supplementary Table 4.** Domains / questions of the customized tool used for the assessment of internal validity / risk of bias. Each question was answered with yes, unclear, or no.

| **Nr** | **Question** |
| --- | --- |
| 1 | Were the aims/objectives of the study clear? |
| 2 | Were the criteria for inclusion in the sample clearly defined? |
| 3 | Were the study subjects and the setting described in detail (time, location, demographics)? |
| 4 | Was the sample size justified (sample size calculation)? |
| 5 | Were objective, standard criteria used for measurement of the condition (cut-off values for cephalometric parameters used to categorize the patients in the sagittal and vertical groups)? |
| 6 | Were confounding factors identified (age, sex, skeletal maturation stage, body mass index-BMI, vertical sub-categorization for the sagittal groups and vise-versa, transversal discrepancies, asymmetries, cross-bites, functional shifts, use of linear or curved ultrasonographic probe, conditions during image acquisition such as providing patients with instructions)? |
| 7 | Were strategies to deal with confounding factors stated (analyses adjusted for confounders)? |
| 8 | Were the outcomes measured in a valid way (validity of the method used to measure the outcomes of interest)? |
| 9 | Were the outcomes measured in a reliable way (experience and/or training and/or calibration of the one-s performing muscular measurements)? |
| 10 | Method error calculation performed? |
| 11 | Were the assessors blinded to the groups (the one-s measuring the muscles being aware of the cephalometric values)? |
| 12 | Was appropriate statistical analysis used? |
| 13 | Is it clear what was used to determined statistical significance and/or precision estimates (p values, confidence intervals, etc.)? |
| 14 | Were all specified outcome data adequately described and reported (incomplete reporting)? |

Supplementary Table 6. Study Design, Method used for muscular assessment and software, patients, inclusion and exclusion criteria of the sample groups. Patients (group description). Measurement Techniques & Software

| **Study** | **Study design**  **Institution** | **Method used for muscle assessment, equipment**  **(scanner and software), instructions during image acquisition & muscle function (relaxed-max.bite)** | **Sample (number, age, sex)**  **Sagittal subgroups**  **Cut-off points for diagnosis**  **Class I**  **Class II**  **Class III** | **Sample**  **Vertical subgroups**  **Cut-off points for diagnosis**  **Normodivergent (AV)**  **Hypodivergent (LA)**  **Hyperdivergent (HA)** | **Inclusion criteria** | **Exclusion criteria** |
| --- | --- | --- | --- | --- | --- | --- |
| Ariji et al. 2000 | Prospective (test)  Retrospective (controls)  Aichi-Gakuin University  Japan | CT  (Somatom Art, Simens AG, Germany and X-Vision, Toshiba, Japan)  Voxel view (vital Images, Inc., Tokyo, Japan)  Med Vision (Innet Systems, Castine, ME, USA)  Position: NR  Instructions: NR  Muscle: NR | Class I  N: 91  Males: 49  Females: 42  Class III Md prognathism  N: 69  Males: 31  Females: 38  Age Class I: 24.2±3.5  Age Class III Md pr: 22.5±3.8  Cut-off points : NR | No grouping | Class I  Class III with Md prognathism  Both masseter muscles scanned  Muscles asymptomatic and free of pathology | Mandibular prognathism with asymmetry |
| Azaroual et al. 2014 | Retrospective  Université Mohamed-V Souissi  Morocco | CT  (Siemens 32-row 64 slice spiral CT-scan)  Software: NR  Position: NR  Instructions: mouth closed  Muscle: only right side for measurements | No grouping | N : 40  Males : 21  Females : 19  Age : 40.9±12.8  Cut-off points : NR | NR | NR |
| Becht 2009 (Thesis)  Becht et al. 2014 | Retrospective  West Virginia University School of Dentistry  USA | CT  Dolphin Imaging 10.5 Premium (Dolphin Imaging, Chatsworth, CA)  Position: NR  Instructions: NR  Muscle: NR | N: 182  Males: NR  Females: NR  Age: NR  Cut-off points:  Class I: ANB 0-5°  Class II: ANB >5°  Class III: <0° | N:182  Males: NR  Females: NR  Age: NR  Cut-off points:  AV: FH/MP 22-30°  LA: FH/MP <22°  HA: FH/MP >30° | Permanent dentition  Patient seeking comprehensive orthodontic treatment  Good quality DICOM file images | NR |
| Benington et al. 1999 | Prospective  Glasgow Dental Hospital and School,  University College London and University of London  UK | 3D ultrasonography  (Acuson 128^TM^, Acuson Corporation, Mountain View, CA) with a 38-mm wide, 7 MHz linear probe  Software: NR  Position: supine position  Instructions: to keep their heads still and clench their back teeth together during scan registration  Muscle : NR | N: 10  Males: 4  Females: 6  Age: NR  Males: 20.11 to 26.7  Females: 15.7 to 31.8  Cut-off points: NR | N: 10  Males: 4  Females: 6  Age: NR  Males: 20.11 to 26.7  Females: 15.7 to 31.8  Cut-off points: NR | Patients attending the orthognatic surgery  Post-adolescent  Intact dentition | Jaw asymmetries  History of orthodontic treatment or orthognathic surgery |
| Biondi et al. 2016 | Prospective  University of Insubria and University of Pavia  Italy | MRI  (Toshiba Vantage 1.5 Tesla, Toshiba Medical System SRL, Rome, Italy) for masseter volume  Ultrasonography  (7.5 MHz linear transducer, Toshiba Power-Vision 6000 Ultrasound System, Toshiba Medical System SRL, Rome, Italy) for masseter thickness  Software: NR  Position: supine position (MRI)  Instructions: to maintain slight interocclusal contacts, then to clench maximally  Muscle: RS and MVC | No grouping | N: 61  Males: 30  Females: 31  Age: 11.5  Cut-off points:  AV: FH/MP 21-28°  LA: FH/MP <20°  HA: FH/MP >29° | NR | Previous orthodontic treatment  Bad habits  Oro-facial disorders  Skeletal malocclusion (Cl II or III)  Dental malocclusion (Cl II or III)  Maxillo-facial asymmetries  Transversal discrepancies |
| Boom et al. 2008 | Prospective  Free University Medical Centre Amsterdam and University Medical Center Groningen  The Netherlands | MRI  (1.0 T system – Siemens) to assess muscles CSA and volume  View-box (dHal software, Kifissia, Greece)  Customized software (Visian) and 3D Doctor (able Software Corp., Lexington, USA)  Position: supine position (MRI)  Instructions: hold their teeth together but avoid clenching  Muscle: NR | No grouping | N: 31  Males: 9  Females: 22  Age: 24.3±9.0  Cut-off points: NR | NR | NR |
| Capaccioli et al. 1998 | Prospective  Università di Firenze  Italy | Ultrasonogrpahy  (5 MHz and 7.5 MHz probes)  Position: supine position  Instructions: to turn the head to the opposite side of the instrumentation  Muscle: RS | No grouping | N: 26  Males: 10  Females: 16  Age: 9 to 13 years  Cut-off points: NR | NR | NR |
| Chan et al. 2008 | Prospective  University of Melbourne  Australia | CT  (SOMATOM Sentation 16, Siemens Medical Solutions AG, Erlangen, Germany)  Syngo CT software (Siemens Medical Solutions AG)  Position: NR  Instructions: teeth brought together lightly in natural condylar position  Muscle: NR | No grouping | N: 20  Males: 12  Females: 8  Age: 8.6 to 15.1 (mean 11.9)  Cut-off-points:  AV: FH/MP 22-28°  LA: FH/MP <22°  HA: FH/MP >28° | NR | No obvious facial asymmetry or malformation |
| Charalampidou et al. 2008 | Retrospective or  Gothenburg University, Sweden  University of Geneva  Switzerland | Ultrasonography  (Pie Medical Scanner 480, 7.5 MHz linear array transducer)  Position: upright position without lining on a headrest  Instructions: NR  Muscle: RS and MVC | No grouping | M: 72  Males: 36  Females: 36  Age: 8.5 to 9.5  Cut-off points: NR | Any dental or skeletal malocclusion  Available lateral cephalometric radiograph for each subject  Presence of the 1^st^ permanent molars and least the 2^nd^ primary molars | Previous orthodontic treatment  Jaw asymmetries or craniofacial and TMJ disorders  Congenital or developmental anomalies of the lips, mouth or face |
| Farella et al. 2003 | Prospective  University of Naples and University of Copenhagen  Italy and Denmark | Ultrasonography  (Linear array probe, Logiq 500, General Electric Medical System, Buc, France, 10 MHz, length 70 mm)  Position: sitting on a chair with headrest  Instructions: teeth in slight contact  Muscle: RS and MVC | No grouping | N: 30  Males: 30  Females: 0  Age: NR  Cut-off-points:  AV: FH/MP ≥23°  LA: FH/MP <15°  HA : incl. in the AV group | NR | Acute or chronic inflammatory processes in the orofacial region  Temporomandibular disorders according to the RDC criteria  Current or prior orthodontic treatment  Sports activities requiring frequent training |
| Gardovska et al. 2020 | Retrospective or Prospective is unclear  Riga Stradins University, Riga  Latvia | MRI  (1.5T GE Medical Systems Sign HDx)  Software: NR  Position: supine with FHP oriented perpendicular to the floor  Instructions: mouth closed with teeth together, short and calm breathing  Muscle: NR | M: 76  Males: NR  Females: NR  Age: 20.8 (3.17)  Cut-off points:  Class I: ANB 0-3°  Class II: ANB ≥4°  Class III: <0° | M: 76  Males: NR  Females: NR  Age: 20.8 (3.17)  Cut-off-points:  AV: SN/MP 270-370°  LA: SN/MP <270°  HA: SN/MP >370° | ANB ≥4° for Class II  ANB <0° for Class III  Dentoalveolar Class II or III | Congenital dentofacial syndromes  Clinically evident facial asymmetry or functional mandibular deviations  Symptoms of temporomandibular disorders  Rheumatoid or other types of arthritis  Previous orthodontic treatment |
| Gedrange et al. 2005 | Retrospective  Institute and Polyclinic for Orthodontics and for Radiological Diagnostics – Carl Gustav Carus University Hospital of Dresden  Germany | CT  (Somaton HiQ – Siemens, Erlangen, Germany) with voltage 133 kV and 175 mA, slice thickness of 5 mm  Software: NR  Position: NR  Instructions: NR  Muscle: NR | M: 30  Males: 14  Females: 16  Age: 22±7.2  Cut-off-points: NR | M: 30  Males: 14  Females: 16  Age: 22±7.2  Cut-off-points: NR | Class II division 2 patients  Class III with anterior crossbite patients  Anterior openbite patients | NR |
| Gregor et al. 2013 | Prospective  Technical University of Dresden, Dresden  Germany | MRI  (1.5T Magnetom Sonata whole-body MRI/MRS system, Siemens AG)  Leonardo Workstation software (Siemens AG, Erlangen, Germany)  Position: NR  Instructions: NR  Muscle: NR | No grouping | M: 22  Males: 22  Females: 0  Age: 22 to 30 (24.6)  Cut-off-points:  AV: ML/MP 15-30°  LA: ML/MP <15°  HA: ML/MP >30° | Complete, healthy dentition  No functional disorders of the temporomandibular joint  Free of any orofacial pain  No previous orthodontic treatment  No bruxism | NR |
| Higashino et al. 2006 | Prospective  Tokyo University  Japan | MRI  Siemens, Magnetom Vision 1.5T  Volume-Rugle software (Medic Engineering)  Position: supine position  Instructions : central occlusion position (with a wax-bite recorded in advance)  Muscle: NR | N: 16  Males: 5  Females: 11  Age:  Males: mean 26.2  Females: mean 19.1  Cut-off-points: NR | N: 16  Males: 5  Females: 11  Age:  Males: mean 26.2  Females: mean 19.1  Cut-off-points: NR | NR | NR |
| Ispir et al. 2022 | Prospective  Gazi University Ankara  Turkey | Ultrasonography  (Fuji-film SonoSite M-Turbo (Fujifilm, WA, USA) with HFL38 x 13-6 MHz linear probe  Position: asked to lie on their back  Instructions: to stay in the most comfortable position by focusing on a point in front of them  Muscle: RS and MVC | No grouping | N: 115  Males: 51  Females: 64  Age: 15 to 55 (mean 22.2)  Cut-off-points:  AV: SN/GoGn 26-38°  LA: SN/GoGn <26°  HA: SN/GoGn >38°  AV: Po-Or/SGn 53-66°  LA: Po-Or/SGn <53°  HA: Po-Or/SGn >66°  SGo/NMe (Jarabak ratio) | No history of any congenital and/or acquired anomalies of the lips, mouth and face (cleft lip-palate, trauma, etc.)  No history of trauma in the head and neck region  Not having undergone a surgical operation on the head and neck region  No previous orthodontic treatment  Absence of posterior teeth missing other than third molars | NR |
| Kim et al. 2020 | Prospective  College of Dentistry, Dankook University  Korea | Multidetetor Computer Tomography (MDCT)  (Somatom Emotion 6 scanner, Siemens, Erlangen, Germany)  Software: NR  Position: supine position  Instructions: asked to place their mandible in the resting position with no force on the masticatory muscles  Muscle: NR | M: 100  Males: 50  Females: 50  Age: 18 to 30  Cut-off points:  Normal occlusion: NR  Mandibular prognathism: NR | M: 100  Males: 50  Females: 50  Age: 18 to 30  Cut-off points:  FH/MP : NR | Normal occlusion (N:50)  Mandibular prognathism (N:50) | Pre-existing syndrome  History of surgery due to facial fracture  History of orthognathic surgery |
| Kim et al. 2021 | Prospective  Wonkwang University College of Dentistry  Korea | CT  (Somatom Definition Dual Source CT, DSCT, Siemens, Forchhelm, Germany)  Software: Mimics 10.0 (Materialize n.v., Leuven, Belgium)  Position: NR  Instructions: NR  Muscle: NR | M: 60  Males: 30  Females: 30  Age: 18 to 29  Cut-off points:  Normal occlusion: NR  Mandibular prognathism: NR | No grouping | Age between 18 – 29  Complete dentition apart third molar  Patient’s agreement to 3DCT of the head | Severe asymmetry  Congenital Deformities  Chronic periodontitis  Trauma history relating to bilateral sides of the jawbone  Orthognathic procedures |
| Kubota et al. 1998 | Prospective  Iwate Medical University  Japan | Ultrasonography  (Aloka Co., Ltd. SSD-500 Japan) 7.5 MHz scanning probe (Aloka Co., Ltd. UST-5512U-7.5, linear rype, Japan)  Position: NR  Instructions: NR  Muscle: RS and MVC | No grouping | N: 80  Males: 80  Females: 0  Age: 23.8 (1.9)  Cut-off-points: NR | No history of orthodontic treatment  No missing teeth in the incisor region  No more than two missing teeth in the molar region (third molar excluded)  No asymmetry demonstrated on a postero-anterior roentgenographic cephalogram  No pain in temporomandibular joint | NR |
| Kusumah et al. 2009 | Prospective  Maxillofacial Orthognathic, Graduate School,  Tokyo Medical and Dental University  Japan | MRI  (1.5 T superconduction MRI system, Megnetom Vision, Siemens AG, Erlangen, Germany)  Position: supine position  Instructions: central occlusion position (with a wax-bite recorded in advance)  Muscle: NR | N: 16  Males: 5  Females: 11  Age: 13.5 to 27.5  Cut-off-points: NR | N: 16  Males: 5  Females: 11  Age: 13.5 to 27.5  Cut-off-points: NR | NR | NR |
| Li et al. 2008 | Prospective  Qingdao University Medical College  China | Ultrasonography  (5 MHz broadband transducer. Siemens Elegra, Erlangen, Germany)  Position: subject seated at an upright position, with the head in natural posture  Instructions: NR  Muscle: RS and MVC | No grouping | N: 60  Males: 0  Females: 60  Age: 18.96 (1.57)  Cut-off-points:  LA: FH/MP <22°  HA: FH/MP >32° | Complete dentition  Class I molar relation  Normal anteroposterior skeletal relationship  No temporomandibular joint disorders | NR |
| Lione et al. 2013  &  Novielo et al 2015 | Prospective  University of Rome, University of Florence and University of Michigan  Italy and USA | Ultrasonography  (Stream iU22, Philips Medical System, Amsterdam, The Netherlands) with 7 MHz VL13-5 Volume Linear Array, Philips xMATRIX Array Technology  Position: subjects seated upright with the FH parallel to the floor  Instructions: to maintain slight interocclusal contacts to avoid muscle stretching  Muscle: RS and MVC | No grouping | N: 60  Males: 33  Females: 27  Age: 11.5 (1.6)  Cut-off-points:  AV: FH/MP 22-28°  LA: FH/MP <22°  HA: FH/MP >28° | Prepuberal stage of development (CS1-CS3)  Late mixed or permanent dentition  Class I molar and skeletal relationship | Absence of first molars  Skeletal or functional asymmetries  Temporomandibular joint diseases  Genetic diseases  Previous orthodontic treatment |
| Mastroianni et al. 2019 | Retrospective  Oral and Maxillofacial Surgery Unit,  Royal Melbourne Hospital  Australia | CT  (Somatom Sensation 16, Siemens Medical Solutions AG, Erlangen, Germany)  Amira  (version 5.2.0 imaging software, Thermo Fisher Scientific, Waltham, Massachussetts, USA  Position: NR  Instructions: NR  Muscle: NR | No grouping | N: 58  Males: 32  Females: 26  Age: 13.27 (1.59)  Cut-off-points:  AV: FH/MP 22-29°  LA: FH/MP <22°  HA: FH/MP >29° | Both mandibular permanent canines erupted | NR |
| Naser-Ud-Din et al. 2010  &  Naser-Ud-Din et al. 2011 | Prospective  Orthodontic Unit, School of Dentistry, University of Adelaide  Australia | Ultrasonography  Position: comfortably seated with the left side of the face directed toward the examining sonographer  Instructions: NR  Muscle: NR | No grouping | N: 11  Males: 3  Females: 8  Age: 22 to 30  Cut-off-points: NR | Natural and healthy dentitions  Free of dental and neuromuscular symptoms  No discernable craniofacial dysmorphic traits | NR |
| Ngom et al. 2008 | Prospective  Department of orthodontics, Cheikh Anta Diop University, Dakar  Senegal | Ultrasonography  (Philips sono Diagnostic 260), linear probe with a width of 50 mm and 8 MHzz (L 7-4 Philips)  Position: NR  Instructions: NR  Muscle: RS and MVC | N: 102  Males: 56  Females: 46  Age: 25.75±4.09  Cut-off-points: NR | N: 102  Males: 56  Females: 46  Age: 25.75±4.09  Cut-off-points: NR | Reached complete adult dentition status with healthy, natural teeth | Missing teeth (third molar excluded)  Pain in the masticatory organs |
| Rani & Ravi 2010 | Prospective  A.B. Shetty Memorial Institute of Dental Science, Mangalore  India | Ultrasonography  (LOGIQ 400 PROSERIES scanner, GE Medical System, Milwaulkee, USA) using a multifrquency 7-.5-11.0 MHz broadband transducer  Position: NR  Instructions: teeth occluding gently with muscle in a relaxed position  Muscle: RS and MVC | N: 72  Males: 36  Females: 36  Age: 18 to 25 years  Cut-off points:  Class I: ANB 2-4°  Class II maxillary excess: ANB >4°  Class II mandibular deficiency: ANB >4° | No grouping | NR | NR |
| Rohila et al. 2012 | Prospective  University of Health Sciences, Rohtak, Haryana  India | Ultrasonography  (Toshiba Ultrasound Scanner and Probe (model no. Nemio SSA-550) with a 7.5-9.0 MHz broadband transducer  Position: supine position  Instructions: teeth occluding gently with the muscle in a relaxed position  Muscle: RS and MVC | No grouping | N: 60  Males: 30  Females: 30  Age : 18 to 24 years (22.3)  Cut-off-points:  AV: FHR 59-63%  LA: FHR >63%  HA: FHR <59% |  | Marked jaw asymmetries  Craniofacial disorders  History of trauma to dentofacial region  Parafunctional habits  Temporomandibular joint (TMJ) dysfunction |
| Satiroglu et al. 2005 | Prospective  Yeditepe University, Istanbul  Turkey | Ultrasonography  (Siemens Elegra, Erlangen, Germany) with a 7.5 to 9.0 MHz broadband transducer  Instructions: NR  Position: NR  Muscle: RS and MVC | No grouping | N: 47  Males: 24  Females: 23  Age : 24.96 (3.57)  Cut-off-points:  AV: NR  LA: NR  HA: NR | Young adults.  Permanent dentition  Skeletal jaw discrepancies (antero-posterior and vertical) | History of orthognathic treatment  Marked jaw asymmetries  Craniofacial disorders  Congenital or developmental anomalies of the lips, mouth or face |
| Soyoye et al. 2018 | Prospective  Obafemi Awolowo University  Nigeria | Ultrasonography  (time MINDRAY DC-7, 7.5 MHz linear probe)  Instructions: to maintain slight interocclusal contact in order to avoid muscle stretching  Position: supine position  Muscle: RS and MVC | No grouping | N: 66  Males: 21  Females: 45  Age : 12 to 30 years (19.15)  Cut-off-points:  AV: FH/MP 24-30°  LA: FH/MP <24°  HA: FH/MP >30° | Any history of orthodontic treatment or orthognathic surgery | Marked jaw asymmetry or craniofacial disorder and congenital developmental anomalies |
| Tekucheva et al. 2021 | Prospective  Samara State Medical University  Russia | Ultrasonography  (Logics-can 128, linear multi-frequency sensor HL10.0/25/96Z, with an operating frequency of 5-12 MHz  Instructions: rest & clench  Position: upright, natural head position  Muscle: RS and MVC | No grouping | N: 30  Males: NR  Females: NR  Age : 18 to 25 years (21.0)  Cut-off-points:  AV: FHR 65-75%  LA: FHR >75%  HA: FHR <65% | No molar and canine asymmetries  Less than 4 mm crowding  No symptoms of TMJ disorders  No pain in the masticatory muscles | Visible facial asymmetries  History of orthodontic treatment  Presence of congenital disorders  Trauma to the maxillofacial area  Presence of a neuromuscular disease  Pain in the masticatory muscles or TMJ  Bruxism |
| Uchide et al. 2011 | Prospective  Nihon University School of Dentistry, Tokyo  Japan | Ultrasonography  (SSA-250A; Toshiba, Tokyo, Japan), piezoelectric material, polymer film P(VDF-TrFE) with 7.5 MHz centre frequency  Instructions: muscle relaxed in the intercuspidal position  Position: comfortably seated  Muscle: RS and MVC | N: 24  Males: 11  Females: 13  Age : 27.6±5.6  Cut-off-points: NR | N: 24  Males: 11  Females: 13  Age : 27.6±5.6  Cut-off-points: NR | NR | History of orthodontic treatment  Missing teeth (third molar excluded)  Prosthesis  Diseases involving the neuromuscular mechanisms  Temporomandibular disorders including limitation of jaw opening on interview |
| Van Sprosen et al. 1992 | Prospective  Academic Centre for Dentistry Amsterdam  The Netherlands | MRI  (0.6 T system, Technicare Teslacon)  Software: NR  Instructions: to hold their teeth together but to avoid clenching  Position: supine position  Muscle: RS | No grouping | N: 48  Males: 48  Females: 0  Age: 31.2 (6.3)  Cut-off-points:  AV: ALFH/ATHF <59%  HA: ALFH/ATHF>59% | Complete or nearly complete dentition  No functional disorders of the temporomandibular joint | NR |
| Weijs et al. 1986 | Prospective  University of Groningen  The Netherlands | CT  Software: NR  Instructions: NR  Position: NR  Muscle: NR | N: 29  Males: 50  Females: 0  Age: 33.1±6.1  Cut-off-points: NR | N: 29  Males: 50  Females: 0  Age: 33.1±6.1  Cut-off-points: NR | Age between 22 and 46  Good general and dental health | More than three missing teeth apart from third molars  Gross orthodontic problems |
| Wong et al. 2016 | Retrospective  University of Melbourne, Royal Melbourne Hospital  Australia | CT  (SOMATOM Sensation 16, Siemens Medical Solutions AG, Erlangen, Germany)  Software: OsiriX® version 3.0, amira® 4.1.2 imagin software (Mercury Visualization Sciences), Dolphin 3D® (Dolhpin imagin and management solutions)  Instructions: NR  Position: head posture symmetrical bilaterally relative to the sagittal and coronal planes  Muscle: NR | No grouping | N: 30  Males: 17  Females: 13  Age: 8y7.5m to 15y1m  Cut-off-points:  AV: FH/MP 22-29°  LA: FH/MP <22°  HA: FH/MP >29° | NR | NR |
| Zepa et al. 2009 | Prospective  Riga Stradins University  Latvia | MRI  (GE Sign Advantage 1.0 MR System)  Software: GE Medical Systems Light Speed Pro 16CT99_Oc0 system  Instructions: NR  Position: supine position  Muscle: NR | N: 23  Males: NR  Females: NR  Age:  Mean 18.4 for Class II patients  Mean 19.2 for Class III patients  Cut-off points:  Class I: ANB 1-3°  Class II: ANB ≥4°  Class III: ≤0° | No grouping | Class II patients:  OJ ≥ 6mm  ANB ≥ 4°  Wits appraisal ≥ 4 mm  Class III patients:  OJ ≤ 0 mm  ANB ≤ 0°  Wits appraisal ≤ -4mm | Clinically evident facial asymmetry  Functional mandibular deviations  Symptoms of temporomandibular disorders  Previous orthodontic treatment |

NR: not reported; N: number of participants, SD: standard deviation, SE: standard error, AV: Average: HA: high angle, LA: low angle, AP: anteroposterior, RS: relaxation state ; MVC: maximum voluntary contraction ; CSA: cross-sectional area, FHR: facial height ratio, ALFH: anterior lower facial height, ATHF: anterior total facial height

Supplementary Table 7. Outcomes and results of the included studies according to the vertical skeletal pattern (Key Results).

| **Study** | **Outcomes**  **Hypodivergent**  **Mean (SD)** | **Outcomes**  **Normodivergent**  **Mean (SD)** | **Outcomes**  **Hyperdivergent**  **Mean (SD)** | **Difference**  **(significance *P*)** |
| --- | --- | --- | --- | --- |
| Ariji et al. 2000 | No grouping | No grouping | No grouping |  |
| Azaroual et al. 2014 | Masseter thickness: significant negative correlation with Ar-Go-Me (r: -0.34)  Masseter length: significant negative correlation with Ar-Go-Me (r: -0.35)  Masseter width: non-significant correlation with Ar-Go-Me (r: NR)  Masseter angle: non-significant correlation with Ar-Go-Me (r: NR)  LPM thickness: non-significant correlation with Ar-Go-Me (r: NR)  LPM pterygoid length: non-significant correlation with Ar-Go-Me (r: NR)  LPM pterygoid width: non-significant correlation with Ar-Go-Me (r: NR) | | | P=0.04  P=0.041  P˃0.05  P˃0.05  P˃0.05  P˃0.05  P˃0.05 |
| Becht et al. 2014 | Masseter angle (R): 73.37 (7.69)  Masseter angle (L): 73.15 (7.92)  Masseter length (R): 43.29 (3.95)  Masseter length (L): 43.44 (4.06) | Masseter angle (R): 74.31 (7.20)  Masseter angle (L): 74.19 (7.21)  Masseter length (R): 45.67 (4.45)  Masseter length (L): 45.61 (4.64) | Masseter angle (R): 70.29 (9.92)  Masseter angle (L): 70.23 (10.06)  Masseter length (R): 42.30 (4.76)  Masseter length (L): 42.36 (4.67) | R: 0.06  L: 0.07  R: 0.0002 Hypo / Norm  L: 0.0008 Hypo / Hyper |
| Benington et al. 1999 | Masseter volume: significant positive correlation with LPFH/TPFH (r: 0.77)  Masseter thickness: significant negative correlation with LAFH/TAFH (r: -0.63) | | | P≤0.05  P≤0.01 |
| Biondi et al. 2016 | **RS**  Masseter thickness (m): 8.52 (0.78)  Masseter thickness (f): 7.80 (1.19)  **MVC**  Masseter thickness (m): 10.50 (0.69)  Masseter thickness (f): 12.60 (0.80)  Masseter volume (m): 28.50 (0.90)  Masseter volume (f): 27.30 (1.09) | **RS**  Masseter thickness (m): 7.58 (0.78)  Masseter thickness (f): 6.80 (0.77)  **MVC**  Masseter thickness (m): 9.75 (1.13)  Masseter thickness (f): 10.71 (1.21)  Masseter volume (m): 26.20 (1.89)  Masseter volume (f): 24.35 (1.74) | **RS**  Masseter thickness (m): 6.70 (0.36)  Masseter thickness (f): 6.00 (0.52)  **MVC**  Masseter thickness (m): 8.96 (0.74)  Masseter thickness (f): 9.05 (0.90)  Masseter volume (m): 23.70 (2.01)  Masseter volume (f): 22.95 (2.30) | P<0.05  P<0.05  P<0.05  P<0.05  P<0.05  P<0.05 |
| Boom et al. 2008 | Masseter CSA (R): 5.1  Masseter CSA (L): 5.0  Masseter volume (R): 24.1  Masseter volume (L): 24.4  MPM CSA (R): 3.4  MPM CSA (L): 3.1  MPM volume (R): 9.9  MPM volume (L): 10.1 | NR | Masseter CSA (R): 4.8  Masseter CSA (L): 4.3  Masseter volume (R): 21.3  Masseter volume (L): 19.7  MPM CSA (R): 2.3  MPM CSA (L): 2.5  MPM volume (R): 7.1  MPM volume (L): 8.5 | NR  NR  NR  NR  NR  NR  NR  NR |
| Capaccioli et al. 1998 | Masseter thickness: non-significant negative correlation with SN/MP (r: -0.24)  Masseter length: non-significant negative correlation with SN/MP (r: -0.19)  Masseter width: non-significant negative correlation with SN/MP (r: -0.15) | | | P˃0.05  P˃0.05  P˃0.05 |
| Chan et al. 2008 | Masseter CSA (R): 3.39 (0.20)  Masseter CSA (L): 3.33 (0.26)  Masseter volume (R): 16.18 (2.18)  MPM CSA (R): 2.38 (0.30)  MPM CSA (L): 2.43 (0.37)  MPM volume (R): 6.73 (1.63)  LPM CSA (R): 2.40 (0.20)  LPM CSA (L): 2.31 (0.14)  LPM volume (R): 6.57 (0.53) | Masseter CSA (R): 3.39 (0.45)  Masseter CSA (L):3.45 (0.47)  Masseter volume (R): 17.12 (2.30)  MPM CSA (R): 2.48 (0.46)  MPM CSA (L): 2.60 (0.54)  MPM volume (R): 7.23 (1.10)  LPM CSA (R): 2.50 (0.39)  LPM CSA (L): 2.66 (0.45)  LPM volume (R): 7.03 (1.18) | Masseter CSA (R): 3.06 (0.59)  Masseter CSA (L): 3.00 (0.54)  Masseter volume (R): 13.21 (3.06)  MPM CSA (R): 2.21 (0.42)  MPM CSA (L): 2.10 (0.22)  MPM volume (R): 5.89 (1.13)  LPM CSA (R): 2.00 (0.27)  LPM CSA (L): 2.00 (0.32)  LPM volume (R): 5.73 (0.83) | P>0.05  P>0.05  P≤0.05  P>0.05  P>0.05  P>0.05  P≤0.05  P≤0.05  P>0.05 |
| Charalampidou et al. 2008 | **MVC**  Masseter thickness (m): NR  Masseter thickness (f): significant negative association with MP-PP (b: -1.075) in females | | | NR  P=0.045 |
| Farella et al. 2003 | **RS**  Masseter CSA: 14.3 (1.2) | **RS**  Masseter CSA: 3.1 (0.7) | | P=0.03 |
| Gardovska et al. 2020 | Masseter CSA: 5.182 (0.780)  Masseter volume: 25.0 (6.16)  MPM CSA: 3.082 (0.671)  MPM volume:11.3 (3.5) | Masseter CSA: 4.702 (1.0474)  Masseter volume: 22.1 (5.46)  MPM CSA: 2.904 (0.597)  MPM volume: 10.1 (3.04) | Masseter CSA: 4.287 (0.7761)  Masseter volume: 19.5 (4.58)  MPM CSA: 2.815 (0.81)  MPM volume: 10.3 (4.7) | P=0.008  P=0.006  P>0.05  P>0.05 |
| Gedrange et al. 2005 | Masseter CSA: 5.4 (0.7) | No grouping | Masseter CSA: 3.8 (0.4) | P<0.05 |
| Gregor et al. 2013 | Masseter volume: 32.0 (4.75) | NR | 21.82 (4.5) | P=0.00002 |
| Higashino et al. 2006 | Masseter inclination:   - significant negative correlation with FH-MP (r: NR) - significant negative correlation with ANB (r: NR) - significant negative correlation with Y axis (r: NR)   Masseter volume:   - significant negative correlation with FH-MP (r: NR) - significant negative correlation with gonial angle (r: NR) - significant negative correlation with Y axis (r: NR) | | | P=0.006  P=0.041  P=0.010  P=0.020  P=0.036  P=0.003 |
| Ispir et al. 2022 | **RS**  Right side  Masseter thickness (m): 14.23 (2.54)  Masseter thickness (f): 13.17 (2.19)  Masseter thick. (total): 13.81 (2.42)  Left side  Masseter thickness (m): 13.80 (2.84)  Masseter thickness (f): 12.91 (2.24)  Masseter thick. (total): 13.45 (2.62)  **MVC**  Right side  Masseter thickness (m): 15.88 (2.48)  Masseter thickness (f): 14.53 (2.05)  Masseter thick. (total) : 15.35 (2.37)  Left side  Masseter thickness (m): 15.41 (3.08)  Masseter thickness (f): 14.52 (1.99)  Masseter thick. (total): 15.06 (2.70) | **RS**  Right side  Masseter thickness (m): 13.34 (2.17)  Masseter thickness (f): 12.21 (2.77)  Masseter thick. (total): 12.75 (2.55)  Left side  Masseter thickness (m): 13.63 (2.21)  Masseter thickness (f): 11.82 (2.75)  Masseter thick. (total): 12.75 (2.55)  **MVC**  Right side  Masseter thickness (m): 14.98 (2.29)  Masseter thickness (f): 13.75 (2.78)  Masseter thick. (total): 14.33 (2.61)  Left side  Masseter thickness (m): 15.36 (2.49)  Masseter thickness (f): 13.47 (2.72)  Masseter thick. (total): 14.37 (2.76) | **RS**  Right side  Masseter thickness (m): 11.85 (2.53)  Masseter thickness (f): 12.04 (2.93)  Masseter thick. (total): 11.99 (3.02)  Left side  Masseter thickness (m): 12.13 (3.08)  Masseter thickness (f): 12.06 (2.59)  Masseter thick. (total): 12.08 (2.67)  **MVC**  Right side  Masseter thickness (m): 13.59 (3.63)  Masseter thickness (f): 13.65 (2.72)  Masseter thick. (total) : 13.64 (2.91)  Left side  Masseter thickness (m): 14.52 (2.46)  Masseter thickness (f): 13.76 (2.22)  Masseter thick. (total): 13.95 (2.27) | **RS**  Right side (total): P=0.034  Left side (total): P=0.139  **MVC**  Right side (total): P=0.047  Left side (total): P=0.261 |
| Kim et al. 2020 | Masseter thickness: non-significant negative correlation with FH/MP (r: -0.152)  MPM thickness: non-significant negative correlation with FH/MP (r: 0.029)  LPM thickness: non-significant negative correlation with FH/MP (r: -0.156)  Temporalis thickness: non-significant negative correlation with FH/MP (r: -0.041) | | | P>0.05  P>0.05  P>0.05  P>0.05 |
| Kim et al. 2021 | No grouping | No grouping | No grouping |  |
| Kubota et al. 1998 | **RS**  Masseter thickness: significant negative correlation with FH/MP (r: -0.41)  **MVC**  Masseter thickness: significant negative correlation with FH/MP (r: -0.37) | | | **RS**  P<0.01  **MVC**  P<0.01 |
| Kubota et al. 1998 | No grouping | No grouping | No grouping |  |
| Kusumah et al. 2009 | MPM volume: significant negative correlation with FH/MP (r: -0.282) | | | P=0.145 |
| Li et al. 2008 | **RS**  Masseter thickness (f): 14.14 (1.70)  **MVC**  Masseter thickness (f): 16.40 (1.22) |  | **RS**  Masseter thickness (f): 12.36 (1.78)  **MVC**  Masseter thickness (f): 14.09 (1.79) | P=0.009  P=0.000 |
| Lione et al. 2013  &  Noviello et al 2015 | **RS**  Masseter CSA: 3.1 (0.4)  Masseter thickness: 0.9 (0.1)  Masseter volume: 14.9 (0.9)  Masseter width: 4.6 (0.4)  **MVC**  Masseter CSA: 3.6 (0.4)  Masseter thickness: 1.2 (0.1)  Masseter volume: 16.7 (1.1)  Masseter width: 4.2 (0.3) | **RS**  Masseter CSA: 2.6 (0.5)  Masseter thickness: 0.8 (0.1)  Masseter volume: 13.5 (1.2)  Masseter width: 4.5 (0.4)  **MVC**  Masseter CSA: 3.3 (0.6)  Masseter thickness: 1.0 (0.1)  Masseter volume: 14.9 (1.4)  Masseter width: 4.0 (0.4) | **RS**  Masseter CSA: 2.1 (0.4)  Masseter thickness: 0.6 (0.1)  Masseter volume: 11.8 (1.0)  Masseter width: 4.0 (0.4)  **MVC**  Masseter CSA: 2.4 (0.4)  Masseter thickness: 0.8 (0.1)  Masseter volume:12.7 (1.1)  Masseter width: 3.7 (0.3) | P<0.05  P<0.05 (Norm vs Hyper ; Hypo vs Hyper)  P<0.05  P<0.05 (Norm vs Hyper ; Hypo vs Hyper)  P<0.05  P<0.05  P<0.05  P<0.05 (Norm vs Hyper ; Hypo vs Hyper) |
| Mastroianni et al. 2019 | Masseter CSA: 4.71 (0.33)  Masseter volume: 19.07 (1.49) | Masseter CSA: 3.48 (0.51)  Masseter volume: 16.62 (1.99) | Masseter CSA: 3.10 (0.44)  Masseter volume:13.94 (2.61) | P≤0.05  P≤0.05 |
| Naser-Ud-Din et al. 2010 & 2011 | Significant correlations were reported between the following parameters.   - Masseter length – mandibular length (Ar-Gn) (r: 0.64) - Masseter thickness – mandibular body length (Go-Me) (r: 0.62) - Masseter thickness – anterior facial height (N-Me) (r: 0.62) - Masseter thickness – ramus shape (R1-R2) (r: 0.83) - Masseter thickness – ramus shape (R3-R4) (r: 0.61) - Masseter volume – superficial masseter muscle length (co-Go) (r: 0.8) - Masseter volume – mandibular body length (Go-Me) (r: 641) - Masseter volume – ramus shape (R1-R2) (r: 0.79) - Masseter volume – ramus shape (R3-R4) (r: 0.69) - Masseter area – superficial masseter muscle length (Co-Go) (r: 0.81) - Masseter area – mandibular length (Ar-Gn) (r: 0.85) - Masseter area – facial proportion (ArGo-GoMe) (r: 0.84) - Masseter area – facial proportion (NGn/ArGo) (r: -0.85) | | | P=0.03  P=0.04  P=0.04  P=0.002  P=0.05  P=0.003  P=0.03  P=0.004  P=0.02  P=0.002  P=0.001  P=0.001  P=0.001 |
| Ngom et al. 2008 | **RS**  Masseter thickness: significant negative correlation with SN/MP (r: -0.28)  **MVC**  Masseter thickness: significant negative correlation with SN/MP (r: -0.30) | | | P=0.005  P=0.002 |
| Rani & Ravi 2010 | No grouping | No grouping | No grouping |  |
| Rohila et al. 2012 | **RS**  Masseter thickness (m): 14.75 (1.30)  Masseter thickness (f): 13.14 (1.29)  **MVC**  Masseter thickness (m): 16.07 (1.20)  Masseter thickness (f): 14.85 (1.22) | **RS**  Masseter thickness (m): 13.14 (1.05)  Masseter thickness (f): 11.92 (1.08)  **MVC**  Masseter thickness (m): 14.47 (1.23)  Masseter thickness (f): 13.16 (1.23) | **RS**  Masseter thickness (m): 11.40 (1.13)  Masseter thickness (f): 10.87 (1.23)  **MVC**  Masseter thickness (m): 12.53 (1.25)  Masseter thickness (f): 12.01 (1.28) | NR  NR  NR  NR |
| Satiroglu et al. 2005 | **RS**  Masseter thickness (m): 15.87 (1.83)  Masseter thickness (f): 13.55 (0.74)  **MVC**  Masseter thickness (m): 17.01 (2.15)  Masseter thickness (f): 14.57 (0.98) | **RS**  Masseter thickness (m): 14.92 (1.59)  Masseter thickness (f): 12.74 (1.69)  **MVC**  Masseter thickness (m): 15.92 (1.89)  Masseter thickness (f): 13.76 (1.24) | **RS**  Masseter thickness (m): 15.50 (1.99)  Masseter thickness (f): 12.08 (1.89)  **MVC**  Masseter thickness (m): 17.19 (2.05)  Masseter thickness (f): 13.37 (1.82) | P>0.05  P>0.05  P>0.05  P>0.05 |
| Soyoye et al. 2018 | **RS**  Masseter thickness (m): 14.13 (1.16)  Masseter thickness (f): 10.51 (2.36)  **MVC**  Masseter thickness (m): 16.71 (2.65)  Masseter thickness (f): 12.80 (2.20) | **RS**  Masseter thickness (m): 11.08 (1.76)  Masseter thickness (f): 12.24 (2.60)  **MVC**  Masseter thickness (m): 13.17 (1.92)  Masseter thickness (f): 13.48 (2.20) | **RS**  Masseter thickness (m): 10.00 (1.58)  Masseter thickness (f): 10.63 (2.32)  **MVC**  Masseter thickness (m): 11.32 (1.44)  Masseter thickness (f): 12.77 (2.64) | P=0.001  P>0.05  P<0.001  P>0.05 |
| Tekucheva et al. 2021 | **RS**  Masseter CSA (R): 2.85 (0.09)  Masseter CSA (L): 3.14 (0.10)  Masseter thickness (R): 10.39 (0.9)  Masseter thickness (L): 12.37 (0.8)  **MVC**  Masseter CSA (R): 4.03 (0.14)  Masseter CSA (L): 4.18 (0.07)  Masseter thickness (R): 15.97 (1.1)  Masseter thickness (L): 15.27 (0.9) | **RS**  Masseter CSA (R): 2.59 (0.08)  Masseter CSA (L): 2.58 (0.09)  Masseter thickness (R): 11.22 (0.8)  Masseter thickness (L): 10.24 (0.4)  **MVC**  Masseter CSA (R): 3.36 (0.07)  Masseter CSA (L): 3.27 (0.11)  Masseter thickness (R): 13.30 (0.5)  Masseter thickness (L): 12.95 (0.9) | **RS**  Masseter CSA (R): 2.51 (0.08)  Masseter CSA (L): 2.16 (0.07)  Masseter thickness (R): 9.94 (0.7)  Masseter thickness (L): 8.51 (0.6)  **MVC**  Masseter CSA (R): 2.08 (0.10)  Masseter CSA (L): 3.05 (0.09)  Masseter thickness (R): 12.17 (0.8)  Masseter thickness (L): 11.93 (0.8) | **RS**  Comparison between groups (R=L):  P=0.001 (Hypo vs Norm)  P<0.001 (Hypo vs Hyper)  P=0.02 (Hyper vs Norm)  **MVC**  Comparison between groups (R):  P=0.001 (Hypo vs Norm)  P<0.001 (Hypo vs Hyper)  P=0.10 (Hyper vs Norm)  Comparison between groups (L):  P=0.002 (Hypo vs Norm)  P<0.001 (Hypo vs Hyper)  P=0.02 (Hyper vs Norm) |
| Uchide et al. 2011 | **RS**  Masseter CSA: significant negative correlation with SN/MP (r: -0.50)  **MVC**  Masseter CSA: significant negative correlation with SN/MP (r: -0.53) | | | P<0.05  P<0.01 |
| Van Spronsen et al. 1992 |  | **RS**  Temporalis CSA (R): 5.31 (0.75)  Temporalis CSA (L): 4.99 (0.89)  AT CSA (R): 2.91 (0.58)  AT CSA (L): 2.58 (0.51)  Masseter CSA (R): 4.86 (0.83)  Masseter CSA (L): 4.64 (1.03)  MPM CSA (R): 3.19 (0.54)  MPM CSA (L): 3.21 (0.52)  LPM CSA (R): 4.18 (0.61)  LPM CSA (L): 4.15 (0.58) | **RS**  Temporalis CSA (R): 4.54 (0.75)  Temporalis CSA (L): 4.32 (0.60)  AT CSA (R): 2.53 (0.50)  AT CSA (L): 2.25 (0.49)  Masseter CSA (R): 3.64 (0.43)  Masseter CSA (L): 3.41 (0.50)  MPM CSA (R): 2.43 (0.55)  MPM CSA (L):2.70 (0.44)  LPM CSA (R): 3.62 (0.42)  LPM CSA (L): 3.75 (0.58) | NR  NR  NR  NR  NR  NR  NR  NR  NR  NR |
| Weijs et al. 1986 | Masseter CSA: non-significant negative correlation with PP/MP (r: -0.21)  MPM CSA: non-significant negative correlation with PP/MP (r: -0.22)  LPM CSA: non-significant positive correlation with PP/MP (r: 0.17)  Temporalis CSA: non-significant negative correlation with PP/MP (r: -0.01) | | | P>0.05  P>0.05  P>0.05  P>0.05 |
| Wong et al. 2016 | Masseter CSA (R): 3.69 (0.29)  Masseter CSA (L): 3.66 (0.24)  MPM CSA (R): 2.79 (0.42)  MPM CSA (L): 2.77 (0.43)  Masseter volume (R): 18.08 (1.55)  MPM volume (R): 6.80 (1.52) | Masseter CSA (R): 3.45 (0.43)  Masseter CSA (L): 3.41 (0.42)  MPM CSA (R): 2.60 (0.48)  MPM CSA (L): 2.63 (0.48)  Masseter volume (R): 16.97 (2.30)  MPM volume (R): 7.21 (1.11) | Masseter CSA (R): 3.07 (0.42)  Masseter CSA (L): 3.08 (0.49)  MPM CSA (R): 2.20 (0.36)  MPM CSA (L): 2.21 (0.43)  Masseter volume (R): 15.12 (2.83)  MPM volume (R): 5.77 (0.92) | P>0.05  P>0.05  P>0.05  P>0.05  P≤0.05 (masseter volume hyperdivergent)  P>0.05 |
| Zepa et al. 2009 | No grouping | No grouping | No grouping |  |

RS: relaxation state ; MVC: maximum voluntary contraction; R: right, L: left, m: male, f: female, MPM: medial pterygoid muscle, LPM: lateral pterygoid muscle, AT: anterior temporalis, T: temporalis

**Supplementary Table 8. Outcomes and results of the included studies according to the sagittal skeletal pattern (Key results).**

| **Study** | **Outcomes**  **Class I**  **Mean (SD)** | **Outcomes**  **Class II**  **Mean (SD)** | **Outcomes**  **Class III** | **Differences between groups**  **(significance *P*)** |
| --- | --- | --- | --- | --- |
| Ariji et al. 2000 | Masseter angle: 65.1 (4.4)    Masseter CSA: 368.3 (97.2) | No grouping | Masseter angle: 76.6 (4.4)    Masseter CSA: 318.3 (77.2) | P<0.001    P<0.001 |
| Azaroual et al. 2014 | No grouping | No grouping | No grouping |  |
| Becht et al. 2014 | Masseter angle (R): 71.70 (4.55)  Masseter angle (L): 71.53 (4.73)    Masseter length (R): 43.72 (4.21)  Masseter length (L): 43.67 (4.24) | Masseter angle (R): 67.41 (6.55)  Masseter angle (L): 67.09 (6.62)    Masseter length (R): 44.51 (4.92)  Masseter length (L): 44.63 (4.93) | Masseter angle (R): 81.64 (6.87)  Masseter angle (L): 81.67 (6.78)    Masseter length (R): 43.70 (4.38)  Masseter length (L): 43.87 (4.57) | R: P< 0.0001 Class I / Class II  L: P< 0.0001 Class I / Class II                        Class II / Class III  R: P=0.54  L: P=0.46 |
| Benington et al. 1999 | NR | NR | NR | NR |
| Biondi et al. 2016 | No grouping | No grouping | No grouping |  |
| Boom et al. 2008 | No grouping | No grouping | No grouping |  |
| Capaccioli et al. 1998 | No grouping | No grouping | No grouping |  |
| Chan et al. 2008 | No grouping | No grouping | No grouping |  |
| Charalampidou et al. 2008 | No grouping | No grouping | No grouping |  |
| Farella et al. 2003 | No grouping | No grouping | No grouping |  |
| Gardovska et al. 2020 | NR | NR | NR | NR |
| Gedrange et al. 2005 | No grouping | No grouping | NR | NR |
| Gregor et al. 2013 | No grouping | No grouping | No grouping |  |
| Higashino et al. 2006 | Masseter inclination: significant negative correlation with ANB (r: NR) | | | P=0.041 |
| Ispir et al. 2020 | No grouping | No grouping | No grouping |  |
| Kim et al. 2020 | Masseter thickness (m): 15.73 (1.98)  Masseter thickness (f): 12.85 (1.75)  MPM thickness (m): 14.73 (1.32)  MPM thickness (f): 13.58 (0.84)  LPM thickness (m): 15.59 (1.40)  LPM thickness (f): 13.51 (1.22) |  | Masseter thickness (m): 14.43 (1.75)  Masseter thickness (f): 12.60 (1.27)  MPM thickness (m): 14.52 (1.76)  MPM thickness (f): 13.31 (1.71)  LPM thickness (m): 15.15 (1.66)  LPM thickness (f): 14.31 (1.42) | P˂0.05  P˂0.05  P˃0.05  P˃0.05  P˂0.05  P˂0.05 |
| Kim et al. 2021 | LPM volume/length ratio: 268.5 (42.6) | No grouping | LPM volume/length ratio: 231.8 (58.5) | P<0.05 |
| Kubota et al. 1998 | No grouping | No grouping | No grouping |  |
| Kusumah et al. 2009 | MPM volume: significant negative correlation with ANB (r: -0.191) | | | P=0.239 |
| Li et al. 2008 | No grouping | No grouping | No grouping |  |
| Lione et al. 2013  &  Noviello et al. 2015 | No grouping | No grouping | No grouping |  |
| Mastroianni et al. 2019 | No grouping | No grouping | No grouping |  |
| Naser-Ud-Din et al. 2010  &  Naser-Ud-Din et al. 2011 | No grouping | No grouping | No grouping |  |
| Ngom et al. 2008 | **RS**  Masseter thickness: non-significant negative correlation with ANB (r: -0.11)    **MVC**  Masseter thickness: non-significant negative correlation with ANB (r: -0.08) | | | P=0.26      P=0.44 |
| Rani & Ravi 2010 | **RS**  Masseter thickness (m): 11.21 (0.98)  Masseter thickness (f): 9.65 (0.58)  **MVC**  Masseter thickness (m): 13.74 (1.11)  Masseter thickness (f): 11.93 (0.81) | **Mx excess – RS**  M.t (m): 11.15 (1.01)  M.t (f): 8.69 (0.82)  **Md deficiency – RS**  M.t (m): 9.39 (1.21)  M.t (f): 8.65 (1.43)  **Mx excess – MVC**  M.t (m): 13.70 (1.20)  M.t (f): 11.12 (1.21)  **Md deficiency – MVC**  M.t (m): 11.74 (1.60)  M.t (f): 11.08 (1.72) | No grouping | P=0.002  P=0.009 |
| Rohila et al. 2012 | No grouping | No grouping | No grouping |  |
| Satiroglu et al. 2005 | No grouping | No grouping | No grouping |  |
| Soyoye et al. 2018 | No grouping | No grouping | No grouping |  |
| Tekucheva et al. 2021 | No grouping | No grouping | No grouping |  |
| Uchide et al. 2011 | **RS**  Masseter CSA: non-significant negative correlation with ANB (r: -0.011)    **MVC**  Masseter CSA: non-significant negative correlation with ANB (r: -0.07) | | | P>0.05      P>0.05 |
| Van Spronsen et al. 1992 | No grouping | No grouping | No grouping |  |
| Weijs et al. 1986 | Masseter CSA: significant negative correlation with relative mandibular prognathism* (r: -0.40)  MPM CSA: significant negative correlation with relative mandibular prognathism (r: -0.44)  LPM CSA: significant negative correlation with relative mandibular prognathism (r: -0.51)  Temporalis CSA:  non-significant negative correlation with relative mandibular prognathism (r: -0.17)  *distance between the projections A’ and B’ of A and B on the occlusal line | | | P<0.05  P<0.02  P<0.01  P>0.05 |
| Wong et al. 2016 | No grouping | No grouping | No grouping |  |
| Zepa et al. 2009 |  | Masseter CSA (R): 4.46 (1.06)  Masseter CSA (L): 4.50 (0.91)    Masseter length (R): 71.50 (5.8)  Masseter length (L): 70.24 (6.8)    Masseter thickness (R): 14.50 (1.8)  Masseter thickness (L): 14.00 (1.7)    Masseter volume (R): 22.70 (4.5)  Masseter volume (L): 21.50 (4.7)    Masseter width (R): 41.40 (4.0)  Masseter width (L): 42.00 (4.3)    MPM CSA (R):3.06 (0.54)  MPM CSA (L): 3.12 (0.65)    MPM volume (R): 9.70 (3.8)  MPM volume (L): 9.70 (3.9)    MPM thickness (R): 16.90 (1.7)  MPM thickness (L): 17.80 (1.7) | Masseter CSA (R): 4.91 (1.03)  Masseter CSA (L): 4.96 (1.10)    Masseter length (R): 72.20 (4.7)  Masseter length (L): 73.10 (5.4)    Masseter thickness (R): 14.70 (2.7)  Masseter thickness (L):14.00 (2.10)    Masseter volume (R): 25.00 (5.4)  Masseter volume (L):24.20 (6.2)    Masseter width (R): 43.1 (3.7)  Masseter width (L): 43.20 (4.8)    MPM CSA (R): 3.45 (0.61)  MPM CSA (L): 3.48 (0.62)    MPM volume (R): 16.60 (5.3)  MPM volume (L): 16.7 (5.3)    MPM thickness (R): 19.90 (1.9)  MPM thickness (L): 19.90 (1.5) | P>0.05  P>0.05    P>0.05  P>0.05    P>0.05  P>0.05    P>0.05  P>0.05    P>0.05  P>0.05    P>0.05  P>0.05    P<0.05  P<0.05    P<0.05  P<0.05 |

CSA: Cross-sectional area, SD: standard deviation; R: right, L: left, MPM : medial pterygoid muscle, LPM: lateral pterygoid muscle.

**Supplementary Table 9. Risk of Bias Assessment tool.** **Domains of the Risk of bias (RoB) assessment tool.** 1. Were the aims/objectives of the study clear? 2. Were the criteria for inclusion in the sample clearly defined? 3. Were the study subjects and the setting described in detail (time, location, demographics)? 4. Was the sample size justified (sample size calculation)? 5. Were objective, standard criteria used for measurement of the condition (cut-off values for cephalometric parameters used to categorize the patients in the sagittal and vertical groups)? 6. Were confounding factors identified (age, sex, sagittal classification for studies that grouped per vertical and vice-versa, functional shifts, asymmetries, linear or curved probe used etc.)? 7. Were strategies to deal with confounding factors stated (analyses adjusted for confounders)? 8. Were the outcomes measured in a valid way (valid and accepted boundaries also used in the medical terminology)? 9. Were the outcomes measured in a reliable way (experience and/or training and/or calibration of the one-s measuring the muscles)? 10. method error calculation performed? 11. Were the assessors blinded to the groups (the ones measuring the muscles being aware of the cephalometric values)? 12. Was appropriate statistical analysis used? 13. Is it clear what was used to determine statistical significance and/or precision estimates (*p* values, Confidence Intervals-CIs etc.)? 14. Were all specified outcome data adequately described and reported (incomplete reporting)? Y: Yes; U: Unclear; N: No.

| **Studies** | **1** | **2** | **3** | **4** | **5** | **6** | **7** | **8** | **9** | **10** | **11** | **12** | **13** | **14** |
| --- | --- | --- | --- | --- | --- | --- | --- | --- | --- | --- | --- | --- | --- | --- |
| Ariji 2000 | y | u | u | n | u | n | n | y | y | y | u | y | u | y |
| Azaroual 2014 | y | u | u | n | n | n | n | y | u | n | u | y | y | n |
| Becht 2014 | y | u | u | n | y | n | n | y | u | y | u | y | y | y |
| Benigton 1999 | y | y | u | n | y | n | n | y | u | y | u | y | y | n |
| Biondi 2016 | y | y | u | n | y | n | n | y | u | n | u | y | y | y |
| Boom 2008 | y | u | u | n | y | n | n | y | u | y | u | y | y | n |
| Capaccioli 1998 | y | y | u | n | n | n | n | y | u | n | u | y | y | n |
| Chan 2008 | y | u | y | n | y | n | n | y | u | y | u | y | y | y |
| Charalampidou 2008 | y | y | u | n | u | n | n | y | u | y | u | y | y | n |
| Farella 2003 | y | y | y | n | y | n | n | y | u | y | u | y | y | y |
| Gardovska 2020 | y | y | u | n | y | n | n | y | u | y | u | y | y | y |
| Gedrange 2005 | y | u | u | n | u | n | n | y | u | y | u | y | y | n |
| Gregor 2013 | y | y | u | n | y | n | n | y | u | y | u | y | y | y |
| Hagashino 2006 | y | u | u | n | u | n | n | y | u | n | u | y | y | n |
| Ispir 2022 | y | y | u | n | y | n | n | y | u | y | u | y | y | y |
| Kim 2020 | y | y | u | n | u | n | n | y | u | n | u | y | y | n |
| Kim 2021 | y | y | y | n | n | n | n | u | u | y | u | y | y | n |
| Kubota 1998 | y | y | u | n | y | n | n | y | u | y | u | y | y | n |
| Kusumah 2009 | y | u | u | n | u | n | n | u | u | y | y | y | y | n |
| Li 2008 | y | u | u | n | y | n | n | y | u | n | u | y | y | y |
| Lione 2013 & Noviello 2015 | y | y | y | y | y | n | n | y | y | y | y | y | y | y |
| Mastroianni & Woods 2019 | y | u | y | n | y | n | n | y | u | y | u | y | y | y |
| Naser-Ud-Din 2010 & 2011 | y | u | u | n | u | n | n | y | u | y | u | y | y | n |
| Ngom 2008 | y | u | u | n | u | n | n | y | u | y | u | y | y | y |
| Rani & Ravi 2010 | y | u | u | n | y | n | n | y | u | n | u | y | y | y |
| Rohila 2012 | y | u | u | n | u | n | n | y | u | y | u | y | y | n |
| Satiroglu 2005 | y | y | u | n | u | n | n | y | y | y | u | y | y | y |
| Soyoye 2018 | y | y | u | n | y | n | n | y | y | y | u | y | y | y |
| Tekucheva 2021 | y | y | u | n | y | n | n | y | u | n | u | y | y | y |
| Uchida 2011 | y | y | u | n | u | n | n | y | u | y | u | y | y | n |
| vanSpronsen 1992 | y | u | u | n | y | n | n | y | u | n | u | y | n | y |
| Weijs & Hillen 1986 | y | y | u | n | u | n | n | y | u | n | u | y | y | n |
| Wong 2016 | y | y | u | n | y | n | n | y | u | y | u | y | y | y |
| Zepa 2009 | y | y | u | n | y | n | n | y | u | y | u | y | y | y |

**Supplementary Table 10.** Summary of findings table of **hypodivergent** versus **normodivergent** patients according to GRADE.


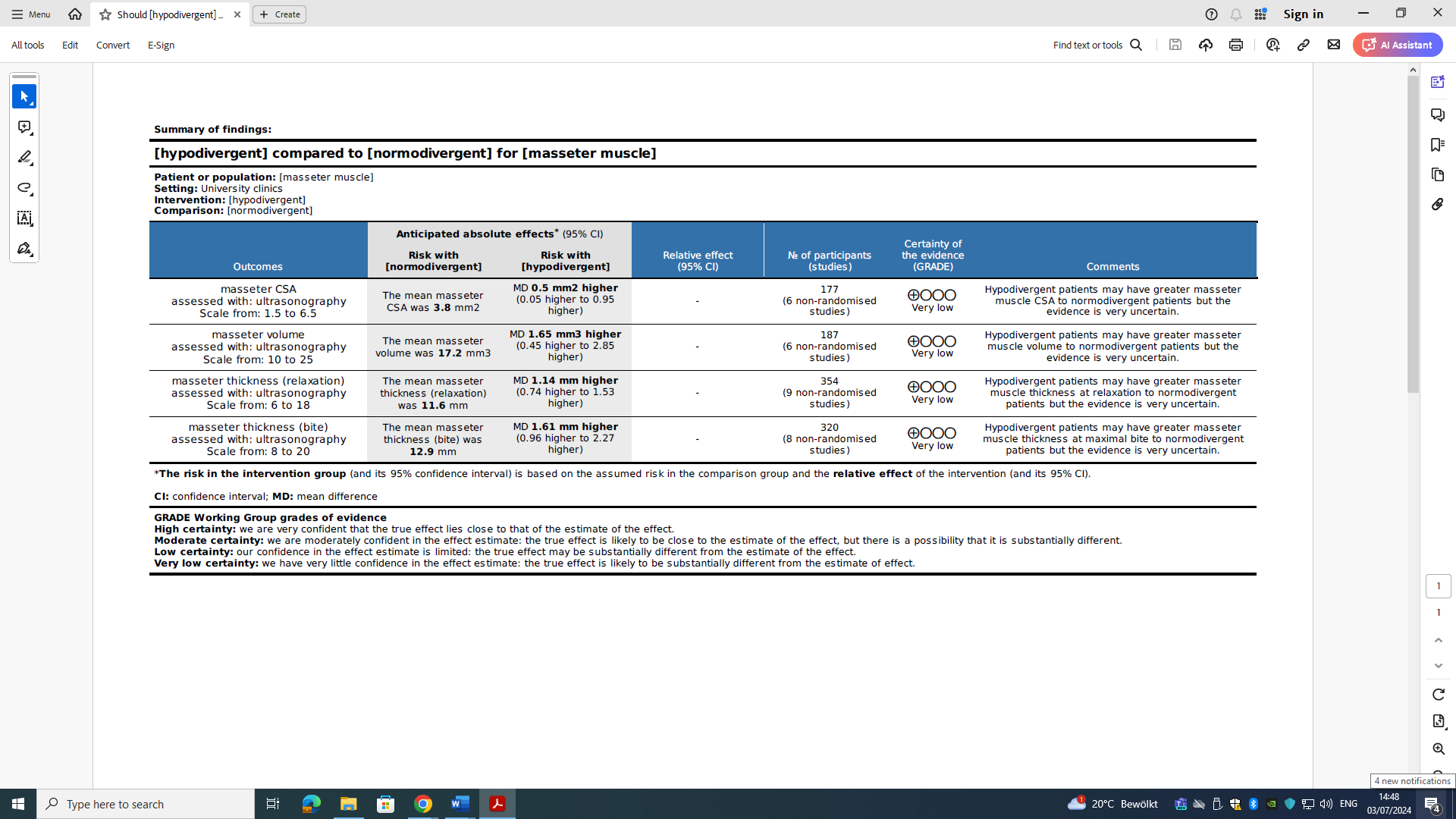


**Supplementary Table 11.** Summary of findings table of **hyperdivergent** versus **normodivergent** patients according to GRADE.


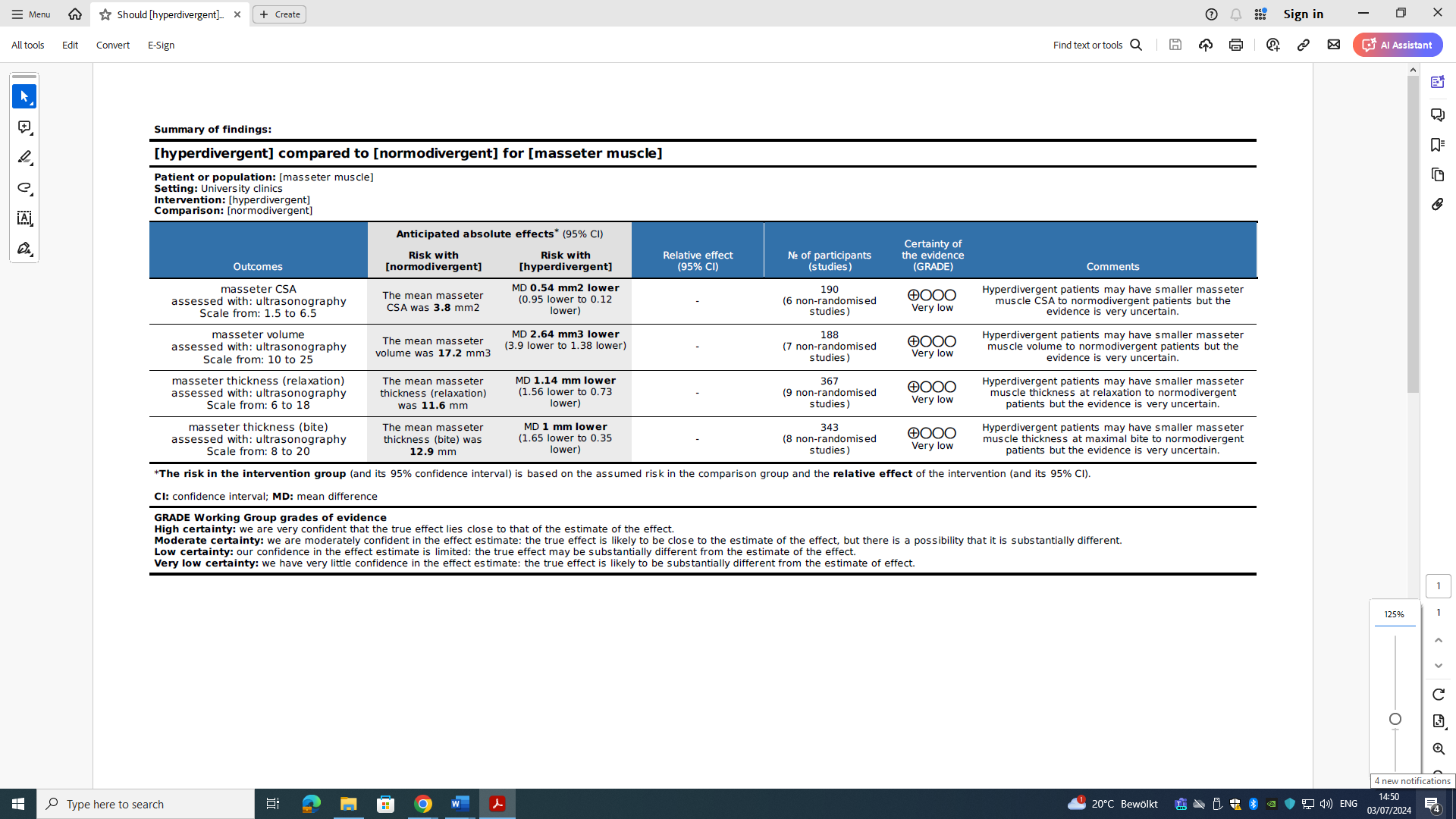


**Supplementary Table 12.** Summary of findings table of **hyperdivergent** versus **hypodivergent** patients according to GRADE.


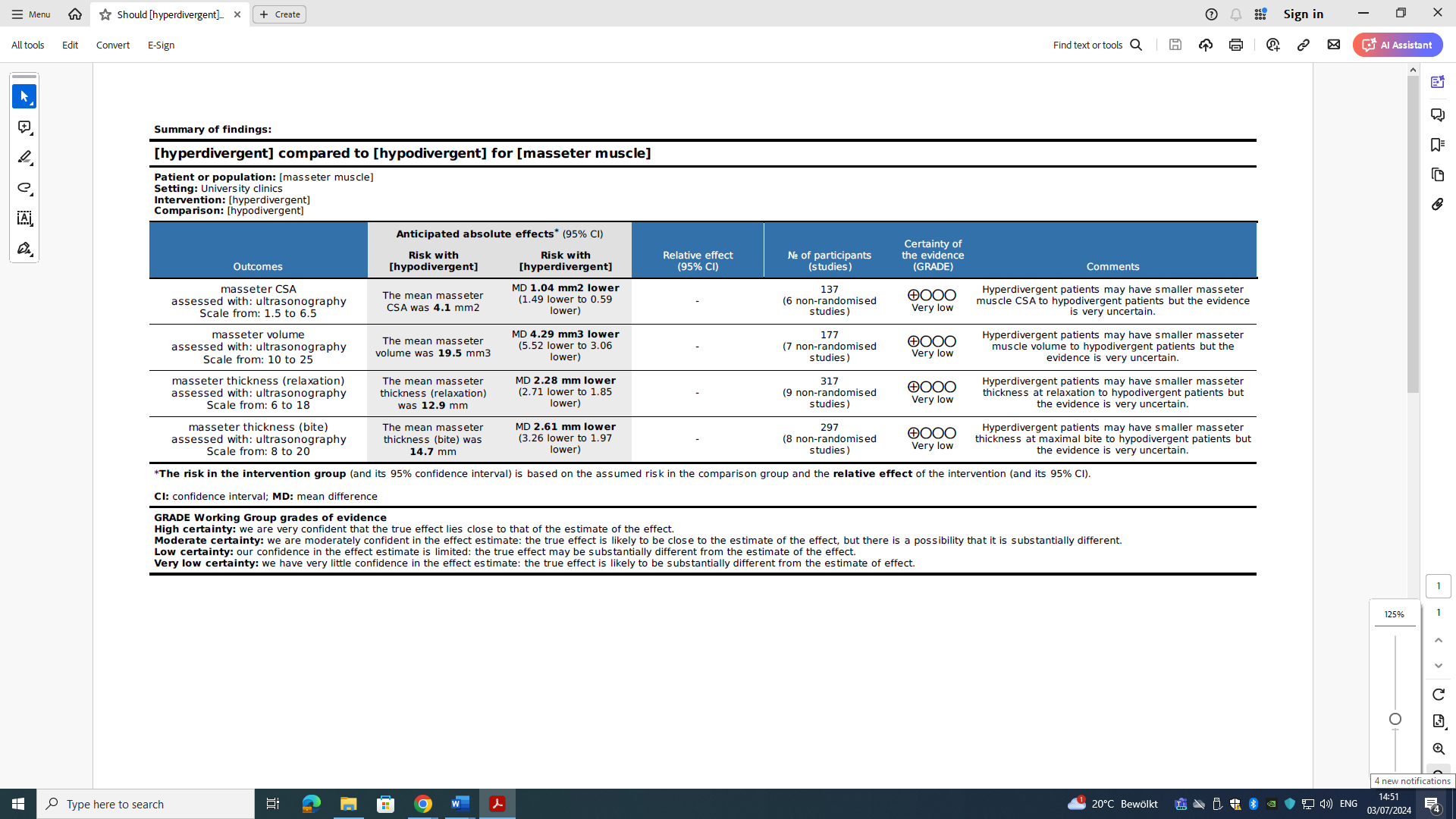

Supplement: Supplementary file 1 — Additional file1 (DOCX 1154 KB) [file 40510_2024_534_MOESM1_ESM.docx]
