# Supplementary material for: Relationship between craniofacial skeletal patterns and anatomic characteristics of masticatory muscles: a systematic review and meta-analysis
Source: Prog Orthod. 2024 Sep 9;25:36. doi: 10.1186/s40510-024-00534-2 (PMC11381490; doi:10.1186/s40510-024-00534-2)
Supplement: Supplementary file 2 — Additional file2 (DOCX 464 KB) [file 40510_2024_534_MOESM2_ESM.docx]

|  | **Study** | **Excluded by** |
| --- | --- | --- |
|  | AAPM 2012 Annual Meeting Abstracts. Pain Medicine (United States). 2012;13(2). | Title |
|  | Summer Meeting of the Anatomical Society. Journal of Anatomy. 2023;242(4). | Title |
|  | Aal-Blowi A, Aal-Mutairi R, Ghabbany RM, Manaa AM, Aloufi MM, Ternati GK, et al. The prevalence of malnutrition and the nutritional status in children with cerebral palsy and its causes in madinah maternity and children hospital. Current Pediatric Research. 2020;24(7):273-80. | Title |
|  | Abarca M, Van Steenberghe D, Malevez C, Jacobs R. The neurophysiology of osseointegrated oral implants. A clinically underestimated aspect. Journal of Oral Rehabilitation. 2006;33(3):161-9. | Title |
|  | Abdi AH, Sagl B, Srungarapu VP, Stavness I, Prisman E, Abolmaesumi P, Fels S. Characterizing Motor Control of Mastication With Soft Actor-Critic. Frontiers in Human Neuroscience. 2020;14. | Abstract |
|  | Abe N, Yashiro K, Hidaka O, Takada K. Influence of gum-chewing on the haemodynamics in female masseter muscle. Journal of Oral Rehabilitation. 2009;36(4):240-9. | Title |
|  | Abe S, Miyagi A, Yoshinaga K, Matsuka Y, Matsumoto F, Uyama E, et al. Immediate effect of masticatory muscle activity with transcutaneous electrical nerve stimulation in muscle pain of temporomandibular disorders patients. Journal of Clinical Medicine. 2020;9(10):1-13. | Title |
|  | Abed GS, Buschang PH, Taylor R, Hinton RJ. Maturational and functional related differences in rat craniofacial growth. Archives of Oral Biology. 2007;52(11):1018-25. | Title |
|  | Abel EW, Hilgers A, McLoughlin PM. Finite element analysis of a condylar support prosthesis to replace the temporomandibular joint. British Journal of Oral and Maxillofacial Surgery. 2015;53(4):352-7. | Title |
|  | Abrahim M. The Pleating Effect Explains the Cardioauricular Connection. Journal of Oral and Maxillofacial Surgery. 2021;79(2):273. | Title |
|  | Abukawa H, Ogawa T, Kono M, Koizumi T, Kawase-Koga Y, Chikazu D. Intravenous Dexamethasone Administration Before Orthognathic Surgery Reduces the Postoperative Edema of the Masseter Muscle: A Randomized Controlled Trial. Journal of Oral and Maxillofacial Surgery. 2017;75(6):1257-62. | Title |
|  | Abukawa H, Watanabe M, Asada Y, Satomi T, Matsuo A, Chikazu D. Ultrasound-guided intralesional photocoagulation of intramuscular vascular malformation in the masseter muscle. Journal of Oral and Maxillofacial Surgery. 2012;70(11):2674-9. | Title |
|  | Achmad H, Mutmainnah N, Ramadhany YF. A Systematic Review of Oral Myofunctional Therapy, Methods and Development of Class II Skeletal Malocclusion Treatment in Children. Systematic Reviews in Pharmacy. 2020;11(6):511-21. | Title |
|  | Achmad H, Safitri N, Paromova Y, Goncharov VV, Primarti RS, Riyanti E. Functional generating bite therapy in children during growth and development period. Indian Journal of Forensic Medicine and Toxicology. 2021;15(2):4125-33. | Title |
|  | Ackland DC, Moskaljuk A, Hart C, Vee Sin Lee P, Dimitroulis G. Prosthesis Loading after Temporomandibular Joint Replacement Surgery: A Musculoskeletal Modeling Study. Journal of Biomechanical Engineering. 2015;137(4). | Title |
|  | Acri TM, Shin K, Seol D, Laird NZ, Song I, Geary SM, et al. Tissue Engineering for the Temporomandibular Joint. Advanced Healthcare Materials. 2019;8(2). | Title |
|  | Actrn. Changes in masticatory mechanosensitivity, mouth opening and head posture after intervention with a myofascial induction protocol. https://trialsearchwhoint/Trial2aspx?TrialID=ACTRN12612000733875. 2012. | Title |
|  | Actrn. Effects of low level laser therapy after extraction of 3rd molar on mouth opening, pain and facial swelling. http://wwwwhoint/trialsearch/Trial2aspx?TrialID=ACTRN12616000181404. 2016. | Title |
|  | Actrn. Effects of craniofacial manual therapy on the masseter muscle morphology and postural stability in healthy adults. https://trialsearchwhoint/Trial2aspx?TrialID=ACTRN12623000540617. 2023. | Title |
|  | Adachi S, Takada K, Sakuda M, Lowe AA. Associations between jaw-opening muscle activity and craniofacial morphology. The Journal of Osaka University Dental School. 1989;29:25-32. | Full text  Other outcomes |
|  | Adanur Uzunlar E. Nutritional Problems Related to Oropharyngeal and Esophageal Changes in Aging: A Narrative Review. Journal of the American Nutrition Association. 2023;42(8):790-7. | Title |
|  | Addante RR. Masseter muscle hypertrophy: Report of case and literature review. Journal of Oral and Maxillofacial Surgery. 1994;52(11):1199-202. | Title |
|  | Adina S, Dipalma G, Bordea IR, Lucaciu O, Feurdean C, Inchingolo AD, et al. Orthopedic joint stability influences growth and maxillary development: Clinical aspects. Journal of Biological Regulators and Homeostatic Agents. 2020;34(3):747-56. | Abstract |
|  | Adisen MZ, Okkesim A, Misirlioglu M. A possible association between medial depression of mandibular ramus and maximum bite force. Folia Morphologica (Poland). 2018;77(4):711-6. | Title |
|  | Adnet PJ, Reyford H, Tavernier BM, Etchrivi T, Krivosic I, Krivosic-Horber R, Haudecoeur G. In vitro human masseter muscle hypersensitivity: A possible explanation for increase in masseter tone. Journal of Applied Physiology. 1996;80(5):1547-53. | Title |
|  | Afsar FS, Oziz E, Hamdioglu Y, Karasoy I, Uguz B. Intramuscular haemangioma of the masseter muscle in a 9-year-old girl. Acta Angiologica. 2007;13(1):42-6. | Title |
|  | Afshari FT, Parida A, Debenham P, Solanki GA. Myasthenia gravis complicating the surgical management of achondroplasia: a case-based update. Child's Nervous System. 2022;38(10):1855-9. | Title |
|  | Agnihotri A, Magu S, Dutta S. M-mode echomyography: Functional assessment of the effects of thumb-sucking habit on masticatory and circumoral musculature. Oral Radiology. 2010;26(2):71-6. | Full text  Other outcomes |
|  | Ahlberg JP, Kovero OA, Hurmerinta KA, Zepa I, Nissinen MJ, Könönen MH. Maximal bite force and its association with signs and symptoms of TMD, occlusion, and body mass index in a cohort of young adults. Cranio-the Journal of Craniomandibular & Sleep Practice. 2003;21(4):248-52. | Title |
|  | Ahlgren JGA, Ingervall BF, Thilander BL. Muscle activity in normal and postnormal occlusion. American Journal of Orthodontics. 1973;64(5):445-56. | Full text  Other outcomes |
|  | Ahmad K, Ansari S, Dhungel K, Gupta MK, Rauniyar RK, Amanullah MF. Myositis ossificans traumatica of the left masseter muscle presenting as soft tissue mass: A case report. Bangladesh Journal of Medical Science. 2014;13(1):73-5. | Title |
|  | Ahmad Tarmizi NE, Abdul Rahim S, Mohan Singh AS, Chooi LL, Ong FM, Lum SG. Parotid sialolithiasis and sialadenitis in a 3-year-old child: a case report and review of the literature. Egyptian Pediatric Association Gazette. 2020;68(1). | Title |
|  | Ahmed S, Al-Ibrahim RM, Alghuwainem NS, Alotaibi RM, Aljudhaie AF, Al Afaliq F, et al. EFFECTIVENESS OF VARIOUS MODALITIES OF PHYSIOTHERAPY INRELIEF OF PAIN ASSOCIATED WITH TEMPOROMANDIBULAR DISORDERS. A SYSTEMATIC REVIEW. European Journal of Molecular and Clinical Medicine. 2022;9(7):3992-4000. | Title |
|  | Ahuja AT, King AD, Bradley MJ, Yeo WW, Mok TSK, Metreweli C. Sonographic findings in masseter-muscle metastases. Journal of Clinical Ultrasound. 2000;28(6):299-302. | Title |
|  | Aijima R, Mori K, Danjo A, Ohishi M, Egashira R, Yamada Y, et al. A case of a malignant peripheral nerve sheath tumor on the cheek following trauma. Japanese Journal of Head and Neck Cancer. 2021;47(4):388-94. | Title |
|  | Akagi R, Kusama S. Comparison Between Neck and Shoulder Stiffness Determined by Shear Wave Ultrasound Elastography and a Muscle Hardness Meter. Ultrasound in Medicine and Biology. 2015;41(8):2266-71. | Title |
|  | Akan H, Aksöz T, Belet Ü, Şeşen T. Dynamic upper airway soft-tissue and caliber changes in healthy subjects and snoring patients. American Journal of Neuroradiology. 2004;25(10):1846-50. | Title |
|  | Akan S, Kocadereli I, Aktas A, Taşar F. Effects of maxillary molar intrusion with zygomatic anchorage on the stomatognathic system in anterior open bite patients. Eur J Orthod. 2013;35(1):93-102. | Title |
|  | Akbulut N, Altan A, Akbulut S, Atakan C. Evaluation of the 3 mm Thickness Splint Therapy on Temporomandibular Joint Disorders (TMDs). Pain Research and Management. 2018;2018. | Title |
|  | Akdemir B, Okkesim S, Kara S, Günes S. Correlation- and covariance-supported normalization method for estimating orthodontic trainer treatment for clenching activity. Proceedings of the Institution of Mechanical Engineers, Part H: Journal of Engineering in Medicine. 2009;223(8):991-1001. | Title |
|  | Akkaya S, Haydar S, Bilir E. Effects of spring-loaded posterior bite-block appliance on masticatory muscles. Am J Orthod Dentofacial Orthop. 2000;118(2):179-83. | Title |
|  | Akl A, Kuliralo M, Van Reck J. The frequency of various types of disk displacement and its relation to occlusion. Acta stomatologica Belgica. 1996;93(2):53-9. | Title |
|  | Aksoy S, Orhan K. Comparison of T2 Weighted, Fat-Suppressed T2 Weighted, and Three-Dimensional (3D) Fast Imaging Employing Steady-State Acquisition (FIESTA-C) Sequences in the Temporomandibular Joint (TMJ) Evaluation. BioMed Research International. 2021;2021. | Title |
|  | Al Ahmary AW, Alqhtani SM, Alshahrani BA, Alkaram WA, Alhadad BS, Elmarakby AM. Clinical applications of botulinum toxin in oral and maxillofacial surgery. Open Access Macedonian Journal of Medical Sciences. 2020;8:260-71. | Title |
|  | Al Sayegh S, Borgwardt A, Svensson KG, Kumar A, Grigoriadis A, Christidis N. Effects of Chronic and Experimental Acute Masseter Pain on Precision Biting Behavior in Humans. Frontiers in Physiology. 2019;10. | Title |
|  | Al-Ahmad HT, Al-Qudah MA. The treatment of masseter hypertrophy with botulinum toxin type A. Saudi Medical Journal. 2006;27(3):397-400. | Title |
|  | Al-Azzawi TR, Hamdan FB, Ali AK. Neurophysiologic evaluation of the temporomandibular joint and related masticatory muscles in rheumatoid arthritis patients. Neurosciences. 2008;13(3):253-8. | Title |
|  | Al-Belasy FA. Ultrasound-guided drainage of submasseteric space abscesses. Journal of Oral and Maxillofacial Surgery. 2005;63(1):36-41. | Title |
|  | Al-Belasy FA. TMD—Is It Destined for Oblivion? Journal of Oral and Maxillofacial Surgery. 2020;78(8):1221. | Title |
|  | Al-Farra ET, Vandenborne K, Swift A, Ghafari J. Magnetic resonance spectroscopy of the mas seter muscle in different facial morphological patterns. American Journal of Orthodontics and Dentofacial Orthopedics. 2001;120(4):427-34. | Full text  Other outcomes |
|  | Al-Fotawei R, Ayoub AF, Heath N, Naudi KB, Tanner KE, Dalby MJ, McMahon J. Radiological assessment of bioengineered bone in a muscle flap for the reconstruction of critical-size mandibular defect. PLoS One. 2014;9(9):e107403. | Title |
|  | Al-Gunaid TH. Bite force - What we should know: A literature review. International Journal of Orthodontic Rehabilitation. 2019;10(4):168-74. | Title |
|  | Al-Kalaly AA, Dyson JE, Wong RWK, Schätzle M, Cheung LK, Rabie ABM. Effect of different advancement positions on the maximum retrusive force of the mandible. Orthodontics and Craniofacial Research. 2013;16(1):56-64. | Title |
|  | Al-Muharraqi MA, Fedorowicz Z, Al Bareeq J, Al Bareeq R, Nasser M. Botulinum toxin for masseter hypertrophy. Cochrane Database of Systematic Reviews. 2009(3). | Title |
|  | Alabdullah M, Saltaji H, Abou-Hamed H, Youssef M. Association between facial growth pattern and facial muscle activity: A prospective cross-sectional study. International orthodontics / Collège européen d'orthodontie. 2015;13(2):181-94. | Full text  Other outcomes |
|  | Alabdullah MM, Saltaji H, Abou-Hamed H, Youssef M. The relationship between molar bite force and incisor inclination: A prospective cross-sectional study. International Orthodontics. 2014;12(4):494-504. | Title |
|  | Alajbeg IZ, Mestrovic S, Zlendic M, Zrinski MT, Vrbanovic E. Sudden, Severe, Idiopathic Occlusal Relationship Change Coexisting with Pain-Related Temporomandibular Disorders: A Case Report. Acta Stomatologica Croatica. 2022;56(4):405-16. | Title |
|  | Alani B, Alaqeeli AA, Aldoori M, Farag I. Giant cell tumour of the infra temporal fossa treated with denosumab in an elderly patient: A case report. Oral and Maxillofacial Surgery Cases. 2022;8(1). | Title |
|  | Alarcón JA, Martín C, Palma JC. Effect of unilateral posterior crossbite on the electromyographic activity of human masticatory muscles. American Journal of Orthodontics and Dentofacial Orthopedics. 2000;118(3):328-34. | Title |
|  | Alarcón JA, Martín C, Palma JC, Menéndez-Núñez M. Activity of jaw muscles in unilateral cross-bite without mandibular shift. Archives of Oral Biology. 2009;54(2):108-14. | Title |
|  | Aldemir K, Üstüner E, Erdem E, Demiralp AS, Oztuna D. Ultrasound evaluation of masseter muscle changes in stabilization splint treatment of myofascial type painful temporomandibular diseases. Oral Surgery Oral Medicine Oral Pathology Oral Radiology. 2013;116(3):377-83. | Title |
|  | Alexander TA, Gibbs CH, Thompson WJ. Investigation of chewing patterns in deep-bite malocclusions before and after orthodontic treatment. American Journal of Orthodontics. 1984;85(1):21-7. | Title |
|  | Alfaro-Moctezuma P, Osorno-Escareño MC, Nuño-Licona A, Leiva-Cartes F, Ángeles-Medina F. Effects of orthodontic treatment on the inhibitory masseteric reflex. Revista de Investigacion Clinica. 2003;55(3):289-96. | Title |
|  | Alhilou AM, Shimada A, Svensson CI, Svensson P, Ernberg M, Cairns BE, Christidis N. Sex-related differences in response to masseteric injections of glutamate and nerve growth factor in healthy human participants. Scientific Reports. 2021;11(1). | Title |
|  | Ali FM, Alsheri MIA, Shami SMA, Mohana AJI, Abujamilah EEI, Alshehri FAA. A Case Report of Bruxism and Its Management with The Help of Occlusal Splints. International Journal of Life Science and Pharma Research. 2023;13(2):L27-L30. | Title |
|  | Ali K, Sittampalam G, Malik MA. Bilateral temporalis hypertrophy. International Journal of Oral and Maxillofacial Surgery. 2010;39(3):305-7. | Title |
|  | Aliko A, Ciancaglini R, Alushi A, Tafaj A, Ruci D. Temporomandibular joint involvement in rheumatoid arthritis, systemic lupus erythematosus and systemic sclerosis. International Journal of Oral and Maxillofacial Surgery. 2011;40(7):704-9. | Title |
|  | Alkindi M, Alotaibi N, Alshiddi M. Inflammatory myofibroblastic tumour of the mandible-case report. International Journal of Oral and Maxillofacial Surgery. 2015;44:e184. | Title |
|  | AlKindi MG. A rare case of inflammatory myofibroblastic tumor of the mandible mimicking a malignant tumor. Saudi Dental Journal. 2017;29(1):36-40. | Title |
|  | Almond JR, Leroux BG, Knight DJ, Ramsay DS. Craniofacial morphology and tooth wear: A longitudinal study of orthodontic patients. Angle Orthodontist. 1999;69(1):7-13. | Full text  Other methods  & outcomes |
|  | Almotairy N. Biting and Chewing Behaviours in Humans : Development and Age-related Changes [Dissertation/Thesis]2020. | Title |
|  | Almotairy N, Kumar A, Grigoriadis A. Effect of food hardness on chewing behavior in children. Clin Oral Investig. 2021;25(3):1203-16. | Full text  Other methods  & outcomes |
|  | Almotairy N, Kumar A, Trulsson M, Grigoriadis A. Development of the jaw sensorimotor control and chewing - a systematic review. Physiology and Behavior. 2018;194:456-65. | Title |
|  | Almousa HM, Albesher MB, Alsolami AL, Al Mutairy AS. Intramuscular Hemangioma of the Sternocleidomastoid: A Rare Tumor in an Unusual Location. Ear, Nose and Throat Journal. 2023. | Title |
|  | Alnofaie H, Alshammeri T, Alturkistany Y, Aljabab A, Alweteid A. Giant Pleomorphic Adenoma of Parotid Gland in Saudi Arabia: a Rare Case Report. SN Comprehensive Clinical Medicine. 2020;2(8):1258-63. | Title |
|  | Alotaiby F. Ancient Schwannoma: Case Report of an Unusual Entity in an Unusual Oral Location. American Journal of Case Reports. 2022;23(1). | Title |
|  | Aloyouny AY, Mehanny MS, Albagieh HN, Alfaleh WM, Mansour SM, Mobarak FA. Intramuscular hemangioma in the zygomaticus muscle: A rare case report presentation and diagnosis. International Journal of Surgery Case Reports. 2020;74:42-5. | Title |
|  | Alshammari A, Almotairy N, Kumar A, Grigoriadis A. Effect of malocclusion on jaw motor function and chewing in children: a systematic review. Clin Oral Investig. 2022;26(3):2335-51. | Title |
|  | Amarasena JKC, Ariyawardana A, Amarasena N, Yamada Y. Mastication and swallowing in patients with oral submucous fibrosis. Asian Journal of Oral and Maxillofacial Surgery. 2007;19(3):145-9. | Title |
|  | Amarsaikhan B, Miura H, Okada D, Masuda T, Ishihara H, Shinki T, Kanno T. Influence of environmental factors on tooth displacement. Journal of Medical and Dental Sciences. 2002;49(1):19-26. | Title |
|  | Amarsaikhan B, Sukhbaatar N. Chewing hard food and its importance for general health. European Journal of Translational Myology. 2023;33(1):67. | Title |
|  | Ambrosio AR, Trevilatto PC, Sakima T, Ignácio SA, Shimizu RH. Correlation between morphology and function of the upper lip: A longitudinal evaluation. European Journal of Orthodontics. 2009;31(3):306-13. | Title |
|  | Anagnostou E, Lagos P, Plakas S, Mitsos A, Samelis A. Two-step treatment of a giant skull vault hemangioma: A rare case report and literature review. Neurocirugia. 2022;33(3):135-40. | Title |
|  | Anchlia S. Abdominal dermal fat graft versus nasolabial flap in oral submucous fibrosis: a randomised clinical trial. International journal of oral and maxillofacial surgery. 2021;51:e13‐e4. | Title |
|  | Andrade Ada S, Gameiro GH, Derossi M, Gavião MB. Posterior crossbite and functional changes. A systematic review. Angle Orthod. 2009;79(2):380-6. | Title |
|  | Andrade AdS, Gaviao MBD, Gameiro GH, De Rossi M. Characteristics of masticatory muscles in children with unilateral posterior crossbite. Brazilian Oral Research. 2010;24(2):204-10. | Title |
|  | Andrade AS, Gaviáo MBD, Derossi M, Gameiro GH. Electromyographic activity and thickness of masticatory muscles in children with unilateral posterior crossbite. Clinical Anatomy. 2009;22(2):200-6. | Full text  Cross-bites |
|  | Andrade N, Rajpari K, Mathai P, Aggarwal N, Nerurkar SA, Desai H. Intra-muscular haemangioma of the masseter: A clinical update and differential diagnosis of a rare entity. Journal of Stomatology, Oral and Maxillofacial Surgery. 2018;119(6):510-3. | Title |
|  | Andradea AD, Gameiro GH, DeRossi M, Gaviao MBD. Posterior Crossbite and Functional Changes. Angle Orthodontist. 2009;79(2):380-6. | Title |
|  | Anehosur V, Mehra A, Kumar N. Management of Masseter Muscle Hypertrophy and Role of Adjunctive Surgical Procedures. Craniomaxillofacial Trauma and Reconstruction Open. 2020;5. | Title |
|  | Ângelo DF, Sanz D, Maffia F, Cardoso HJ. Outcomes of IncobotulinumtoxinA Injection on Myalgia and Arthralgia in Patients Undergoing Temporomandibular Joint Arthroscopy: A Randomized Controlled Trial. Toxins. 2023;15(6). | Title |
|  | Anton SC. Masticatory muscle architecture and bone morphology in primates [Dissertation/Thesis]1994. | Title |
|  | Anton SC. Tendon-associated bone features of the masticatory system in Neandertals. Journal of Human Evolution. 1996;31(5):391-408. | Title |
|  | Antón SC. Macaque masseter muscle:: Internal architecture, fiber length and cross-sectional area. International Journal of Primatology. 1999;20(3):441-62. | Title |
|  | Antonarakis GS, Herzog G, Kiliaridis S. Vertical relapse after orthodontic and orthognathic surgical treatment in a patient with myotonic dystrophy. European Journal of Paediatric Dentistry. 2019;20(1):53-8. | Title |
|  | Antonarakis GS, Kiliaridis S. Predictive value of masseter muscle thickness and bite force on Class II functional appliance treatment: a prospective controlled study. Eur J Orthod. 2015;37(6):570-7. | Title |
|  | Antonarakis GS, Kiliaridis S. Treating Class II malocclusion in children. Vertical skeletal effects of high-pull or low-pull headgear during comprehensive orthodontic treatment and retention. Orthodontics & Craniofacial Research. 2015;18(2):86-95. | Title |
|  | Antonarakis GS, Kjellberg H, Kiliaridis S. Predictive value of molar bite force on Class II functional appliance treatment outcomes. European Journal of Orthodontics. 2012;34(2):244-9. | Full text  Only Class IIs |
|  | Antonarakis GS, Kjellberg H, Kiliaridis S. Bite force and its association with stability following Class II/1 functional appliance treatment. Eur J Orthod. 2013;35(4):434-41. | Full text  Only Class IIs |
|  | Antonopoulou M, Iatrou I, Paraschos A, Anagnostopoulou S. Variations of the attachment of the superior head of human lateral pterygoid muscle. Journal of Cranio-Maxillofacial Surgery. 2013;41(6):e91-e7. | Title |
|  | Antoun JS, Thomson WM, Merriman TR, Farella M. Self-reported oral behaviour habits in hyperdivergent and normodivergent facial types. Journal of Oral Rehabilitation. 2017;44(1):16-21. | Title |
|  | Anyonge W, Baker A. Craniofacial morphology and feeding behavior in <i>Canis dirus</i>, the extinct Pleistocene dire wolf. Journal of Zoology. 2006;269(3):309-16. | Title |
|  | Anzelmo M, Barbeito-Andrés J, Ventrice F, Pucciarelli HM, Sardi ML. Ontogenetic patterns of morphological variation in the ectocranial human vault. Anatomical Record. 2013;296(7):1008-15. | Abstract |
|  | Anzelmo M, Ventrice F, Kelmansky D, Sardi M. Complex pattern of variation in neurocranial ontogeny revealed by CT-scanning. Anthropol Anz. 2018;75(2):113-30. | Abstract |
|  | Aoki T, Naito H, Ota Y, Shiiki K. Myositis ossificans traumatica of the masticatory muscles: Review of the literature and report of a case. Journal of Oral and Maxillofacial Surgery. 2002;60(9):1083-8. | Title |
|  | Aoun M, Mesnard M, Monède-Hocquard L, Ramos A. Stress analysis of temporomandibular joint disc during maintained clenching using a viscohyperelastic finite element model. Journal of Oral and Maxillofacial Surgery. 2014;72(6):1070-7. | Title |
|  | Aquilina P, Chamoli U, Parr WCH, Clausen PD, Wroe S. Finite element analysis of three patterns of internal fixation of fractures of the mandibular condyle. British Journal of Oral and Maxillofacial Surgery. 2013;51(4):326-31. | Title |
|  | Aquino VM, Rock JP, Perry KD, Barbetta BT. Functional reconstruction of the glenoid fossa utilizing a pedicled temporal osteomuscular flap. Oral and Maxillofacial Surgery Cases. 2022;8(1). | Title |
|  | Aragón N, Díaz C, Contreras A. Dental, Occlusal, and Craniofacial Features of Children With Microcephaly Due to Congenital Zika Infection: 3 Cases Report From Valle del Cauca, Cali—Colombia—2020. Cleft Palate-Craniofacial Journal. 2021;58(10):1318-25. | Title |
|  | Arakawa M, Kitahara T, Inadomi D, Iikubo M, Hyakutake H, Yuasa K, Takahashi I. Molecular imaging in masseter muscle observed by muscle function magnetic resonance imaging and (31) P-magnetic resonance spectroscopy in patients with a jaw deformity. Clin Exp Dent Res. 2022;8(1):231-8. | Title |
|  | Arat FE, Arat ZM, Acar M, Beyazova M, Tompson B. Muscular and condylar response to rapid maxillary expansion. Part 1: Electromyographic study of anterior temporal and superficial masseter muscles. American Journal of Orthodontics and Dentofacial Orthopedics. 2008;133(6):815-22. | Title |
|  | Araujo DS, Marquezin MC, Barbosa Tde S, Gavião MB, Castelo PM. Evaluation of masticatory parameters in overweight and obese children. Eur J Orthod. 2016;38(4):393-7. | Title |
|  | Araya T, Kasahara K, Fujimura M, Tanbo Y, Tamori S, Sone T, Nakao S. Three cases of non-small cell lung cancer associated with skeletal muscle metastases. Japanese Journal of Lung Cancer. 2006;46(2):117-25. | Title |
|  | Arda K, Ciledag N, Aktas E, Aribas BK, Köse K. Quantitative assessment of normal soft-tissue elasticity using shear-wave ultrasound elastography. American Journal of Roentgenology. 2011;197(3):532-6. | Title |
|  | Arenaz Búa J, Luáces R, Lorenzo Franco F, García-Rozado A, Crespo Escudero JL, Fonseca Capdevila E, López-Cedrún JL. Angiolipoma in head and neck: report of two cases and review of the literature. International Journal of Oral and Maxillofacial Surgery. 2010;39(6):610-5. | Title |
|  | Ariji E, Moriguchi S, Kuroki T, Kanda S. Computed tomography of maxillofacial infection. Dentomaxillofac Radiol. 1991;20(3):147-51. | Title |
|  | Ariji Y, Fuwa N, Tachibana H, Ariji E. Denervation atrophy of the masticatory muscles in a patient with nasopharyngeal cancer: MR examinations before and after radiotherapy. Dentomaxillofacial Radiology. 2002;31(3):204-8. | Title |
|  | Ariji Y, Fuwa N, Toyama M, Katoh M, Gotoh M, Ariji E. MR features of masticatory muscles in adenoid cystic carcinoma involving the masticator space. Dentomaxillofacial Radiology. 2004;33(5):345-50. | Title |
|  | Ariji Y, Kawamata A, Yoshida K, Sakuma S, Nawa H, Fujishita M, Ariji E. Three-dimensional morphology of the masseter muscle in patients with mandibular prognathism. Dentomaxillofac Radiol. 2000;29(2):113-8. | Included |
|  | Ariji Y, Sakuma S, Kimura Y, Kawamata A, Toyama M, Kurita K, et al. Colour Doppler sonographic analysis of blood-flow velocity in the human facial artery and changes in masseter muscle thickness during low-level static contraction. Archives of Oral Biology. 2001;46(11):1059-64. | Abstract |
|  | Arima T, Arendt-Nielsen L, Minagi S, Svensson P. Effect of capsaicin-evoked jaw-muscle pain on intramuscular blood-flow. Archives of Oral Biology. 2009;54(3):241-9. | Title |
|  | Arima T, Svensson P, Arendt-Nielsen L. Capsaicin-induced muscle hyperalgesia in the exercised and non-exercised human masseter muscle. Journal of Orofacial Pain. 2000;14(3):213-23. | Title |
|  | Arima T, Takeuchi T, Honda K, Tomonaga A, Tanosoto T, Ohata N, Svensson P. Effects of interocclusal distance on bite force and masseter EMG in healthy participants. Journal of Oral Rehabilitation. 2013;40(12):900-8. | Title |
|  | Armellini M, Sánchez L, Lorek A, Shelton GD, De Risio L. Clinical presentation, MRI findings, histopathology and outcome in a cat with masticatory myositis. Journal of Veterinary Internal Medicine. 2020;34(6):3051. | Title |
|  | Arnaud-Brachet M, Foletti JM, Graillon N, Chaumoître K, Chossegros C, Guyot L. Could mastication modify the shape of the orbit? A scannographic study in humans. Surgical and Radiologic Anatomy. 2020;42(1):63-7. | Abstract |
|  | Arroyo-Morales M, Olea N, Martínez MM, Hidalgo-Lozano A, Ruiz-Rodríguez C, Díaz-Rodríguez L. Psychophysiological effects of massage-myofascial release after exercise: a randomized sham-control study. Journal of alternative and complementary medicine (New York, NY). 2008;14(10):1223‐9. | Title |
|  | Arsenina OI, Shishkin KM, Shishkin MK, Popova AV. [Adaptive dentoalveolar changes by insufficient sizes of the jaws]. Stomatologii{combining double inverted breve}a. 2013;92(5):29-37. | Title |
|  | Arslan ZB, Yaşar F. Evaluation of the thickness and internal structure of the masseter muscle with ultrasonography in female bruxism patients. Oral Radiology. 2023;39(4):708-14. | Title |
|  | Arteaga G, Schiltz B. Trismus and rhabdomyolysis associated with sertraline in a pediatric patient. Critical Care Medicine. 2013;41(12):A293. | Title |
|  | Arun RM, Lakkakula BV, Chitharanjan AB. Role of myosin 1H gene polymorphisms in mandibular retrognathism. Am J Orthod Dentofacial Orthop. 2016;149(5):699-704. | Title |
|  | Arun T, Kayhan F, Kiziltan M. Treatment of Condylar Hypoplasia with Distraction Osteogenesis: A Case Report. Angle Orthodontist. 2002;72(4):371-6. | Title |
|  | Arutiunov SD, Persin LS, Petrosian DE, Arutiunov DS. Correlation of x-ray cephalometric parameters of the gnathic part of the skull with dental anthropometric parameters and data of functional tests in subjects with normal occlusion. Stomatologiya. 2001;80(5):40-6. | Abstract |
|  | Asada K, Usui H, Nakayama A, Nagashima H, Ishibashi K. A case of Garrè's osteomyelitis of the mandible associated with perimandibular abscess. Oral Therapeutics and Pharmacology. 2007;26(2):61-7. | Title |
|  | Asami T, Ishizaki A, Ogawa A, Kwon H, Murakami K, Tanaka A, Hironaka S. Basic research of pediatric dysphagiaidentification of factors associated with tongue pressure during childhood. Dysphagia. 2018;33(4):571-2. | Title |
|  | Asano T, Kawara M, Suzuki H, Komiyama O, Fukumoto M, Iida T. Masticatory muscle activity during exertion of the back. Nihon Hotetsu Shika Gakkai zasshi. 2006;50(1):45-53. | Title |
|  | Askar H, Aronovich S, Christensen BJ, McCain J, Hakim M. Is Arthroscopic Disk Repositioning Equally Efficacious to Open Disk Repositioning? A Systematic Review. Journal of Oral and Maxillofacial Surgery. 2021;79(10):2030-41.e2. | Title |
|  | Aspestrand F, Boysen M. CT and MR imaging of primary tumors of the masticator space. Acta Radiologica. 1992;33(6):518-22. | Title |
|  | Athanasiou AE. Morphologic and functional implications of the surgical-orthodontic management of mandibular prognathism: A comprehensive review. American Journal of Orthodontics and Dentofacial Orthopedics. 1993;103(5):439-47. | Title |
|  | Auliya P. Autonomic Dysfunction P-AD001. Gastroparesis as autonomic manifestation of myasthenia gravis: A rare case report. Clinical Neurophysiology. 2021;132(8):e94-e5. | Title |
|  | Auluck A, Mudera V, Hunt NP, Lewis MP. A three-dimensional in vitro model system to study the adaptation of craniofacial skeletal muscle following mechanostimulation. Eur J Oral Sci. 2005;113(3):218-24. | Title |
|  | Aung PT, Kato C, Abe Y, Ogawa T, Ishidori H, Fujita A, et al. Functional Analysis of Rhythmic Jaw Movements Evoked by Electrical Stimulation of the Cortical Masticatory Area During Low Occlusal Loading in Growing Rats. Frontiers in Physiology. 2020;11. | Title |
|  | Aung PT, Kato C, Fujita A, Abe Y, Ogawa T, Ishidori H, et al. Effects of low occlusal loading on the neuromuscular behavioral development of cortically-elicited jaw movements in growing rats. Scientific Reports. 2021;11(1). | Title |
|  | Awachat A, Jadhav V, Reche A. Correlation of Mandibular Angle and Malocclusion with Hand Grip Strength in Vertical, Horizontal and Average Grower. Journal of Research in Medical and Dental Science. 2022;10(9):30-+. | Title |
|  | Awachat A, Jadhav V, Reche A, Nerurkar S, Paul P, Kakde K, Taori D. Association of Gonial Angle with Hand Grip Strength in Vertical, Horizontal and Average Growers: A Cross-sectional Study. Journal of Clinical and Diagnostic Research. 2022;16(12):7-11. | Title |
|  | Aydin Aksu S, Kursoglu P, Turker I, Baskak F, Ozen Sutuven E, Meric K, Cabbar F. Dynamic Quantitative Imaging of the Masseter Muscles in Bruxism Patients with Myofascial Pain: Could It Be an Objective Biomarker? Journal of Personalized Medicine. 2023;13(10). | Title |
|  | Azami N, Nanda R, Uribe F. Effective Vertical Control of the Entire Maxillary Arch with a Palatal TAD-Supported Appliance. Journal of Clinical Orthodontics. 2020;54(10):620-9. | Title |
|  | Azaroual MF, Fikri M, Abouqal R, Benyahya H, Zaoui F. Relationship between dimensions of muscles of mastication (masseter and lateral pterygoid) and skeletal dimensions: study of 40 cases. Int Orthod. 2014;12(1):111-24. | Included |
|  | Azlag Pekince K, Caglayan F, Pekince A. Imaging of masseter muscle spasms by ultrasonography: a preliminary study. Oral Radiology. 2020;36(1):85-8. | Title |
|  | Baba K, Ai M, Mizutani H, Enosawa S. Influence of experimental occlusal discrepancy on masticatory muscle activity during clenching. Journal of Oral Rehabilitation. 1996;23(1):55-60. | Title |
|  | Baba K, Tsukiyama Y, Clark GT. Reliability, validity, and utility of various occlusal measurement methods and techniques. Journal of Prosthetic Dentistry. 2000;83(1):83-9. | Title |
|  | Baba K, Yugami K, Yaka T, Ai M. Impact of balancing-side tooth contact on clenching induced mandibular displacements in humans. Journal of Oral Rehabilitation. 2001;28(8):721-7. | Title |
|  | Baba M. [Some characteristics of orthodontic treatment in high mandibular cases]. Aichi Gakuin Daigaku Shigakkai Shi. 1990;28(1 Pt 2):499-507. | Title |
|  | Baccarani A, Follmar KE, Erdmann D, Levin LS. Face transplantation surgical options and open problems in cadaveric models: A review article. Microsurgery. 2013;33(3):239-46. | Title |
|  | Backlund E. Facial growth, and the significance of oral habits, mouthbreathing and soft tissues for malocclusion. A study on children around the age of 10. Acta Odontologica Scandinavica. 1963;21:Suppl36:9-139. | Title |
|  | Badel T, Marotti M, Pavičin IS, Bašić-Kes V. Temporomandibular disorders and occlusion. Acta Clinica Croatica. 2012;51(3):419-24. | Title |
|  | Baduni A, Krishnamoorthy B. Treatment of hemifacial spasm in patient with hemifacial atrophy using combination therapy (ultrasound therapy and TENS): A case report. Korean Journal of Pain. 2017;30(4):304-7. | Title |
|  | Bae H, Kim J, Seo KK, Hu KS, Kim ST, Kim HJ. Comparison between Conventional Blind Injections and Ultrasound-Guided Injections of Botulinum Toxin Type A into the Masseter: A Clinical Trial. Toxins. 2020;12(9). | Title |
|  | Baéz A, Paleari J, Durán MN, Rudy T, Califano I, Barbosa N, Casas Parera I. Síndrome de Frey por submaxilectomía y tratamiento con toxina botulínica. Medicina (BAires). 2007;67(5):478-80. | Title |
|  | Bai S, Yu Y, Zhang WB, Mao YQ, Wang Y, Mao C, Peng X. Three-dimensional attachment morphometry and volumetric changes of masticatory muscles after free fibular flap reconstruction of the mandibular condyle. Journal of Cranio-Maxillofacial Surgery. 2022;50(1):19-25. | Title |
|  | Bai Y, Tang Y, Ren M, Wang M, Zhao W, Zeng T, et al. Orofacial myofunctional changes in skeletal Class III patients after bimaxillary orthognathic surgery. Journal of Plastic, Reconstructive and Aesthetic Surgery. 2022;75(9):3526-33. | Title |
|  | Baird T, Cuff A, Fitton L. Using musculoskeletal modelling to investigate the functional significance of craniofacial form variation within the genus Homo. Journal of Anatomy. 2023;242(4):724-5. | Title |
|  | Bakathir A, Al-Azri S. Infantile cortical hyperostosis presenting as painful bilateral swelling of the mandible. International Journal of Oral and Maxillofacial Surgery. 2017;46:300. | Title |
|  | Bakke M. Occlusal equilibration by grinding as a treatment of malocclusion causing functional disturbances. Tandlaegebladet. 1981;85(20):657-67. | Title |
|  | Bakke M. Mandibular elevator muscles: physiology, action, and effect of dental occlusion. Scand J Dent Res. 1993;101(5):314-31. | Title |
|  | Bakke M, Michler L. Temporalis and masseter muscle activity in patients with anterior open bite and craniomandibular disorders. Scand J Dent Res. 1991;99(3):219-28. | Title |
|  | Bakke M, Michler L, Möller E. Occlusal control of mandibular elevator muscles. European Journal of Oral Sciences. 1992;100(5):284-91. | Title |
|  | Bakke M, MØLler E. Distortion of maximal elevator activity by unilateral premature tooth contact. European Journal of Oral Sciences. 1980;88(1):67-75. | Title |
|  | Bakke M, Moller E. CRANIOMANDIBULAR DISORDERS AND MASTICATORY MUSCLE FUNCTION. Scandinavian Journal of Dental Research. 1992;100(1):32-8. | Title |
|  | Bakke M, Stoltze K, Tuxen A. Variables related to masseter muscle function: a maximum R2 improvement analysis. Scand J Dent Res. 1993;101(3):159-65. | Title |
|  | Bakke M, Thomsen CE, Vilmann A, Soneda K, Farella M, Møller E. Ultrasonographic assessment of the swelling of the human masseter muscle after static and dynamic activity. Arch Oral Biol. 1996;41(2):133-40. | Title |
|  | Baklacı D, Güngör V, Özcan M, Yılmaz YF, Ünal A, Çolak A. Adenoid cystic carcinoma of the accessory parotid gland. Kulak burun boğaz ihtisas dergisi : KBB = Journal of ear, nose, and throat. 2015;25(5):302-5. | Title |
|  | Bakor SF, Enlow DH, Pontes P, De Biase NG. Craniofacial growth variations in nasal-breathing, oral-breathing, and tracheotomized children. American Journal of Orthodontics and Dentofacial Orthopedics. 2011;140(4):486-92. | Title |
|  | Bakradze A. Peculiarities of the chewing muscles electrophysiological activity in mouth breathing individuals. European Journal of Translational Myology. 2023;33(1):67-8. | Title |
|  | Balaban E, Yılmaz O, Tımarcıoğlu G, Bahran U, Candirli C, Koşucu P. Preoperative and postoperative assessment of temporal and masseter muscle size with magnetic resonance imaging in patients undergoing unilateral temporomandibular joint surgery. Journal of Cranio-Maxillofacial Surgery. 2021;49(8):705-10. | Title |
|  | Balakrishnan K, Ebenezer V, Dakir A, Kumar SK, Prakash D. Management of tripod fractures (zygomaticomaxillary complex) 1 point and 2 point fixations: A 5-year review. Journal of Pharmacy and Bioallied Sciences. 2015;7:S242-S7. | Title |
|  | Balakrishnan PK, Kumar SM, Chippala P, Hegde C. An in vivo electromyographic evaluation of pain relief using different therapies in masticatory myalgia patients. Journal of the Korean Association of Oral and Maxillofacial Surgeons. 2020;46(5):321‐7. | Title |
|  | Balanta-Melo J, Torres-Quintana MA, Bemmann M, Vega C, González C, Kupczik K, et al. Masseter muscle atrophy impairs bone quality of the mandibular condyle but not the alveolar process early after induction. Journal of Oral Rehabilitation. 2019;46(3):233-41. | Abstract |
|  | Balcioglu HA, Uyanikgil Y, Yuruker S, Tuna HS, Karacayli U. Volumetric assessment of lateral pterygoid muscle in unilateral chewing: A stereologic study. Journal of Craniofacial Surgery. 2009;20(5):1364-6. | Full text  Irrelevant groups  No craniofacial patterns |
|  | Baldini A, Nota A, Cioffi C, Ballanti F, Cozza P. Infrared thermographic analysis of craniofacial muscles in military pilots affected by bruxism. Aerosp Med Hum Perform. 2015;86(4):374-8. | Title |
|  | Baltrusaityte A, Surna A, Pileicikiene G, Kubilius R, Gleiznys A, Baltrusaitis M. Dynamical changes of occlusion and articulation during treatment of mandibular angle fractures. Stomatologija / issued by public institution "Odontologijos studija" [et al]. 2013;15(1):12-9. | Title |
|  | Banakar VT, Bhandage S, Kumar AH, Swennen GRJ. Resistance and Stress Analysis of 3D Plate and Locking Plate Fixation in Bilateral Sagittal Split Osteotomy: A Comparative Finite Element Study. Journal of Maxillofacial & Oral Surgery. 2023. | Title |
|  | Banerjee A, Basu B, Saha S, Chowdhury AR. Design and development of a patient-specific temporomandibular joint implant: Probing the influence of bone condition on biomechanical response. Journal of Biomedical Materials Research - Part B Applied Biomaterials. 2023;111(12):2089-97. | Title |
|  | Bao D, Zhao Y, Liu Z, Zhong H, Geng Y, Lin M, et al. Prognostic and predictive value of radiomics features at MRI in nasopharyngeal carcinoma. Discover Oncology. 2021;12(1). | Title |
|  | Barakat D, Bakdach WMM, Youssef M. Treatment effects of Carriere Motion Appliance on patients with class II malocclusion: A systematic review and meta-analysis. International Orthodontics. 2021;19(3):353-64. | Title |
|  | Barbarino GG, Jabareen M, Mazza E. Experimental and numerical study on the mechanical behavior of the superficial layers of the face. Skin Research and Technology. 2011;17(4):434-44. | Abstract |
|  | Barber TK, Bonus HW. Dental relationships in tongue-thrusting children as affected by circumoral myofunctional exercise. Journal of the American Dental Association (1939). 1975;90(5):979-88. | Title |
|  | Barbosa TdS, Miyakoda LS, Pocztaruk RdL, Rocha CP, Gavião MBD. Temporomandibular disorders and bruxism in childhood and adolescence: Review of the literature. International Journal of Pediatric Otorhinolaryngology. 2008;72(3):299-314. | Title |
|  | Bardinet E, Bazert C, Boileau MJ, Carat T, Darqué F, de Brondeau F, et al. Vertical control and orthopedic therapy. L' Orthodontie française. 2003;74(3):377-409. | Title |
|  | Barlow ST, Drage NA, Thomas DW. Ectopic submandibular gland presenting as a swelling in the floor of mouth. Journal of Laryngology and Otology. 2005;119(11):928-30. | Title |
|  | Barotsis N, Michail X, Panagiotopoulos E. The role of musculoskeletal ultrasonography in the diagnosis of sarcopenia. Journal of Musculoskeletal Neuronal Interactions. 2021;21(1):178-9. | Title |
|  | Barotsis N, Tsiganos P, Kokkalis Z, Panayiotakis G, Panagiotopoulos E. Reliability of muscle thickness measurements in ultrasonography. International Journal of Rehabilitation Research. 2020;43(2):123-8. | Abstract |
|  | Barrera LM, Buschang PH, Throckmorton GS, Roldán SI. Mixed longitudinal evaluation of masticatory performance in children 6 to 17 years of age. American Journal of Orthodontics and Dentofacial Orthopedics. 2011;139(5):E427-E34. | Title |
|  | Barrientos E, Pelayo F, Tanaka E, Lamela-Rey MJ, Fernández-Canteli A, de Vicente JC. Effects of loading direction in prolonged clenching on stress distribution in the temporomandibular joint. Journal of the Mechanical Behavior of Biomedical Materials. 2020;112. | Title |
|  | Barriere P, Lutz JC, Zamanian A, Wilk A, Rhiem S, Veillon F, Kahn JL. MRI evidence of lateral pterygoïd muscle palpation. International Journal of Oral and Maxillofacial Surgery. 2009;38(10):1094-5. | Abstract |
|  | Barros TS, Santos MB, Shinozaki EB, Santos JF, Marchini L. Effects of use of anabolic steroids on the masticatory system: a pilot study. J Oral Sci. 2008;50(1):19-24. | Title |
|  | Barthélémy I, Karanas Y, Sannajust JP, Emering C, Mondié JM. Gout of the temporomandibular joint: Pitfalls in diagnosis. Journal of Cranio-Maxillofacial Surgery. 2001;29(5):307-10. | Title |
|  | Baruah N. Assessment of the Temporomandibular Joint Morphology in Temporomandibular Joint Dysfunction – A Cone Beam Computed Tomography Study [Dissertation/Thesis]2017. | Title |
|  | Baş B, Özan B, Muǧlali M, Çelebi N. Treatment of masseteric hypertrophy with botulinum toxin: A report of two cases. Medicina Oral, Patologia Oral y Cirugia Bucal. 2010;15(4):e649-e52. | Title |
|  | Bas B, Ylmaz N, Gkce E, Akan H. Diagnostic value of ultrasonography in temporomandibular disorders. Journal of Oral and Maxillofacial Surgery. 2011;69(5):1304-10. | Title |
|  | Basafa E, Murphy RJ, Gordon CR, Armand M. Modeling the biomechanics of swine mastication - An inverse dynamics approach. Journal of Biomechanics. 2014;47(11):2626-32. | Title |
|  | Bashiri R, Luke A, Weiner S. Comparative Analysis of Sensory Responses from Dental Implants vs Natural Teeth: An In Vivo Study. International Journal of Oral & Maxillofacial Implants. 2023;38(2):321-7. | Title |
|  | Basi DL, Velly AM, Schiffman EL, Lenton PA, Besspiata DA, Rankin AM, et al. Human temporomandibular joint and myofascial pain biochemical profiles: a case-control study. Journal of Oral Rehabilitation. 2012;39(5):326-37. | Title |
|  | Bates KT, Wang LJ, Dempsey M, Broyde S, Fagan MJ, Cox PG. Back to the bones: do muscle area assessment techniques predict functional evolution across a macroevolutionary radiation? Journal of the Royal Society Interface. 2021;18(180). | Title |
|  | Battista G, Guida L, Avvanzo P, Mastrovincenzo M, Chimenti C, Muzio LL, Ciavarella D. Instrumental evaluation of angle class II malocclusion. Mondo Ortodontico. 2011;36(4):175-82. | Title |
|  | Baumrind S, Peltzman P, Moffitt FH. New system for integrated three dimensional craniofacial mapping. Journal of Dental Research. 1971;50(6):1496-7. | Title |
|  | Baur DA, Jannuzzi JR, Mercan U, Quereshy FA. Treatment of long term anterior dislocation of the TMJ. International Journal of Oral and Maxillofacial Surgery. 2013;42(8):1030-3. | Title |
|  | Baurmash HD. Sialoendoscopy Versus Conventional Surgical Sialolithotomy. Journal of Oral and Maxillofacial Surgery. 2008;66(2):412-5. | Title |
|  | Baverstock H. Craniofacial integration, plasticity and biomechanics in the mouse masticatory system. 2014. | Title |
|  | Baverstock H, Jeffery NS, Cobb SN. The morphology of the mouse masticatory musculature. Journal of Anatomy. 2013;223(1):46-60. | Title |
|  | Baykul T, Aydin M, Aksoy M, Findik Y. Unusual unilateral fracture of the condylar and coronoid processes of the mandible. Journal of Clinical Imaging Science. 2014;4:3. | Title |
|  | Bayrak NB, Zeybek M, Sanlioglu I, Dolanmaz D. Assessment of changes in masseter muscle by three-dimensional close-range photogrammetry after botulinum toxin type-A injection: A case report with review of literature. Journal of the Pakistan Medical Association. 2019;69(3):418-22. | Title |
|  | Bayram B, Uckan S, Cetinsahin A, Ozcirpici AA, Ozdemir H, Yazici C. Repositioning of the masseter muscle and its effect on skeletal growth. Oral Surgery Oral Medicine Oral Pathology Oral Radiology and Endodontology. 2010;109(5):E1-E5. | Abstract |
|  | Bechara J. Relationships between Craniofacial Muscle Morphology and Dietary Specializations across Mammal Phylogeny [Dissertation/Thesis]2021. | Title |
|  | Becht MP. A study using Three-Dimensional Cone Beam Computed Tomography to evaluate masseter muscle morphology in observed skeletal malocclusions [Dissertation/Thesis]2009. | Included |
|  | Becht MP, Mah J, Martin C, Razmus T, Gunel E, Ngan P. Evaluation of masseter muscle morphology in different types of malocclusions using cone beam computed tomography. Int Orthod. 2014;12(1):32-48. | Included |
|  | Becker OE, Avelar RL, Rivero ER, De Oliveira RB, Meurer MI, Santos AM, et al. Myositis Ossificans of the Temporalis Muscle. Head Neck Pathol. 2016;10(3):340-4. | Title |
|  | Becker P, Pabst A, Thiele O, Rudat J, Werkmeister R. Adverse side effects with hyaluronic acid fillers: A case report. Advances in Oral and Maxillofacial Surgery. 2021;2. | Title |
|  | Beckley ML, Ghafourpour KL, Indresano AT. The Use of Argon Beam Coagulation to Control Hemorrhage: A Case Report and Review of the Technology. Journal of Oral and Maxillofacial Surgery. 2004;62(5):615-8. | Title |
|  | Bekcioglu B, Bulut E, Bas B. The Effects of Unilateral Alloplastic Temporomandibular Joint Replacement on the Opposite-Side Natural Joint: A Finite-Element Analysis. Journal of Oral and Maxillofacial Surgery. 2017;75(11):2316-22. | Title |
|  | Bekri S, Trifi W, Labidi A, Bizani C, Mansour L. Full-mouth rehabilitation of an acromegaly disease patient with removable prostheses: A clinical case report. Pan African Medical Journal. 2019;33. | Title |
|  | Belleflamme MM, Geerts SO, Louwette MM, Grenade CF, Vanheusden AJ, Mainjot AK. No post-no core approach to restore severely damaged posterior teeth: An up to 10-year retrospective study of documented endocrown cases. Journal of Dentistry. 2017;63:1-7. | Title |
|  | Belmonte-Caro R, Garcia-Perla-Garcia A, Martinez-de-Fuentes R, Infante-Cossio P. Penetrating Glass Foreign Body in the Deep Temporal Space. Journal of Emergency Medicine. 2019;57(2):e61-e3. | Title |
|  | Bencini CA. Biprotrusion. Revista de la Sociedad Odontologica de La Plata. 1990;3(5):11-4. | Title |
|  | Benington PCM, Gardener JE, Hunt NP. Masseter muscle volume measured using ultrasonography and its relationship with facial morphology. European Journal of Orthodontics. 1999;21(6):659-70. | Included |
|  | Benoit R, Falque E. Interactions between the muscles and therapy. L" Orthodontie francaise. 1992;63 Pt 2:341-5;discussion7. | Title |
|  | Bergamaschi L, Marvaso G, Zaffaroni M, Vincini MG, D’Ecclesiis O, Volpe S, et al. Prognostic Impact of Sarcopenia’s Occurrence during Radiotherapy in Oropharyngeal Cancer Patients. Cancers. 2023;15(3). | Title |
|  | Bergamaschi L, Zaffaroni M, Vincini MG, Marvaso G, Volpe S, Colombo F, et al. Sarcopenia in oropharyngeal cancer treated with curative radiotherapy: time for a tailored approach? Radiotherapy and Oncology. 2023;182:S937-S8. | Title |
|  | Bergman H, Andersson F, Isberg A. Incidence of temporomandibular joint changes after whiplash trauma: A prospective study using MR imaging. American Journal of Roentgenology. 1998;171(5):1237-43. | Title |
|  | Bermell-Baviera A, Bellot-Arcís C, Montiel-Company JM, Almerich-Silla JM. Effects of mandibular advancement surgery on the temporomandibular joint and muscular and articular adaptive changes—a systematic review. International Journal of Oral and Maxillofacial Surgery. 2016;45(5):545-52. | Title |
|  | Berni KCDS, Dibai-Filho AV, Pires PF, Rodrigues-Bigaton D. Accuracy of the surface electromyography RMS processing for the diagnosis of myogenous temporomandibular disorder. Journal of Electromyography and Kinesiology. 2015;25(4):596-602. | Title |
|  | Bernkopf E, Colleselli P, Broia V, De Benedictis FM. Is recurrent parotitis in childhood still an enigma? A pilot experience. Acta Paediatrica, International Journal of Paediatrics. 2008;97(4):478-82. | Title |
|  | Bertot BE, Presti ML, Stormes K, Raskin JS, Jea A, Chelius D, Lam S. Trigeminal schwannoma presenting with malocclusion: A case report and review of the literature. Surgical Neurology International. 2020;11. | Title |
|  | Beukes J, Reyneke JP, Becker PJ. Medial pterygoid muscle and stylomandibular ligament: The effects on postoperative stability. International Journal of Oral and Maxillofacial Surgery. 2013;42(1):43-8. | Title |
|  | Bezerra TP, Silva Jr FI, Scarparo HC, Costa FWG, Studart-Soares EC. Do erupted third molars weaken the mandibular angle after trauma to the chin region? A 3D finite element study. International Journal of Oral and Maxillofacial Surgery. 2013;42(4):474-80. | Title |
|  | Bhat KK, Chinnappa AG, Mohan M, Shetty H, Banerjee S. Intraocular foreign body-a peculiar challenge. Nitte University Journal of Health Science. 2015;5(2):84-7. | Title |
|  | Bhutada MK, Phanachet I, Whittle T, Peck CC, Murray GM. Regional properties of the superior head of human lateral pterygoid muscle. European Journal of Oral Sciences. 2008;116(6):518-24. | Title |
|  | Biglioli F, Colletti G. Mini-retromandibular approach to condylar fractures. Journal of Cranio-Maxillofacial Surgery. 2008;36(7):378-83. | Title |
|  | Biglioli F, Colletti G. Transmasseter Approach to Condylar Fractures by Mini-Retromandibular Access. Journal of Oral and Maxillofacial Surgery. 2009;67(11):2418-24. | Title |
|  | Bikey D, Agur AMR, Fattah AY. Extra- and intramuscular innervation of the masseter: Implications for facial reanimation. Journal of Plastic, Reconstructive and Aesthetic Surgery. 2023;85:508-14. | Title |
|  | Bikey D, Davies J, Ebrahimi E, Hannam A, Holmes H, Liebgott B, Agur A. A 3D modeling study of the intramuscular course of the masseteric nerve. Journal of Oral and Maxillofacial Surgery. 2015;73(9):e72. | Title |
|  | Binello PB, Bandelloni R, Labanca M, Buffoli B, Rezzani R, Rodella LF. Osteonecrosis and the jaws and bevacizumab therapy: A case report. International Journal of Immunopathology and Pharmacology. 2012;25(3):789-91. | Title |
|  | Bins A, Koolstra J, Baart J, Forouzanfar T, Van Loon J. Intermaxillary fixation screws for conservative condylar fracture treatment: A torsion strength comparison and screw pattern recommendation. International Journal of Oral and Maxillofacial Surgery. 2017;46:256-7. | Title |
|  | Biondi K, Lorusso P, Fastuca R, Mangano A, Zecca PA, Bosco M, et al. Evaluation of masseter muscle in different vertical skeletal patterns in growing patients. Eur J Paediatr Dent. 2016;17(1):47-52. | Included |
|  | Bishop TM, Glass EN, de Lahunta A, Shelton GD. Imaging diagnosis-masticatory muscle myositis in a young dog. Veterinary Radiology & Ultrasound. 2008;49(3):270-2. | Title |
|  | Blazevich AJ. Effects of physical training and detraining, immobilisation, growth and aging on human fascicle geometry. Sports Medicine. 2006;36(12):1003-17. | Title |
|  | Blocquel H. Euclid and Pythagorus in the 21st century. A proposal on various harmonious craniofacial and occlusal constants. Bulletin du Groupement international pour la recherche scientifique en stomatologie & odontologie. 2001;43(1):26-33. | Title |
|  | Blocquel H, Laude M, Justin J, Thilloy G. Pterygoclival area: the structure of stable superposition during growth. Bulletin du Groupement international pour la recherche scientifique en stomatologie & odontologie. 1983;26(4):265-84. | Title |
|  | Boahene K. Skeletal Contouring Techniques in the Ethnic Patient. Facial Plastic Surgery Clinics of North America. 2022;30(4):499-506. | Title |
|  | Boamah MO, Brakohiapa EKK, Choi S, Blankson PK, Parkins GE. A rare case of multiple minor salivary gland sialoliths in the masseteric region. Clinical Case Reports. 2021;9(5). | Title |
|  | Boccaccio A, Pappalettere C, Kelly DJ. The influence of expansion rates on mandibular distraction osteogenesis: a computational analysis. Ann Biomed Eng. 2007;35(11):1940-60. | Title |
|  | Bocchialini G, Castellani A, Bozzola A, Rossi A. Soft-Tissue Chondroma in the Preauricular Region: An Unusual Presentation. Craniomaxillofacial Trauma and Reconstruction. 2018;11(1):49-53. | Title |
|  | Bocchialini G, Castellani A, Negrini S, Rossi A. New Management in Bilateral Masseter Muscle Hypertrophy. Craniomaxillofacial Trauma and Reconstruction. 2017;10(4):325-8. | Title |
|  | Bocchialini G, Ferrari L, Rossini M, Bozzola A, Burlini D. Chronic nonbacterial osteomyelitis involving the mandible: A case report. International Journal of Surgery Case Reports. 2017;37:149-53. | Title |
|  | Bocquet E, Moreau A, Decrucq E, Djerbi N, Danguy-Derot C, Danguy M. [Supraclusion origins]. L' Orthodontie française. 2010;81(3):227-34. | Title |
|  | Bodner L, Miller VJ. Temporomandibular joint dysfunction in children: Evaluation of treatment. International Journal of Pediatric Otorhinolaryngology. 1998;44(2):133-7. | Title |
|  | Bogucki ZA, Kownacka M. Clinical aspects of the use of botulinum toxin type a in the treatment of dysfunction of the masticatory system. Advances in Clinical and Experimental Medicine. 2016;25(3):569-73. | Title |
|  | Boléo-Tomé J. Some ideas on relapse after remodeling of prognathism: Aesthetic and functional results. Aesthetic Plastic Surgery. 1998;22(3):185-9. | Title |
|  | Bolonkin VP. Rationale for the choice of surgical strategy in patients with prognathism. Stomatologiya. 1990;69(3):46-8. | Title |
|  | Bolt KJ, Orchardson R. Relationship between mouth-opening force and facial skeletal dimensions in human females. Archives of Oral Biology. 1986;31(12):789-93. | Full text  Other outcomes |
|  | Bommarito S, Zanato LE, Vieira MM, Angelieri F. Aglossia: Case report. International Archives of Otorhinolaryngology. 2016;20(1):87-92. | Title |
|  | Bong KC, Kim CH, Baek SH. Skeletal sagittal and vertical facial types and electromyographic activity of the masticatory muscle. Angle Orthodontist. 2007;77(3):463-70. | Title |
|  | Boom HP, van Spronsen PH, van Ginkel FC, van Schijndel RA, Castelijns JA, Tuinzing DB. A comparison of human jaw muscle cross-sectional area and volume in long- and short-face subjects, using MRI. Arch Oral Biol. 2008;53(3):273-81. | Included |
|  | Boon AJ, Wijntjes J, O'Brien TG, Sorenson EJ, Cazares Gonzalez ML, van Alfen N. Diagnostic accuracy of gray scale muscle ultrasound screening for pediatric neuromuscular disease. Muscle and Nerve. 2021;64(1):50-8. | Title |
|  | Borda MG, Hassan EB, Weon JH, Wakabayashi H, Tovar-Rios DA, Oppedal K, et al. Muscle Volume and Intramuscular Fat of the Tongue Evaluated With MRI Predict Malnutrition in People Living With Dementia: A 5-Year Follow-up Study. Journals of Gerontology Series a-Biological Sciences and Medical Sciences. 2022;77(2):228-34. | Title |
|  | Boricić I, Stojsić Z, Mikić A, Brasanac D, Tomanović N, Bacetić D. Intramuscular hemangioma of the retropharyngeal space. Vojnosanit Pregl. 2007;64(7):485-8. | Title |
|  | Bosak A, Raschke R. An atypical presentation of neuroinvasive west nile virus. American Journal of Respiratory and Critical Care Medicine. 2015;191. | Title |
|  | Boscato N, Exposto FG, Costa YM, Svensson P. Effect of standardized training in combination with masseter sensitization on corticomotor excitability in bruxer and control individuals: a proof of concept study. Scientific Reports. 2022;12(1). | Title |
|  | Botteron S, Verdebout CM, Jeannet PY, Kiliaridis S. Orofacial dysfunction in Duchenne muscular dystrophy. Archives of Oral Biology. 2009;54(1):26-31. | Title |
|  | Botzenhart UU, Keil C, Tsagkari E, Zeidler-Rentzsch I, Gredes T, Gedrange T. Influence of botulinum toxin A on craniofacial morphology after injection into the right masseter muscle of dystrophin deficient (mdx-) mice. Ann Anat. 2021;236:151715. | Title |
|  | Boughner JC. Implications of Vertebrate Craniodental Evo-Devo for Human Oral Health. J Exp Zool B Mol Dev Evol. 2017;328(4):321-33. | Title |
|  | Bourdiol P, Soulier-Peigue D, Lachaze P, Nicolas E, Woda A, Hennequin M. Only severe malocclusion correlates with mastication deficiency. Archives of Oral Biology. 2017;75:14-20. | Title |
|  | Bourjat P. Useful imaging in maxillofacial surgery. Part II: practical applications. Revue de Stomatologie et de Chirurgie Maxillo-Faciale. 2007;108(1):31-45. | Title |
|  | Boyd SB, Gonyea WJ, Finn RA, Woodard CE, Bell WH. Histochemical study of the masseter muscle in patients with vertical maxillary excess. Journal of Oral and Maxillofacial Surgery. 1984;42(2):75-83. | Title |
|  | Boyd SB, Gonyea WJ, Legan HL, Bell WH. Masseter muscle adaptation following surgical correction of vertical maxillary excess. Journal of Oral and Maxillofacial Surgery. 1989;47(9):953-62. | Title |
|  | Bozhkova T. Comparison of two generations of systems for digital occlusion examination. Open Access Macedonian Journal of Medical Sciences. 2021;9(D):133-7. | Title |
|  | Brachetta-Aporta N, Gonzalez PN, Bernal V. Variation in facial bone growth remodeling in prehistoric populations from southern South America. American Journal of Physical Anthropology. 2019;169(3):422-34. | Title |
|  | Brady M. Mechanical or biological cues?  Parameters to engineer a 3D human masseter muscle. 2008. | Title |
|  | Brady MA, Lewis MP, Mudera V. Synergy between myogenic and non-myogenic cells in a 3D tissue-engineered craniofacial skeletal muscle construct. Journal of Tissue Engineering and Regenerative Medicine. 2008;2(7):408-17. | Title |
|  | Brassard C, Merlin M, Guintard C, Monchâtre-Leroy E, Barrat J, Callou C, et al. Interrelations Between the Cranium, the Mandible and Muscle Architecture in Modern Domestic Dogs. Evolutionary Biology. 2020;47(4):308-24. | Title |
|  | Brassard C, Merlin M, Monchâtre-Leroy E, Guintard C, Barrat J, Callou C, et al. How Does Masticatory Muscle Architecture Covary with Mandibular Shape in Domestic Dogs? Evolutionary Biology. 2020;47(2):133-51. | Title |
|  | Braun S, Hnat WP, Freudenthaler JW, Marcotte MR, Hönigle K, Johnson BE. A study of maximum bite force during growth and development. Angle Orthodontist. 1996;66(4):261-4. | Title |
|  | Bredell MG. Condylar, segmental or marginal resection and reconstruction, have the options changed? Oral Oncology. 2011;47:S25. | Title |
|  | Brennick MJ, Delikatny J, Pack AI, Pickup S, Shinde S, Zhu JX, et al. Tongue Fat Infiltration in Obese Versus Lean Zucker Rats. Sleep. 2014;37(6):1095-U198. | Title |
|  | Bresin A, Bagge U, Kiliaridis S. Adaptation of normal and hypofunctional masseter muscle after bite-raising in growing rats. European Journal of Oral Sciences. 2000;108(6):493-503. | Title |
|  | Bresin A, Kiliaridis S. Dento-skeletal adaptation after bite-raising in growing rats with different masticatory muscle capacities. European Journal of Orthodontics. 2002;24(3):223-37. | Title |
|  | Breuel W, Krause M, Schneider M, Harzer W. Genetic stretching factors in masseter muscle after orthognathic surgery. British Journal of Oral and Maxillofacial Surgery. 2013;51(6):530-5. | Title |
|  | Briesemeister M, Schmidt KC, Ries LGK. Changes in masticatory muscle activity in children with cerebral palsy. Journal of Electromyography and Kinesiology. 2013;23(1):260-6. | Title |
|  | Brinkworth RSA, Türker KS, Savundra AW. Response of human jaw muscles to axial stimulation of the incisor. Journal of Physiology. 2003;547(1):233-45. | Title |
|  | Brisset M, Ben Yaou R, Carlier RY, Chanut A, Nicolas G, Romero NB, et al. X-linked Emery–Dreifuss muscular dystrophy manifesting with adult onset axial weakness, camptocormia, and minimal joint contractures. Neuromuscular Disorders. 2019;29(9):678-83. | Title |
|  | Brontoladi S, Sembronio S, Tel A, Lazzarotto A, Robiony M. A case report of chondrocalcinosis of the temporomandibular joint: Surgical management and literature review. Oral and Maxillofacial Surgery Cases. 2020;6(3). | Title |
|  | Brown G, Ludwig D, Lazarus M. An unusual case of dermatomyositis with antismall ubiquitin-like modifier activating enzyme (sae) antibodies present. Rheumatology Advances in Practice. 2019;3:i19-i20. | Title |
|  | Brucoli M, Arcuri F, Borello G, Benech A. Surgical technique of the transoral approach to remove a lipoma of the buccal fat pad. J Craniofac Surg. 2011;22(6):2415-8. | Title |
|  | Brundage EA, Biesiadecki BJ, Reiser PJ. Nucleotide and protein sequences for dog masticatory tropomyosin identify a novel <i>Tpm4</i> gene product. Journal of Muscle Research and Cell Motility. 2015;36(4-5):339-47. | Title |
|  | Brustowicz KA, Padwa BL. Malocclusion in children caused by temporomandibular joint effusion. International Journal of Oral and Maxillofacial Surgery. 2013;42(8):1034-6. | Title |
|  | Bu B, Zhou DB, Xu BN, Yu XG, Zhang YZ, Wei SB. Spontaneous and evoked facial muscle electromyogram in monitoring nervous function in acoustic neuroma surgery and nervous prognosis: A character analysis in 120 cases. Chinese Journal of Clinical Rehabilitation. 2006;10(34):162-5. | Title |
|  | Bucci R, Lobbezoo F, Michelotti A, Koutris M. Two repetitive bouts of intense eccentric-concentric jaw exercises reduce experimental muscle pain in healthy subjects. Journal of Oral Rehabilitation. 2018;45(8):575-80. | Title |
|  | Buduru S, Kui A, Talmaceanu D, Baru O, Bolog N, Popa RS, et al. Acute dental malocclusion associated with lateral pterygoid muscle partial tear: Case Report and literature review. Cranio-the Journal of Craniomandibular & Sleep Practice. 2021. | Title |
|  | Buduru S, Prigoana A, Valean A, Manziuc M, Iacob S, Bacali C, et al. CORRELATIONS BETWEEN DENTAL OCCLUSION AND UPPER CERVICAL SPINE. Romanian Journal of Oral Rehabilitation. 2021;13(3):51-8. | Title |
|  | Bulut DG, Avci F, Özcan G. Ultrasonographic evaluation of jaw elevator muscles in young adults with bruxism and with and without attrition-type tooth wear: A pilot study. Cranio-the Journal of Craniomandibular & Sleep Practice. 2020;38(4):248-55. | Title |
|  | Bundgaard M, Bjerregaard J, Melsen B, Terp S. An electromyographical study of the effect of a mandibular lipbumper. European Journal of Orthodontics. 1983;5(2):149-56. | Title |
|  | Bunpu P, Changsiripun C. Assessment of masticatory performance in patients undergoing orthognathic surgery: A systematic review and meta-analysis. Journal of Oral Rehabilitation. 2023;50(7):596-616. | Title |
|  | Burdi AR, Spyropoulos MN. Prenatal growth patterns of the human mandible and masseter muscle complex. Am J Orthod. 1978;74(4):380-7. | Title |
|  | Burstone CJ. Lip posture and its significance in treatment planning. American Journal of Orthodontics. 1967;53(4):262-84. | Title |
|  | Busato A, Balconi G, Vismara V, Bertelè L, Tonti G, Pedrizzetti G. STRAIN ANALYSIS OF MASSETER MUSCLE BY ULTRASOUND. Journal of Biological Regulators and Homeostatic Agents. 2015;29(3):74-81. | Abstract |
|  | Buschang PH, Throckmorton GS, Travers KH, Hayasaki H. Incisor and mandibular condylar movements of young adult females during maximum protrusion and lateratrusion of the jaw. Archives of Oral Biology. 2001;46(1):39-48. | Title |
|  | Byeon KS, Lee YJ, Yoon YJ, Hong SM, Park YH, Choi DJ, Park JW. Postoperative stability after setback of sagittal split ramus osteotomy: A comparison of three techniques. Journal of Oral and Maxillofacial Surgery. 2013;71(3):597-609. | Title |
|  | Byron C, Reed D, Iriarte-Diaz J, Wang Q, Strait D, Laird MF, Ross CF. Sagittal suture strain in capuchin monkeys (Sapajus and Cebus) during feeding. American Journal of Biological Anthropology. 2023;180(4):633-54. | Title |
|  | Byron CD, Borke J, Yu J, Pashley D, Wingard CJ, Hamrick M. Effects of increased muscle mass on mouse sagittal suture morphology and mechanics. Anatomical Record Part a-Discoveries in Molecular Cellular and Evolutionary Biology. 2004;279A(1):676-84. | Title |
|  | Byron CD, Hamrick MW, Wingard CJ. Alterations of temporalis muscle contractile force and histological content from the myostatin and <i>Mdx</i> deficient mouse. Archives of Oral Biology. 2006;51(5):396-405. | Title |
|  | Cabezas-Camarero S, Alonso-Ovies A, Merino-Menéndez S, Cabrera-Martín MN, Plaza-Hernández JC, Pérez-Segura P. Major pathological response and durable locoregional control after neoadjuvant pembrolizumab-carboplatin-paclitaxel in head and neck cancer. Oral Oncology. 2021;123. | Title |
|  | Cacho A, Tordera C, Colmenero C. Use of Transcutaneous Electrical Nerve Stimulation (TENS) for the Recovery of Oral Function after Orthognathic Surgery. Journal of clinical medicine. 2022;11(12). | Title |
|  | Çaglayan F, Ocak A, Sümbüllü MA. Examination of facial supramandibular lymph nodes in dental patients by ultrasonography. Nobel Medicus. 2019;15(2):35-9. | Title |
|  | Calis AS, Colakoglu Z, Gunbay S. The use of botulinum toxin-a in the treatment of muscular temporomandibular joint disorders. Journal of Stomatology Oral and Maxillofacial Surgery. 2019;120(4):322-5. | Title |
|  | Cambala A. The Orofacial Musculoskeletal Structures and Function in Cerebral Palsy - A Scoping Review [Dissertation/Thesis]2022. | Title |
|  | Cambala A, Nguyen T, Zimmermann E, Morris M. The Effects of Cerebral Palsy on Orofacial Musculoskeletal Structure and Function -A Scoping Review. Developmental Medicine and Child Neurology. 2022;64:92-3. | Title |
|  | Canellas JVS, Araujo MM, Arce JPA. The use of anatomical models for learning anesthesia techniques in oral surgery. Indian Journal of Dental Research. 2013;24(3):326-30. | Title |
|  | Capaccio P, Luca N, Sigismund PE, Pignataro L. Recurrent inflammation of accessory parotid tissue associated with unilateral parotid gland aplasia: Diagnostic and therapeutic implications. European Archives of Oto-Rhino-Laryngology. 2012;269(5):1551-4. | Title |
|  | Capaccioli L, Antonini A, Franchi L, Tollaro I, Zecchi Orlandini S, Stecco A, Villari N. Correlation between the US pattern of perioral and masticatory muscles and dento-skeletal characteristics. Radiologia Medica. 1998;95(6):567-72. | Included |
|  | Capozzi L, Köle H, Rossi G, Perko M, Miclavez N. Orthognathodontic surgery. Rivista italiana di stomatologia. 1968;23(8):1075-332. | Title |
|  | Capra NF, Hisley CK, Masri RM. The influence of pain on masseter spindle afferent discharge. Archives of Oral Biology. 2007;52(4):387-90. | Title |
|  | Cárdenas H, Ogalde A. Relationship between Occlusion and EMG Activity of the Masseter Muscles during Clenching at Maximal Intercuspal Position: A Comparative Study between Prognathics and Controls. Cranio - Journal of Craniomandibular and Sleep Practice. 2002;20(2):99-104. | Full text  Other outcomes |
|  | Carels C, Steenberghe Dv. Changes in neuromuscular reflexes in the masseter muscles during functional jaw orthopedic treatment in children. American Journal of Orthodontics and Dentofacial Orthopedics. 1986;90(5):410-9. | Title |
|  | Carels C, Van Steenberghe D. Posterior periodontal loading plays a key role in the suppression of a short latency excitatory reflex in the masseter muscle in children being treated with the bionator. European Journal of Orthodontics. 1986;8(2):84-90. | Title |
|  | Carlier RY. Advances in imaging for the diagnosis and disease monitoring of Pompe disease. BMC Musculoskeletal Disorders. 2013;14(1). | Title |
|  | Carlier RY, Quijano-Roy S. Myoimaging in Congenital Myopathies. Seminars in Pediatric Neurology. 2019;29:30-43. | Title |
|  | Carlotti AE, George R. A diagnostic adjunct in treatment planning for the dentofacial deformity patient. American Journal of Orthodontics and Dentofacial Orthopedics. 1987;91(6):451-62. | Title |
|  | Carra MC. Sleep-Related Bruxism. Current Sleep Medicine Reports. 2018;4(1):28-38. | Title |
|  | Carrier DR, Morgan MH. Protective buttressing of the hominin face. Biological Reviews. 2015;90(1):330-46. | Title |
|  | Carrière M, Prudentos JB, Lecigne A, Laran A, Nguyen CT, Destruhaut F, Naveau A. Digital optimization of teeth setup in an edentulous patient with partial glossectomy: A case report. Journal of Prosthodontics. 2023;32(6):461-8. | Title |
|  | Carvalho DR, Farage L, Martins B, Speck-Martins CE. Craniofacial findings in fibrodysplasia ossificans progressiva: computerized tomography evaluation. Oral Surgery Oral Medicine Oral Pathology Oral Radiology and Endodontology. 2011;111(4):499-502. | Title |
|  | Carvalho DR, Farage L, Speck-Martins CE. The signature of craniofacial deformation in fibrodysplasia ossificans progressiva. American Journal of Medical Genetics, Part A. 2012;158 A(11):2977-8. | Title |
|  | Casas EBL, Ferreira PC, Cimini CA, Toledo EM, Barra L, Cruz M. Comparative 3D finite element stress analysis of straight and angled wedge-shaped implant designs. International Journal of Oral & Maxillofacial Implants. 2008;23(2):215-25. | Title |
|  | Cascino F, Chisci G, Latini L, Gabriele G. Masseteric giant capillary haemangioma: a bulky mass treated with scarless and minimally invasive surgery. BMJ Case Reports. 2022;15(12). | Title |
|  | Cassetta M, Pranno N, Pompa V, Barchetti F, Pompa G. High resolution 3-T MR imaging in the evaluation of the trigeminal nerve course. European Review for Medical and Pharmacological Sciences. 2014;18(2):257-64. | Title |
|  | Cassoni A, Catalano C, Di Giorgio D, Raponi I, Di Brino M, Perotti S, Valentini V. Masseter-facial neurorrhaphy for facial palsy reanimation: What happens after masseter denervation? Histomorphometric and stomatognathic functional analysis. Journal of Cranio-Maxillofacial Surgery. 2020;48(7):680-4. | Title |
|  | Cassoni A, Romano A, Terenzi V, Bartoli D, Buonaccorsi S, Valentini V. Intramasseterin-infiltrating angiolipoma: A challenging diagnosis. Journal of Craniofacial Surgery. 2012;23(4):e290-e2. | Title |
|  | Casteleyn C, Wydooghe E, Bakker J. Osteology of the Hamadryas Baboon (Papio hamadryas). Animals. 2023;13(19). | Title |
|  | Castelo PM, Bonjardim LR, Pereira LJ, Gavião MB. Facial dimensions, bite force and masticatory muscle thickness in preschool children with functional posterior crossbite. Braz Oral Res. 2008;22(1):48-54. | Full text  Cross-bites |
|  | Castelo PM, Duarte Gavião MB, Pereira LJ, Bonjardim LR. Evaluation of changes in muscle thickness, bite force and facial asymmetry during early treatment of functional posterior crossbite. Journal of Clinical Pediatric Dentistry. 2010;34(4):369-74. | Full text  Cross-bites |
|  | Castelo PM, Gavião MB, Pereira LJ, Bonjardim LR. Masticatory muscle thickness, bite force, and occlusal contacts in young children with unilateral posterior crossbite. Eur J Orthod. 2007;29(2):149-56. | Full text  Cross-bites |
|  | Castelo PM, Gavião MBD, Pereira LJ, Bonjardim LR. Avaliação ultra-sonográfica dos músculos mastigatórios e dimensões faciais em crianças com oclusão normale mordida cruzada posterior unilateral. Revista CEFAC. 2007;9(1):61-71. | Full text  Cross-bites |
|  | Castelo PM, Gaviao MBD, Pereira LJ, Bonjardim LR. Maximal bite force, facial morphology and sucking habits in young children with functional posterior crossbite. Journal of Applied Oral Science. 2010;18(2):143-8. | Abstract |
|  | Castelo PM, Pereira LJ, Andrade AS, Marquezin MC, Gavião MB. Evaluation of facial asymmetry and masticatory muscle thickness in children with normal occlusion and functional posterior crossbite. Minerva stomatologica. 2010;59(7-8):423-30. | Full text  Cross-bites |
|  | Castelo PM, Pereira LJ, Bonjardim LR, Gavião MB. Changes in bite force, masticatory muscle thickness, and facial morphology between primary and mixed dentition in preschool children with normal occlusion. Ann Anat. 2010;192(1):23-6. | Full text  No skeletal pattens |
|  | Castro J, Likhterov I, Mehra S, Bassiri-Tehrani M, Scherl S, Clain J, et al. Approach to en bloc resection and reconstruction of primary masticator space malignancies. Laryngoscope. 2016;126(2):372-7. | Title |
|  | Castroflorio T, Falla D, Wang K, Svensson P, Farina D. Effect of experimental jaw-muscle pain on the spatial distribution of surface EMG activity of the human masseter muscle during tooth clenching. Journal of Oral Rehabilitation. 2012;39(2):81-92. | Title |
|  | Catanzariti JF, Debuse T, Duquesnoy B. Chronic neck pain and masticatory dysfunction. Joint Bone Spine. 2005;72(6):515-9. | Title |
|  | Catelani C, Valente A, Rossi A, Bertolai R. Broken anesthetic needle in the pterygomandibular space. Four case reports. Minerva Stomatologica. 2013;62(11-12):455-63. | Title |
|  | Cathcart J, Johnson RC, Hughes N, Patel M. Diagnostic difficulties in a patient with multiple sclerosis who presents with cranial nerve palsies: An unusual complication of dental work. BMJ Case Reports. 2020;13(10). | Title |
|  | Cattaneo PM, Dalstra M, Melsen B. The transfer of occlusal forces through the maxillary molars: A finite element study. American Journal of Orthodontics and Dentofacial Orthopedics. 2003;123(4):367-73. | Title |
|  | Cattaneo PM, Kofod T, Dalstra M, Melsen B. Using the finite element method to model the biomechanics of the asymmetric mandible before, during and after skeletal correction by distraction osteogenesis. Computer Methods in Biomechanics and Biomedical Engineering. 2005;8(3):157-65. | Title |
|  | Çebi AT. Ultrasonographic evaluation of masseter muscle thickness in patients with disk displacement with reduction. Oral Radiology. 2019;35(3):239-44. | Title |
|  | Cecílio FA, Regalo SCH, Palinkas M, Issa JPM, Siéssere S, Hallak JEC, et al. Ageing and surface EMG activity patterns of masticatory muscles. Journal of Oral Rehabilitation. 2010;37(4):248-55. | Title |
|  | Celakil D, Ozdemir F, Eraydin F, Celakil T. Effect of orthognathic surgery on masticatory performance and muscle activity in skeletal Class III patients. Cranio - Journal of Craniomandibular Practice. 2018;36(3):174-80. | Title |
|  | Celebi N, Rohner EC, Gateno J, Noble PC, Ismaily SK, Teichgraeber JF, Xia JJ. Development of a mandibular motion simulator for total joint replacement. Journal of Oral and Maxillofacial Surgery. 2011;69(1):66-79. | Title |
|  | Celebic A, Valentic-Peruzovic M, Alajbeg IZ, Mehulic K, Knezovic-Zlataric D. Jaw elevator silent periods in complete denture wearers and dentate individuals. Journal of Electromyography and Kinesiology. 2008;18(6):947-54. | Title |
|  | Cenzato N, Marcolongo L, Stabilini A, Macrì L. Mandibular occlusal stability in patients undergoing orthognathic surgery. Dental Cadmos. 2023;91(3):218-23. | Title |
|  | Cesur E, Ozdiler O, Koklu A, Orhan K, Seki U. Effects of wear time differences of removable functional appliances in class II patients: prospective MRI study of TMJ and masticatory muscle changes. Oral radiology. 2020;36(1):47‐59. | Title |
|  | Cezairli B, Torul D, Kahveci K. The association between bruxism and mandibular morphology: A cross-sectional study. Journal of Oral Health and Oral Epidemiology. 2022;11(1). | Title |
|  | Chabokdast A. Multibody dynamics modelling of the masticatory system of the house mouse (mus musculus). 2015. | Title |
|  | Chai G, Zhang Y, Zhu M, Ma XF, Yu ZY, Mu XZ, Qi ZL. Evaluation of Dynamic Morphologic Changes in the Masseter Muscle in Patients Undergoing Mandibular Angle Sagittal Split Osteotomy <i>A Report of 130 Cases</i>. Archives of Facial Plastic Surgery. 2011;13(5):301-4. | Title |
|  | Chakfa AM, Mehta NR, Forgione AG, Al-Badawi EA, Lobo SL, Zawawi KH. The Effect of Stepwise Increases in Vertical Dimension of Occlusion on Isometric Strength of Cervical Flexors and Deltoid Muscles in Nonsymptomatic Females. Cranio - Journal of Craniomandibular and Sleep Practice. 2002;20(4):264-73. | Title |
|  | Chan HJ, Woods M, Stella D. Mandibular muscle morphology in children with different vertical facial patterns: A 3-dimensional computed tomography study. Am J Orthod Dentofacial Orthop. 2008;133(1):10.e1-3. | Included |
|  | Chandran R, Precheur H. Measurement of the isometric mouth opening forces in an ASA I, 20-40-year-old patient population. Journal of Oral and Maxillofacial Surgery. 2012;70(9):e-103. | Title |
|  | Chang CH, Mun GH, Lim SY, Hyon WS, Bang SI, Oh KS. Cavernous vascular tumor of the accessory parotid gland. Journal of Craniofacial Surgery. 2007;18(6):1493-6. | Title |
|  | Chang SW, Tsai YH, Hsu CM, Huang EI, Chang GH, Tsai MS, Tsai YT. Masticatory muscle index for indicating skeletal muscle mass in patients with head and neck cancer. PLoS ONE. 2021;16(5 May 2021). | Title |
|  | Chang Y, Cantelmi D, Wisco JJ, Fattah A, Hannam AG, Agur AM. Evidence for the functional compartmentalization of the temporalis muscle: A 3-dimensional study of innervation. Journal of Oral and Maxillofacial Surgery. 2013;71(7):1170-7. | Title |
|  | Chang YH, Chan MY, Hsu JT, Hsiao HY, Su KC. Biomechanical analysis of the forces exerted during different occlusion conditions following bilateral sagittal split osteotomy treatment for mandibular deficiency. Applied Bionics and Biomechanics. 2019;2019. | Title |
|  | Charalampidou M, Kjellberg H, Georgiakaki I, Kiliaridis S. Masseter muscle thickness and mechanical advantage in relation to vertical craniofacial morphology in children. Acta Odontologica Scandinavica. 2008;66(1):23-30. | Included |
|  | Chaudhry A, Sidhu MS, Chaudhary G, Grover S, Chaudhry N, Kaushik A. Evaluation of stress changes in the mandible with a fixed functional appliance: A finite element study. American Journal of Orthodontics and Dentofacial Orthopedics. 2015;147(2):226-34. | Title |
|  | Chauhan M, Punga T, Punga AR. Muscle-specific regulation of the mTOR signaling pathway in MuSK antibody seropositive (MuSK plus ) experimental autoimmune Myasthenia gravis (EAMG). Neuroscience Research. 2013;77(1-2):102-9. | Title |
|  | Che X, Luo S, Li Y. [A study of ultrasound images under 3 different functional mandibular positions in young females]. Hua Xi Kou Qiang Yi Xue Za Zhi. 2002;20(3):200-2. | Abstract |
|  | Che XX. Ultrasound image of maseter muscle in young woman and its relation to facial morphology. 2001. | Title |
|  | Chen CF, Chen CM, Chen HS, Huang WC, Chen YC, Chang HC, et al. The Use of Customized Three-Dimensionally Printed Mandible Prostheses with a Pressure-Reducing Device: A Finite Element Analysis in Different Chewing Positions, Biomechanical Testing, and In Vivo Animal Study Using Lanyu Pigs. BioMed Research International. 2022;2022. | Title |
|  | Chen J, Zhang R, Liang Y, Ma Y, Song S, Jiang C. Deviation Analyses of Computer-Assisted, Template-Guided Mandibular Reconstruction With Combined Osteotomy and Reconstruction Pre-Shaped Plate Position Technology: A Comparative Study. Frontiers in Oncology. 2021;11. | Title |
|  | Chen L, Pröschel PA, Morneburg TR. Influence of bite force on jaw muscle activity ratios in subject-controlled unilateral isometric biting. Journal of Electromyography and Kinesiology. 2010;20(5):961-6. | Title |
|  | Chen M, Yang C, He D, Zhang S, Jiang B. Soft tissue reduction during open treatment of intracapsular condylar fracture of the temporomandibular joint: Our institution's experience. Journal of Oral and Maxillofacial Surgery. 2010;68(9):2189-95. | Title |
|  | Chen M, Yang C, Qiu Y, He D, Huang D, Wei W. Superior half of the sternoclavicular joint pedicled with the sternocleidomastoid muscle for reconstruction of the temporomandibular joint: A preliminary study with a simplified technique and expanded indications. International Journal of Oral and Maxillofacial Surgery. 2015;44(6):685-91. | Title |
|  | Chen MJ, Yang C, Zhang SY, Cai XY. Use of coblation in arthroscopic surgery of the temporomandibular joint. Journal of Oral and Maxillofacial Surgery. 2010;68(9):2085-91. | Title |
|  | Chen P, Yan C, Xie T, Li C, Liu T, Yang Q, et al. Endoscopic far-lateral supracerebellar infratentorial approach for resection of dumbbell-shaped trigeminal schwannoma: surgical techniques and preliminary results. Acta Neurochirurgica. 2023;165(10):2913-21. | Title |
|  | Chen T, Liu Z, Xue C, Tian W, Bai D, Chen YP. Association of Dysplastic Coronoid Process with Long-Face Morphology. Journal of Dental Research. 2020;99(3):339-48. | Title |
|  | Chen TC, Cheng DH, Hsu ML, Lei YP. Application of masticatory control in dental treatment for elderly individuals. Journal of the Chinese Medical Association. 2021;84(2):125-8. | Title |
|  | Chen YJ, Yao CC, Chang ZC, Lai HH, Hsu LF, Hsu TH, Kok SH. Occlusal function and electromyographic activity of masticatory muscles in skeletal Class III patients with different patterns of mandibular asymmetry. J Oral Rehabil. 2023;50(4):276-85. | Abstract |
|  | Chen YT, Yu CC, Lin YC, Chan SH, Lin YY, Chen NC, Lin WC. Brain CT can predict low lean mass in the elderly with cognitive impairment: a community-dwelling study. BMC Geriatr. 2022;22(1):3. | Title |
|  | Chen Z, Liu G, Geng Y, Wu H. Iodine-125 brachytherapy for the treatment of central mucoepidermoid carcinoma of the jaw in a pre-teen. International Journal of Oral and Maxillofacial Surgery. 2022;51(10):1273-8. | Title |
|  | Chen ZX, Chen ZQ, Zhao N, Shen G. An Animal Model for Inducing Deviation of the Mandible. Journal of Oral and Maxillofacial Surgery. 2015;73(11):2207-18. | Title |
|  | Cheynet F, Gola R, Chossegros C, Orthlieb JD, Giraudeau A, Falanga HJ. [The contribution of standard radiographs in the evaluation of masticatory system dysfunction]. Rev Stomatol Chir Maxillofac. 1998;99(2):88-102. | Title |
|  | Chèze L, Navailles B. Impact on temporomandibular joint of two mandibular advancement device designs. ITBM-RBM. 2006;27(5-6):233-7. | Title |
|  | Chichareon V, Arpornmaeklong P, Donsakul N. Fibrodysplasia ossificans progressiva and associated osteochondroma of the coronoid process in a child. Plastic and Reconstructive Surgery. 1999;103(4):1238-43. | Title |
|  | Chikui T, Shiraishi T, Ichihara T, Kawazu T, Hatakenaka M, Kami Y, et al. Effect of clenching on T<sub>2</sub> and diffusion parameters of the masseter muscle. Acta Radiologica. 2010;51(1):58-63. | Title |
|  | Chikui T, Shiraishi T, Tokumori K, Inatomi D, Hatakenaka M, Yuasa K, Yoshiura K. Assessment of the sequential change of the masseter muscle by clenching: A quantitative analysis of T<sub>1</sub>, T<sub>2</sub>, and the signal intensity of the balanced steady-state free precession. Acta Radiologica. 2010;51(6):669-78. | Title |
|  | Chin SY, Berahim NB, Adnan KB, Ramasamy SN. Delayed Management of Unrecognized Bilateral Temporomandibular Joint Dislocation: A Case Report. Craniomaxillofacial Trauma and Reconstruction. 2018;11(2):145-9. | Title |
|  | Chintakanon K, Türker KS, Sampson W, Wilkinson T, Townsend G. Effects of Twin-block therapy on protrusive muscle functions. American Journal of Orthodontics and Dentofacial Orthopedics. 2000;118(4):392-6. | Title |
|  | Chintakanon K, Türker KS, Sampson WJ, Townsend GC, Wilkinson TM. A method for protrusive mandibular force measurement in children. Archives of Oral Biology. 2000;45(2):113-21. | Title |
|  | Cho ES, Cho HM, Nam W, Kim HS. Multiple Oral Deep Penetrating Nevi Extending to the Masseter and Buccal Fat Pad. Journal of Oral and Maxillofacial Surgery. 2017;75(12):2579-92. | Title |
|  | Cho YM, Kim SG, Choi DS, Jang I, Cha BK. Botulinum Toxin Injection to Treat Masticatory Movement Disorder Corrected Mandibular Asymmetry in a Growing Patient. J Craniofac Surg. 2019;30(6):1850-4. | Title |
|  | Choi BH. Comparison of computed tomography imaging before and after functional treatment of bilateral condylar fractures in adults. International Journal of Oral and Maxillofacial Surgery. 1996;25(1):30-3. | Title |
|  | Choi J, Baik JE, Kranich E, Conboy LA. Case series: potential of facial and orthodontic skull correction through oral cosmetic acupuncture. Integrative Medicine Research. 2020;9. | Title |
|  | Choi JW, Kim HJ, Moon JW, Kang SH, Tak HJ, Lee SH. Compensatory dentoalveolar supraeruption and occlusal plane cant after botulinum-induced hypotrophy of masticatory closing muscles in juvenile rats. Archives of Oral Biology. 2019;101:34-42. | Title |
|  | Choi YJ, Lim H, Chung CJ, Park KH, Kim KH. Two-year follow-up of changes in bite force and occlusal contact area after intraoral vertical ramus osteotomy with and without Le Fort I osteotomy. International Journal of Oral and Maxillofacial Surgery. 2014;43(6):742-7. | Title |
|  | Chong J, Som PM, Silvers AR, Dalton JF. Extranodal non-Hodgkin lymphoma involving the muscles of mastication. AJNR Am J Neuroradiol. 1998;19(10):1849-51. | Title |
|  | Christensen LV. An electromyographic and cephalometric study on facial pains and facial morphology in children. Journal of Oral Rehabilitation. 1981;8(3):267-77. | Full text  Other outcomes |
|  | Christensen LV, McKay DC. Kinematic and kinetic observations on ballistic depression and elevation of the human mandible. Journal of Oral Rehabilitation. 2000;27(6):494-507. | Title |
|  | Christensen LV, Rassouli NM. EXPERIMENTAL OCCLUSAL INTERFERENCES .2. MASSETERIC EMG RESPONSES TO INTERCUSPAL INTERFERENCE. Journal of Oral Rehabilitation. 1995;22(7):521-31. | Title |
|  | Chuang HY, Hwang JJ. Combination effects of sorafenib with ionizing radiation on orthotopic human oral-bearing mice model. Molecular Imaging and Biology. 2016;18(1):S1009-S10. | Title |
|  | Chugh VK, Sharma VP, Tandon P, Singh GP. Brodie bite with an extracted mandibular first molar in a young adult: A case report. American Journal of Orthodontics and Dentofacial Orthopedics. 2010;137(5):694-700. | Title |
|  | Chung K. ENPP1 and ESR1 Genotype Associate With Craniofacial Asymmetry and Severity of TMD [Dissertation/Thesis]2018. | Title |
|  | Chung K, Richards T, Nicot R, Vieira AR, Cruz CV, Raoul G, et al. ENPP1 and ESR1 genotypes associated with subclassifications of craniofacial asymmetry and severity of temporomandibular disorders. Am J Orthod Dentofacial Orthop. 2017;152(5):631-45. | Title |
|  | Chung MK, Ro JY. Peripheral glutamate receptor and transient receptor potential channel mechanisms of craniofacial muscle pain. Molecular Pain. 2020;16. | Title |
|  | Ciavarella D, Mastrovincenzo M, Sabatucci A, Parziale V, Chimenti C. Effect of the Enveloppe Linguale Nocturne on atypical swallowing: Surface electromyography and computerised postural test evaluation. European Journal of Paediatric Dentistry. 2010;11(1):141-5. | Title |
|  | Ciavarella D, Monsurrò A, Padricelli G, Battista G, Laino L, Perillo L. Unilateral posterior crossbite in adolescents: Surface electromyographic evaluation. European Journal of Paediatric Dentistry. 2012;13(1):25-8. | Title |
|  | Ciavarella D, Russo LL, Nichelini J, Mastrovincenzo M, Barbato E, Laurenziello M, et al. Treatment of hyperdivergent growth pattern and anterior open bite with posterior metallic bite planes. Minerva Stomatologica. 2017;66(6):267-74. | Title |
|  | Ciccone De Faria TDS, Hallak Regalo SC, Thomazinho A, Vitti M, De Felício CM. Masticatory muscle activity in children with a skeletal or dentoalveolar open bite. European Journal of Orthodontics. 2010;32(4):453-8. | Full text  Other outcomes |
|  | Ciftci V, Uzel A. Dento-skeletal effects of myofunctional appliance on patients with class II div 1 in mixed dentition stage: A cephalometric study. Pediatric Dental Journal. 2021;31(3):235-41. | Title |
|  | Cihangiroglu M, Akfirat M, Yildirim H. CT and MRI findings of ameloblastoma in two cases. Neuroradiology. 2002;44(5):434-7. | Title |
|  | Cioffi I, Gallo LM, Palla S, Erni S, Farella M. Macroscopic analysis of human masseter compartments assessed by magnetic resonance imaging. Cells Tissues Organs. 2012;195(5):465-72. | Full text  Other outcomes |
|  | Cirillo S, Regge D, Garagiola U, Tortarolo A, Iorio GC, Spahiu O, Piancino MG. Arthrogryposis multiplex congenita with maxillofacial involvement: a case report. Maxillofacial Plastic and Reconstructive Surgery. 2023;45(1). | Title |
|  | Clark GT, Adler RC. A critical evaluation of occlusal therapy: occlusal adjustment procedures. Journal of the American Dental Association (1939). 1985;110(5):743-50. | Title |
|  | Clement WA, Graham I, Ablett M, Rawlings D, Dempster JH. Intramuscular hemangioma of the posterior belly of the digastric muscle failing to highlight on magnetic resonance imaging. Annals of Otology, Rhinology and Laryngology. 2002;111(11):1050-3. | Title |
|  | Clemente MP, Mendes JG, Vardasca R, Ferreira AP, Amarante JM. Combined Acquisition Method of Image and Signal Technique (CAMIST) for assessment of temporomandibular disorders in performing arts medicine a pilot study. Medical Problems of Performing Artists. 2018;33(3):205-12. | Title |
|  | Coccaro PJ. Restitution of mandibular form after condylar injury in infancy (a 7-year study of a child). American Journal of Orthodontics. 1969;55(1):32-49. | Title |
|  | Coclici A, Hedeşiu M, Bran S, Băciuţ M, Dinu C, Rotaru H, Roman R. Early and long-term changes in the muscles of the mandible following orthognathic surgery. Clin Oral Investig. 2019;23(9):3437-44. | Title |
|  | Coclici A, Roman RA, Bran S, Crasnean E, Baciut M, Dinu C, Hedesiu M. Ultrasound dimensional changes of the anterior belly of the digastric muscle induced by orthognathic surgery and botulinum toxin A injection in Class II malocclusion. Oral Radiology. 2021;37(4):625-30. | Title |
|  | Coelho M, Marti MJ, Valls-Solé J, Pujol T, Tolosa E. Left hemibody myoclonus due to anomalous right vertebral artery. Movement Disorders. 2005;20(1):72-5. | Title |
|  | Collin J, French K, Davies R, Hughes C. Aneurysm of the facial artery. British Journal of Oral and Maxillofacial Surgery. 2015;53(10):e82-e3. | Title |
|  | Colombo JR, Dagher W, Wein RO. Benign proliferative myositis of the sternohyoid muscle: Review and case report. American Journal of Otolaryngology. 2015;36(1):87-9. | Title |
|  | Connachan R, Evans SE, Fagan MJ, Gröning F. Collagen fibre orientation in the human temporal fascia. Journal of Anatomy. 2018;232(6):1057. | Title |
|  | Connor SE, Chavda SV, West R. Recurrence of non-Hodgkin's lymphoma isolated to the right masticator and left psoas muscles. Eur Radiol. 2000;10(5):841-3. | Title |
|  | Constantaras ME, Charlier CJ. Maxillofacial Injuries and Diseases That Cause an Open Mouth in Cats. Journal of Veterinary Dentistry. 2014;31(3):168-76. | Title |
|  | Conte R, Forin Valvecchi F, Gracco AL, Bruno G, de Stefani A. Condylar dysfunctional remodeling and recortication: A case-control study. Minerva Stomatologica. 2019;68(2):74-83. | Title |
|  | Conway SMD. Re: Newton J.P. et al. masseteric hypertrophy? Preliminary report [4]. British Journal of Oral and Maxillofacial Surgery. 2000;38(5):573. | Title |
|  | Cook RW, Vazzana A, Sorrentino R, Benazzi S, Smith AL, Strait DS, Ledogar JA. Evaluating the craniofacial feeding biomechanics in Homo floresiensis using the finite element method. American Journal of Physical Anthropology. 2021;174(SUPPL 71):21-2. | Title |
|  | Cooper BC. The role of bioelectronic instrumentation in the documentation and management of temporomandibular disorders. Oral Surgery, Oral Medicine, Oral Pathology, Oral Radiology, and Endodontics. 1997;83(1):91-100. | Title |
|  | Cordray FE. Three-dimensional analysis of models articulated in the seated condylar position from a deprogrammed asymptomatic population: A prospective study. Part 1. American Journal of Orthodontics and Dentofacial Orthopedics. 2006;129(5):619-30. | Title |
|  | Corte GM, Hünigen H, Richardson KC, Niehues SM, Plendl J. Cephalometric studies of the mandible, its masticatory muscles and vasculature of growing Göttingen Minipigs-A comparative anatomical study to refine experimental mandibular surgery. PLoS One. 2019;14(4):e0215875. | Title |
|  | Coskun Akar G, Govsa F, Ozgur Z. Examination of the heads of the lateral pterygoid muscle on the temporomandibular joint. Journal of Craniofacial Surgery. 2009;20(1):219-23. | Title |
|  | Cossellu G, Farronato M, Biagi R, Assandri F, Farronato G. Idiopathic hypoplasia of the masseter muscle: A case report. Cranio-the Journal of Craniomandibular & Sleep Practice. 2017;35(3):192-6. | Title |
|  | Costa F, Cian R, Robiony M, Zerman N, Politi M. Unilateral Swelling of the Cheek. Journal of Oral and Maxillofacial Surgery. 2008;66(2):342-8. | Title |
|  | Costa YM, Exposto FG, Kothari M, Castrillon EE, Conti PCR, Bonjardim LR, Svensson P. Masseter corticomotor excitability is decreased after intramuscular administration of nerve growth factor. European Journal of Pain (United Kingdom). 2019;23(9):1619-30. | Title |
|  | Costa YM, Porporatti AL, Stuginski-Barbosa J, Cassano DS, Bonjardim LR, Conti PC. Coronoid process hyperplasia: an unusual cause of mandibular hypomobility. Braz Dent J. 2012;23(3):252-5. | Title |
|  | Coutand A, Fraudet R, Ovazza G, Maleysson G. Is the morphological appearance alone sufficient for a diagnosis to be made in the case of a child? Importance of supplementary examinations in dentofacial orthopedics. REVSTOMATOL. 1975;76(3):233-40. | Abstract |
|  | Crawford SR. The effect of sex, age and ethnicity on craniofacial bone mineral density. 2014. | Title |
|  | Crawley MB, Anand SM, Clain JB, Scherl S, Buchbinder D, Urken ML. Trismus release in a pediatric patient using a parascapular free flap reconstruction following desmoid tumor resection. Laryngoscope. 2013;123(6):1451-4. | Title |
|  | Cray J, Kneib J, Vecchione L, Byron C, Cooper GM, Losee JE, et al. Masticatory HyperMuscularity is not Related to Reduced Cranial Volume in Myostatin-Knockout Mice. Anatomical Record-Advances in Integrative Anatomy and Evolutionary Biology. 2011;294(7):1170-7. | Title |
|  | Cruz DZ, Rodrigues L, Luz JGD. Effects of detachment and repositioning of the medial pterygoid muscle on the growth of the maxilla and mandible of young rats. Acta Cirurgica Brasileira. 2009;24(2):93-7. | Title |
|  | Ctri. Reduction in oro-facial (muscular) pain following grinding the teeth causing inappropriate teeth contact using T-scan device to understant amount of masticatory forces. http://wwwwhoint/trialsearch/Trial2aspx?TrialID=CTRI/2021/05/033874. 2021. | Title |
|  | Ctri. THERAPEUTIC BOTULINUM TOXIN INJECTION IN MASSETER MUSCLE FOR DEEP BITE CORRECTION. https://trialsearchwhoint/Trial2aspx?TrialID=CTRI/2023/06/054024. 2023. | Title |
|  | Ctri. Effectiveness of Maitlandâ??s Temporomandibular joint mobilization in Temporomandibular Joint Disorders. https://trialsearchwhoint/Trial2aspx?TrialID=CTRI/2023/03/050246. 2023. | Title |
|  | Cullati F, Mapelli A, Beltramini G, Codari M, Pimenta Ferreira CL, Baj A, et al. Surface electromyography before and after orthognathic surgery and condylectomy in active laterognathia: a case report. Eur J Paediatr Dent. 2017;18(2):131-8. | Title |
|  | Cunha A, Nelson P, Marañón-Vásquez GA, Ramos AGD, Dantas B, Sebastiani AM, et al. Genetic variants in <i>ACTN3</i> and <i>MYO1H</i> are associated with sagittal and vertical craniofacial skeletal patterns. Archives of Oral Biology. 2019;97:85-90. | Title |
|  | Curry FRE, Taxt T, Rygh CB, Pavlin T, Bjonrstad R, Doskeland SO, Reed RK. Epac1<SUP>-/-</SUP> mice have elevated baseline permeability and do not respond to histamine as measured with dynamic contrast-enhanced magnetic resonance imaging with contrast agents of different molecular weights. Acta Physiologica. 2019;225(3). | Title |
|  | Curtis DA, Gansky SA, Plesh O. Deep and Superficial Masseter Muscle Blood Flow in Women. Journal of Prosthodontics-Implant Esthetic and Reconstructive Dentistry. 2012;21(6):472-7. | Title |
|  | Curtis N. Craniofacial biomechanics: An overview of recent multibody modelling studies. Journal of Anatomy. 2011;218(1):16-25. | Title |
|  | Curtis N, Kupczik K, O'Higgins P, Moazen M, Fagan M. Predicting skull loading: Applying multibody dynamics analysis to a macaque skull. Anatomical Record-Advances in Integrative Anatomy and Evolutionary Biology. 2008;291(5):491-501. | Title |
|  | Custódio M, Antunes ES, Alves GBM, Braz-Silva PH. Unexpected diagnosis of an intramuscular myxoma arising from the masseter muscle. British Journal of Oral and Maxillofacial Surgery. 2020;58(1):109-11. | Title |
|  | Custodio W, Gomes SG, Faot F, Garcia RC, Del Bel Cury AA. Occlusal force, electromyographic activity of masticatory muscles and mandibular flexure of subjects with different facial types. J Appl Oral Sci. 2011;19(4):343-9. | Full text  Other outcomes |
|  | Čutović T, Pavlović J, Kozomara R. Radiographic cephalometry analysis of dimensions of condylar processus in persons with mandibular prognathism. Vojnosanitetski Pregled. 2008;65(7):513-9. | Title |
|  | Cvetko E, Janáček J, Kubínová L, Eržen I. The capillary pattern in human masseter muscle during ageing. Image Analysis and Stereology. 2013;32(3):135-44. | Title |
|  | Cvetko E, Karen P, Eržen I. Wearing of complete dentures reduces slow fibre and enhances hybrid fibre fraction in masseter muscle. Journal of Oral Rehabilitation. 2012;39(8):608-14. | Title |
|  | D'Alessandro G, Tagariello T, Piana G. Oral and craniofacial findings in a patient with methylmalonic aciduria and homocystinuria: review and a case report. Minerva Stomatol. 2010;59(3):129-37. | Title |
|  | D'Andrea E, Barbaix E. Anatomic research on the perioral muscles, functional matrix of the maxillary and mandibular bones. Surgical and Radiologic Anatomy. 2006;28(3):261-6. | Abstract |
|  | D'Antò V, Michelotti A, Esposito L, Zagari A, Liguori R, Sacchetti L. Nonsynonimous mutation of catechol-O-methyl-transferase (COMT) gene in a patient with temporomandibular disorder. Journal of Science and Medicine in Sport. 2010;13(6):174-9. | Title |
|  | d'Apuzzo F, Minervini G, Grassia V, Rotolo RP, Perillo L, Nucci L. Mandibular Coronoid Process Hypertrophy: Diagnosis and 20-Year Follow-Up with CBCT, MRI and EMG Evaluations. Applied Sciences-Basel. 2021;11(10). | Title |
|  | D'Ippolito S, Ursini R, Giuliante L, Deli R. Correlations between mandibular asymmetries and temporomandibular disorders (TMD). International Orthodontics. 2014;12(2):222-38. | Title |
|  | D'Orlandi G, Raguzzi L, Defraia E, Pierleoni F. Functional approach to a Class II patient with upper first molar impaction. Indian Journal of Dental Research. 2014;25(5):662-6. | Title |
|  | D’Arcangelo C, Vadini M, Buonvivere M, De Angelis F. Safe clinical technique for increasing the occlusal vertical dimension in case of erosive wear and missing teeth. Clinical Case Reports. 2021;9(12). | Title |
|  | da Consolação Canuto Salgueiro M, Carvalho Bortoletto C, Ratto Tempestini Horliana AC, Costa Mota AC, Jansiski Motta L, de Barros Motta P, et al. Evaluation of muscle activity, bite force and salivary cortisol in children with bruxism before and after low level laser applied to acupoints: study protocol for a randomised controlled trial. BMC complementary and alternative medicine. 2017;17:1‐7. | Title |
|  | Da Silva AP, Sassi FC, Bastos E, Alonso N, Furquim De Andrade CR. Oral motor and electromyographic characterization of adults with facial fractures: A comparison between different fracture severities. Clinics. 2017;72(5):276-83. | Title |
|  | da Silva H, Cecanho R. Cephalometric changes produced by locally applied anabolic steroid in Wistar rats. Archives of Oral Biology. 2009;54(4):389-95. | Title |
|  | da Silva JB, Giglio LD, Regalo SH, de Mello-Filho FV, Trawitzki LVV. Effect of dentofacial deformity on maximum isometric tongue strength. Journal of Oral Rehabilitation. 2013;40(4):247-51. | Title |
|  | Daboul A, Schwahn C, Bülow R, Kiliaridis S, Kocher T, Klinke T, et al. Influence of Age and Tooth Loss on Masticatory Muscles Characteristics: A Population Based MR Imaging Study. Journal of Nutrition, Health and Aging. 2018;22(7):829-36. | Title |
|  | Da̧browski J, Piȩtka T, Przybysz J, Krzymański G. Acinic cell carcinoma of the parotid gland. Case report. Wspolczesna Onkologia. 2011;15(1):47-50. | Title |
|  | Daegling DJ, Hotzman JL. Functional significance of cortical bone distribution in anthropoid mandibles: An in vitro assessment of bone strain under combined loads. American Journal of Physical Anthropology. 2003;122(1):38-50. | Title |
|  | Dagsuyu IM, Kuftinec MM, Voudouris JC. Role of lateral pterygoid muscle in functional orthopedic treatment. American journal of orthodontics and dentofacial orthopedics : official publication of the American Association of Orthodontists, its constituent societies, and the American Board of Orthodontics. 2004;125(4):19A. | Title |
|  | Dahan J. Die isometrische Muskelkontraktion: Eine Wirkungskraft in den funktionskieferorthopädischen Geräten. Fortschritte der Kieferorthopädie. 1983;44(4):282-97. | Title |
|  | Dahan J. Muscle involvement in functional jaw orthopedics. Bilten Udruzenja ortodonata Jugoslavije = Bulletin of Orthodontic Society of Yugoslavia. 1988;21(1):47-64. | Title |
|  | Dahlström L, Kahnberg KE, Lindahl L. 15 years follow-up on condylar fractures. International Journal of Oral and Maxillofacial Surgery. 1989;18(1):18-23. | Title |
|  | Dai Z, Hou M, Ma W, Song DL, Zhang CX, Zhou WY. Evaluation of the Transverse Displacement of the Proximal Segment After Bilateral Sagittal Split Ramus Osteotomy With Different Lingual Split Patterns and Advancement Amounts Using the Finite Element Method. Journal of Oral and Maxillofacial Surgery. 2016;74(11). | Title |
|  | Dal Santo F, Ellis Iii E, Throckmorton GS. The effects of zygomatic complex fracture on masseteric muscle force. Journal of Oral and Maxillofacial Surgery. 1992;50(8):791-9. | Title |
|  | Dalkiz M, Yurdakul RH, Pakdemirli E, Beydemir B. Recurrent osseous choristoma of the masseter muscle: Case report. Journal of Oral and Maxillofacial Surgery. 2001;59(7):836-9. | Title |
|  | Damodar D, Chan N, Kokot N. Pigmented villonodular synovitis of the temporomandibular joint: Case report and review of the literature. Head and Neck. 2015;37(12):E194-E9. | Title |
|  | Dandriyal R, Giri KY, Alam S, Singh AP. Accidental intraoral formalin injection: A rare case report. Clinics and Practice. 2014;4(3):60-3. | Title |
|  | Daniels SJ. Botulnum toxin type a (bont/a), the mandibular neuromuscular envelope, and bite jumping appliances: skeletal effects. 2018. | Title |
|  | Danilova MA, Ishmurzin PV, Zakharov SV. [The theoretical substantiation of myofunctional correction of sagittal occlusion abnormalities and temporomandibular joint dysfunction]. Stomatologiia (Mosk). 2012;91(3):65-9. | Title |
|  | Daramola OO, Sabino ML, Flanary VA. Unilateral masticatory muscle hypertrophy with mandibular ramus hyperostosis. International Journal of Pediatric Otorhinolaryngology Extra. 2011;6(4):403-5. | Title |
|  | Das S, Nayak UK, Buggavetti R, Sekhar S. Adenoid Cystic Carcinoma of Accessory Parotid Gland: A Case Report. Journal of Oral and Maxillofacial Surgery. 2016;74(5):1097.e1-.e5. | Title |
|  | Dasilva AF, Love T, Dos M. Santos H, Martella A, Enoch MA, Hodgkinson C, et al. BDNF Val66Met is associated with functional and structural brain changes in dopaminergic pathways in relation to the human trigeminal pain experience. Headache. 2010;50:6. | Title |
|  | Davies JC, Charles M, Cantelmi D, Liebgott B, Ravichandiran M, Ravichandiran K, Agur AM. Lateral pterygoid muscle: A three-dimensional analysis of neuromuscular partitioning. Clinical Anatomy. 2012;25(5):576-83. | Abstract |
|  | Davis JS. Functional Morphology of Mastication in Musteloid Carnivorans [Dissertation/Thesis]2014. | Title |
|  | Davis JS, Williams SH. The influence of diet on masticatory motor patterns in musteloid carnivorans: An analysis of jaw adductor activity in ferrets (<i>Mustela putorius furo</i>) and kinkajous (<i>Potos flavus</i>). Journal of Experimental Zoology Part a-Ecological and Integrative Physiology. 2017;327(9):551-61. | Title |
|  | de Almeida ANS, de Souza Ferreira SL, Balata PMM, da Cunha DA, Pernambuco L, da Silva HJ. Thermography in complementary assessments of head and neck muscles: A scoping review. Journal of Oral Rehabilitation. 2022;49(12):1188-96. | Title |
|  | De Boer EWJ, Dijkstra PU, Stegenga B, De Bont LGM, Spijkervet FKL. Value of cone-beam computed tomography in the process of diagnosis and management of disorders of the temporomandibular joint. British Journal of Oral and Maxillofacial Surgery. 2014;52(3):241-6. | Title |
|  | de Caxias FP, Exposto FG, Turcio KHL, Dos Santos DM, Svensson P. Nerve Growth Factor-Induced Sensitization of the Sternocleidomastoid Muscle and Its Effects on Trigeminal Muscle Sensitivity and Pain Profiles: a Randomized Double-Blind Controlled Study. Journal of oral & facial pain and headache. 2021;35(1):7‐16. | Title |
|  | de Caxias FP, Túrcio KHL, Neto C, de Athayde FRF, Goiato MC, dos Santos DM. Effects of rehabilitation with complete dentures on bite force and electromyography of jaw and neck muscles and the correlation with occlusal vertical dimension. Clinical Oral Investigations. 2021;25(7):4691-8. | Title |
|  | De Groot R, Merkx T, De Haan T, Rosenberg A, Speksnijder C. Tongue function and its influence on masticatory performance in patients treated for oral cancer; a 5-year prospective study. Supportive Care in Cancer. 2019;27(1):S152-S3. | Title |
|  | de Mello EC, Regalo SCH, Diniz LH, Lage JB, Ribeiro MF, Junior DEB, et al. Electromyographic analysis of stomatognathic muscles in elderly after hippotherapy. PLoS ONE. 2020;15(8 August). | Title |
|  | De Paolo M, Gracis M, Lacava G, Vapniarsky N, Arzi B. Management of bilateral pterygoid myositis ossificans-like lesion in dogs. Frontiers in Veterinary Science. 2022;9. | Title |
|  | De Ponte FS, Anastasi G, Catalfamo L, Calvo A, Runci M. Dentofacial asymmetries, the study of the morphological and functional aspects with tractographic imaging. International Journal of Oral and Maxillofacial Surgery. 2017;46:13-4. | Abstract |
|  | De Riu G, Meloni SM, Gobbi R, Contini M, Tullio A. Soft-tissue chondroma of the masticatory space. International Journal of Oral and Maxillofacial Surgery. 2007;36(2):174-6. | Title |
|  | De Rossi M, De Rossi A, Hallak JEC, Vitti M, Regalo SCH. Electromyographic evaluation in children having rapid maxillary expansion. American Journal of Orthodontics and Dentofacial Orthopedics. 2009;136(3):355-60. | Title |
|  | de Rossi M, Palinkas M, de Lima-Lucas B, Santos CM, Semprini M, Oliveira LF, et al. Masticatory muscle activity evaluation by electromyography in subjects with zygomatic implants. Medicina Oral, Patologia Oral y Cirugia Bucal. 2017;22(3):e392-e7. | Title |
|  | de Sonnaville WFC, Steenks MH, Zuithoff NPA, Wulffraat NM, Rosenberg AJWP, Speksnijder CM. Reliability and measurement error of anterior maximum voluntary bite force in children with juvenile idiopathic arthritis and healthy children. PLoS ONE. 2023;18(1 January). | Title |
|  | De Souza MA, Krefer AG, Benvenutti Borba G, Vizinoni E Silva GJ, Franco APGO, Gamba HR, editors. Generation of 3D thermal models for dentistry applications. Proceedings of the Annual International Conference of the IEEE Engineering in Medicine and Biology Society, EMBS; 2016. | Title |
|  | de Zee M, Dalstra M, Cattaneo PM, Rasmussen J, Svensson P, Melsen B. Validation of a musculo-skeletal model of the mandible and its application to mandibular distraction osteogenesis. Journal of Biomechanics. 2007;40(6):1192-201. | Title |
|  | Dean D, Bookstein FL, Koneru S, Lee JH, Kamath J, Cutting CB, et al. Average African American three-dimensional computed tomography skull images: The potential clinical importance of ethnicity and sex. Journal of Craniofacial Surgery. 1998;9(4):348-59. | Abstract |
|  | Dean JS, Throckmorton GS, Ellis E, 3rd, Sinn DP. A preliminary study of maximum voluntary bite force and jaw muscle efficiency in pre-orthognathic surgery patients. J Oral Maxillofac Surg. 1992;50(12):1284-8. | Title |
|  | Decaup PH, Couture C, Garot E. Is the distribution of cortical bone in the mandibular corpus and symphysis linked to loading environment in modern humans? A systematic review. Archives of Oral Biology. 2023;152. | Title |
|  | Dechow P, Wang Q, Smith LP. A biomedical synthesis of the zygoma: Bone elastic properties, morphology and function, and reconstructive medicine. FASEB Journal. 2015;29(1). | Title |
|  | Dechow PC. CRANIOFACIAL BIOMECHANICS, ELECTROMYOGRAPHY AND OCCLUSAL FORCE AT DIFFERENT GAPES IN THE MUSCLES OF MASTICATION. Anatomical Record. 1987;218(1):A31-A. | Title |
|  | Defabianis P. TMJ fractures in children: importance of functional activation of muscles in preventing mandibular asymmetries and facial maldevelopment. Funct Orthod. 2002;19(2):34-42. | Title |
|  | Defabianis P. Treatment of condylar fractures in children and youths: the clinical value of the occlusal plane orientation and correlation with facial development (case reports). The Journal of clinical pediatric dentistry. 2002;26(3):243-50. | Title |
|  | Defabianis P, Carli E, Cogo C, Ninivaggi R. Mechanics of facial growth in young patients. European Journal of Paediatric Dentistry. 2022;23(4):288-90. | Title |
|  | Deguchi T, Iwahara K. Electromyographic investigation of chin cup therapy in class III malocclusion. Angle Orthodontist. 1998;68(5):419-24. | Title |
|  | Deguchi T, Kumai T, Garetto L. Statistics of differential Lissajous EMG for normal occlusion and Class II malocclusion. American Journal of Orthodontics and Dentofacial Orthopedics. 1994;105(1):42-8. | Full text  Other outcomes |
|  | Delaire J. The evolution of the lower jaw and the jaw joint, from reptiles to man. Revue de stomatologie et de chirurgie maxillo-faciale. 1998;99(1):3-10. | Title |
|  | Dell'Aversana Orabona G, Abbate V, Piombino P, Iaconetta G, Califano L. Midcheek mass: 10 year of clinical experience. Journal of Cranio-Maxillofacial Surgery. 2014;42(7):e353-e8. | Title |
|  | Dellavia C, Rosati R. Instrumental evaluations of the stomatognathic apparatus: static and dynamic tests. European Journal of Translational Myology. 2023;33(1):50-1. | Title |
|  | Demir A, Uysal T, Basciftci FA, Guray E. The association of occlusal factors with masticatory muscle tenderness in 10- to 19-year old Turkish subjects. Angle Orthodontist. 2005;75(1):40-6. | Title |
|  | Demir N, Taner T, Tekcicek M, Serel S, Yýlmaz O, Baharoolu E, et al. Multidisiplinary approach in a case with congenital cranial nerve dysplasia. Neuromuscular Disorders. 2010;20(9-10):618. | Title |
|  | Demiralp K, Orhan K, Kursun-Çakmak ES, Görürgöz C, Bayrak S. Comparison of Cone Beam Computed Tomography and ultrasonography with two types of probes in the detection of opaque and non-opaque foreign bodies. Medical Ultrasonography. 2018;20(4):467-74. | Title |
|  | Demirjian A, David B. Learning medical and dental sciences through interactive multi-media. Medinfo. 1995;8 Pt 2:1705. | Title |
|  | Denes BJ, Bresin A, Kiliaridis S. The influence of altered functional loading and posterior bite-blocks on the periodontal ligament space and alveolar bone thickness in rats. Acta Odontologica Scandinavica. 2016;74(7):518-24. | Title |
|  | Denes BJ, Lazzarotto B, Bresin A, Kiliaridis S. Effect of different masticatory functional demands on the 3D mandibular condyle morphology of growing rats using posterior bite-blocks. European Journal of Orthodontics. 2018;40(3):312-6. | Title |
|  | Dergin G, Kilic C, Gozneli R, Yildirim D, Garip H, Moroglu S. Evaluating the correlation between the lateral pterygoid muscle attachment type and internal derangement of the temporomandibular joint with an emphasis on MR imaging findings. Journal of Cranio-Maxillofacial Surgery. 2012;40(5):459-63. | Title |
|  | Derin S, Sahan M, Hazer DB, Sahan L. Subdural empyema and unilateral pansinusitis due to a tooth infection. BMJ Case Reports. 2015;2015. | Title |
|  | Derwich M, Mitus-Kenig M, Pawlowska E. Interdisciplinary Approach to the Temporomandibular Joint Osteoarthritis-Review of the Literature. Medicina (Kaunas). 2020;56(5). | Title |
|  | Deshayes MJ. [Dentofacial Orthopedics to treat facial asymmetries before six years of age. How to balance craniofacial growth and enhance temporomandibular function]. L' Orthodontie française. 2010;81(3):189-207. | Title |
|  | Dessem D, Moritani M, Ambalavanar R. Nociceptive craniofacial muscle primary afferent neurons synapse in both the rostral and caudal brain stem. Journal of Neurophysiology. 2007;98(1):214-23. | Title |
|  | Dhawan A, Shenoy AM, Chavan P, Sandhu S, Sriprakash D. Synovial sarcoma of the infratemporal fossa with extension into the oral cavity - A rare presentation and literature review. Journal of Oral and Maxillofacial Surgery. 2012;70(12):2923-9. | Title |
|  | Diaconescu N, Ardelean I, Erdei A, Popovici M, Popovici D. Biomechanical interpretations of some bony structures in the skull. Romanian journal of morphology and embryology = Revue roumaine de morphologie et embryologie. 1990;36(2):81-91. | Title |
|  | Dicker G, Koolstra JH, Castelijns J, Schijndel RV, Tuinzing B. Positional changes of jaw closing muscles after surgical mandibular advancement. International Journal of Oral and Maxillofacial Surgery. 2011;40(10):1078. | Title |
|  | Dicker G, Van Spronsen P, Van Schijndel R, van Ginkel F, Manoliu R, Boom H, Tuinzing DB. Adaptation of jaw closing muscles after surgical mandibular advancement procedures in different vertical craniofacial types: a magnetic resonance imaging study. Oral Surg Oral Med Oral Pathol Oral Radiol Endod. 2007;103(4):475-82. | Title |
|  | Dicker GJ, Castelijns JA, Tuinzing DB, Stoelinga PJ. Do the changes in muscle mass, muscle direction, and rotations of the condyles that occur after sagittal split advancement osteotomies play a role in the aetiology of progressive condylar resorption? Int J Oral Maxillofac Surg. 2015;44(5):627-31. | Title |
|  | Dicker GJ, Koolstra JH, Castelijns JA, Van Schijndel RA, Tuinzing DB. Positional changes of the masseter and medial pterygoid muscles after surgical mandibular advancement procedures: An MRI study. International Journal of Oral and Maxillofacial Surgery. 2012;41(8):922-9. | Title |
|  | Dicker GJ, Tuijt M, Koolstra JH, Van Schijndel RA, Castelijns JA, Tuinzing DB. Static and dynamic loading of mandibular condyles and their positional changes after bilateral sagittal split advancement osteotomies. International Journal of Oral and Maxillofacial Surgery. 2012;41(9):1131-6. | Title |
|  | Dicker GJ, van Spronsen PH, van Ginkel FC, Castelijns JA, van Schijndel RA, Boom HPW, Tuinzing DB. Adaptation of lateral pterygoid and anterior digastric muscles after surgical mandibular advancement procedures in different vertical craniofacial types: A magnetic resonance imaging study. Oral Surgery, Oral Medicine, Oral Pathology, Oral Radiology and Endodontology. 2008;105(6):688-97. | Title |
|  | Dickinson E, Basham C, Rana A, Hartstone-Rose A. Visualization and Quantification of Digitally Dissected Muscle Fascicles in the Masticatory Muscles of <i>Callithrix jacchus</i> Using Nondestructive DiceCT. Anatomical Record-Advances in Integrative Anatomy and Evolutionary Biology. 2019;302(11):1891-900. | Title |
|  | Dickinson E, Fitton LC, Kupczik K. Modelling ontogenetic changes in masticatory performance within Macaca fascicularis and their impact upon dietary and social ecology: A multibody dynamics study. American Journal of Physical Anthropology. 2019;168:58. | Title |
|  | Dickinson E, Stark H, Kupczik K. Non-Destructive Determination of Muscle Architectural Variables Through the Use of DiceCT. Anatomical Record-Advances in Integrative Anatomy and Evolutionary Biology. 2018;301(2):363-77. | Abstract |
|  | Dieterle MP, Husari A, Steinberg T, Wang X, Ramminger I, Tomakidi P. From the matrix to the nucleus and back: Mechanobiology in the light of health, pathologies, and regeneration of oral periodontal tissues. Biomolecules. 2021;11(6). | Title |
|  | Dilip Kumar B, Dave B, Meghana SM. Cysticercosis of masseter. Indian Journal of Dental Research. 2011;22(4):617. | Title |
|  | Dimova M, Arnautska H, Konstantinova D, Gerdzhikov I, Georgiev T, Yovchev D. Correlations between findings of occlusal and manual analysis in TMD-patients. Journal of IMAB - Annual Proceeding (Scientific Papers). 2016;22(3):1242-7. | Title |
|  | Dimova-Gabrovska M, Dimitrova D, Georgiev T. Application of MRI in the diagnostics of m. masseter. Journal of IMAB - Annual Proceeding (Scientific Papers). 2017;23(2):1607-10. | Abstract |
|  | Dittmar M, Spruss T, Schuierer G, Horn M. External carotid artery territory ischemia impairs outcome in the endovascular filament model of middle cerebral artery occlusion in rats. Stroke. 2003;34(9):2252-7. | Title |
|  | Dizdarevic D, Masic T, Muslic E. Lower jaw grip strength in healthy and sick population measured by special force transducer. Acta Informatica Medica. 2017;25(4):236-9. | Title |
|  | Djokic B, Ristic B, Kocic M. Ultrasonography findings of jaws disorders. Ultraschall in der Medizin, Supplement. 2013;34. | Title |
|  | Dogdas B, Stout D, Chatziioannou AF, Leahy RM. Digimouse: a 3D whole body mouse atlas from CT and cryosection data. Physics in Medicine and Biology. 2007;52(3):577-87. | Title |
|  | Dogru SC, Cansiz E, Arslan YZ. Biomechanical evaluation of resorbable and titanium miniplates and of single and double miniplates for the treatment of mandibular condyle fractures. Biocybernetics and Biomedical Engineering. 2019;39(3):709-18. | Title |
|  | Dominguez MF, Sanchez Sanchez R, Gonzalez FS, Perticone MAR, Gonzalez JMM, Mancha De La Plata M. Synovial sarcoma of the masticator space: Report of a case. Journal of Oral and Maxillofacial Surgery. 2011;69(11):e482-e7. | Title |
|  | Doshi UH, Bhad-Patil WA. Early management of skeletal open bite with spring-loaded and magnetic bite blocks. World journal of orthodontics. 2010;11(2):107‐16. | Title |
|  | Dreyer CJ. The stability of the dentition and the integrity of its supporting structures. American Journal of Orthodontics. 1970;58(5):433-47. | Title |
|  | Droste S, Jörg J, Lux G, Sellhaus B, Schröder JM. Immunomediated interstitial myositis and aplastic anemia accompanying a benign thymoma. Aktuelle Neurologie. 2001;28(6):281-4. | Title |
|  | Du X, Hägg U. Muscular adaptation to gradual advancement of the mandible. Angle Orthodontist. 2003;73(5):525-31. | Title |
|  | Dua R, McGurk M, Fan K. Adenoid cystic carcinoma mimicking pericoronitis. International Journal of Oral and Maxillofacial Surgery. 2013;42(10):1314. | Title |
|  | Duan J, Li H, Zhen T, Liang J, Ge S, Zhang F, Han A. A clinicopathologic study of 13 cases of primary lymphoma in soft tissue and review of literature. American Journal of Blood Research. 2022;12(4):144-55. | Title |
|  | Duarte Gavião MB, Durval Lemos A, Diaz Serra M, Riqueto Gambareli F, Nobre Dos Santos M. Masticatory performance and bite force in relation to signs and symptoms of temporomandibular disorders in children. Minerva stomatologica. 2006;55(10):529-39. | Title |
|  | Duggal I, Sidhu MS, Chawla A, Dabas A, Dhimole VK. Effects of miniplate anchored Herbst appliance on skeletal, dental and masticatory structures of the craniomandibular apparatus: A finite element study. Int Orthod. 2021;19(2):301-9. | Title |
|  | Dumitru D, Wasserburger LB. Electrophysiologic investigation of mandibular nerve injury. Archives of Physical Medicine and Rehabilitation. 1991;72(3):230-2. | Title |
|  | Dumont ER, Herrel A. The effects of gape angle and bite point on bite force in bats. Journal of Experimental Biology. 2003;206(13):2117-23. | Title |
|  | Dumoulin A, Schmidt H, Rathjen FG. Sensory Neurons: The Formation of T-Shaped Branches Is Dependent on a cGMP-Dependent Signaling Cascade. Neuroscientist. 2021;27(1):47-57. | Title |
|  | Dunn D, Howe A. Targeted muscle relaxation in the treatment of parafunction and TMD - Part 2. Australasian dental practice. 2016;27(5):154‐8. | Title |
|  | Dunphy B, Quach H, Ghaly GA. Surgical management of a long-standing temporomandibular joint dislocation: A case report. British Journal of Surgery. 2021;108(SUPPL 6):vi171. | Title |
|  | Ebrahimi E, Li Z, Bikey D, Hannam A, Holmes H, Liebgott B, Agur A. The internal geometry of the masseter muscle: A 3D map to guide in vivo ultrasound. Journal of Oral and Maxillofacial Surgery. 2015;73(9):e75. | Title |
|  | Eckardt L, Harzer W, Schneevoigt R. Comparative study of excitation patterns in the masseter muscle before and after orthognathic surgery. Journal of Cranio-Maxillo-Facial Surgery. 1997;25(6):344-52. | Title |
|  | Edmonds HM, Daly ES, Smail IE. Zygomatic arch root position in relation to dietary type in haplorhine primates. Anatomical Record-Advances in Integrative Anatomy and Evolutionary Biology. 2023. | Title |
|  | Edmonds HM, Glowacka H. The ontogeny of maximum bite force in humans. Journal of Anatomy. 2020;237(3):529-42. | Title |
|  | Eekhoff EMW, Netelenbos JC, de Graaf P, Hoebink M, Bravenboer N, Micha D, et al. Flare-Up After Maxillofacial Surgery in a Patient With Fibrodysplasia Ossificans Progressiva: An [18F]-NaF PET/CT Study and a Systematic Review. JBMR Plus. 2018;2(1):55-8. | Title |
|  | Egli F, Botteron S, Morel C, Kiliaridis S. Growing patients with Duchenne muscular dystrophy: longitudinal changes in their dentofacial morphology and orofacial functional capacities. Eur J Orthod. 2018;40(2):140-8. | Title |
|  | Ehmer U, Sanftenberg U, Broll P. A gnathographic research of mandibular border movements and chewing patterns before and after surgical correction of a mandibular setback and progenia. Fortschritte der Kieferorthopädie. 1991;52(5):274-81. | Title |
|  | Ehrlich R, Garlick D, Ninio M. The effect of jaw clenching on the electromyographic activities of 2 neck and 2 trunk muscles. Journal of Oral and Facial Pain and Headache. 1999;13(2):115-20. | Title |
|  | El Khatib N, Nehme A, Nasser S, Moukarzel N, Abtar HK. Intramasseteric Schwanoma mimicking an isolated cheek mass: Case report and review of literature. International Journal of Surgery Case Reports. 2018;46:24-7. | Title |
|  | Elfekey ESA, Hanafy HM, Salem H, Hasanin ME. Effect of Topical Diclofenac Phonophoresis in Treatment of Temporomandibular Joint Disorders in Females During Reproductive Age. NeuroQuantology. 2022;20(15):4811‐8. | Title |
|  | Ellis E, 3rd, Throckmorton G, Sinn DP. Functional characteristics of patients with anterior open bite before and after surgical correction. Int J Adult Orthodon Orthognath Surg. 1996;11(3):211-23. | Title |
|  | Ellis E, Throckmorton GS. Treatment of mandibular condylar process fractures: Biological considerations. Journal of Oral and Maxillofacial Surgery. 2005;63(1):115-34. | Title |
|  | Ellis ED, Throckmorton GS. Bite forces after open or closed treatment of mandibular condylar process fractures. Journal of Oral and Maxillofacial Surgery. 2001;59(4):389-95. | Title |
|  | Ellis Iii E, Throckmorton GS, Sinn DP. Bite forces before and after surgical correction of mandibular prognathism. Journal of Oral and Maxillofacial Surgery. 1996;54(2):176-81. | Title |
|  | Ellis JL, Thomason J, Kebreab E, Zubair K, France J. Cranial dimensions and forces of biting in the domestic dog. Journal of Anatomy. 2009;214(3):362-73. | Title |
|  | Elmshiti HG. Evaluation of Submandibular Infections using 3-Dimensional Reconstruction of Computed Tomography Images [Dissertation/Thesis]2016. | Title |
|  | Elsayed N, Shimo T, Harada F, Takeda S, Hiraki D, Abiko Y, et al. Masticatory muscle tendon-aponeurosis hyperplasia diagnosed as temporomandibular joint disorder: A case report and review of literature. International Journal of Surgery Case Reports. 2021;78:120-5. | Title |
|  | Elsayed N, Shimo T, Tashiro M, Nakayama E, Nagayasu H. Disuse atrophy of masticatory muscles after intracranial trigeminal schwannoma resection: A case report and review of literature. International Journal of Surgery Case Reports. 2020;75:23-8. | Title |
|  | Elumalai M, Doraikannan SS, Indiran MA, Rathinavelu PK. Association of signs and symptoms of temporomandibular joint disorder between gender, partial edentulism, and morphological occlusion among dental patients in Chennai. Drug Invention Today. 2018;10(Special Issue 4):3617-22. | Title |
|  | Emara AS, Faramawey MI, Hassaan MA, Hakam MM. Botulinum toxin injection for management of temporomandibular joint clicking. International Journal of Oral and Maxillofacial Surgery. 2013;42(6):759-64. | Title |
|  | Eng CM, Lieberman DE, Zink KD, Peters MA. Bite force and occlusal stress production in hominin evolution. Am J Phys Anthropol. 2013;151(4):544-57. | Title |
|  | Eng CM, Ward SR, Vinyard CJ, Taylor AB. The morphology of the masticatory apparatus facilitates muscle force production at wide jaw gapes in tree-gouging common marmosets (<i>Callithrix jacchus</i>). Journal of Experimental Biology. 2009;212(24):4040-55. | Title |
|  | Engvall M, Birkhed D. Oral sugar clearance and other caries-related factors in patients with myotonic dystrophy. Acta Odontologica Scandinavica. 1997;55(2):111-5. | Title |
|  | Enomoto A, Watahiki J, Yamaguchi T, Irie T, Tachikawa T, Maki K. Effects of mastication on mandibular growth evaluated by microcomputed tomography. European Journal of Orthodontics. 2010;32(1):66-70. | Title |
|  | Enright KM, Nikolis A. A randomized, open-label, evaluator-blinded trial on the use of incobotulinumtoxinA for the treatment of masseteric hypertrophy: post-therapy changes in three-dimensional volumetric analyses. Toxicon. 2021;190:S20‐S1. | Title |
|  | Eppley BL, Elluru R, Sadove AM. Lower facial recontouring in craniofacial asymmetry. Annals of Plastic Surgery. 1992;29(5):464-8. | Title |
|  | Erdem A, Kilic N, Eröz B. Changes in soft tissue profile and electromyographic activity after activator treatment. Australian orthodontic journal. 2009;25(2):116‐22. | Title |
|  | Eren H, Bagis N, Biyikoglu B. Clinical and ultrasonographic evaluation of masticatory muscles in young subjects with and without bruxism. Meandros Medical and Dental Journal. 2021;22(1):38-46. | Title |
|  | Eriksson PO, Zafar H, Häggman-Henrikson B. Deranged jaw-neck motor control in whiplash-associated disorders. European Journal of Oral Sciences. 2004;112(1):25-32. | Title |
|  | Ernberg M, Schopka JH, Fougeront N, Svensson P. Changes in jaw muscle EMG activity and pain after third molar surgery. Journal of Oral Rehabilitation. 2007;34(1):15-26. | Title |
|  | Erturk AF, Yelken Kendirci M, Ozcan I, Gokcen Rohlig B. Use of ultrasonography in the diagnosis of temporomandibular disorders: a prospective clinical study. Oral Radiology. 2023;39(2):282-91. | Title |
|  | Etoz M, Demirbas AE, Topsakal KG, Etoz OA, Kaya MO, Alkan A. Sonoelastographic evaluation of the masseter muscle before and after mandibular setback surgery. Nigerian journal of clinical practice. 2020;23(8):1095‐102. | Title |
|  | Etoz OA, Ataoglu H, Erdal ME. Association between trytophan hydroxylase gene polymorphism and painful non-osseous temporomandibular disorders. Saudi Medical Journal. 2008;29(9):1352-4. | Title |
|  | Ezure T, Hosoi J, Amano S, Tsuchiya T. Sagging of the cheek is related to skin elasticity, fat mass and mimetic muscle function. Skin Research and Technology. 2009;15(3):299-305. | Title |
|  | Fabi SG, Park JY, Ho WWS, Vachiramon V, Dayan S. Aesthetic considerations for treating the Asian patient: Thriving in diversity international roundtable series. Journal of Cosmetic Dermatology. 2023;22(6):1805-13. | Title |
|  | Fabre AC, Perry JMG, Hartstone-Rose A, Lowie A, Boens A, Dumont M. Do Muscles Constrain Skull Shape Evolution in Strepsirrhines? Anatomical Record-Advances in Integrative Anatomy and Evolutionary Biology. 2018;301(2):291-310. | Title |
|  | Fabre PH, Herrel A, Fitriana Y, Meslin L, Hautier L. Masticatory muscle architecture in a water-rat from Australasia (Murinae, <i>Hydromys</i>) and its implication for the evolution of carnivory in rodents. Journal of Anatomy. 2017;231(3):380-97. | Title |
|  | Falcinelli C, Li Z, Lam WW, Stanisz GJ, Agur AM, Whyne CM. Diffusion-tensor imaging versus digitization in reconstructing the masseter architecture. Journal of Biomechanical Engineering. 2018;140(11). | Abstract |
|  | Falcini F, Melchiorre D, Cappelli S, Carnesecchi G, Biondi K, Bosco M, Matucci Cerinic M. Temporomandibular joints (TMJ) involvement in juvenile idiopathic arthritis (JIA): Longitudinal evaluation after orthopaedic treatment. Annals of the Rheumatic Disease. 2013;71. | Title |
|  | Falcini F, Melchiorre D, Carnesecchi G, Bertini F, Biondi K, Bosco M, Matucci-Cerinic M. Orthopaedic treatment of temporomandibular joint (TMJ) damage in adolescents with juvenile idiopathic arthritis (JIA): Longitudinal evaluation. Arthritis and Rheumatism. 2012;64:S855. | Title |
|  | Falleti J, De Cecio R, Mentone A, Lamberti V, Friscia M, De Biasi S, et al. Extraskeletal chondroma of the masseter muscle: a case report with review of the literature. International Journal of Oral and Maxillofacial Surgery. 2009;38(8):895-9. | Title |
|  | Falque E, Benoit R. Musculoskeletal connections. Study of two cases of oto-mandibular dysplasia. L' Orthodontie française. 2005;76(3):229-38. | Title |
|  | Fantozzi MPT, Diciotti S, Tessa C, Castagna B, Chiesa D, Barresi M, et al. Unbalanced occlusion modifies the pattern of brain activity during execution of a finger to thumb motor task. Frontiers in Neuroscience. 2019;13(MAY). | Title |
|  | Farella M, Bakke M, Michelotti A, Rapuano A, Martina R. Masseter thickness, endurance and exercise-induced pain in subjects with different vertical craniofacial morphology. Eur J Oral Sci. 2003;111(3):183-8. | Included |
|  | Farella M, Iodice G, Michelotti A, Leonardi R. The relationship between vertical craniofacial morphology and the sagittal path of mandibular movements. Journal of Oral Rehabilitation. 2005;32(12):857-62. | Title |
|  | Farella M, Michelotti A, Carbone G, Gallo LM, Palla S, Martina R. Habitual daily masseter activity of subjects with different vertical craniofacial morphology. European Journal of Oral Sciences. 2005;113(5):380-5. | Abstract |
|  | Farella M, Palumbo A, Milani S, Avecone S, Gallo LM, Michelotti A. Synergist coactivation and substitution pattern of the human masseter and temporalis muscles during sustained static contractions. Clinical Neurophysiology. 2009;120(1):190-7. | Title |
|  | Farhat de Araujo A, Kazutoyo da Paixão Uyeda D, Almeida da Silva R. Pseudo-ankylosis of the temporomandibular joint due to hyperplasia of articular eminence: Rare case. Oral and Maxillofacial Surgery Cases. 2020;6(3). | Title |
|  | Farooq M, Sazonov E. Segmentation and Characterization of Chewing Bouts by Monitoring Temporalis Muscle Using Smart Glasses with Piezoelectric Sensor. IEEE Journal of Biomedical and Health Informatics. 2017;21(6):1495-503. | Title |
|  | Farronato G, Giannini L, Galbiati G, Grillo E, Maspero C. Occlus-o-Guide® versus Andresen activator appliance: Neuromuscular evaluation. Progress in Orthodontics. 2013;14(1):1-6. | Title |
|  | Farronato G, Giannini L, Galbiati G, Sesso G, Maspero C. Orthodontic-surgical treatment: Neuromuscular evaluation in skeletal Class II and Class III patients. Progress in Orthodontics. 2012;13(3):226-36. | Title |
|  | Farronato G, Giannini L, Galbiati G, Stabilini SA, Maspero C. Orthodontic-surgical treatment: Neuromuscular evaluation in open and deep skeletal bite patients. Progress in Orthodontics. 2013;14(1):1-7. | Title |
|  | Farronato G, Giannini L, Galbiati G, Stabilini SA, Sarcina M, Maspero C. Functional evaluation in orthodontic surgical treatment: long-term stability and predictability. Progress in Orthodontics. 2015;16(1). | Title |
|  | Farrugia ME, Robson MD, Clover L, Anslow P, Newsom-Davis J, Kennett R, et al. MRI and clinical studies of facial and bulbar muscle involvement in MuSK antibody-associated myasthenia gravis. Brain. 2006;129(6):1481-92. | Title |
|  | Fartash L. Masticatory muscle volume and function in migraineurs. 1999. | Title |
|  | Faulkner MG, Hatcher DC, Hay A. A three-dimensional investigation of temporomandibular joint loading. Journal of Biomechanics. 1987;20(10):997-1002. | Title |
|  | Feldreich A, Ernberg M, Lund B, Rosén A. Increased β-endorphin levels and generalized decreased pain thresholds in patients with limited jaw opening and movement-evoked pain from the temporomandibular joint. Journal of Oral and Maxillofacial Surgery. 2012;70(3):547-56. | Title |
|  | Fernández-Carnero S, Calvo-Lobo C, Garrido-Marin A, Arias-Buría JL. 2nd Rehabilitative Ultrasound Imaging Symposium in Physical Therapy, Madrid, Spain, 3-5 June 2016. British Journal of Sports Medicine. 2018;52:A1-A4. | Title |
|  | Fernández-Núñez T, Amghar-Maach S, Gay-Escoda C. Efficacy of botulinum toxin in the treatment of bruxism: Systematic review. Medicina Oral Patologia Oral y Cirugia Bucal. 2019;24(4):e416-e24. | Title |
|  | Ferrario VF, Marciandi PV, Tartaglia GM, Dellavia C, Sforza C. Neuromuscular evaluation of post-orthodontic stability: an experimental protocol. The International journal of adult orthodontics and orthognathic surgery. 2002;17(4):307-13. | Title |
|  | Ferrario VF, Sforza C, Miani A, Jr., Colombo A. Simplified cephalometric lines for the estimation of muscular lines of action. Int J Adult Orthodon Orthognath Surg. 1999;14(1):47-54. | Title |
|  | Ferrario VF, Sforza C, Sartori M, Ciusa V. The mechanical advantage of the masseter muscle in subjects with different vertical and sagittal facial morphology. Clin Orthod Res. 1999;2(3):162-70. | Full text  Other outcomes |
|  | Ferrario VF, Sforza C, Serrao G, Fragnito N, Grassi G. The influence of different jaw positions on the endurance and electromyographic pattern of the biceps brachii muscle in young adults with different occlusal characteristics. Journal of Oral Rehabilitation. 2001;28(8):732-9. | Title |
|  | Ferrario VF, Tartaglia GM, Galletta A, Grassi GP, Sforza C. The influence of occlusion on jaw and neck muscle activity: A surface EMG study in healthy young adults. Journal of Oral Rehabilitation. 2006;33(5):341-8. | Title |
|  | Ferré JC, Barbin JY, Helary JL, Lumineau JP. The mandible, an overhanging mechanically suspended structure - Considerations on the system of attachment and servo-command of the mandible. Anatomia Clinica. 1984;6(1):3-10. | Title |
|  | Ferreira B, Da Silva GP, Gonçalves CR, Arnoni VW, Siéssere S, Semprini M, et al. Stomatognathic function in Duchenne muscular dystrophy: a case-control study. Developmental Medicine and Child Neurology. 2016;58(5):516-21. | Title |
|  | Ferreira PM, Sandoval I, Whittle T, Mojaver YN, Murray GM. Reorganization of Masseter and Temporalis Muscle Single Motor Unit Activity During Experimental Masseter Muscle Pain. Journal of Oral & Facial Pain and Headache. 2020;34(1):40-52. | Title |
|  | Ferreira-Cardoso S, Fabre PH, de Thoisy B, Delsuc F, Hautier L. Comparative masticatory myology in anteaters and its implications for interpreting morphological convergence in myrmecophagous placentals. Peerj. 2020;8. | Title |
|  | Ferri J, Ricard D, Genay A. Posterior Vertical Deficiencies of the Mandible: Presentation of a New Corrective Technique and Retrospective Study of 21 Cases. Journal of Oral and Maxillofacial Surgery. 2008;66(1):35-44. | Title |
|  | Festa F, Capasso L, D'Anastasio R, Anastasi G, Festa M, Caputi S, Tecco S. Maxillary and mandibular base size in ancient skulls and of modern humans from Opi, Abruzzi, Italy: a cross-sectional study. World journal of orthodontics. 2010;11(1):e1-4. | Title |
|  | Festa F, Rotelli C, Scarano A, Navarra R, Caulo M, Macrì M. Functional Magnetic Resonance Connectivity in Patients With Temporomadibular Joint Disorders. Frontiers in Neurology. 2021;12. | Title |
|  | Festa P, Arezzo E, Vallogini G, Vittucci AC, Barbuti D, Galeotti A. “Multidisciplinary management of post- infective osteoarthritis and secondary condylar resorption of temporomandibular joint: a case report in a 9 years-old female patient and a review of literature”. Italian Journal of Pediatrics. 2022;48(1). | Title |
|  | Fieux J, Coutand A. Semiology and diagnosis of mandibular prognathism. Revue de Stomatologie et de Chirurgie Maxillo-Faciale. 1968;69(6):449-66. | Title |
|  | Fillietaz-bacigalupo E. Influência de um dispositivo mastigatório com hiperboloide na função mastigatória, nos parâmetros salivares e do sono e no risco de doenças cardiometabólicas em pacientes com sindrome de Down. 2020. p. 98-. | Title |
|  | Finn RA, Throckmorton GS, Bell WH, Legan HL. Biomechanical considerations in the surgical correction of mandibular deficiency. Journal of Oral Surgery. 1980;38(4):257-64. | Title |
|  | Fiorelli G, Merlo P, Dalstra M, Melsen B. Mandibular repositioning in adult patients - an alternative to surgery? A two-year follow-up. Australasian Orthodontic Journal. 2019;35(1):61-70. | Title |
|  | Fitton LC. The form-function complex of the primate masticatory apparatus. 2008. | Title |
|  | Fitton LC, Shi JF, Fagan MJ, O'Higgins P. Masticatory loadings and cranial deformation in Macaca fascicularis: a finite element analysis sensitivity study. Journal of Anatomy. 2012;221(1):55-68. | Title |
|  | Flowers E, Miaskowski C, Conley Y, Hammer MJ, Levine J, Mastick J, et al. Differential expression of genes and differentially perturbed pathways associated with very high evening fatigue in oncology patients receiving chemotherapy. Supportive Care in Cancer. 2018;26(3):739-50. | Title |
|  | Flury D, Vonhochstetter AR, Landolt U, Schmid S. PROLIFERATIVE MYOSITIS - A LITTLE KNOWN PSEUDOMALIGNANT LESION. Schweizerische Medizinische Wochenschrift. 1993;123(1-2):29-34. | Title |
|  | Fogle LL, Glaros AG. CONTRIBUTIONS OF FACIAL MORPHOLOGY, AGE, AND GENDER TO EMG ACTIVITY UNDER BITING AND RESTING CONDITIONS - A CANONICAL CORRELATION-ANALYSIS. Journal of Dental Research. 1995;74(8):1496-500. | Title |
|  | Foley AJ. Bruxism: Prevalent pathology, problematic paleopathology. American Journal of Physical Anthropology. 2018;165:86-7. | Title |
|  | Foley AJ. The daily grind: Assessing bruxism as a potential indicator of stress in archaeological human remains. Journal of Archaeological Science. 2020;117. | Title |
|  | Foley-Friel K, Fitzgerald R, Stanton M, Herlihy T. Case Report: Rhabdomyosarcoma: A cheeky thing. Ultrasound. 2017;25(2):NP43. | Title |
|  | Fonseca VJ, de Moraes ACM, Olate S, Asprino L, de Moraes M. Immunoglobulin G4-Related Disease of the Maxillofacial Region. A Rare Case. International Journal of Morphology. 2018;36(4):1509-13. | Title |
|  | Forgione AG, Mehta NR, Westcott WL. Strength and bite, Part 1: An analytical review. Cranio : the journal of craniomandibular practice. 1991;9(4):305-15. | Title |
|  | Forrester SE, Allen SJ, Presswood RG, Toy AC, Pain MTG. Neuromuscular function in healthy occlusion. Journal of Oral Rehabilitation. 2010;37(9):663-9. | Title |
|  | Forrester SE, Pain MT, Presswood R, Toy A. Effect of occlusal conditions on neuromuscular function for a healthy population. Texas dental journal. 2009;126(3):222-36. | Title |
|  | Forrester SE, Presswood RG, Toy AC, Pain MTG. Occlusal measurement method can affect SEMG activity during occlusion. Journal of Oral Rehabilitation. 2011;38(9):655-60. | Title |
|  | Forsberg CM, Hellsing E. The effect of a lingual arch appliance with anterior bite plane in deep overbite correction. European Journal of Orthodontics. 1984;6(1):107-15. | Title |
|  | Foster TD, Griffiths MI, Gordon PH. The effects of cerebral palsy on the size and form of the skull. American Journal of Orthodontics. 1974;66(1):40-9. | Title |
|  | Foucart JM, Pajoni D, Carpentier P, Pharaboz C. MRI study of temporomandibular joint disk behavior in chilren with hyperpropulsion appliances. L' Orthodontie française. 1998;69(1):79-91. | Title |
|  | Franc DT, Muetzel RL, Robinson PR, Rodriguez CP, Dalton JC, Naughton CE, et al. Cerebral and muscle MRI abnormalities in myotonic dystrophy. Neuromuscular Disorders. 2012;22(6):483-91. | Title |
|  | Franchi L, Branchi R, Tollaro I. Craniofacial changes following early prosthetic treatment in a case of hypohidrotic ectodermal dysplasia with complete anodontia. ASDC J Dent Child. 1998;65(2):116-21. | Title |
|  | Franco AL. Estudo da prevalência de cefaléias primárias e da sua associação com a dor orofacial em pacientes com disfunção temporomandibular crônica. 2009. p. 127-. | Title |
|  | Franco T, La Boria A, Domanico R, Piazzetta GL, Donato G, Allegra E. Rare adult masseteric rhabdomyosarcoma and a review of the literature. Case Reports in Oncology. 2013;6(3):472-9. | Title |
|  | Fränkel R, Fränkel C. Functional aspects of skeletal open bites. Fortschritte der Kieferorthopadie. 1982;43(1):8-18. | Title |
|  | Franks EM, Jeltema M, Luck PJ, Beckley J, Foegeding EA, Vinyard CJ. Morphological and masticatory performance variation of mouth behavior groups. Journal of Texture Studies. 2020;51(2):343-51. | Title |
|  | Freeland TD. Muscle function during treatment with the functional regulator. Angle Orthodontist. 1979;49(4):247-58. | Title |
|  | Freund B, Schwartz M, Symington JM. The use of botulinum toxin for the treatment of temporomandibular disorders: Preliminary findings. Journal of Oral and Maxillofacial Surgery. 1999;57(8):916-20. | Title |
|  | Freund B, Schwartz M, Symington JM. Botulinum toxin: New treatment for temporomandibular disorders. British Journal of Oral and Maxillofacial Surgery. 2000;38(5):466-71. | Title |
|  | Frey M, Giovanoli P, John CH, Tzou J, Kropf N, Friedl S. Dynamic reconstruction of eye closure by muscle transposition or functional muscle transplantation in facial palsy. Plastic and Reconstructive Surgery. 2004;114(4):865-75. | Title |
|  | Fried JH, Sanzari JK, Cengel KA, Jensen J. Chronic mandibular deviation in a miniature swine model of simulated solar particle event radiation. Journal of the American Association for Laboratory Animal Science. 2012;51(5):661. | Title |
|  | Frongia G, Ramieri G, De Biase C, Bracco P, Piancino MG. Changes in electric activity of masseter and anterior temporalis muscles before and after orthognathic surgery in skeletal class III patients. Oral Surgery, Oral Medicine, Oral Pathology and Oral Radiology. 2013;116(4):398-401. | Title |
|  | Fu KY, Chen HM, Sun ZP, Zhang ZK, Ma XC. Long-term efficacy of botulinum toxin type A for the treatment of habitual dislocation of the temporomandibular joint. British Journal of Oral and Maxillofacial Surgery. 2010;48(4):281-4. | Title |
|  | Fuentes E, Frugone R, Paolinelli C, Hack GD, Bittner V. Electromyographic activity of the sphenomandibularis and lateral pterygoid human muscles during mandibular lateral movements. Chirurgia (Turin). 2012;25(2):97-9. | Title |
|  | Fujihara Y, Mori Y, Saijo H, Abe T, Susami T, Haga N, Hoshi K. Long-term dental outcomes in patients with fibrodysplasia ossificans progressiva: a report of three cases of tooth extraction. Quintessence International. 2022;53(8):712-20. | Title |
|  | Fujii A, Shinogaya T, Toda S, Hayakawa I. Quantification of oxidative metabolism in masseter muscle of denture wearers. Clin Oral Investig. 2005;9(3):173-9. | Title |
|  | Fujii K, Ishizaki A, Ogawa A, Asami T, Kwon H, Tanaka A, et al. Validity of using multi-frequency bioelectrical impedance analysis to measure skeletal muscle mass in preschool children. Journal of Physical Therapy Science. 2017;29(5):863-8. | Title |
|  | Fujimoto K, Sasaki A, Kawajiri A, Yamaguchi K, Oshima T, Togane Y, et al. Relation between masseter muscle volumes and mandibular body lengths in skeletal Class III adult female cases without severe mandibular asymmetry. Clinical and Investigative Orthodontics. 2022;81(3):144-52. | Full text  Only Class IIIs |
|  | Fujimura K, Segami N, Sato J, Kanayama K, Nishimura M, Demura N. Advantages of intraoral verticosagittal ramus osteotomy in skeletofacial deformity patients with temporomandibular joint disorders. Journal of Oral and Maxillofacial Surgery. 2004;62(10):1246-52. | Title |
|  | Fujioka M, Fujii T, Hirano A. Comparative study of mandibular stability after sagittal split osteotomies: Biocortical versus monocortical osteosynthesis. Cleft Palate-Craniofacial Journal. 2000;37(6):551-5. | Title |
|  | Fujishita A, Koga Y, Utsumi D, Nakamura A, Yoshimi T, Yoshida N. Effects of feeding a soft diet and subsequent rehabilitation on the development of the masticatory function. Journal of Oral Rehabilitation. 2015;42(4):266-74. | Title |
|  | Fujita T, Hayashi H, Shirakura M, Tsuka Y, Fujii E, Kawata T, et al. Regeneration of Condyle with a Functional Appliance. Journal of Dental Research. 2013;92(4):322-8. | Title |
|  | Fukuda M, Inoue K, Sakashita H. Periostitis Ossificans Arising in the Mandibular Bone of a Young Patient: Report of an Unusual Case and Review of the Literature. Journal of Oral and Maxillofacial Surgery. 2017;75(9):1834.e1-.e8. | Title |
|  | Fukui R, Yamamoto A, Tsunoda M, Matsumoto K, Namaki S, Asano M. Sclerosing odontogenic carcinoma with local recurrence and lymph node metastasis. Pathology. 2023;55(6):897-900. | Title |
|  | Fukumoto I, Kondo O. Three-dimensional craniofacial variation and occlusal wear severity among inhabitants of Hokkaido: comparisons of Okhotsk culture people and the Ainu. Anthropological Science. 2010;118(3):161-72. | Title |
|  | Fukumoto Y, Miyama T. Alleviation of masticatory disturbance with an occlusal splint in a Duchenne muscular dystrophy patient. Spec Care Dentist. 2021;41(5):572-8. | Title |
|  | Fukura M, Kashima K, Maeda S, Shiba R. Changes in bite force and muscle forces in the upper extremities after counter irritation. Cranio : the journal of craniomandibular practice. 2004;22(1):45‐9. | Title |
|  | Fukuta K, Jackson IT, Choi HY, Van Wyck LG. Measurement of masticatory muscles using 3DCT; Accuracy and application to hemifacial microsomia. Japanese Journal of Plastic and Reconstructive Surgery. 1991;34(3):231-8. | Title |
|  | Fukuyama E, Fujita Y, Soma K. Changes in jaw-jerk on different levels of jaw closure and teeth-clenching in humans. Journal of Oral Rehabilitation. 2000;27(11):967-77. | Title |
|  | Fulciniti F, Pia Curcio M, Liguori G, Aquino G, Botti G, Campanile AC, et al. Hyalinizing clear cell carcinoma of the parotid gland: Report of a recurrent case with aggressive cytomorphology and behavior diagnosed on fine-needle cytology sample. Diagnostic Cytopathology. 2014;42(1):63-8. | Title |
|  | Fulks BA, Callaghan KX, Tewksbury CD, Gerstner GE. Relationships between chewing rate, occlusion, cephalometric anatomy, muscle activity, and masticatory performance. Arch Oral Biol. 2017;83:161-8. | Abstract |
|  | Furtner J. Craniofacial musculature assessment: A novel technique for predicting stroke patient outcomes. European Journal of Radiology. 2023;167. | Title |
|  | Furuuchi T, Kochi S, Sasano T, Iikubo M, Komai S, Igari K. Morphologic characteristics of masseter muscle in cleidocranial dysplasia: a report of 3 cases. Oral Surg Oral Med Oral Pathol Oral Radiol Endod. 2005;99(2):185-90. | Title |
|  | Fusetti S, Ghirotto C, Ferronato G. A case of cephalic tetanus in a developed country. International Journal of Immunopathology and Pharmacology. 2013;26(1):273-7. | Title |
|  | Gaggl A, Bottini GB, Sagl B, Rasse M. Functional reconstruction of the masseter muscle by microvascular free gracilis muscle transfer: technique and outcome. International Journal of Oral and Maxillofacial Surgery. 2023. | Title |
|  | Gal TJ, Ridley MB, Arrington JA, Muro-Cacho C. Renal cell carcinoma presenting as a masseteric space mass. American Journal of Otolaryngology - Head and Neck Medicine and Surgery. 1997;18(4):280-2. | Title |
|  | Gałczyńska-Rusin M, Pobudek-Radzikowska M, Gawriołek K, Czajka-Jakubowska A. Gender-Related Biomechanical Properties of Masseter Muscle among Patients with Self-Assessment of Bruxism: A Comparative Study. Journal of Clinical Medicine. 2022;11(3). | Title |
|  | Gallo LM. Modeling of temporomandibular joint function using MRI and jaw-tracking technologies - Mechanics. Cells Tissues Organs. 2005;180(1):54-68. | Title |
|  | Gálvez-López E, Kilbourne B, Cox PG. Cranial shape variation in mink: Separating two highly similar species. J Anat. 2022;240(2):210-25. | Title |
|  | Gambareli FR, Serra MD, Pereira LJ, Gaviao MB. Influence of measurement technique, test food, teeth and muscle force interactions in masticatory performance. Journal of Texture Studies. 2007;38(1):2-20. | Title |
|  | Gambarota G, Cairns BE, Berde CB, Mulkern RV. Osmotic effects on the <i>T</i><sub>2</sub> relaxation decay of in vivo muscle. Magnetic Resonance in Medicine. 2001;46(3):592-9. | Title |
|  | Gamoh S, Akiyama H, Tsuji K, Nakazawa T, Morita S, Tanaka A, Shimizutani K. Non-contrast computed tomography and magnetic resonance imaging features of mucoepidermoid carcinoma in the salivary glands. Oral Radiology. 2018;34(1):24-30. | Title |
|  | Gan Y, Sasai T, Nishiyama H, Ma X, Zhang Z, Fuchihata H. Magnetic resonance imaging of human mandibular elevator muscles after repetitive maximal clenching exercise. Arch Oral Biol. 2000;45(3):247-51. | Abstract |
|  | Gao J, Li XJ, He J, Jiang LL, Zhao BH. The effect of mandibular flexure on the design of implant-supported fixed restorations of different facial types under two loading conditions by three-dimensional finite element analysis. Frontiers in Bioengineering and Biotechnology. 2022;10. | Title |
|  | Gaofeng L, Jun T, Bo P, Bosheng Z, Qian Z, Dongping L. Evaluation and selecting indications for the treatment of improving facial morphology by masseteric injection of botulinum toxin type A. Journal of Plastic, Reconstructive and Aesthetic Surgery. 2010;63(12):2026-31. | Title |
|  | García AEJ, Clari VR, Gallardo VP. Relationship between demographic and cephalometric measures and electromyographic activity of the facial musculature. A preliminary study in children and adolescents. Revista De Investigacion En Logopedia. 2022;12(1). | Full text  Other outcomes |
|  | García-García A, Gándara-Rey JM, Crespo-Abelleira A, Jorge-Barreiro J. Botulinum toxin A for treating muscular contractures in cephalic tetanus. British Journal of Oral and Maxillofacial Surgery. 2007;45(7):573-5. | Title |
|  | García-Morales P, Buschang PH, Throckmorton GS, English JD. Maximum bite force, muscle efficiency and mechanical advantage in children with vertical growth patterns. Eur J Orthod. 2003;25(3):265-72. | Abstract |
|  | Gardovska K, Urtane I, Krumina G. Musculomandibular morphology in individuals with different vertical skeletal growth patterns: an MRI and cone beam computed tomography study. Stomatologija. 2020;22(4):99-106. | Included |
|  | Garip H, Tufekcioglu S, Kaya E. Changes in the temporomandibular joint disc and temporal and masseter muscles secondary to bruxism in Turkish patients. Saudi Medical Journal. 2018;39(1):81-5. | Title |
|  | Garliner D. The importance of oro-facial muscle function and dysfunction in the treatment of various occlusal problems. Fortschritte der Kieferorthopädie. 1986;47(3):215-20. | Title |
|  | Garner LD, Kotwal NS. Correlation Study of Incisive Biting Forces with Age, Sex, and Anterior Occlusion. Journal of Dental Research. 1973;52(4):698-702. | Title |
|  | Garstka AA, Brzózka M, Bitenc-Jasiejko A, Ardan R, Gronwald H, Skomro P, Lietz-Kijak D. Cause-Effect Relationships between Painful TMD and Postural and Functional Changes in the Musculoskeletal System: A Preliminary Report. Pain Research and Management. 2022;2022. | Title |
|  | Gaszynska E, Kopacz K, Fronczek-Wojciechowska M, Padula G, Szatko F. Electromyographic activity of masticatory muscles in elderly women - a pilot study. Clin Interv Aging. 2017;12:111-6. | Abstract |
|  | Gaur V, Perumal SM, Rahmaan F, Pałka Ł. A practical approach to orofacial rehabilitation in a patient after inferior maxillectomy and rhinectomy with mono framework construction supported on a zygomatic implant placed in the glabella: a case report. Maxillofacial Plastic and Reconstructive Surgery. 2021;43(1). | Title |
|  | Gaur V, Singh N, Doshi AG, Chandrahas B. Immediate rehabilitation of a rheumatoid arthritis patient with single-piece implants. International Journal of Surgery Case Reports. 2021;82. | Title |
|  | Gazit-Rappaport T, Bayer A, Gazit E. An innovative orthodontic-prosthetic approach for a patient with dental and skeletal asymmetry. American Journal of Orthodontics and Dentofacial Orthopedics. 2003;123(2):185-91. | Title |
|  | Gedrange T, BÜTtner C, Schneider M, Lauer G, Mai R, Oppitz R, Harzer W. Change of mRNA amount of myosin heavy chain in masseter muscle after orthognathic surgery of patients with malocclusion. Journal of Cranio-Maxillofacial Surgery. 2006;34(SUPPL. 2):110-5. | Title |
|  | Gedrange T, Büttner C, Schneider M, Oppitz R, Harzer W. Myosin heavy chain protein and gene expression in the masseter muscle of adult patients with distal or mesial malocclusion. Journal of Applied Genetics. 2005;46(2):227-36. | Title |
|  | Gedrange T, Harzer W. Muscle influence on postnatal craniofacial development and diagnostics. Journal of Orofacial Orthopedics. 2004;65(6):451-66. | Abstract |
|  | Gedrange T, Hietschold V, Haase I, Haase J, Laniado M, Harzer W. Computed tomographic examination of muscle volume, cross-section and density in patients with dysgnathia. RoFo Fortschritte auf dem Gebiet der Rontgenstrahlen und der Bildgebenden Verfahren. 2005;177(2):204-9. | Included |
|  | Geers C, Nyssen-Behets C, Cosnard G, Lengelé B. The deep belly of the temporalis muscle: An anatomical, histological and MRI study. Surgical and Radiologic Anatomy. 2005;27(3):184-91. | Abstract |
|  | Geist JR, Chen FH. Nasopharyngeal carcinoma. Computed tomographic imaging of four cases. Oral Surg Oral Med Oral Pathol. 1993;75(6):759-66. | Title |
|  | Genc A, Isler SC, Keskin C, Oge AE, Matur Z. Prospective Analysis of the Swallowing Reflex After Sagittal Split Osteotomy: Comparison with Normal Volunteers. Dysphagia. 2020;35(5):798-805. | Title |
|  | Genovese FR. Implant prosthesis rehabilitation with scheme M-4 and its electromyographic analysis. Dental Cadmos. 2022;90(10):778-93. | Title |
|  | Gerlach HG. Asymmetrien im Kiefer-Gesichtsbereich. Fortschritte der Kieferorthopädie. 1968;29(3-4):436-532. | Title |
|  | Gerstner GE, Marchi F, Haerian H. Relationship between anteroposterior maxillomandibular morphology and masticatory jaw movement patterns. American Journal of Orthodontics and Dentofacial Orthopedics. 1999;115(3):258-66. | Title |
|  | Ghadimi N, Mehralizadeh S, Rahimian E, Hafezi L, Talaiepour A. Correlation Between the Masticatory Muscle Dimensions and Internal Derangement of Temporomandibular Joints Based on Magnetic Resonance Imaging. Iranian Journal of Radiology. 2023;20(1). | Title |
|  | Ghazzawi Z. Modelling of the craniofacial skeleton: An investigation of skull biomechanics [Dissertation/Thesis]2002. | Title |
|  | Gholami M, Anbiaee N, Bakhshi Moqaddam Firouz Abad S, Asadi M. What Are the Effects of Methylprednisolone Injection Into the Masseter and Gluteal Muscle on Pain, Edema and Trismus After Impacted Lower Third Molar Surgery? A Randomized Clinical Trial. Journal of Oral and Maxillofacial Surgery. 2021;79(9):1829-36. | Title |
|  | Ghousia S, Nyer Firdoose CS. Coronoid foramina in a pediatric mandible: An incidental finding of a morphologic and developmental anatomic variant as a distinctive documented feature. National Journal of Clinical Anatomy. 2021;10(1):51-4. | Title |
|  | Giannini L, Maspero C, Batia C, Galbiati G. Electromyographic and electrognatographic evaluation in orthodontic surgical treatment. Mondo Ortodontico. 2011;36(1):12-28. | Title |
|  | Gianniri AI, Melsen B, Nielsen L, Athanasiou AE. OCCLUSAL CONTACTS IN MAXIMUM INTERCUSPATION AND CRANIOMANDIBULAR DYSFUNCTION IN 16-YEAR-OLD TO 17-YEAR-OLD ADOLESCENTS. Journal of Oral Rehabilitation. 1991;18(1):49-59. | Title |
|  | Gibbs SJ. Comparative imaging of the jaws. Curr Opin Dent. 1992;2:55-63. | Title |
|  | Gilat H, Vainer I, Avishai G, Maymon SL, Alkan U, Hod R, et al. Radioiodine therapy induced sialadenitis versus chronic idiopathic sialadenitis—Presentation and outcomes. Head and Neck. 2021;43(9):2724-30. | Title |
|  | Gili T, Di Carlo G, Capuani S, Auconi P, Caldarelli G, Polimeni A. Complexity and data mining in dental research: A network medicine perspective on interceptive orthodontics. Orthodontics & Craniofacial Research. 2021;24:16-25. | Title |
|  | Ginszt M, Zieliński G, Szkutnik J, Wójcicki M, Baszczowski M, Litko-Rola M, et al. The Effects of Wearing a Medical Mask on the Masticatory and Neck Muscle Activity in Healthy Young Women. Journal of Clinical Medicine. 2022;11(2). | Title |
|  | Gionhaku N, Iinuma T, Izumi K, Shimizu M, Kawamura Y, Mamiya K, Moriya Y. Correlation between jaw muscle activity and bite force under the known different craniofacial form. Part 1. Classification of craniofacial form and its influence on jaw muscle activity and bite force. Nihon Hotetsu Shika Gakkai zasshi. 1988;32(6):1378-85. | Full text  Other outcomes |
|  | Gionhaku N, Lowe AA. Relationship between jaw muscle volume and craniofacial form. J Dent Res. 1989;68(5):805-9. | Full text  Sleep apnea &  normal occlusion males |
|  | Giovanini AF, Miranda K, Costa Vaz AF, Cavalcante RC, Corso P, Klüppel LE, Scariot R. Traumatic myositis chondro-ossificans of masseter muscle associated with TGF-β1, Indian Hegdehog, BMP2, osteopontin and osteocalcin upregulation: Case report. Indian J Dent Res. 2019;30(4):634-8. | Title |
|  | Gisel A. The influence of environment on growth and configuration of the human skull. Wiener Medizinische Wochenschrift. 1974;124(23):353-7. | Title |
|  | Giudice M, Piazza C, Bolzoni A, Peretti G. Head and neck intramuscular haemangioma: Report of two cases with unusual localization. European Archives of Oto-Rhino-Laryngology. 2003;260(9):498-501. | Title |
|  | Gjorup CA, Hendel HW, Svane IM, Hölmich LR. Isolated asymptomatic masseter muscle metastasis as first sign of metastatic disease in a patient with known melanoma. JPRAS Open. 2016;10:1-4. | Title |
|  | Gkantidis N, Halazonetis DJ. Morphological integration between the cranial base and the face in children and adults. Journal of Anatomy. 2011;218(4):426-38. | Abstract |
|  | Glerup M, Tagkli A, Küseler A, Christensen AE, Verna C, Bilgrau AE, et al. Incidence of Orofacial Manifestations of Juvenile Idiopathic Arthritis From Diagnosis to Adult Care Transition: A Population-Based Cohort Study. Arthritis and Rheumatology. 2023;75(9):1658-67. | Title |
|  | Glöggler JC, Hellmann D, Von Manstein M, Jäger R, Repky S, Beyersmann J, Lapatki BG. Motor learning might contribute to a therapeutic anterior shift of the habitual mandibular position—An exploratory study. Journal of Oral Rehabilitation. 2021;48(8):891-900. | Title |
|  | Glowacka H, Schwartz GT. A biomechanical perspective on variation in molar emergence ages in Primates. American Journal of Physical Anthropology. 2015;156:146. | Title |
|  | Glowacka H, Schwartz GT. Developmental coordination of the masticatory system constrains molar emergence across primates. American Journal of Physical Anthropology. 2016;159:156. | Title |
|  | Glowacka H, Schwartz GT. A biomechanical perspective on molar emergence and primate life history. Science Advances. 2021;7(41). | Title |
|  | Go JH. A case of soft tissue myoepithelial tumor arising in masticator space. Yonsei Medical Journal. 2005;46(5):710-4. | Title |
|  | Goddard G, Mauro G. Temporomandibular disorders, a review of current diagnosis and treatment. Dental Cadmos. 2018;86(5):364-75. | Title |
|  | Godinho RM, Fitton LC, Toro-Ibacache V, Stringer CB, Lacruz RS, Bromage TG, O'Higgins P. The biting performance of Homo sapiens and Homo heidelbergensis. J Hum Evol. 2018;118:56-71. | Title |
|  | Godse NR, Alsulaimani S, Sindwani R, Recinos PF. Endoscopic multiport resection of a transspatial, retromaxillary tumor. Clinical Neurology and Neurosurgery. 2023;231. | Title |
|  | Goh BT, Lee S, Tideman H, Stoelinga PJW. Mandibular reconstruction in adults: a review. International Journal of Oral and Maxillofacial Surgery. 2008;37(7):597-605. | Title |
|  | Gohil J, Rajasekar G, Shivhare P, Nair P, Abraham M. A rare case of an extensive multi-compartment epidermoid presenting with pure motor trigeminal neuropathy, case report and review of literature. Journal of Neurosciences in Rural Practice. 2019;10(2):364-6. | Title |
|  | Gokce HS, Gokce SM, Akin E, Bengi O. Effects of complete denture wearing on the head posture and posterior airway space: A cephalometric study. Journal of Dental Sciences. 2011;6(1):6-13. | Title |
|  | Gola R, Cheynet F, Guyot L, Richard O, Sauvant J. Complications of nasal obstruction in children. L' Orthodontie française. 2000;71(3):219-31. | Title |
|  | Golab A. MUSCLE FUNCTIONAL MRI - CAN IT BE USEFUL FOR PHYSIOTHERAPY? THE EXAMPLE OF CRANIOCERVICAL REGION. Acta Physica Polonica B. 2017;48(10):1731-6. | Title |
|  | Goldenberg DC, Alonso N, Goldenberg FC, Gebrin ES, Amaral TS, Scanavini MA, Ferreira MC. Using computed tomography to evaluate maxillary changes after surgically assisted rapid palatal expansion. Journal of Craniofacial Surgery. 2007;18(2):302-11. | Title |
|  | Gomes AC, Vitti M, Regalo SC, Semprini M, Siéssere S, Watanabe PC, Palomari ET. Evidence of muscle role over the cranio-facial skull development in Angle's Class III dental malocclusion under the clinical rest position. Electromyography and clinical neurophysiology. 2008;48(8):335‐41. | Full text  Other outcomes |
|  | Gomes AF, Nejaim Y, Brasil DM, Groppo FC, Caria PHF, Neto FH. Assessment of Volume and Height of the Coronoid Process in Patients With Different Facial Types and Skeletal Classes: A Cone-Beam Computed Tomography Study. Journal of Oral and Maxillofacial Surgery. 2015;73(7). | Title |
|  | Gomes SG, Custodio W, Faot F, Del Bel Cury AA, Garcia RC. Masticatory features, EMG activity and muscle effort of subjects with different facial patterns. J Oral Rehabil. 2010;37(11):813-9. | Full text  Other outcomes |
|  | Gomes SG, Custodio W, Jufer JSM, Del Bel Cury AA, Garcia R. Mastication, EMG Activity and Occlusal Contact Area in Subjects with Different Facial Types. Cranio-the Journal of Craniomandibular & Sleep Practice. 2010;28(4):274-9. | Full text  Other outcomes |
|  | Gomes SGF, Custodio W, Faot F, Del Bel Cury AA, Garcia RCMR. Chewing side, bite force symmetry, and occlusal contact area of subjects with different facial vertical patterns. Brazilian Oral Research. 2011;25(5):446-52. | Full text  Other outcomes |
|  | Gomez YPS, Rockenbach ND, de Moraes AB, Correa EC, da Silva AMT, Busanello-Stella AR. Influence of Breathing Modes and Facial Growth Patterns on Electromyographic Fatigue of Masticatory Muscles in Children. International Archives of Otorhinolaryngology. 2023;27(04):672-9. | Title |
|  | Gomez-Gil DF. Hard tissue changes during the consolidation period after vertical mandibular ramus distraction. A canine pilot study [Dissertation/Thesis]2004. | Title |
|  | Gonçalves FM, Taveira KVM, de Araujo CM, Ravazzi GMNC, Guariza Filho O, Zeigelboim BS, et al. Association between atypical swallowing and malocclusions: a systematic review. Dental Press Journal of Orthodontics. 2022;27(6). | Title |
|  | Gonçalves RCG, Rabelo NN, Figueiredo EG, Welling LC. Oral health and temporal muscle thickness. Surgical Neurology International. 2021;12. | Title |
|  | Gonorazky HD, Dowling JJ, Volpatti JR, Vajsar J. Signs and Symptoms in Congenital Myopathies. Seminars in Pediatric Neurology. 2019;29:3-11. | Title |
|  | González-Fernández M, Perez-Nogueras J, Serrano-Oliver A, Torres-Anoro E, Sanz-Arque A, Arbones-Mainar JM, Sanz-Paris A. Masseter muscle thickness measured by ultrasound as a possible link with sarcopenia, malnutrition and dependence in nursing homes. Diagnostics. 2021;11(9). | Title |
|  | González-García R. Arthroscopic Myotomy of the Lateral Pterygoid Muscle With Coblation for the Treatment of Temporomandibular Joint Anterior Disc Displacement Without Reduction. Journal of Oral and Maxillofacial Surgery. 2009;67(12):2699-701. | Title |
|  | Gonzalez-Perez LM, Vera-Martin R, Montes-Latorre E, Torres-Carranza E, Infante-Cossio P. Botulinum Toxin and Percutaneous Needle Electrolysis for the Treatment of Chronic Masticatory Myalgia. Toxins. 2023;15(4). | Title |
|  | Gordon JS, Mandel L. Masseteric intramuscular hemangioma: Case report. Journal of Oral and Maxillofacial Surgery. 2014;72(11):2192-6. | Title |
|  | Goto S, Fujita Y, Hotta M, Sugiyama A, Maki K. Influence of differences in the hardness and calcium content of diets on the growth of craniofacial bone in rats. Angle Orthodontist. 2015;85(6):969-79. | Title |
|  | Goto TK, Langenbach GEJ. Condylar process contributes to mandibular asymmetry: In vivo 3D MRI study. Clinical Anatomy. 2014;27(4):585-91. | Title |
|  | Goto TK, Langenbach GEJ, Hannam AG. Length changes in the human masseter muscle after jaw movement. Anatomical Record. 2001;262(3):293-300. | Title |
|  | Goto TK, Nishida S, Yahagi M, Langenbach GEJ, Nakamura Y, Tokumori K, et al. Size and orientation of masticatory muscles in patients with mandibular laterognathism. Journal of Dental Research. 2006;85(6):552-6. | Full text  Only Md laterognathism |
|  | Goto TK, Tokumori K, Nakamura Y, Yahagi M, Yuasa K, Okamura K, Kanda S. Volume changes in human masticatory muscles between jaw closing and opening. Journal of Dental Research. 2002;81(6):428-32. | Abstract |
|  | Goto TK, Yamada T, Yoshiura K. Occlusal pressure, contact area, force and the correlation with the morphology of the jaw-closing muscles in patients with skeletal mandibular asymmetry. Journal of Oral Rehabilitation. 2008;35(8):594-603. | Abstract |
|  | Graber TM. Maxillary second molar extraction in Class II malocclusion. American Journal of Orthodontics. 1969;56(4):331-53. | Title |
|  | Grabowski R, Kundt G, Stahl F. Interrelation between occlusal findings and orofacial myofunctional status in primary and mixed dentition: Part III: Interrelation between malocclusions and orofacial dysfunctions. Journal of Orofacial Orthopedics. 2007;68(6):462-76. | Abstract |
|  | Gradl J, Höreth M, Pfefferle T, Prager M, Hilgenfeld T, Gareis D, et al. Application of a Dedicated Surface Coil in Dental MRI Provides Superior Image Quality in Comparison with a Standard Coil. Clinical Neuroradiology. 2017;27(3):371-8. | Title |
|  | Graham AJ, McLoughlin P. INTERESTING CASE: Maxillary swing approach for resection of a large pleomorphic adenoma arising in the soft palate. British Journal of Oral and Maxillofacial Surgery. 2006;44(4):307. | Title |
|  | Granatosky MC, Ross CF. Differences in muscle mechanics underlie divergent optimality criteria between feeding and locomotor systems. Journal of Anatomy. 2020;237(6):1072-86. | Title |
|  | Granger MW, Buschang PH, Throckmorton GS, Iannaccone ST. Masticatory muscle function in patients with spinal muscular atrophy. American Journal of Orthodontics and Dentofacial Orthopedics. 1999;115(6):697-702. | Title |
|  | Granite EL, Ramzy JI, Gillis JM. Metastatic Uterine Cervical Carcinoma of Zygoma: A Case Report and Review of the Literature. Journal of Oral and Maxillofacial Surgery. 2006;64(11):1669-71. | Title |
|  | Gray H. A Fiber Type Assessment of Masseter Muscles in Five Asymmetry Classifications [Dissertation/Thesis]2019. | Title |
|  | Gregor C, Hietschold V, Harzer W. A 31P-magnet resonance spectroscopy study on the metabolism of human masseter in individuals with different vertical facial pattern. Oral Surgery, Oral Medicine, Oral Pathology and Oral Radiology. 2013;115(3):406-14. | Included |
|  | Grossi GB, Garagiola U, Santoro F. Measuring effectiveness of orthognathic surgery by electromyography: A restrospective clinical study. Minerva Stomatologica. 2017;66(3):98-106. | Title |
|  | Grunheid T, Langenbach GEJ, Korfage JAM, Zentner A, Van Eijden TMGJ. The adaptive response of jaw muscles to varying functional demands. European Journal of Orthodontics. 2009;31(6):596-612. | Title |
|  | Grygus I, Kostyshyn A, Ilnytska O, Ornat H. THE CONDITION OF THE MASTICATORY MUSCLE GROUP IN THE TREATMENT OF PATIENTS WITH REDUCED OCCLUSION HEIGHT. Health Problems of Civilization. 2022;16(2):164-72. | Title |
|  | Guarda-Nardini L, Concheri G, Ferronato G, Manfredini D. Spring-bite: a new device for jaw motion rehabilitation. A case report. Stomatologija / issued by public institution "Odontologijos studija" [et al]. 2013;15(2):54-7. | Title |
|  | Guastafierro S, Falcone U, Petriccione L, Rossiello L, Cappabianca S, Rossiello R, Colella G. An unusual cause of facial swelling: Primary extranodal non-hodgkin lymphoma of the masseter muscle. American Journal of the Medical Sciences. 2011;341(2):160-2. | Title |
|  | Gudelj S, Belušić-Gobić M. CHALLENGES IN THE DIAGNOSIS OF MANDIBLE METASTASIS: A CASE REPORT. Acta Stomatologica Croatica. 2022;56(4):425. | Title |
|  | Guerrero CA, Gonzalez M, Throndson RR. Multiple zygoma implants for severe maxillary atrophy. International Journal of Oral and Maxillofacial Surgery. 2017;46:22. | Title |
|  | Guerreschi P, Gahagnon T, Vacher C, Drizenko A, Francke JP, Labbe D. Masseter muscle termination over the deep surface of the temporal fascia: Look out the wrong path. Surgical and Radiologic Anatomy. 2011;33(10):863-8. | Title |
|  | Gugino CF, Dus I. Unlocking orthodontic malocclusions: an interplay between form and function. Seminars in orthodontics. 1998;4(4):246-55. | Title |
|  | Gui H, Yang H, Shen SGF, Xu B, Zhang S, Bautista JS. Image-guided surgical navigation for removal of foreign bodies in the deep maxillofacial region. Journal of Oral and Maxillofacial Surgery. 2013;71(9):1563-71. | Title |
|  | Guimaraes TB, Ferreira MB, Wakamatsu A, Oliveira SR, Guimaraes AS, Galdames IS, Marie SN. Muscle Fiber Type Composition, Fiber Diameter, Capillary Density in Temporalis and Masseter Muscles and Correlation with Bite Force. International Journal of Morphology. 2013;31(2):747-53. | Title |
|  | Gülekon N, Peker T, Turgut HB, Anil A, Karaköse M. Qualitative comparison of anatomical microdissection, Sihler's staining and computerized reconstruction methods for visualizing intramuscular nerve branches. Surg Radiol Anat. 2007;29(5):373-8. | Title |
|  | Güler N, Yatmaz PI, Ataoglu H, Emlik D, Uckan S. Temporomandibular internal derangement: Correlation of MRI findings with clinical symptoms of pain and joint sounds in patients with bruxing behaviour. Dentomaxillofacial Radiology. 2003;32(5):304-10. | Title |
|  | Güler N, Yumuk PF, Ilguy D, Olgac V, Greer J. Limited Painful Mouth Opening. Journal of Oral and Maxillofacial Surgery. 2005;63(8):1201-5. | Title |
|  | Gundlach KKH. Ankylosis of the temporomandibular joint. Journal of Cranio-Maxillofacial Surgery. 2010;38(2):122-30. | Title |
|  | Gupta I. Therapeutic and Cosmetic Correction of Massetric Hypertrohy with Botulinum Toxin Injection and Assessment of Structural and Functional Changes in the Masseter Muscle Following the Treatment [Dissertation/Thesis]2018. | Title |
|  | Gupta R, Markowitz Y, Berman L, Chapman P. High-resolution imaging of an ancient Egyptian mummified head: New insights into the mummification process. American Journal of Neuroradiology. 2008;29(4):705-13. | Title |
|  | Gupta T, Gupta P, Duggal S, Yadav V. Mechanical analysis of the effects of implant position and abutment height on implant-assisted removable partial dentures. Journal of Population Therapeutics and Clinical Pharmacology. 2023;30(17):1016-24. | Title |
|  | Guruprasad R, Rishi S, Nair PP, Thomas S. Masseter and medial pterygoid muscle hypertrophy. BMJ Case Reports. 2011. | Title |
|  | Güzel MZ, Arslan H, Kiliç A. Bilateral congenital trigeminal sensorimotor neuropathy presents as a severe open-bite malocclusion. Journal of Craniofacial Surgery. 2006;17(5):998-1001. | Title |
|  | Habibi HA, Ozturk M, Caliskan E, Turan M. Quantitative assessment of temporomandibular disc and masseter muscle with shear wave elastography. Oral Radiology. 2022;38(1):49-56. | Title |
|  | Haiya Z, Zhou H, Qing Z. Treatment of a patient with considerably thin alveolar bone and severe open bite. Hua xi kou qiang yi xue za zhi = Huaxi kouqiang yixue zazhi = West China journal of stomatology. 2018;36(1):109-14. | Title |
|  | Hakiemtawfieq A. The Relation between Dentofacial Shapes and Depth of Masseter Muscle Using Magnetic Resonance Imaging Technique. Pakistan Journal of Medical and Health Sciences. 2022;16(12):24-6. | Full text  Other outcomes |
|  | Hakim SG, Wolf M, Wendlandt R, Kimmerle H, Sieg P, Jacobsen HC. Comparative biomechanical study on three miniplates osteosynthesis systems for stabilisation of low condylar fractures of the mandible. British Journal of Oral and Maxillofacial Surgery. 2014;52(4):317-22. | Title |
|  | Halazonetis DJ. Morphometric correlation between facial soft-tissue profile shape and skeletal pattern in children and adolescents. American Journal of Orthodontics and Dentofacial Orthopedics. 2007;132(4):450-7. | Title |
|  | Ham JW. Masseter Muscle Reduction Procedure With Radiofrequency Coagulation. Journal of Oral and Maxillofacial Surgery. 2009;67(2):457-63. | Title |
|  | Hamerling J. Dissertations 25 years after date 19. Children with a lateral forced bite. Nederlands tijdschrift voor tandheelkunde. 2009;116(3):145-8. | Title |
|  | Han MD, Obrez A, Sahni J, Greene CS. Acute anterior open bite: Case report and review of the literature. Oral and Maxillofacial Surgery Cases. 2019;5(4). | Title |
|  | Han MJ, Seo YK, Yoon HH, Song KY, Park JK. Upregulation of bone-like extracellular matrix expression in human dental pulp stem cells by mechanical strain. Biotechnology and Bioprocess Engineering. 2010;15(4):572-9. | Title |
|  | Han W, Xia W, Zhang Z, Kim BS, Chen X, Yan Y, et al. Radiomics and Artificial Intelligence Study of Masseter Muscle Segmentation in Patients With Hemifacial Microsomia. J Craniofac Surg. 2023;34(2):809-12. | Title |
|  | Han W, Yang X, Chen X, Mooi W, Aung ZM, Sun M, et al. Quantitative description of masseter muscle involvement in craniofacial microsomia. International Journal of Oral and Maxillofacial Surgery. 2021;50(10):1312-9. | Title |
|  | Hanke BA, Motschall E, Türp JC. Association between orthopedic and dental findings: What level of evidence is available? Journal of Orofacial Orthopedics. 2007;68(2):91-107. | Title |
|  | Hannam AG. Current computational modelling trends in craniomandibular biomechanics and their clinical implications. Journal of Oral Rehabilitation. 2011;38(3):217-34. | Title |
|  | Hannam AG, Wood WW. Relationships between the size and spatial morphology of human masseter and medial pterygoid muscles, the craniofacial skeleton, and jaw biomechanics. American Journal of Physical Anthropology. 1989;80(4):429-45. | Full text  No craniofacial patterns |
|  | Hansdottir R, Bakke M. Joint tenderness, jaw opening, chewing velocity, and bite force in patients with temporomandibular joint pain and matched healthy control subjects. Journal of Orofacial Pain. 2004;18(2):108-13. | Title |
|  | Hara K, Namiki C, Yamaguchi K, Kobayashi K, Saito T, Nakagawa K, et al. Association between myotonometric measurement of masseter muscle stiffness and maximum bite force in healthy elders. Journal of Oral Rehabilitation. 2020;47(6):750-6. | Full text  Other outcomes |
|  | Harada K, Watanabe M, Ohkura K, Enomoto S. Measure of bite force and. occlusal contact area before and. after bilateral sagittal split ramus osteotomy of the mandible using a new pressure-sensitive device: A preliminary report. Journal of Oral and Maxillofacial Surgery. 2000;58(4):370-3. | Title |
|  | Harila-Kaera V, Grön M, Heikkinen T, Alvesalo L. Sagittal occlusal relationships and asymmetry in prematurely born children. European Journal of Orthodontics. 2002;24(6):615-25. | Title |
|  | Harper RP, De Bruin H, Burcea I. Muscle activity during mandibular movements in normal and mandibular retrognathic subjects. Journal of Oral and Maxillofacial Surgery. 1997;55(3):225-33. | Full text  Other outcomes |
|  | Harper RP, de Bruin H, Burcea I, Engl B. Lateral pterygoid muscle activity in mandibular retrognathism and response to mandibular advancement surgery. American Journal of Orthodontics and Dentofacial Orthopedics. 1987;91(1):70-6. | Title |
|  | Harrison NL, Santoro G, Ellerby N, Samad A. Small bowel obstruction secondary to phytobezoar in a patient with myotonic dystrophy. BMJ Case Reports. 2023;16(10). | Title |
|  | Hart B, Schwartz HC. Cavernous hemangioma of the masseter muscle: Report of a case. Journal of Oral and Maxillofacial Surgery. 1995;53(4):467-9. | Title |
|  | Haruki T, Kanomi R, Morita H, Kawabata J. Oral morphology and tongue habits. The International journal of orofacial myology : official publication of the International Association of Orofacial Myology. 1995;21:4-8. | Title |
|  | Harzer W, Augstein A, Juenger D, Keil C, Weiland B. Notch expression profile and satellite cell stimulation in masseter muscle before and after orthognathic surgery. Journal of Cranio-Maxillofacial Surgery. 2021;49(2):93-7. | Title |
|  | Harzer W, Augstein A, Olbert C, Juenger D, Keil C, Weiland B. Satellite cell capacity for functional adaptation of masseter muscle in Class II and Class III patients after orthognathic surgery-a pilot study. European Journal of Orthodontics. 2021;43(2):234-40. | Title |
|  | Harzer W, Worm M, Gedrange T, Schneider M, Wolf P. Myosin heavy chain mRNA isoforms in masseter muscle before and after orthognathic surgery. Oral Surg Oral Med Oral Pathol Oral Radiol Endod. 2007;104(4):486-90. | Title |
|  | Hasan NMA, Abdelrahman TEF. MRI evaluation of TMJ internal derangement: Degree of anterior disc displacement correlated with other TMJ soft tissue and osseous abnormalities. Egyptian Journal of Radiology and Nuclear Medicine. 2014;45(3):735-44. | Title |
|  | Hasegawa S, Sasaki J, Nakao H, Tomimatsu M, Yamamoto S, Watanabe S, et al. Impact of the lateral skeletal stability following bilateral sagittal split ramus osteotomy for mandibular asymmetry. JPRAS Open. 2023;38:36-47. | Title |
|  | Hasegawa T, Shibuya Y, Kuroki S, Takeuchi J, Yokoo S, Umeda M, Komori T. Two cases of masticator space abcess initially diagnosed as temporomandibular joint disorder. Kobe Journal of Medical Sciences. 2008;54(3):E163-E8. | Title |
|  | Hasegawa Y, Tsuji S, Nagai K, Sakuramoto-Sadakane A, Tamaoka J, Oshitani M, et al. The relationship between bone density and the oral function in older adults: a cross-sectional observational study. BMC Geriatr. 2021;21(1):591. | Title |
|  | Hashimoto T, Kuroda S, Kamioka H, Mishima K, Sugahara T, Takano-Yamamoto T. Bimaxillary protrusion with masseter muscle hypertrophy treated with titanium screw anchorage and masseter surgical reduction. American Journal of Orthodontics and Dentofacial Orthopedics. 2009;135(4):536-48. | Title |
|  | Haskell B, Day M, Tetz J. Computer-aided modeling in the assessment of the biomechanical determinants of diverse skeletal patterns. Am J Orthod. 1986;89(5):363-82. | Abstract |
|  | Haskell B, Farman AG, Wagner G, Day M. Magnetic resonance: an imaging adjunct for orthognathic evaluation? The International journal of adult orthodontics and orthognathic surgery. 1987;2(3):165-73. | Title |
|  | Haskova J, Maini S, Meehan C. Melanoma in the masseter muscle. Journal of Laryngology and Otology. 2004;118(12):985-7. | Title |
|  | Haspel AC, Coviello VF, Stevens M, Robinson PG. Myofibroma of the mandible in an infant: Case Report, Review of the Literature, and Discussion. Journal of Oral and Maxillofacial Surgery. 2012;70(7):1599-604. | Title |
|  | Hassan MG, Kaler H, Zhang B, Cox TC, Young N, Jheon AH. Effects of Multi-Generational Soft Diet Consumption on Mouse Craniofacial Morphology. Frontiers in Physiology. 2020;11. | Title |
|  | Hatcher DC. Anatomy of the Mandible, Temporomandibular Joint, and Dentition. Neuroimaging Clinics of North America. 2022;32(4):749-61. | Title |
|  | Hatef B, Talebain S, Oliyae GR, Bagheri H. Effect of tempromandibular joint sounds on timing of the masseter muscle activity in the open-close-clench cycle. Journal of Medical Sciences. 2007;7(3):339-46. | Title |
|  | Hatta K, Ikebe K. Association between oral health and sarcopenia: A literature review. J Prosthodont Res. 2021;65(2):131-6. | Title |
|  | Haugaard CF, Agander TK, Lelkaitis G, Andersen KF, Andreasen S, Wessel I. Salivary gland carcinomas with unusual presentations. Acta Oncologica. 2019;58(3):382-4. | Title |
|  | He D, Yang C, Chen M, Yang X, Li L, Jiang Q. Surgical treatment of traumatic temporomandibular joint ankylosis with medially displaced residual condyle: Surgical methods and long-term results. Journal of Oral and Maxillofacial Surgery. 2011;69(9):2412-8. | Title |
|  | He D, Yang C, Chen M, Zhang X, Qiu Y, Yang X, et al. Traumatic temporomandibular joint ankylosis: Our classification and treatment experience. Journal of Oral and Maxillofacial Surgery. 2011;69(6):1600-7. | Title |
|  | He T, Stavropoulos D, Hagberg C, Hakeberg M, Mohlin B. Effects of masticatory muscle training on maximum bite force and muscular endurance. Acta odontologica Scandinavica. 2013;71(3‐4):863‐9. | Title |
|  | He TL, Kiliaridis S. Effects of masticatory muscle function on craniofacial morphology in growing ferrets (<i>Mustela putorius furo</i>). European Journal of Oral Sciences. 2003;111(6):510-7. | Title |
|  | Heggie AAC. Skeletal management of craniofacial microsomia. International Journal of Oral and Maxillofacial Surgery. 2015;44:e6-e7. | Title |
|  | Helland SB, Pedersen TØ. Traumatic myositis ossificans of the temporal muscle after dental local anesthesia. Clinical Case Reports. 2023;11(6). | Title |
|  | Hellmann D, Giannakopoulos NN, Blaser R, Eberhard L, Rues S, Schindler HJ. Long-term training effects on masticatory muscles. J Oral Rehabil. 2011;38(12):912-20. | Title |
|  | Hellmann D, Stein T, Potthast W, Rammelsberg P, Schindler HJ, Ringhof S. The effect of force-controlled biting on human posture control. Human Movement Science. 2015;43:125-37. | Title |
|  | Hemphill SB. The Effect of Distal Movement of the Maxillary Dentition on Hyperdivergent and Hypodivergent Individuals [Dissertation/Thesis]2017. | Title |
|  | Henderson LA, Akhter R, Youssef AM, Reeves JM, Peck CC, Murray GM, Svensson P. The effects of catastrophizing on central motor activity. European Journal of Pain (United Kingdom). 2016;20(4):639-51. | Title |
|  | Henderson SE, Desai R, Tashman S, Almarza AJ. Functional analysis of the rabbit temporomandibular joint using dynamic biplane imaging. Journal of Biomechanics. 2014;47(6):1360-7. | Title |
|  | Hendrickx K, Mommaerts MY, Jacobs W, Abeloos JVS, Neyt LF, De Clercq CAS. Proximal segment position after distraction with the MD-DOS device. Journal of Cranio-Maxillofacial Surgery. 1999;27(6):383-6. | Title |
|  | Hennessy J, Ethunandan M. Perineural invasion in malignant melanoma. British Journal of Oral and Maxillofacial Surgery. 2012;50(4):378. | Title |
|  | Herlofson BB, Wranicz P. Severe progression of trismus during 15 years of suffering from adenoid cystic carcinoma in the retro molar area. Supportive Care in Cancer. 2011;19(2):S359. | Title |
|  | Hermans R. Neoplasms of the Oropharynx. 2021. p. 173-90. | Title |
|  | Herrel A, Aerts P, Fret J, de Vree F. Morphology of the feeding system in agamid lizards: Ecological correlates. Anatomical Record. 1999;254(4):496-507. | Title |
|  | Herrel A, Fabre AC, Hugot JP, Keovichit K, Adriaens D, Brabant L, et al. Ontogeny of the cranial system in Laonastes aenigmamus. Journal of Anatomy. 2012;221(2):128-37. | Title |
|  | Herring SW, Liu ZJ, Rafferty KL, Baldwin MC, Salamati A, Cunningham C, et al. Repeated botulinum treatment of rabbit masseter causes cumulative tissue damage. Archives of Oral Biology. 2022;141. | Title |
|  | Hichijo N, Kawai N, Mori H, Sano R, Ohnuki Y, Okumura S, et al. Effects of the masticatory demand on the rat mandibular development. Journal of Oral Rehabilitation. 2014;41(8):581-7. | Title |
|  | Hichijo N, Tanaka E, Kawai N, van Ruijven LJ, Langenbach GEJ. Effects of Decreased Occlusal Loading during Growth on the Mandibular Bone Characteristics. Plos One. 2015;10(6). | Abstract |
|  | Hidaka H, Ishida E, Suzuki T, Matsutani S, Kobayashi T, Takahashi S. Unusual parapharyngeal extension of peritonsillar abscess to the masticator space: Successfully drained by extraoral and intraoral endoscopic approaches. Annals of Otology, Rhinology and Laryngology. 2014;123(5):333-7. | Title |
|  | Higashino R. [Relationship between jaws and the masseter muscle by superimposing MR images on the cephalogram]. Kokubyo Gakkai Zasshi. 2006;73(1):116-24. | Included |
|  | Higley MJ, Walkiewicz TW, Miller JH, Curran JG, Towbin RB. Aplasia of the parotid glands with accessory parotid tissue. Pediatric Radiology. 2010;40(3):345-7. | Title |
|  | Hillam RA, Goodship AE, Skerry TM. Peak strain magnitudes and rates in the tibia exceed greatly those in the skull: An in vivo study in a human subject. Journal of Biomechanics. 2015;48(12):3292-8. | Title |
|  | Hilloowala R, Trent R, Dal Pozzo G. Role of the mandibular first molar in the development of the supraorbital region in apes and humans. Anthropologischer Anzeiger; Bericht über die biologisch-anthropologische Literatur. 1993;51(3):275-82. | Title |
|  | Hînganu D, Stan CI, Ciupilan C, Hînganu MV. Anatomical considerations on the masseteric fascia and superficial muscular aponeurotic system. Rom J Morphol Embryol. 2018;59(2):513-6. | Title |
|  | Hinotume S, Morinushi T, Ogura T. Masticatory function in normal and crowded occlusion using Hellman's dental stages. The Journal of clinical pediatric dentistry. 1994;18(4):267-82. | Abstract |
|  | Hirose K. [The study of the relationships between the masticatory muscles activity and the craniofacial morphology in mandibular prognathism]. Shigaku. 1990;78(1):49-62. | Abstract |
|  | Hiyama S. [An electromyographic study on functional adaptations--associated with herbst appliance]. Kokubyo Gakkai Zasshi. 1996;63(1):18-30. | Title |
|  | Hiyama S, Asakawa S, Ono T, Mochida-Matsubara M, Ohyama K. Evaluation of stomatognathic function in orthodontic treatment. World journal of orthodontics. 2005;6(4):343-54. | Title |
|  | Hiyama S, Ono PT, Ishiwata Y, Kuroda T, McNamara JA, Jr. Neuromuscular and skeletal adaptations following mandibular forward positioning induced by the Herbst appliance. Angle Orthod. 2000;70(6):442-53. | Title |
|  | Hoard MA, Tadje JP, Gampper TJ, Edlich RF. Traumatic chronic TMJ dislocation: Report of an unusual case and discussion of management. Journal of Cranio-Maxillofacial Trauma. 1998;4(4):44-7. | Title |
|  | Hoffman EA, Rowe TB. Jurassic stem-mammal perinates and the origin of mammalian reproduction and growth. Nature. 2018;561(7721):104-8. | Title |
|  | Hogenbirk RNM, Banning LBD, Visser A, Jager-Wittenaar H, Pol RA, Zeebregts CJ, Klaase JM. Article Association between Masseter Muscle Area and Thickness and Outcome after Carotid Endarterectomy: A Retrospective Cohort Study. Journal of Clinical Medicine. 2022;11(11). | Title |
|  | Hogg RT, Ravosa MJ, Ryan TM, Vinyard CJ. The Functional Morphology of the Anterior Masticatory Apparatus in Tree-Gouging Marmosets (Cebidae, Primates). Journal of Morphology. 2011;272(7):833-49. | Title |
|  | Holmlund A, Lund B, Weiner CK. Mandibular condylectomy with osteoarthrectomy with and without transfer of the temporalis muscle. British journal of oral & maxillofacial surgery. 2013;51(3):206‐10. | Title |
|  | Holton NE. Modeling of masticatory biomechanics in living humans as a baseline for testing functional hypotheses in Neandertals [Dissertation/Thesis]2009. | Title |
|  | Holton NE, Franciscus RG, Ravosa MJ, Southard TE. Functional and Morphological Correlates of Mandibular Symphyseal Form in a Living Human Sample. American Journal of Physical Anthropology. 2014;153(3):387-96. | Title |
|  | Homma A, Saheki M, Suzuki F, Fukuda S. Computer image-guided surgery for total maxillectomy. European Archives of Oto-Rhino-Laryngology. 2008;265(12):1521-6. | Title |
|  | Hong B, Petrosyan V, Kruger AR. Unusual presentation of Epstein-Barr virus-positive diffuse large B-cell non-Hodgkin lymphoma of the elderly. Journal of Oral and Maxillofacial Surgery. 2016;74(6):1180e1-e7. | Title |
|  | Hong G, Wang Q, Chu J, Jiang L, Li Z, He S, et al. The value of magnetic resonance imaging of the mandibular nerve using a micro surface coil and three-dimensional double-echo steady-state with water excitation sequence. Chinese Journal of Radiology (China). 2018;52(6):421-5. | Title |
|  | Hong H, Zeng Y, Chen X, Peng C, Deng J, Zhang X, et al. Electromyographic features and efficacy of orofacial myofunctional treatment for skeletal anterior open bite in adolescents: an exploratory study. BMC Oral Health. 2021;21(1). | Title |
|  | Hong HJ, Hong JW, Koh SH, Kim YO, Park BY. A three-dimensional analysis of the relationship among lower facial width, bony width, and masseter muscle volume in subjects with prominent mandible angles. Journal of Craniofacial Surgery. 2009;20(4):1114-9. | Full text  No skeletal patterns |
|  | Hong SO. Cosmetic Treatment Using Botulinum Toxin in the Oral and Maxillofacial Area: A Narrative Review of Esthetic Techniques. Toxins. 2023;15(2). | Title |
|  | Hong SW, Huh KH, Lee JK, Kang JH. Craniofacial anomalies associated with spondyloenchondrodysplasia: Two case reports. Medicine (United States). 2018;97(50). | Title |
|  | Hönicke K, Harzer W, Eckardt L. Correlation between EMG pattern of masseter muscle and facial bone morphology. Fortschritte der Kieferorthopädie. 1995;56(5):237-44. | Abstract |
|  | Hou SY, Peng SS, Dai HW, Song JL, Xu L, Zhou JP, Li LJ. Mechanical loading and autophagy: A study on the BoNT-A injection-induced condylar cartilage degeneration. Archives of Biochemistry and Biophysics. 2023;749. | Title |
|  | Hsu HC, Huang EY, Eng HL. Cheek mass as a presentation of metastatic rectal cancer. Chang Gung Med J. 2002;25(5):345-8. | Title |
|  | Hsu HY, Yamaguchi K. Decreased chewing activity during mouth breathing. Journal of Oral Rehabilitation. 2012;39(8):559-67. | Title |
|  | Hu HL. The Influences on the Condyle and Masticatory Muscles and Ligament by Herbst Appliance --Three Dimensional Arstotropic Finite Element Method. 2001. | Title |
|  | Hu S, Shao Z, Deng L. Clinical Manifestations, Imaging Features, and Pathogenic/Prognostic Risk Factors for Temporomandibular Disorders (TMD): A Case-Control Study Based on Psychogenic Factors of Patients. Computational and Mathematical Methods in Medicine. 2022;2022. | Title |
|  | Huang B, Takahashi K, Jennings EA, Pumtang-On P, Kiso H, Togo Y, et al. Prospective signs of cleidocranial dysplasia in Cebpb deficiency. Journal of Biomedical Science. 2014;21(1). | Title |
|  | Huang HL, Su KC, Fuh LJ, Chen MYC, Wu J, Tsai MT, Hsu JT. Biomechanical analysis of a temporomandibular joint condylar prosthesis during various clenching tasks. Journal of Cranio-Maxillofacial Surgery. 2015;43(7):1194-201. | Title |
|  | Huang IY, Chen CM, Chang SW, Yang CF, Chen CH, Chen CM. Surgical management of accidentally displaced mandibular third molar into the pterygomandibular space: A case report. Kaohsiung Journal of Medical Sciences. 2007;23(7):370-4. | Title |
|  | Huang MY, Matsuura N, Kaneko Y, Ichinohe T. Midazolam increases bite force during intravenous sedation. Journal of Oral and Maxillofacial Surgery. 2012;70(8):e458-e63. | Title |
|  | Huang XY, Hu XN, Zhao YJ, Wang Y, Gu Y. Preliminary comparison of three-dimensional reconstructed palatal morphology in subjects with different sagittal and vertical patterns. Bmc Oral Health. 2020;20(1). | Title |
|  | Huang YL, Yang LY, Lin YX, Mu L, Zhao MH, Ma XY, Teng L. Relationship Between Masticatory Muscle Size and Bone Regeneration After Mandibular Angle Osteotomy. Journal of Craniofacial Surgery. 2021;32(8):2784-7. | Title |
|  | Huang ZL, Xue ZQ, Gu Y. Logistic regression analysis of the relationship between abnormal dental occlusion and temporomandibular disorders in lower grade college students. Journal of Prevention and Treatment for Stomatological Diseases. 2021;29(1):45-9. | Title |
|  | Huggare JA, Raustia AM. Head posture and cervicovertebral and craniofacial morphology in patients with craniomandibular dysfunction. Cranio : the journal of craniomandibular practice. 1992;10(3):173-7;discussion8. | Title |
|  | Huh A, Horton MJ, Cuenco KT, Raoul G, Rowlerson AM, Ferri J, Sciote JJ. Epigenetic influence of KAT6B and HDAC4 in the development of skeletal malocclusion. American Journal of Orthodontics and Dentofacial Orthopedics. 2013;144(4):568-76. | Title |
|  | Huisinga-Fischer CE, Vaandrager JM, Prahl-Andersen B. Longitudinal results of mandibular distraction osteogenesis in hemifacial microsomia. Journal of Craniofacial Surgery. 2003;14(6):924-33. | Title |
|  | Huisinga-Fischer CE, Vaandrager JM, Zonneveld FW, Prahl-Andersen B. Precision and accuracy of CT-based measurements of masticatory muscles in patients with hemifacial microsomia. Dentomaxillofac Radiol. 2004;33(1):12-6. | Title |
|  | Huisinga-Fischer CE, Zonneveld FW, Vaandrager JM, Prahl-Andersen B. Relationship in hypoplasia between the masticatory muscles and the craniofacial skeleton in hemifacial microsomia, as determined by 3-D CT imaging. Journal of Craniofacial Surgery. 2001;12(1):31-40. | Title |
|  | Humphreys C, Jeffery N. Sexual dimorphism of the human masseter muscle and endocranium. Journal of Anatomy. 2018;232(2):339. | Title |
|  | Hunt N, Shah R, Sinanan A, Lewis M. Northcroft Memorial Lecture 2005: Muscling in on malocclusions: Current concepts on the role of muscles in the aetiology and treatment of malocclusion. Journal of Orthodontics. 2006;33(3):187-97. | Title |
|  | Hunt N, Shah R, Sinanan A, Lewis M. Muscular interference in malocclusion: present concepts of the role of the muscles in the etiology and therapy of malocclusion. L' Orthodontie française. 2007;78(2):79-88. | Title |
|  | Hunt NP. Musculo-skeletal adaptation to disturbances of the cranio-facial complex [Dissertation/Thesis]1992. | Title |
|  | Hunter EM. Biomechanical Form and Function in Primate Seed Predators : Common Solutions for Similar Mechanical Challenges? 2021. | Title |
|  | Hupauf L. Clinical functional diagnosis as a screening method. Zahnarztliche Mitteilungen. 1978;68(13):701-5. | Title |
|  | Hwang K, Lee DK, Kim HJ, Shin YH, Chung IH. Zygomaticomandibularis muscle. Journal of Craniofacial Surgery. 2005;16(4):655-7. | Title |
|  | Hwang S, Jeong S, Choi YJ, Chung CJ, Lee HS, Kim KH. Three-dimensional evaluation of dentofacial transverse widths of adults with various vertical facial patterns. American Journal of Orthodontics and Dentofacial Orthopedics. 2018;153(5):692-700. | Abstract |
|  | Hwang Y, Lee YH, Cho DH, Kim M, Lee DS, Cho HJ. Applicability of the masseter muscle as a nutritional biomarker. Medicine (United States). 2020;99(6). | Title |
|  | Hylander WL, Johnson KR. In vivo bone strain patterns in the zygomatic arch of macaques and the significance of these patterns for functional interpretations of craniofacial form. American Journal of Physical Anthropology. 1997;102(2):203-32. | Title |
|  | Iacomino E, Sinatti G, Pasqua M, Tucci C, Picchi G, Cipolloni G, Marco GPD. Anisakis in oral cavity: A rare case of an emerging disease. Oral and Maxillofacial Surgery Cases. 2020;6(1). | Title |
|  | Ibrová A, Dupej J, Stránská P, Velemínský P, Poláček L, Velemínská J. Facial skeleton asymmetry and its relationship to mastication in the Early Medieval period (Great Moravian Empire, Mikulčice, 9th-10th century). Arch Oral Biol. 2017;84:64-73. | Title |
|  | Idriceanu TM, Sindou M. Painful spasms in facial, masticatory, and motor ocular muscles reversed after microvascular decompression of a neurovascular conflict at brainstem. Acta Neurochirurgica. 2017;159(9):1707-11. | Title |
|  | Idris G, Galland B, Robertson CJ, Farella M. Efficacy of a mandibular advancement appliance on sleep disordered breathing in children: a study protocol of a crossover randomized controlled trial. Frontiers in physiology. 2016;7(AUG). | Title |
|  | Iguchi H, Yamada K, Yamane H, Hashimoto S. Epithelioid myoepithelioma of the accessory parotid gland: Pathological and magnetic resonance imaging findings. Case Reports in Oncology. 2014;7(2):310-5. | Title |
|  | Iida T, Komiyama O, Obara R, Baad-Hansen L, Kawara M, Svensson P. Influence of visual feedback on force-EMG curves from spinally innervated versus trigeminally innervated muscles. Archives of Oral Biology. 2013;58(3):331-9. | Title |
|  | Iida T, Komiyama O, Obara R, Baad-Hansen L, Kawara M, Svensson P. Repeated clenching causes plasticity in corticomotor control of jaw muscles. European Journal of Oral Sciences. 2014;122(1):42-8. | Title |
|  | Iida T, Overgaard A, Komiyama O, Weibull A, Baad-Hansen L, Kawara M, et al. Analysis of brain and muscle activity during low-level tooth clenching - a feasibility study with a novel biting device. Journal of Oral Rehabilitation. 2014;41(2):93-100. | Title |
|  | Iinuma T, Arai Y, Fukumoto M, Takayama M, Abe Y, Asakura K, et al. Maximum occlusal force and physical performance in the oldest old: The Tokyo oldest old survey on total health. Journal of the American Geriatrics Society. 2012;60(1):68-76. | Title |
|  | Ikeda R, Kobayashi T, Yoshida M, Yoshida N, Kikuchi T, Oshima T, et al. Patulous Eustachian Tube and Otitis Media With Effusion as Complications After Trigeminal Nerve Injury. Otology & Neurotology. 2017;38(8):1125-8. | Title |
|  | Ikenaga N, Yamaguchi K, Daimon S. Effect of mouth breathing on masticatory muscle activity during chewing food. Journal of Oral Rehabilitation. 2013;40(6):429-35. | Title |
|  | Ikuta M, Iida T, Kothari M, Shimada A, Komiyama O, Svensson P. Impact of sleep bruxism on training-induced cortical plasticity. Journal of Prosthodontic Research. 2019;63(3):277-82. | Title |
|  | Ilhan G, Sarifakiogullari A, Agir Y, Bulgurcu M, Biliz H, Kaya H. A diffuse large B cell lymphoma case presenting with femur mass. Leukemia Research. 2015;39:S29-S30. | Title |
|  | Imai T. The impairment of excitation-contraction (E-C) coupling in myasthenia gravis. Clinical Neurology. 2012;52(11):1309-11. | Title |
|  | Imai T, Tsuda E, Hozuki T, Yamauchi R, Saitoh M, Hisahara S, et al. Early effect of tacrolimus in improving excitation-contraction coupling in myasthenia gravis. Clinical Neurophysiology. 2012;123(9):1886-90. | Title |
|  | Imamura K, Terajima M, Nakashima A, Takahashi I. A three-dimensional analysis of masticatory muscles in laterognathism. Orthodontic Waves. 2019;78(4):143-50. | Full text  No skeletal patterns |
|  | Imanimoghaddam M, Madani AS, Hashemi EM. The evaluation of lateral pterygoid muscle pathologic changes and insertion patterns in temporomandibular joints with or without disc displacement using magnetic resonance imaging. International Journal of Oral and Maxillofacial Surgery. 2013;42(9):1116-20. | Title |
|  | Imanimoghaddam M, Rahpeyma A, Madani A, Armanpoor P, Armanpoor P. Oral submucous fibrosis: A case report. Journal of Kerman University of Medical Sciences. 2019;26(4):316-9. | Title |
|  | Impellizzeri A, Serritella E, Putrino A, Vizzielli G, Polimeni A, Galluccio G. Assessment of Masticatory and Cervical Muscles' Thickness by Ultrasonography in Patients with Facial Asymmetry. Clin Ter. 2019;170(4):e272-e7. | Full text  Only asymmetry |
|  | İnan S, Çaylaklı F, Canpolat T. Parotid gland tuberculosis accompanied by brucellosis. B-ENT. 2021;17(2):124-6. | Title |
|  | Ingervall B. Studies of mandibular positions in children. Odontologisk revy Supplement. 1968;15:1-53. | Title |
|  | Ingervall B, Bitsanis E. A pilot study of the effect of masticatory muscle training on facial growth in long-face children. European Journal of Orthodontics. 1987;9(1):15-23. | Title |
|  | Ingervall B, Carlsson GE. Masticatory muscle activity before and after elimination of balancing side occlusal interference. Journal of Oral Rehabilitation. 1982;9(3):183-92. | Title |
|  | Ingervall B, Minder C. Correlation between maximum bite force and facial morphology in children. Angle Orthodontist. 1997;67(6):415-22. | Title |
|  | Ingervall B, Thilander B. Activity of temporal and masseter muscles in children with a lateral forced bite. Angle Orthodontist. 1975;45(4):249-58. | Title |
|  | Ingervall B, Thüer U, Kuster R. Lack of correlation between mouth-breathing and bite force. Eur J Orthod. 1989;11(1):43-6. | Title |
|  | Innocenti M, Mori F, Raffaini M, Lucattelli E, Innocenti A. Mandibular ramus and condyle reconstruction with vascularized proximal fibular epiphyseal transfer in the pediatric patient: A case report. Microsurgery. 2020;40(7):818-22. | Title |
|  | Inomata T, Tanaka J, Tanaka N, Kurosaka M. A case of buccal vegetative foreign body difficult to diagnose. Advances in Oral and Maxillofacial Surgery. 2021;3. | Title |
|  | Inoue M, Ono T, Kameo Y, Sasaki F, Ono T, Adachi T, Nakashima T. Forceful mastication activates osteocytes and builds a stout jawbone. Scientific Reports. 2019;9. | Title |
|  | Inoue N, Sakashita R, Kamegai T. REDUCTION OF MASSETER MUSCLE-ACTIVITY IN BOTTLE-FED BABIES. Early Human Development. 1995;42(3):185-93. | Title |
|  | Inoue-Minakuchi M, Kuboki T, Maekawa K, Yanagi Y, Inoue E, Wakasa T, et al. Signal intensity changes in T2-weighted MR image of the human trapezius muscle upon cold pressor stimulation. Dentomaxillofacial Radiology. 2002;31(6):350-4. | Title |
|  | Insoft MD, Hocevar RA, Gibbs CH. The nonsurgical treatment of a Class II open bite malocclusion. American journal of orthodontics and dentofacial orthopedics : official publication of the American Association of Orthodontists, its constituent societies, and the American Board of Orthodontics. 1996;110(6):598-605. | Title |
|  | Iodice G, Danzi G, Cimino R, Paduano S, Michelotti A. Association between posterior crossbite, masticatory muscle pain, and disc displacement: A systematic review. European Journal of Orthodontics. 2013;35(6):737-44. | Title |
|  | Iodice G, Danzi G, Cimino R, Paduano S, Michelotti A. Association between posterior crossbite, skeletal, and muscle asymmetry: a systematic review. Eur J Orthod. 2016;38(6):638-51. | Title |
|  | Ioi H, Kawakatsu M, Nakata S, Nakasima A, Counts AL. Mechanomyogram and electromyogram analyses during isometric contraction in human masseter muscle. Australian Orthodontic Journal. 2008;24(2):116-20. | Title |
|  | Iordache CM, Antohe ME, Dascalu CG, Fatu AM, Ancuta C, Tanculescu O. ADVANCED IMAGING FOR THE DIAGNOSIS AND MONITORING OF TEMPOROMANDIBULAR JOINT PATHOLOGY IN SYSTEMIC SCLEROSIS. Romanian Journal of Oral Rehabilitation. 2020;12(3):234-8. | Title |
|  | Ip KC. The Development of a Temporomandibular Force Simulator to Study Craniofacial Strain <i>In-vitro</i> [Dissertation/Thesis]2018. | Title |
|  | Ip KKC, You P, Ferreira LM, Moore CC. Biomechanical Impact of a Zygoma Complex Fracture Using Human Cadaver. J Craniofac Surg. 2021;32(6):2045-9. | Title |
|  | Ip KKC, You P, Moore CC, Ferreira LM. Bite Force Simulator: A Novel Technique to Simulate Craniofacial Strain In Vitro. J Craniofac Surg. 2020;31(3):838-42. | Title |
|  | Isa M, Hongo Y, Sakamoto N, Yamazaki K, Takazaki H, Asakuma J, et al. Immune checkpoint inhibitor-related myositis and myocarditis with multiple myositis-specific/−associated antibodies. Journal of the Neurological Sciences. 2023;444. | Title |
|  | Isberg A, Eliasson S. A cephalometric analysis of patients with coronoid process enlargement and locking. American Journal of Orthodontics and Dentofacial Orthopedics. 1990;97(1):35-40. | Title |
|  | Iscan HN, Sarisoy L. Comparison of the effects of passive posterior bite-blocks with different construction bites on the craniofacial and dentoalveolar structures. American journal of orthodontics and dentofacial orthopedics. 1997;112(2):171‐8. | Title |
|  | Ishak MI, Kadir MRA, Sulaiman E, Kasim NHA. Finite element analysis of zygomatic implants in intrasinus and extramaxillary approaches for prosthetic rehabilitation in severely atrophic maxillae. International Journal of Oral and Maxillofacial Implants. 2013;28(3):e151-e60. | Title |
|  | Ishida T, Yabushita T, Soma K. Functional Changes of Temporomandibular Joint Mechanoreceptors Induced by Reduced Masseter Muscle Activity in Growing Rats. Angle Orthodontist. 2009;79(5):978-83. | Title |
|  | Ishihara Y, Kuroda S, Nishiyama A, Sasaki A, Takano-Yamamoto T, Yamashiro T. Functional improvements after orthodontic-surgical reconstruction in a patient with multiple maxillofacial fractures. American Journal of Orthodontics and Dentofacial Orthopedics. 2012;142(4):534-45. | Title |
|  | Ishii H. A study on the relationships between imbalance of stomatognathic function and asymmetry of craniofacial morphology, and the center of gravity of the upright posture. [Osaka Daigaku shigaku zasshi] The journal of Osaka University Dental Society. 1990;35(2):517-56. | Title |
|  | Islam I, Lim AAT, Wong RCW. Changes in bite force after orthognathic surgical correction of mandibular prognathism: a systematic review. International Journal of Oral and Maxillofacial Surgery. 2017;46(6):746-55. | Title |
|  | Isola G, Anastasi GP, Matarese G, Williams RC, Cutroneo G, Bracco P, Piancino MG. Functional and molecular outcomes of the human masticatory muscles. Oral Diseases. 2018;24(8):1428-41. | Title |
|  | Ispir NG, Toraman M. The relationship of masseter muscle thickness with face morphology and parafunctional habits: an ultrasound study. Dentomaxillofac Radiol. 2022;51(8):20220166. | Included |
|  | Issa SA, Abdulnabi HA, Jameel ME. Orofacial tuberculosis: A diagnostic challenge. IDCases. 2020;21. | Title |
|  | Ito K, Go Y, Tatsumoto S, Usui C, Mizuno Y, Ikami E, et al. Gene expression profiling of the masticatory muscle tendons and Achilles tendons under tensile strain in the Japanese macaque Macaca fuscata. PLoS ONE. 2023;18(1 January). | Title |
|  | Iwai T, Ohashi N, Sugiyama S, Kitajima H, Hirota M, Yamanaka S, Mitsudo K. Actinomycotic osteomyelitis with proliferative periostitis arising in the mandibular ramus: an unusual case with spontaneous bone regeneration after coronoidectomy. Oral Radiology. 2021;37(1):137-45. | Title |
|  | Iwasa A, Tanaka E. Signs, Symptoms, and Morphological Features of Idiopathic Condylar Resorption in Orthodontic Patients: A Survey-Based Study. Journal of Clinical Medicine. 2022;11(6). | Title |
|  | Iwasaki LR, Liu Y, Liu H, Nickel JC. Jaw mechanics in dolichofacial and brachyfacial phenotypes: A longitudinal cephalometric-based study. Orthodontics & Craniofacial Research. 2017;20:145-50. | Title |
|  | Iwasaki LR, Thornton BR, McCall Jr WD, Nickel JC. Individual variations in numerically modeled human muscle and temporomandibular joint forces during static biting. Journal of Orofacial Pain. 2004;18(3):235-45. | Title |
|  | Iwasaki M, Hirano H, Motokawa K, Shirobe M, Edahiro A, Ohara Y, et al. Interrelationships among whole-body skeletal muscle mass, masseter muscle mass, oral function, and dentition status in older Japanese adults. BMC Geriatr. 2021;21(1):582. | Title |
|  | Iwase M, Ohashi M, Tachibana H, Toyoshima T, Nagumo M. Bite force, occlusal contact area and masticatory efficiency before and after orthognathic surgical correction of mandibular prognathism. International Journal of Oral and Maxillofacial Surgery. 2006;35(12):1102-7. | Title |
|  | Iyota K, Mizutani S, Oku S, Asao M, Futatsuki T, Inoue R, et al. A Cross-Sectional Study of Age-Related Changes in Oral Function in Healthy Japanese Individuals. International Journal of Environmental Research and Public Health. 2020;17(4). | Title |
|  | Jaberzadeh S, Brodin P, Flavel SC, O'Dwyer NJ, Nordstrom MA, Miles TS. Pulsatile control of the human masticatory muscles. Journal of Physiology. 2003;547(2):613-20. | Title |
|  | Jacobson A. Orthodontics-Mechanical or biologic objectives? American Journal of Orthodontics. 1973;64(1):1-16. | Title |
|  | Jafari M, Ghasemi M, Dehghan Manshdi F, Akbarzadeh Baghban A. A comparison of ultrasonic thickness of masseter muscle between patients with bruxism and healthy people. Journal of Babol University of Medical Sciences. 2017;19(8):28-32. | Title |
|  | Jäger K, Schneider M. How do occlusion disorder and stress influence cybernetic masticatory control? Schweizer Monatsschrift fur Zahnmedizin = Revue mensuelle suisse d"odonto-stomatologie = Rivista mensile svizzera di odontologia e stomatologia / SSO. 1992;102(5):536-40. | Title |
|  | Jamal BT, Tuluc M, Gold L, Heffelfinger R, Taub DI. A Radiolucent Lesion in the Posterior Mandible. Journal of Oral and Maxillofacial Surgery. 2010;68(6):1371-6. | Title |
|  | Janáček J, Cvetko E, Kubínová L, Travnik L, Eržen I. A novel method for evaluation of capillarity in human skeletal muscles from confocal 3D images. Microvascular Research. 2011;81(2):231-8. | Title |
|  | Jank S, Zangerl A, Kloss FR, Laimer K, Missmann M, Schroeder D, Mur E. High resolution ultrasound investigation of the temporomandibular joint in patients with chronic polyarthritis. International Journal of Oral and Maxillofacial Surgery. 2011;40(1):45-9. | Title |
|  | Jankelson B. Three-dimensional orthodontic diagnosis and treatment. A neuromuscular approach. Journal of clinical orthodontics : JCO. 1984;18(9):627-36. | Title |
|  | Janovic A, Saveljic I, Vukicevic A, Nikolic D, Rakocevic Z, Jovicic G, et al. Occlusal load distribution through the cortical and trabecular bone of the human mid-facial skeleton in natural dentition: A three-dimensional finite element study. Annals of Anatomy. 2015;197:16-23. | Title |
|  | Jarraya M, Quijano-Roy S, Monnier N, Béhin A, Avila-Smirnov D, Romero NB, et al. Whole-Body muscle MRI in a series of patients with congenital myopathy related to TPM2 gene mutations. Neuromuscular Disorders. 2012;22(SUPPL. 2):S137-S47. | Title |
|  | Javadrashid R, Fouladi DF, Golamian M, Hajalioghli P, Daghighi MH, Shahmorady Z, Niknejad MT. Visibility of different foreign bodies in the maxillofacial region using plain radiography, CT, MRI and ultrasonography: An in vitro study. Dentomaxillofacial Radiology. 2015;44(4). | Title |
|  | Jawalekar R, Badole N, Khan A, Jaiswal E. Comparative assessment of cortical bone thickness at Infrazygomatic crest, Buccal shelf area, anterior maxilla for placement of bone screws in normodivergent, hypodivergent and hyperdivergent patients protocol for CBCT study. European Journal of Molecular and Clinical Medicine. 2021;8(3):3180-93. | Title |
|  | Jayakumar P, FelsyPremila G, Muthu MS, Kirubakaran R, Panchanadikar N, Al-Qassar SS. Bite force of children and adolescents: a systematic review and meta-analysis. Journal of Clinical Pediatric Dentistry. 2023;47(3):39-53. | Title |
|  | Jayavelu P, Shrutha SP, Vinit GB. Temperomandibular joint ankylosis in children. Journal of Pharmacy and Bioallied Sciences. 2014;6:S178-S81. | Title |
|  | Jeffery N, Manson A. Postnatal growth and spatial conformity of the cranium, brain, eyeballs and masseter muscles in the macaque (<i>Macaca mulatta</i>). Journal of Anatomy. 2023;243(4):590-604. | Title |
|  | Jeffery N, Mendias C. Endocranial and masticatory muscle volumes in myostatin-deficient mice. Royal Society Open Science. 2014;1(4). | Title |
|  | Jeon J. The Facial, Skeletal, and Dental Effects of Repeated Botulinum Toxin Injections in the Human Masseter [Dissertation/Thesis]2020. | Title |
|  | Jeon KJ, Lee C, Choi YJ, Han SS. Analysis of three-dimensional imaging findings and clinical symptoms in patients with temporomandibular joint disorders. Quantitative Imaging in Medicine and Surgery. 2021;11(5):1921-31. | Title |
|  | Ji B, Wang C, Song F, Chen M, Wang H. A new biomechanical model for evaluation of fixation systems of maxillofacial fractures. J Craniomaxillofac Surg. 2012;40(5):405-8. | Title |
|  | Jiang Q, Chen MJ, Yang C, Qiu YT, Tian Z, Zhang ZY, Qiu WL. Post-infectious myositis ossificans in medial, Lateral pterygoid muscles: A case report and review of the literature. Oncology Letters. 2015;9(2):920-6. | Title |
|  | Jiang Y, Shang F, Peng J, Liang J, Fan Y, Yang Z, et al. Automatic Masseter Muscle Accurate Segmentation from CBCT Using Deep Learning-Based Model. Journal of Clinical Medicine. 2023;12(1). | Title |
|  | Jiménez ID. Electromyography of masticatory muscles in three jaw registration positions. American Journal of Orthodontics and Dentofacial Orthopedics. 1989;95(4):282-8. | Title |
|  | Jindal G, Jindal S, Sharma P, Singla A. Rare enlargement of genial tubercles and its management: A case Report. Journal of Clinical and Diagnostic Research. 2015;9(11):ZD23-ZD4. | Title |
|  | Jockusch J, Hahnel S, Sobotta B, Nitschke I. The Effect of a Masticatory Muscle Training Program on Chewing Efficiency and Bite Force in People with Dementia. International Journal of Environmental Research and Public Health. 2022;19(7). | Title |
|  | Jokaji R, Ooi K, Yahata T, Nakade Y, Kawashiri S. Evaluation of factors related to morphological masseter muscle changes after preoperative orthodontic treatment in female patients with skeletal class III dentofacial deformities. BMC Oral Health. 2022;22(1):292. | Title |
|  | Jonas I, Mann W, Münker G, Junker W, Schumann K. Relationship between tubal function, craniofacial morphology and disorder of deglutition. Arch Otorhinolaryngol. 1978;218(3-4):151-62. | Title |
|  | Jonasson G. Mandibular alveolar bone mass, structure and thickness in relation to skeletal bone density in dentate women. Swed Dent J Suppl. 2005(177):1-63. | Title |
|  | Jonasson G, Kiliaridis S. The association between the masseter muscle, the mandibular alveolar bone mass and thickness in dentate women. Arch Oral Biol. 2004;49(12):1001-6. | Full text  No skeletal patterns |
|  | Jones LC, Waite PD. Orthognathic surgery and partial glossectomy in a patient with merosin-deficient congenital muscular dystrophy. Journal of Oral and Maxillofacial Surgery. 2012;70(2):e141-e6. | Title |
|  | Joshua BZ, Silberstein E, Diomin V, Bodner L. Desmoplastic fibroma of the mandible associated with pathological fracture in a pediatric patient. International Journal of Pediatric Otorhinolaryngology Extra. 2014;9(2):60-3. | Title |
|  | Joujima T, Oda M, Sasaguri M, Habu M, Kataoka S, Miyamura Y, et al. Evaluation of velopharyngeal function using high-speed cine-magnetic resonance imaging based on T2-weighted sequences: a preliminary study. International Journal of Oral and Maxillofacial Surgery. 2020;49(4):432-41. | Title |
|  | Jovic S, Brajkovic D, Borilovic M, Marjanovic U, Brkic M, Kozomara R, Stošic S. Recurring myositis ossificans traumatica of temporal muscle: A case report. Vojnosanitetski Pregled. 2021;78(2):255-60. | Title |
|  | Joyal KM, Michaud J, Van Der Knaap MS, Bugiani M, Venkateswaran S. Severe TUBB4A-related hypomyelination with atrophy of the basal ganglia and cerebellum: Novel neuropathological findings. Journal of Neuropathology and Experimental Neurology. 2019;78(1):3-9. | Title |
|  | Judge RB, Palamara JEA, Taylor RG, Davies HMS, Clement JG. Description of a photoelastic coating technique to describe surface strain of a dog skull loaded in vitro. Journal of Prosthetic Dentistry. 2003;90(1):92-6. | Title |
|  | Julià-Sánchez S, Álvarez-Herms J, Burtscher M. Dental occlusion and body balance: A question of environmental constraints? Journal of Oral Rehabilitation. 2019;46(4):388-97. | Title |
|  | Julià-Sánchez S, Álvarez-Herms J, Cirer-Sastre R, Corbi F, Burtscher M. The influence of dental occlusion on dynamic balance and muscular tone. Frontiers in Physiology. 2020;10. | Title |
|  | Julien KC, Buschang PH, Throckmorton GS, Dechow PC. Normal masticatory performance in young adults and children. Archives of Oral Biology. 1996;41(1):69-75. | Title |
|  | Junaid ST, Muhammad SA, Ahmed Z, Khubeb, Vasandani RK, Ahmed J. Mammary analogue secretory carcinoma of the parotid gland: A rare tumour entity. Journal of the Pakistan Medical Association. 2023;73(2):412-5. | Title |
|  | Jung BK, Park H, Cheon YW, Yun IS, Choi JW, Kim HJ, et al. Clinical investigation of botulinum toxin (prabotulinumtoxin A) for bruxism related to masseter muscle hypertrophy: A prospective study. Journal of Cranio-Maxillofacial Surgery. 2023;51(5):332-7. | Title |
|  | Jung G, Cho KJ, Choi SH, Kim MJ. Dedifferentiated Extraskeletal Myxoid Chondrosarcoma of the Masticator Space - A Case Report. Korean Journal of Pathology. 2011;45:S101-S5. | Title |
|  | Jung H, Strait D, Rolian C, Baab KL. Functional morphological integration related to feeding biomechanics in the hominine skull. Journal of Human Evolution. 2023;182. | Title |
|  | Jung H, Woo EJ, von Cramon-Taubadel N. The relationship between ante-mortem molar loss and mandibular ramus shape in an archaeological population from Korea. International Journal of Osteoarchaeology. 2020;30(2):197-205. | Title |
|  | Junn JC, Som PM. Maxillofacial Skeleton and Facial Anatomy. Neuroimaging Clinics of North America. 2022;32(4):735-48. | Title |
|  | Jussila P, Krooks L, Näpänkangas R, Päkkilä J, Ländesmäki R, Pirttiniemi P, Raustia A. The role of occlusion in temporomandibular disorders (TMD) in the Northern Finland Birth Cohort (NFBC) 1966. Cranio-the Journal of Craniomandibular & Sleep Practice. 2019;37(4):231-7. | Title |
|  | k7dwj RBR. Effects of orthodontic treatment on promoting neural adaptations in adults with malocclusion. https://trialsearchwhoint/Trial2aspx?TrialID=RBR-6k7dwj. 2018. | Title |
|  | Kaban LB, Posnick JC. Re: Role of alloplastic reconstruction of the temporomandibular joint in the juvenile idiopathic arthritis population. British Journal of Oral and Maxillofacial Surgery. 2022;60(5):668-9. | Title |
|  | Kafas P, Chiotaki N, Kafas G. Glucosaminoglycan phonophoresis of the TMJ in the symptomatic treatment of internal derangement. Journal of Medical Sciences. 2007;7(1):158-60. | Title |
|  | Kahn JL, Bourjat P. The masseter: An enigmatic muscle. Journal de Radiologie. 2001;82(5):547-52. | Title |
|  | Kahn JL, Wolfram-Gabel R, Bourjat P. Anatomy and imaging of the deep fat of the face. Clinical Anatomy. 2000;13(5):373-82. | Title |
|  | Kakudo K, Kubo H, Fujii T. Newly concept of the limited mouth opening disorder: Clinical characteristics and diagnosis of patients with masticatory muscle tendon-aponeurosis hyperplasia. Journal of Oral and Maxillofacial Surgery. 2014;72(9):e224. | Title |
|  | Kalai Selvi A, Shanmugham KG, Kannan MS. A review on role of tongue in Malocclusion. Indian Journal of Public Health Research and Development. 2019;10(12):1067-74. | Title |
|  | Kalpidis IP, Kapoukranidou D, Charalambakis N, Chatzisotiriou A, Albani M. THREE-DIMENSIONAL MORPHOMETRIC MAPPING OF RAT MUSCLE FIBERS. Muscle & Nerve. 2013;48(6):951-7. | Title |
|  | Kamada T, Ohdaira H, Ito E, Fuse Y, Takahashi J, Nakashima K, et al. Preoperative Masseter Muscle Sarcopenia Predicts Mortality in Patients with Oesophageal Cancer. Anticancer Research. 2022;42(1):301-10. | Title |
|  | Kamata S. [Reflex response of temporal muscle induced by mechanical stimulation to periodontal ligament--in the lateral jaw movement during mastication]. Kokubyo Gakkai Zasshi. 1994;61(1):82-97. | Title |
|  | Kamegai T, Tatsuki T, Nagano H, Mitsuhashi H, Kumeta J, Tatsuki Y, et al. A determination of bite force in northern Japanese children. European Journal of Orthodontics. 2005;27(1):53-7. | Title |
|  | Kami Y, Chikui T, Togao O, Kawano S, Fujii S, Ooga M, et al. Usefulness of reconstructed images of Gd-enhanced 3D gradient echo sequences with compressed sensing for mandibular cancer diagnosis: comparison with CT images and histopathological findings. European Radiology. 2023;33(2):845-53. | Title |
|  | Kammoun R, Chaabani I, Jaziri R, Sriha B, Ben Alaya T. Foreign body reaction granuloma mimicking an aggressive tumor of the mandible. Clinical Case Reports. 2023;11(1). | Title |
|  | Kämppi A, Kämppi L, Kemppainen P, Kanerva M, Toppila J, Auranen M. Focal atrophy of the unilateral masticatory muscles caused by pure trigeminal motor neuropathy: case report. Clinical Case Reports. 2018;6(5):939-43. | Title |
|  | Kanaujia R, Aggarwal A, Misra RN. Multicompartmental Epidermoid Cyst Causing Chronic Parotid Gland and Masticator Space Muscle Atrophy. World Neurosurgery. 2021;150:89-91. | Title |
|  | Kanayama H, Masuda Y, Adachi T, Arai Y, Kato T, Morimoto T. Temporal alteration of chewing jaw movements after a reversible bite-raising in guinea pigs. Archives of Oral Biology. 2010;55(1):89-94. | Title |
|  | Kane AA, Lo LJ, Christensen GE, Vannier MW, Marsh JL. Relationship between bone and muscles of mastication in hemifacial microsomia. Plastic and Reconstructive Surgery. 1997;99(4):990-9. | Title |
|  | Kang J, Zhang J, Zheng J, Wang L, Li D, Liu S. 3D-printed PEEK implant for mandibular defects repair - a new method. Journal of the Mechanical Behavior of Biomedical Materials. 2021;116. | Title |
|  | Kang JH. Associations Among Temporomandibular Joint Osteoarthritis, Airway Dimensions, and Head and Neck Posture. Journal of Oral and Maxillofacial Surgery. 2020;78(12):2183.e1-.e12. | Title |
|  | Kang JH, Shin DS, Kim SW, Lim HJ, Kim BC. Volumetric Change in the Masseter and Lateral Pterygoid after Mandibular Setback. Journal of Personalized Medicine. 2022;12(5). | Title |
|  | Kang S, Song BI, Lee HJ, Jeong SY, Seo JH, Lee SW, et al. Isolated Facial Muscle Metastasis From Renal Cell Carcinoma on F-18 FDG PET/CT. Clinical Nuclear Medicine. 2010;35(4):263-4. | Title |
|  | Kanke K, Abe T, Abe M, Mori Y, Hoshi K, Takato T. In-hospital surgical treatment for haemorrhage after aesthetic mandibular osteotomy performed as an office-based day surgery: A case report. Annals of Medicine and Surgery. 2017;24:15-8. | Title |
|  | Kaplan M, Caloglu M, Caloglu VY, Aktoz T, Usta U, Karagöl H, Inci O. Parotid gland metastasis from renal cell carcinoma: An unusual site for metastasis. American Journal of Case Reports. 2008;9:316-20. | Title |
|  | Kaplan RG. Induced condylar growth in a patient with hemifacial microsomia. Angle Orthod. 1989;59(2):85-90. | Title |
|  | Kappert KDR, Connesson N, Elahi SA, Boonstra S, Balm AJM, van der Heijden F, Payan Y. In-vivo tongue stiffness measured by aspiration: Resting vs general anesthesia. Journal of Biomechanics. 2021;114. | Title |
|  | Karaali S, Emekli U. Myositis Ossificans Traumatica of the Medial Pterygoid Muscle After Third Molar Tooth Extraction: A Case Report and Review of Literature. Journal of Oral and Maxillofacial Surgery. 2018;76(11):2284.e1-.e5. | Title |
|  | Karaman E, Mercan H, Ozdilek A, Alimoglu Y, Korkut N. Huge arteriovenous malformation in masseter muscle. Journal of Craniofacial Surgery. 2009;20(4):1292-4. | Title |
|  | Karataban P, Demirel O, Ortug G. An investigation into the total mandibular length and its potential correlation with the intraoral forces. Translational Research in Anatomy. 2022;27. | Title |
|  | Karatzanis A, Velegrakis S, Liva G, Kyrmizakis D, Prokopakis E. Management of a Buccal Space Mass: A Clinical Case Report. Case Reports in Otolaryngology. 2020;2020. | Title |
|  | Karjalainen M, Le Bell Y, Jämsä T, Karjalainen S. Prevention of temporomandibular disorder-related signs and symptoms in orthodontically treated adolescents: A 3-year follow-up of a prospective randomized trial. Acta Odontologica Scandinavica. 1997;55(5):319-24. | Title |
|  | Karkas AA, Schmerber SA, Bettega GV, Reyt EP, Righini CA. Osteoplastic maxillotomy approach for infraorbital nerve schwannoma, a case report. Head and Neck. 2008;30(3):401-4. | Title |
|  | Karwetzky R, Tielsch-Keuthen H. Kraniometrische und gnathometrische Auswirkungen bei der Zerebralparese im kauorgan. Fortschritte der Kieferorthopädie. 1972;33(2):221-30. | Title |
|  | Kasahara M, Matsunaga S, Someya T, Kitamura K, Odaka K, Ishimoto T, et al. Micro- and nano-bone analyses of the human mandible coronoid process and tendon-bone entheses. Journal of Biomedical Materials Research - Part B Applied Biomaterials. 2020;108(7):2799-806. | Title |
|  | Kasai K, Kanazawa E, Aboshi H, Tuisuva J, Takahashi M, Matsuno M. Comparative study of craniofacial morphology and bite force in Fijians and Japanese. American Journal of Human Biology. 1998;10(1):63-72. | Abstract |
|  | Kasai K, Nakajima Y, Mashita M, Yasuda K, Enomoto Y, Go K, et al. Correlation between morphologies of mandibular vertical sections and linear measurements of the skull. Nihon Kyōsei Shika Gakkai zasshi = The journal of Japan Orthodontic Society. 1990;49(6):511-21. | Title |
|  | Kasai K, Richards LC, Brown T. Comparative study of craniofacial morphology in Japanese and Australian aboriginal populations. Human biology; an international record of research. 1993;65(5):821-34. | Title |
|  | Kasai K, Richards LC, Kanazawa E, Iwasawa T. Cephalometric analysis of masseter muscle and dentoskeletal morphology in dentate and edentulous humans. J Nihon Univ Sch Dent. 1997;39(2):78-85. | Title |
|  | Kasai K, Richards LC, Kanazawa E, Ozaki T, Iwasawa T. Relationship between attachment of the superficial masseter muscle and craniofacial morphology in dentate and edentulous humans. J Dent Res. 1994;73(6):1142-9. | Title |
|  | Kasai K, Takayama Y, Yokoyama A. Distribution of Occlusal Forces During Occlusal Adjustment of Dental Implant Prostheses: A Nonlinear Finite Element Analysis Considering the Capacity for Displacement of Opposing Teeth and Implants. International Journal of Oral & Maxillofacial Implants. 2012;27(2):329-35. | Title |
|  | Kasemsiri P, Solares CA, Carrau RL, Prosser JD, Prevedello DM, Otto BA, et al. Endoscopic endonasal transpterygoid approaches: Anatomical landmarks for planning the surgical corridor. Laryngoscope. 2013;123(4):811-5. | Title |
|  | Kashima K, Igawa K, Maeda S, Sakoda S. Analysis of muscle hardness in patients with masticatory myofascial pain. Journal of Oral and Maxillofacial Surgery. 2006;64(2):175-9. | Title |
|  | Kato C, Ono T. Anterior open bite due to temporomandibular joint osteoarthrosis with muscle dysfunction treated with temporary anchorage devices. American Journal of Orthodontics and Dentofacial Orthopedics. 2018;154(6):848-59. | Title |
|  | Kato H, Ota Y, Sasaki M, Arai T, Sekido Y, Tsukinoki K. A phlebolith in the anterior portion of the masseter muscle. Tokai Journal of Experimental and Clinical Medicine. 2012;37(1):25-9. | Title |
|  | Kato M, Saruta J, Takeuchi M, Sugimoto M, Kamata Y, Shimizu T, et al. Grinding patterns in migraine patients with sleep bruxism: a case-controlled study. Cranio - Journal of Craniomandibular Practice. 2016;34(6):371-7. | Title |
|  | Kato T, Takahashi S, Domon T. Effects of a Liquid Diet on the Temporomandibular Joint of Growing Rats. Medical Principles and Practice. 2015;24(3):257-62. | Title |
|  | Katsaros C. Masticatory muscle function and transverse dentofacial growth. Swed Dent J Suppl. 2001(151):1-47. | Abstract |
|  | Katsetos CD, Bianchi MA, Jaffery F, Koutzaki S, Zarella M, Slater R. Painful unilateral temporalis muscle enlargement: reactive masticatory muscle hypertrophy. Head Neck Pathol. 2014;8(2):187-93. | Title |
|  | Katsube M, Yamada S, Utsunomiya N, Yamaguchi Y, Takakuwa T, Yamamoto A, et al. A 3D analysis of growth trajectory and integration during early human prenatal facial growth. Sci Rep. 2021;11(1):6867. | Title |
|  | Katsumata A, Fujishita M, Ariji Y, Ariji E, Langlais RP. 3D CT evaluation of masseter muscle morphology after setback osteotomy for mandibular prognathism. Oral Surg Oral Med Oral Pathol Oral Radiol Endod. 2004;98(4):461-70. | Title |
|  | Katzberg RW, Westesson PL, Tallents RH, Drake CM. Anatomic disorders of the temporomandibular joint disc in asymptomatic subjects. Journal of Oral and Maxillofacial Surgery. 1996;54(2):147-55. | Title |
|  | Kawabata A, Kobayashi T, Takagi A, Kuroyanagi F, Washino K, Sabashi K, Kitai N. Multidirectional lip-closing force in adults with mandibular deviation. Journal of Oral Rehabilitation. 2013;40(9):664-9. | Title |
|  | Kawai N, Shibata M, Watanabe M, Horiuchi S, Fushima K, Tanaka E. Effects of functional training after orthognathic surgery on masticatory function in patients with mandibular prognathism. Journal of Dental Sciences. 2020;15(4):419-25. | Title |
|  | Kawai T, Murakami S, Kishino M, Matsuya T, Sakuda M, Fuchihata H. Diagnostic imaging in two cases of recurrent maxillary ameloblastoma: Comparative evaluation of plain radiographs, CT and MR images. British Journal of Oral and Maxillofacial Surgery. 1998;36(4):304-10. | Title |
|  | Kaya MS. Masticatory Parameters of Children with and without Caries in Mixed and Permanent Dentition [Dissertation/Thesis]2014. | Title |
|  | Kct. Effect of chewing exercise during surface electrical stimulation on the volume, muscle strength of masseter muscle in older adults. http://wwwwhoint/trialsearch/Trial2aspx?TrialID=KCT0005312. 2020. | Title |
|  | Kean MR, Houghton P. Polynesian face and dentition: Functional perspective. American Journal of Physical Anthropology. 1990;82(3):361-9. | Title |
|  | Kecik D, Kocadereli I, Saatci I. Evaluation of the treatment changes of functional posterior crossbite in the mixed dentition. American Journal of Orthodontics and Dentofacial Orthopedics. 2007;131(2):202-15. | Title |
|  | Kelley P, Hopper R, Gruss J. Evaluation and treatment of zygomatic fractures. Plastic and Reconstructive Surgery. 2007;120(7 SUPPL. 2):5S-15S. | Title |
|  | Keramidas T, Lagogiannis G, Vlachou V, Katsikeris N. Congenital infiltrating lipomatosis of the face with associated involvement of the TMJ structures. Case report and review of the literature. Journal of Cranio-Maxillofacial Surgery. 2012;40(8):750-6. | Title |
|  | Kermanshah H, Alzwghaibi A, Al-Tufaili M, Ghabraei S. Canine Rise Method: A Conservative Approach for Worn Teeth Rehabilitation with Different Adhesive Restorative Materials. Case Reports in Dentistry. 2022;2022. | Title |
|  | Keser G, Bayrakdar IS, Pekiner FN, Çelik Ö, Orhan K. A deep learning approach for masseter muscle segmentation on ultrasonography. Journal of Ultrasonography. 2022;22(91):E204-E8. | Title |
|  | Kesterke MJ, Butaric LN, Zhang Q, Han T, Zhu H, Zhang Q, Wang Q. Subsistence and facial form: Estimating masticatory muscle mechanical efficiency in historical populations from Northern China. American Journal of Physical Anthropology. 2018;165:138-9. | Title |
|  | Kesterke MJ, Judd MA, Mooney MP, Siegel MI, Elsalanty M, Howie RN, et al. Maternal environment and craniofacial growth: geometric morphometric analysis of mandibular shape changes with <i>in utero</i> thyroxine overexposure in mice. Journal of Anatomy. 2018;233(1):46-54. | Title |
|  | Khan E. Medial pterygoid muscle traumatic myositis ossificans: A case report from Saudi Arabia. Medical Science. 2021;25(117):2744-7. | Title |
|  | Khiabani K, Keyhan SO, Razmdideh R, Chaleh ZC, Amirzade-Iranaq MH. Effect of different miniplate osteosynthesis in different mandibular angle fracture patterns on bite force: A 3D finite element analysis. Journal of Oral and Maxillofacial Surgery Medicine and Pathology. 2018;30(4):324-9. | Title |
|  | Khong PL. Paediatric PET/CT: Physiologic uptake, normal variants and pitfalls. Cancer Imaging. 2019;19. | Title |
|  | Kidder GM, Solow RA. Precision occlusal splints and the diagnosis of occlusal problems in myogenous orofacial pain patients. General Dentistry. 2014;62(2):24-31. | Title |
|  | Kijak E, Margielewicz J, Gąska D, Lietz-Kijak D, Więckiewicz W. Identification of mastication organ muscle forces in the biocybernetic perspective. BioMed Research International. 2015;2015. | Title |
|  | Kikuchi K, Inoue H, Miyazaki Y, Ide F, Matsuki E, Shigematu H, et al. Adult sporadic burkitt lymphoma of the oral cavity: A case report and literature review. Journal of Oral and Maxillofacial Surgery. 2012;70(12):2936-43. | Title |
|  | Kikuchi M, Korioth TW, Hannam AG. The association among occlusal contacts, clenching effort, and bite force distribution in man. J Dent Res. 1997;76(6):1316-25. | Title |
|  | Kiliaridis S. Masticatory muscle influence on craniofacial growth. Acta Odontologica Scandinavica. 1995;53(3):196-202. | Abstract |
|  | Kiliaridis S, Georgiakaki I, Katsaros C. Masseter muscle thickness and maxillary dental arch width. Eur J Orthod. 2003;25(3):259-63. | Full text  No skeletal patterns |
|  | Kiliaridis S, Johansson A, Haraldson T, Omar R, Carlsson GE. CRANIOFACIAL MORPHOLOGY OCCLUSAL TRAITS, AND BITE FORCE IN PERSONS WITH ADVANCED OCCLUSAL TOOTH WEAR. American Journal of Orthodontics and Dentofacial Orthopedics. 1995;107(3):286-92. | Title |
|  | Kiliaridis S, Kälebo P. Masseter Muscle Thickness Measured by Ultrasonography and its Relation to Facial Morphology. Journal of Dental Research. 1991;70(9):1262-5. | Full text  Only photos |
|  | Kiliaridis S, Katsaros C. The effects of myotonic dystrophy and Duchenne muscular dystrophy on the orofacial muscles and dentofacial morphology. Acta Odontol Scand. 1998;56(6):369-74. | Title |
|  | Kiliaridis S, Kjellberg H, Wenneberg B, Engström C. The relationship between maximal bite force, bite force endurance, and facial morphology during growth: A Cross-sectional study. Acta Odontologica Scandinavica. 1993;51(5):323-31. | Abstract |
|  | Kiliaridis S, Mahboubi PH, Raadsheer MC, Katsaros C. Ultrasonographic thickness of the masseter muscle in growing individuals with unilateral crossbite. Angle Orthod. 2007;77(4):607-11. | Full text  Cross-bites |
|  | Kiliaridis S, Mejersjo C, Thilander B. Muscle function and craniofacial morphology: A clinical study in patients with myotonic dystrophy. European Journal of Orthodontics. 1989;11(2):131-8. | Title |
|  | Kiliaridis S, Mills CM, Antonarakis GS. Masseter muscle thickness as a predictive variable in treatment outcome of the twin-block appliance and masseteric thickness changes during treatment. Orthod Craniofac Res. 2010;13(4):203-13. | Title |
|  | Kiliaridis S, Tzakis MG, Carlsson GE. Effects of fatigue and chewing training on maximal bite force and endurance. American Journal of Orthodontics and Dentofacial Orthopedics. 1995;107(4):372-8. | Title |
|  | Kilic N. Associations between upper lip activity and incisor position. Australian orthodontic journal. 2010;26(1):56-60. | Title |
|  | Killaridis S, Engstrom C, Chavez LME. INFLUENCE OF MASTICATORY MUSCLE FUNCTION ON CRANIOFACIAL GROWTH IN HYPOCALCEMIC RATS. Scandinavian Journal of Dental Research. 1992;100(6):330-6. | Title |
|  | Kim AM, Keenan BT, Jackson N, Chan EL, Staley B, Torigian DA, et al. Metabolic Activity of the Tongue in Obstructive Sleep Apnea A Novel Application of FDG Positron Emission Tomography Imaging. American Journal of Respiratory and Critical Care Medicine. 2014;189(11):1416-25. | Title |
|  | Kim CH, Lee JH, Cho JY, Lee JH, Kim KW. Skeletal stability after simultaneous mandibular angle resection and sagittal split ramus osteotomy for correction of mandible prognathism. Journal of oral and maxillofacial surgery. 2007;65(2):192‐7. | Title |
|  | Kim D, Park JH, Favero V, Mah J, Jung YS, Kim ST. Effect of botulinum toxin injection on asymmetric lower face with chin deviation. Toxins. 2020;12(7). | Title |
|  | Kim DD, Lazow SK, Har-El G, Berger JR. Myositis ossificans traumatica of masticatory musculature: A case report and literature review. Journal of Oral and Maxillofacial Surgery. 2002;60(9):1072-6. | Title |
|  | Kim DH, Lee JS, Pyo SW, Lee JH. Ascending Facial Necrotizing Fasciitis in a Patient Taking a Bisphosphonate. Journal of Oral and Maxillofacial Surgery. 2017;75(2):317-21. | Title |
|  | Kim H, Shin D, Kang J, Kim S, Lim H, Lee J, Kim B. Anatomical Characteristics of the Lateral Pterygoid Muscle in Mandibular Prognathism. Applied Sciences-Basel. 2021;11(17). | Included |
|  | Kim HE, Lee H. Factors affecting subjective and objective masticatory function in older adults: Importance of an integrated approach. Journal of Dentistry. 2021;113. | Title |
|  | Kim HE, Wallace J, Sohn W. Factors Affecting Masticatory Performance of Older Adults Are Sex-Dependent: A Cross-Sectional Study. International Journal of Environmental Research and Public Health. 2022;19(23). | Title |
|  | Kim HJ, Park KM, Tak HJ, Choi JW, Kang SH, Park W, et al. Skeletal unit construction of rat mandible based on the masticatory muscle anatomy and double microcomputed tomography. Anatomia Histologia Embryologia. 2018;47(5):417-27. | Title |
|  | Kim HJ, Tak HJ, Moon JW, Kang SN, Kim ST, He JQ, et al. Mandibular Vertical Growth Deficiency After Botulinum-Induced Hypotrophy of Masticatory Closing Muscles in Juvenile Nonhuman Primates. Frontiers in Physiology. 2019;10. | Title |
|  | Kim HW, Kim MY, Kim CH. A systematic review of therapeutic outcomes following treatment of squamous cell carcinoma of the retromolar trigone. Journal of the Korean Association of Oral and Maxillofacial Surgeons. 2021;47(4):291-314. | Title |
|  | Kim JR, Jo JH, Chung JW, Park JW. Upper cervical spine abnormalities as a radiographic index in the diagnosis and treatment of temporomandibular disorders. Oral Surg Oral Med Oral Pathol Oral Radiol. 2020;129(5):514-22. | Title |
|  | Kim JY, You HS, Huh JK, Park KH. Is There a Difference in Condyle Position Changing Pattern Between Deviated and Non-Deviated Sides After Intraoral Vertical Ramus Osteotomy in Facial Asymmetry? Journal of Oral and Maxillofacial Surgery. 2020;78(4). | Title |
|  | Kim KA, Park HS, Lee SY, Kim SJ, Baek SH, Ahn HW. Short-term changes in muscle activity and jaw movement patterns after orthognathic surgery in skeletal Class III patients with facial asymmetry. Korean Journal of Orthodontics. 2019;49(4):254-64. | Title |
|  | Kim MJ, Min SK. Bone marrow invasion and mylohyoid muscle invasion may indicate cervical lymph node metastasis from oral squamous cell carcinoma of the posterior mandibular alveolar ridge. Journal of Oral and Maxillofacial Surgery. 2014;72(9):e159-e60. | Title |
|  | Kim SE, Arzi B, Garcia TC, Verstraete FJM. Bite Forces and Their Measurement in Dogs and Cats. Frontiers in Veterinary Science. 2018;5. | Title |
|  | Kim SJ, Baik HS, Hwang CJ, Yu HS. Diagnosis and evaluation of skeletal Class III patients with facial asymmetry for orthognathic surgery using three-dimensional computed tomography. Seminars in Orthodontics. 2015;21(4):274-82. | Title |
|  | Kim SY, Alfafara A, Kim JW, Kim SJ. Traumatic Buccal Fat Pad Herniation in Young Children: A Systematic Review and Case Report. Journal of Oral and Maxillofacial Surgery. 2017;75(9):1926-31. | Title |
|  | Kim TH, Kim CH. Correlation between mandibular morphology and masticatory muscle thickness in normal occlusion and mandibular prognathism. Journal of the Korean Association of Oral and Maxillofacial Surgeons. 2020;46(5):313-20. | Included |
|  | Kim WH, Hong K, Lim D, Lee JH, Jung YJ, Kim B. Optimal Position of Attachment for Removable Thermoplastic Aligner on the Lower Canine Using Finite Element Analysis. Materials. 2020;13(15). | Title |
|  | Kim YG, Hwan S. Effect of mandibular setback surgery on occlusal force. Journal of Oral and Maxillofacial Surgery. 1997;55(2):121-6. | Title |
|  | Kim YI, Park SB, Jung YH, Hwang DS, Lee JY. Evaluation of intersegmental displacement according to osteosynthesis method for mandibular setback sagittal split ramus osteotomy using cone-beam computed tomographic superimposition. Journal of Oral and Maxillofacial Surgery. 2012;70(12):2893-8. | Title |
|  | Kimura A, Nagasao T, Kaneko T, Tamaki T, Miyamoto J, Nakajima T. Adaquate fixation of plates for stability during mandibular reconstruction. Journal of Cranio-Maxillofacial Surgery. 2006;34(4):193-200. | Title |
|  | Kimura K, Murakami H, Yamamoto M, Yokoyama T, Morita T, Ito Y, Hiraba K. Velocity-dependent EMG activity of masseter and sternocleidomastoideus muscles during a ballistic arm thrusting movement. Human Movement Science. 2007;26(1):48-67. | Title |
|  | Kimura T, Ohba S, Yoshimura H, Fujita S, Imamura Y, Kitagawa Y, Sano K. Keratocystic odontogenic tumor arising at the mandibular ramus with an impacted tooth: A case report and mimic lesions. Cranio - Journal of Craniomandibular Practice. 2016;34(1):58-63. | Title |
|  | King EW. Relapse of orthodontic treatment. Angle Orthodontist. 1974;44(4):300-15. | Title |
|  | King MK, Lee RR, Davis LE. Magnetic resonance imaging and computed tomography of skeletal muscles in oculopharyngeal muscular dystrophy. Journal of Clinical Neuromuscular Disease. 2005;6(3):103-8. | Title |
|  | Kinugawa K, Mano T, Nakagawa Y, Hotta N, Sugie K. Case report: Unilateral masticatory atrophy caused by pure trigeminal motor neuropathy. Radiology Case Reports. 2022;17(12):4542-5. | Title |
|  | Kirupa K, Divya Mary SM, Vaishnavi G, Nithya Nisha R, Rennie Mercy J, Jaiganesh G. A comparative study of ultrasound therapy and transcutaneous electrical nerve stimulation in reducing pain for temporomandibular joint disorder. Drug invention today. 2019;12(3):515‐7. | Title |
|  | Kitai N, Fujii Y, Murakami S, Furukawa S, Kreiborg S, Takada K. Human masticatory muscle volume and zygomatico-mandibular form in adults with mandibular prognathism. J Dent Res. 2002;81(11):752-6. | Full text  Only Md prognathism |
|  | Kitai N, Kreiborg S, Bakke M, Paulsen HU, Møller E, Darvann TA, et al. Three-dimensional magnetic resonance image of the mandible and masticatory muscles in a case of juvenile chronic arthritis treated with the Herbst appliance. Angle Orthod. 2002;72(1):81-7. | Title |
|  | Kito N, Matsuo K, Ogawa K, Izumi A, Kishima M, Itoda M, Masuda Y. POSITIVE EFFECTS OF "TEXTURED LUNCHES" GATHERINGS AND ORAL EXERCISES COMBINED WITH PHYSICAL EXERCISES ON ORAL AND PHYSICAL FUNCTION IN OLDER INDIVIDUALS: A CLUSTER RANDOMIZED CONTROLLED TRIAL. Journal of Nutrition Health & Aging. 2019;23(7):669-76. | Title |
|  | Kjellberg H, Fasth A, Kiliaridis S, Wenneberg B, Thilander B. Craniofacial structure in children with juvenile chronic arthritis (JCA) compared with healthy children with ideal or postnormal occlusion. American Journal of Orthodontics and Dentofacial Orthopedics. 1995;107(1):67-78. | Title |
|  | Kleine LJ, Mulkern RV, Guttmann CRG, Colucci VM, Jolesz FA. IN-VIVO CHARACTERIZATION OF CYTOTOXIC INTRACELLULAR EDEMA BY MULTICOMPONENT ANALYSIS OF TRANSVERSE MAGNETIZATION DECAY CURVES. Academic Radiology. 1995;2(5):365-72. | Title |
|  | Klemetti E. A review of residual ridge resorption and bone density. Journal of Prosthetic Dentistry. 1996;75(5):512-4. | Title |
|  | Klemetti E, Vainio P, Kröger H. Craniomandibular disorders and skeletal mineral status. Cranio : the journal of craniomandibular practice. 1995;13(2):89-92. | Title |
|  | Klepáček I, Malá PZ. "Bochdalek's" skull: Morphology report and reconstruction of face. Forensic Science, Medicine, and Pathology. 2012;8(4):451-9. | Title |
|  | Klukkert ZS. The Functional Morphology of Ingestion in the Platyrrhine Sclerocarpic Harvesters (Platyrrhini, Primates) [Dissertation/Thesis]2019. | Title |
|  | Knigge RP. Integration of the anthropoid skull: An ontogenetic perspective with insights into jaw fusion. American Journal of Physical Anthropology. 2017;162:249. | Title |
|  | Knoll WD, Gaida A, Maurer P. Analysis of mechanical stress in reconstruction plates for bridging mandibular angle defects. Journal of Cranio-Maxillofacial Surgery. 2006;34(4):201-9. | Title |
|  | Ko EW, Huang CS, Lo LJ, Chen YR. Alteration of masticatory electromyographic activity and stability of orthognathic surgery in patients with skeletal class III malocclusion. J Oral Maxillofac Surg. 2013;71(7):1249-60. | Title |
|  | Ko EWC, Teng TTY, Huang CS, Chen YR. The effect of early physiotherapy on the recovery of mandibular function after orthognathic surgery for class III correction. Part II: Electromyographic activity of masticatory muscles. Journal of Cranio-Maxillofacial Surgery. 2015;43(1):138-43. | Title |
|  | Kobayashi T, Honma K, Shingaki S, Nakajima T. Changes in masticatory function after orthognathic treatment in patients with mandibular prognathism. British Journal of Oral and Maxillofacial Surgery. 2001;39(4):260-5. | Title |
|  | Kober C, Berg BI, Hellmich C, Sader R, Kjeller G. Mandibular finite element analysis after partial alveolar ridge resection due to progressive osteoradionecrosis. International Journal of Computer Assisted Radiology and Surgery. 2016;11(1):S142-S3. | Title |
|  | Kober C, Hayakawa Y, Kinzinger G, Gallo L, Otonari-Yamamoto M, Sano T, Sader RA. 3D-visualization of the temporomandibular joint with focus on the articular disc based on clinical T1-, T2-, and proton density weighted MR images. International Journal of Computer Assisted Radiology and Surgery. 2007;2(3-4):203-10. | Title |
|  | Kober C, Hellmich C, Gurin A, Komlev V, Kjeller G, Sader R, et al. Pathological mandibular biomechanics: Finite element analysis based on partial dentition, cystic lesion, and partial resection. International Journal of Computer Assisted Radiology and Surgery. 2017;12(1):S169-S71. | Title |
|  | Kober C, Hellmich C, Gurin A, Komlev VS, Kjeller G, Sader R, et al. Pathological mandibular biomechanics: Quantitative estimation of pathological fracture risk by finite element analysis. International Journal of Computer Assisted Radiology and Surgery. 2018;13:S116-S7. | Title |
|  | Kober C, Hellmich C, Stübinger S, Zeilhofer HF, Sader R. "Anatomical simulation" of the biomechanical behavior of the human mandible. International Journal of Computerized Dentistry. 2015;18(4):333-42. | Title |
|  | Koca-Ceylan G, Taskaya-Yilmaz N, Guler AU, Incesu L, Aksoz T. The effect of unilateral partial edentulism to muscle thickness. Saudi Medical Journal. 2003;24(12):1352-9. | Title |
|  | Kocaelli H, Balcioglu HA, Erdem TL. Displacement of a maxillary third molar into the buccal space: Anatomical implications apropos of a case. International Journal of Oral and Maxillofacial Surgery. 2011;40(6):650-3. | Title |
|  | Koch R. Detection of Sleep Bruxism with Automated Mandibular Movement Monitoring in Children and Adolescents [Dissertation/Thesis]2023. | Title |
|  | Kochhar A, Byrne PJ. Surgical Management of Complex Midfacial Fractures. Otolaryngologic Clinics of North America. 2013;46(5):759-78. | Title |
|  | Kofod T, Cattaneo PM, Melsen B. Three-dimensional finite element analysis of the mandible and temporomandibular joint on simulated occlusal forces before and after vertical ramus elongation by distraction osteogenesis. Journal of Craniofacial Surgery. 2005;16(3):421-9. | Title |
|  | Kofod T, Nørholt SE, Pedersen TK, Jensen J. Reliability of distraction vector transfer in unilateral vertical distraction of the mandibular ramus. Journal of Craniofacial Surgery. 2005;16(1):15-22. | Title |
|  | Koga S, Sato I, Li ZL, Miyaso H, Kawata S, Itoh M. Analysis of the mylohyoid nerve in elderly Japanese cadavers for dental implant surgery. Clinical and Experimental Dental Research. 2021;7(1):20-32. | Title |
|  | Kohno S, Yoshida K, Kobayashi H. Pain in the sternocleidomastoid muscle and occlusal interferences. Journal of Oral Rehabilitation. 1988;15(4):385-92. | Title |
|  | Kois JC, Phillips KM. Occlusal vertical dimension: alteration concerns. Compendium of continuing education in dentistry (Jamesburg, NJ : 1995). 1997;18(12):1169-74,76. | Title |
|  | Kojima Y, Sendo R, Ohno S, Sugimura M. Ultrasound-guided inferior alveolar nerve block for trismus during dental treatment: a case report. JA Clinical Reports. 2020;6(1). | Title |
|  | Koldas T, Barbek B, Aǧir H, Görgün B. Hemangioma of the masseter muscle: Report of a case. European Journal of Plastic Surgery. 1997;20(4):223-4. | Title |
|  | Konchak PA, Thomas NR, Lanigan DT, Devon RM. Freeway space measurement using mandibular kinesiograph and EMG before and after TENS. Angle Orthodontist. 1988;58(4):343-50. | Title |
|  | Kondo E. Occlusal stability in Class II, Division 1, deep bite cases followed up for many years after orthodontic treatment. American journal of orthodontics and dentofacial orthopedics : official publication of the American Association of Orthodontists, its constituent societies, and the American Board of Orthodontics. 1998;114(6):611-30. | Title |
|  | Kondo E. Features and treatment of skeletal class III malocclusion with severe lateral mandibular shift and asymmetric vertical dimension. World journal of orthodontics. 2004;5(1):9-24. | Title |
|  | Kondo E. Nonextraction and nonsurgical treatment of an adult with skeletal Class II open bite with severe retrognathic mandible and temporomandibular disorders. World journal of orthodontics. 2007;8(3):261-76. | Title |
|  | Kondo E, Arai S. Nonsurgical and nonextraction treatment of a skeletal class III adult patient with severe prognathic mandible. World journal of orthodontics. 2005;6(3):233-47. | Title |
|  | Kong MS, Huh KH, Kho HS. Case series: Noninfectious myositis of temporal muscle: a report of 2 cases. Medicine (United States). 2023;102(25):E34100. | Title |
|  | Konno M, Sato K, Mito T, Mitani H. Relationship between the direction of mandibular growth and masseter muscle conduction velocity. Am J Orthod Dentofacial Orthop. 2005;128(1):35-43; discussion -4. | Full text  Other outcomes |
|  | Kono K, Tanikawa C, Murata Y, Yanagita T, Kamioka H, Yamashiro T. Three-dimensional changes in the craniofacial complex associated with soft-diet feeding. European Journal of Orthodontics. 2020;42(5):509-16. | Title |
|  | Kono K, Tanikawa C, Yanagita T, Kamioka H, Yamashiro T. A Novel Method to Detect 3D Mandibular Changes Related to Soft-Diet Feeding. Frontiers in Physiology. 2017;8. | Title |
|  | Konouchi H, Asaumi JI, Yanagi Y, Hisatomi M, Arita ES, Watanabe PCA, et al. Diagnostic value of MR imaging for dentigerous cysts. Oral Radiology. 2014;30(1):13-9. | Title |
|  | Koolstra JH, Kommers SC, Forouzanfar T. Biomechanical analysis of fractures in the mandibular neck (collum mandibulae). Journal of Cranio-Maxillofacial Surgery. 2014;42(8):1789-94. | Title |
|  | Koolstra JH, Van Eijden TMGJ. A method to predict muscle control in the kinematically and mechanically indeterminate human masticatory system. Journal of Biomechanics. 2001;34(9):1179-88. | Title |
|  | Koolstra JH, Van Eijden TMGJ. Functional significance of the coupling between head and jaw movements. Journal of Biomechanics. 2004;37(9):1387-92. | Title |
|  | Koos B, De Castrillon FS, Ciesielski R, Tzaribachev N. Orofacial anomalies in children with confirmed juvenile idiopathic arthritis. Arthritis and Rheumatism. 2012;64:S499-S500. | Title |
|  | Koparal M, Kucuk AO, Alan H, Asutay F, Avci M. Effects of low-level laser therapy following surgical extraction of the lower third molar with objective measurement of swelling using a three-dimensional system. Experimental and Therapeutic Medicine. 2018;15(4):3820-6. | Title |
|  | Kopecká B, Ravnik D, Jelen K, Bittner V. Objective Methods of Muscle Tone Diagnosis and Their Application-A Critical Review. Sensors. 2023;23(16). | Title |
|  | Kor HS, Yang HJ, Hwang SJ. Relapse of skeletal class III with anterior open bite after bimaxillary orthognathic surgery depending on maxillary posterior impaction and mandibular counterclockwise rotation. Journal of Cranio-Maxillofacial Surgery. 2014;42(5):e230-e8. | Title |
|  | Korfage JAM, Van Eijden T. Regional differences in fibre type composition in the human temporalis muscle. Journal of Anatomy. 1999;194:355-62. | Title |
|  | Korfage JAM, Wang J, Lie S, Langenbach GEJ. Influence of botulinum toxin on rabbit jaw muscle activity and anatomy. Muscle & Nerve. 2012;45(5):684-91. | Title |
|  | Korn HJ. Biofeedback in the treatment of temporomandibular disorders and bruxism. Verhaltenstherapie. 2005;15(2):94-102. | Title |
|  | Kostur BK, Minyaeva VA, Lyubomirova NK. A new technique of measuring the central relationship of jaws with special reference to the activity of maxillofacial muscles. Stomatologiya. 1989;68(4):53-5. | Title |
|  | Kouame P, N'Dindin A, Savane S. Correlations among mandibular structures in black African children. Odonto-stomatologie tropicale = Tropical dental journal. 2003;26(101):7-12. | Title |
|  | Kovalko I, Stoustrup P, Benseler S, Twilt M. Update on temporomandibular joint arthritis in juvenile idiopathic arthritis. Journal of Rheumatology. 2018;45(7):1030. | Title |
|  | Kovero O, Hurmerinta HS, Zepa I, Huggare J, Nissinen M, Könönen M. Maximal bite force and its associations with spinal posture and craniofacial morphology in young adults. Acta Odontologica Scandinavica. 2002;60(6):365-9. | Title |
|  | Koyabu DB, Endo H. Craniofacial variation and dietary adaptations of African colobines. Journal of Human Evolution. 2009;56(6):525-36. | Title |
|  | Koyabu DB, Oshida T, Dang NX, Can DN, Kimura J, Sasaki M, et al. Craniodental mechanics and the feeding ecology of two sympatric callosciurine squirrels in Vietnam. Journal of Zoology. 2009;279(4):372-80. | Title |
|  | Krajewska J, Olczyk T, Roskosz J, Paliczk-Cieślik E, Kukulska A, Śmietana A, et al. Treatment with sorafenib in advanced thyroid cancer - A case report. Endokrynologia Polska. 2010;61(5):492-6. | Title |
|  | Kramer ST, Murat S, Wakely PE. Slow-growing right mandibular mass. JAMA Otolaryngology - Head and Neck Surgery. 2016;142(3):291-2. | Title |
|  | Krane NA, Fagin A, Ghanem TA, Cannady SB, Petrisor D, Wax MK. Simultaneous maxillary and mandibular reconstruction with a single Osteocutaneous fibula free flap: A description of three cases. Microsurgery. 2021;41(1):79-83. | Title |
|  | Krechina EK, Guseva IE, Pogabalo IV, Markov NM, Abdurakhmanova ZU, Rassadina AV. [Modern achievements of the functional diagnostics in dentistry]. Stomatologiia (Mosk). 2022;101(4):30-3. | Title |
|  | Kreiborg S, Bakke M, Kirkeby S, Michler L, Vedtofte P, Seidler B, Møller E. Facial growth and oral function in a case of juvenile rheumatoid arthritis during an 8-year period. Eur J Orthod. 1990;12(2):119-34. | Title |
|  | Kreiborg S, Jensen BL, Møller E, Björk A. Craniofacial growth in a case of congenital muscular dystrophy. A roentgencephalometric and electromyographic investigation. American Journal of Orthodontics. 1978;74(2):207-15. | Title |
|  | Kreiborg S, Moller E, Bjork A. Skeletal and functional craniofacial adaptations in plagiocephaly. Journal of Craniofacial Genetics and Developmental Biology. 1985;5(SUPPL. 1):199-210. | Title |
|  | Kriegbaum RK, Hillerup S. Fibrodysplasia ossificans progressiva (FOP): Report of a case with extra-articular ankylosis of the mandible. Journal of Cranio-Maxillofacial Surgery. 2013;41(8):856-60. | Title |
|  | Kroon RHMJM, Kalf JG, Meijers RL, de Swart BJM, Cameron IGM, Doorduin J, et al. Muscle ultrasound is a sensitive biomarker in oculopharyngeal muscular dystrophy. Muscle and Nerve. 2022. | Title |
|  | Kruse T, Heller R, Wirth B, Glöggler J, Wurster CD, Ludolph AC, Braumann B. Maximum bite force in patients with spinal muscular atrophy during the first year of nusinersen therapy – A pilot study. Acta Myologica. 2020;39(2):83-9. | Title |
|  | Krüsi A, Dritsas K, Kalimeri E, Kloukos D, Gkantidis N. Association of Craniofacial Patterns with the Curve of Spee and the Time Required for Orthodontic Levelling. Dentistry Journal. 2022;10(9). | Title |
|  | Kryeziu K, Prekazi-Loxha M, Hajdari B, Salihu L, Vela-Gaxha Z, Stubljar D, Starc A. Masticatory muscles activity in patients with mandibular angle fractures: A literature review on which procedure to use to reverse the best masticatory muscles functionality. Heliyon. 2023;9(4). | Title |
|  | Kubo K, Kawata T, Ogawa T, Watanabe M, Sasaki K. Outer shape changes of human masseter with contraction by ultrasound morphometry. Archives of Oral Biology. 2006;51(2):146-53. | Abstract |
|  | Kuboki T, Maekawa K, Clark G. Intramuscular haemodynamics using near infra-red spectroscopy as a research strategy to understand chronic muscle pain pathophysiology. Spectroscopy-an International Journal. 2005;19(1):27-36. | Title |
|  | Kuboki T, Suzuki K, Maekawa K, Inoue-Minakuchi M, Acero CO, Yanagi Y, et al. Correlation of the near-infrared spectroscopy signals with signal intensity in T<sub>2</sub>-weighted magnetic resonance imaging of the human masseter muscle. Archives of Oral Biology. 2001;46(8):721-7. | Title |
|  | Kubota M, Nakano H, Sanjo I, Satoh K, Sanjo T, Kamegai T, Ishikawa F. Maxillofacial morphology and masseter muscle thickness in adults. Eur J Orthod. 1998;20(5):535-42. | Included |
|  | Kubota T, Yagi T, Tomonari H, Ikemori T, Miyawaki S. Influence of surgical orthodontic treatment on masticatory function in skeletal Class III patients. J Oral Rehabil. 2015;42(10):733-41. | Title |
|  | Kuć J, Szarejko KD, Gołębiewska M. Comparative evaluation of occlusion before and after soft tissue mobilization in patients with temporomandibular disorder—myofascial pain with referral. International Journal of Environmental Research and Public Health. 2021;18(12). | Title |
|  | Kuftinec MM, Voudouris JC. Ask us. American Journal of Orthodontics and Dentofacial Orthopedics. 2004;125(4):A19. | Title |
|  | Kugimiya Y, Iwasaki M, Ohara Y, Motokawa K, Edahiro A, Shirobe M, et al. Relationship between oral hypofunction and sarcopenia in community-dwelling older adults: The otassha study. International Journal of Environmental Research and Public Health. 2021;18(12). | Title |
|  | Kugimiya Y, Iwasaki M, Ohara Y, Motokawa K, Edahiro A, Shirobe M, et al. Association between sarcopenia and oral functions in community-dwelling older adults: A cross-sectional study. Journal of Cachexia, Sarcopenia and Muscle. 2023;14(1):429-38. | Title |
|  | Kugimoto T, Nishii N, Oikawa Y, Kuroshima T, Hirai H, Tomioka H, et al. Invasion of the bucco-mandibular space by oral squamous cell carcinoma: histopathological analysis of invasion pattern. Frontiers in Oncology. 2023;13. | Title |
|  | Kulkarni AU, Gadre PK, Kulkarni PA, Gadre KS. Diagnosing psoriatic arthritis of the temporomandibular joint: A study in radiographic images. BMJ Case Reports. 2013. | Title |
|  | Kulmer S, Ruzicka B, Niederwanger A, Moschen I. Incline and length of guiding elements in untreated naturally grown dentition. Journal of Oral Rehabilitation. 1999;26(8):650-60. | Title |
|  | Kumai T, Shibukawa Y, Suzuki T. Characteristics of event related potentials elicited by trains of teeth clenching in humans. The Bulletin of Tokyo Dental College. 2000;41(1):1-7. | Title |
|  | Kumar A, Brierley D, Hunter KD, Lee N. Rapidly-growing buccal mass in a 6-month-old infant. British Journal of Oral and Maxillofacial Surgery. 2015;53(9):888-90. | Title |
|  | Kumar A, Castrillon E, Svensson KG, Baad-Hansen L, Trulsson M, Svensson P. Effects of experimental craniofacial pain on fine jaw motor control: a placebo-controlled double-blinded study. Experimental brain research. 2015;233(6):1745‐59. | Title |
|  | Kumar A, Castrillon E, Svensson P. Can Experimentally Evoked Pain in the Jaw Muscles or Temporomandibular Joint Affect Anterior Bite Force in Humans? Journal of Oral & Facial Pain and Headache. 2015;29(1):31-40. | Title |
|  | Kumar A, Singh R, Santhosh M, Vijay S, Surendran N, Sahu GC, et al. Role of structures in the masticator space in selecting patients with resectable T4b oral cancer: findings from a survival analysis. International Journal of Oral and Maxillofacial Surgery. 2021;50(5):579-84. | Title |
|  | Kumar N, Sardana R, Kaur R, Jain A. Intraoperative mandibular nerve block with peripheral nerve stimulator for temporomandibular joint ankylosis. Journal of Clinical Anesthesia. 2016;35:207-9. | Title |
|  | Kumar S, Mokhtar EA, Rattan V, Singh S. Analysis of Maximum Bite Force, Chewing Efficiency and Electromyographic Study of Temporalis and Masseter Muscles in Postoperative Temporomandibular Joint Ankylosis Patients. Journal of Oral and Maxillofacial Surgery. 2020;78(10):e98. | Title |
|  | Kumar VV, Malik NA, Visscher CM, Ebenezer S, Sagheb K, Lobbezoo F. Comparative evaluation of thickness of jaw-closing muscles in patients with long-standing bilateral temporomandibular joint ankylosis: a retrospective case-controlled study. Clin Oral Investig. 2015;19(2):421-7. | Title |
|  | Kummer B. Anatomie und Biomechanik des Unterkiefers. Fortschritte der Kieferorthopädie. 1985;46(5):335-42. | Title |
|  | Kün-Darbois JD, Manero F, Rony L, Chappard D. Contrast enhancement with uranyl acetate allows quantitative analysis of the articular cartilage by microCT: Application to mandibular condyles in the BTX rat model of disuse. Micron. 2017;97:35-40. | Title |
|  | Kundinger KK, Austin BP, Christensen LV, Donegan SJ, Ferguson DJ. An evaluation of temporomandibular joints and jaw muscles after orthodontic treatment involving premolar extractions. American Journal of Orthodontics and Dentofacial Orthopedics. 1991;100(2):110-5. | Title |
|  | Kuntamukkula S, Sinha R, Tiwari PK, Paul D. Dynamic Stability Assessment of the Temporomandibular Joint as a Sequela of Open Reduction and Internal Fixation of Unilateral Condylar Fracture. Journal of Oral and Maxillofacial Surgery. 2018;76(12):2598-609. | Title |
|  | Kupczik K, Dobson CA, Crompton RH, Phillips R, Oxnard CE, Fagan MJ, O'Higgins P. Masticatory Loading and Bone Adaptation in the Supraorbital Torus of Developing Macaques. American Journal of Physical Anthropology. 2009;139(2):193-203. | Title |
|  | Kupczik K, Stark H, Mundry R, Neininger FT, Heidlauf T, Röhrle O. Reconstruction of muscle fascicle architecture from iodine-enhanced microCT images: A combined texture mapping and streamline approach. Journal of Theoretical Biology. 2015;382:34-43. | Title |
|  | Kupers RC, Svensson P, Jensen TS. Central representation of muscle pain and mechanical hyperesthesia in the orofacial region: A positron emission tomography study. Pain. 2004;108(3):284-93. | Title |
|  | Kupferman SB, Schwartz HC. Malposed Teeth in the Pterygomandibular Space: Report of 2 Cases. Journal of Oral and Maxillofacial Surgery. 2008;66(1):167-9. | Title |
|  | Kurabeishi H, Tatsuo R, Makoto N, Kazunori F. Relationship between tongue pressure and maxillofacial morphology in Japanese children based on skeletal classification. Journal of Oral Rehabilitation. 2018;45(9):684-91. | Abstract |
|  | Kuroda K, Saitoh I, Inada E, Takemoto Y, Iwasaki T, Iwase Y, et al. Head motion may help mouth opening in children. Archives of Oral Biology. 2011;56(1):102-7. | Title |
|  | Kuroda M, Otonari-Yamamoto M, Araki K. Evaluation of lateral pterygoid muscles in painful temporomandibular joints by signal intensity on fluid-attenuated inversion recovery images. Oral Radiology. 2018;34(1):17-23. | Title |
|  | Kuroda S, Tanimoto K, Izawa T, Fujihara S, Koolstra JH, Tanaka E. Biomechanical and biochemical characteristics of the mandibular condylar cartilage. Osteoarthritis and Cartilage. 2009;17(11):1408-15. | Title |
|  | Kuster R, Ingervall B. The effect of treatment of skeletal open bite with two types of bite-blocks. Eur J Orthod. 1992;14(6):489-99. | Title |
|  | Kusumah SW, Suzuki S, Itoh K, Higashino R, Ohbayashi N, Kurabayashi T, Moriyama K. Morphological observation of the medial pterygoid muscle by the superimposition of images obtained by lateral cephalogram and MRI. Journal of Orthodontics. 2009;36(4):243-52. | Included |
|  | Kwak ES. Asian cosmetic facial Surgery. Facial Plastic Surgery. 2010;26(2):102-9. | Title |
|  | Kwak YY, Jang I, Choi DS, Cha BK. Functional evaluation of orthopedic and orthodontic treatment in a patient with unilateral posterior crossbite and facial asymmetry. Korean Journal of Orthodontics. 2014;44(3):143-53. | Title |
|  | Kwakwa K, Cannonier S, Vanderburgh J, Granke M, Bullock K, Nyman J, et al. Targeting Gli2 to circumvent anti-EGFR therapeutic resistance in oral squamous cell carcinoma. Journal of Bone and Mineral Research. 2019;34:299. | Title |
|  | Kwiatkowska KK, Bloching M, Flügel W. Case report: Melanoma-the black chameleon. Laryngo- Rhino- Otologie. 2018;97:S53. | Title |
|  | Kwon H, Park SH, Jung HI, Hwang WC, Choi YJ, Chung C, Kim KH. Comparison of the bite force and occlusal contact area of the deviated and non-deviated sides after intraoral vertical ramus osteotomy in skeletal Class III patients with mandibular asymmetry: Two-year follow-up. Korean Journal of Orthodontics. 2022;52(3):172-81. | Title |
|  | Kwon JS, Kim ST, Jeon YM, Choi JH. Effect of botulinum toxin type A injection into human masseter muscle on stimulated parotid saliva flow rate. International Journal of Oral and Maxillofacial Surgery. 2009;38(4):316-20. | Title |
|  | Kwon TG, Lee KH, Park HS, Ryoo HM, Kim HJ, Lee SH. Relationship Between the Masticatory Muscles and Mandibular Skeleton in Mandibular Prognathism With and Without Asymmetry. Journal of Oral and Maxillofacial Surgery. 2007;65(8):1538-43. | Full text  Prognathism & asymmetries |
|  | Kwon TG, Park HS, Lee SH, Park IS, An CH. Influence of unilateral masseter muscle atrophy on craniofacial morphology in growing rabbits. Journal of Oral and Maxillofacial Surgery. 2007;65(8):1530-7. | Title |
|  | La Croix S, Zelditch ML, Shivik JA, Lundrigan BL, Holekamp KE. Ontogeny of feeding performance and biomechanics in coyotes. Journal of Zoology. 2011;285(4):301-15. | Title |
|  | Lacouture CY. Effects of functional appliances on the temporomandibular joint and masticatory muscles in Macaca fascicularis [Dissertation/Thesis]1991. | Title |
|  | Lagarde M, Knuijt S, Groothuis J, De Groot I, Van Den Engel-Hoek L. Longitudinal changes in oral and masticatory muscles in duchenne muscular dystrophy: A disturbed balance. Dysphagia. 2017;32(1):142. | Title |
|  | Lagarde MLJ, van Alfen N, Geurts ACH, de Groot IJM, van den Engel-Hoek L. Orofacial muscles may be affected in early stages of Becker muscular dystrophy: A preliminary study. Muscle and Nerve. 2020;61(2):213-7. | Title |
|  | Laguna L, Sarkar A, Chen J. Assessment of eating capability of elderly subjects in UK: A quantitative evaluation. Proceedings of the Nutrition Society. 2015;74(OCE2). | Title |
|  | Lam EWN, Hannam AG, Wood WW, Fache JS, Watanabe M. Imaging orofacial tissues by magnetic resonance. Oral Surgery Oral Medicine and Oral Pathology. 1989;68(1):2-8. | Title |
|  | Lamey PJ, Burnett CA, Fartash L, Clifford TJ, McGovern JM. Migraine and masticatory muscle volume, bite force, and craniofacial morphology. Headache. 2001;41(1):49-56. | Title |
|  | Landi F, Barraclough J, Evteev A, Anikin A, Satanin L, O'Higgins P. The role of the nasal region in craniofacial growth: An investigation using path analysis. Anatomical Record. 2022;305(8):1892-909. | Title |
|  | Langenbach G, van de Pavert S, Savalle W, Korfage H, van Eijden T. Influence of food consistency on the rabbit masseter muscle fibres. European Journal of Oral Sciences. 2003;111(1):81-4. | Title |
|  | Langenbach GEJ, Weijs WA, Koolstra JH. BIOMECHANICAL CHANGES IN THE RABBIT MASTICATORY SYSTEM DURING POSTNATAL-DEVELOPMENT. Anatomical Record. 1991;230(3):406-16. | Title |
|  | Langenbach GEJ, Zhang F, Herring SW, Hannam AG. Modelling the masticatory biomechanics of a pig. Journal of Anatomy. 2002;201(5):383-93. | Title |
|  | Langevin CJ, Hanasono MM, Riina HA, Stieg PE, Spinelli HM. Lateral transzygomatic approach to sphenoid wing meningiomas. Neurosurgery. 2010;67(SUPPL. 2):ons377-ons83. | Title |
|  | Langrodi SSR, Goudarzi F, Stanbouly D. Etiology of Tinnitus on CT and CBCT: A Narrative Review. International Tinnitus Journal. 2022;26(2):95-100. | Title |
|  | Lauriti L, De Cerqueira Luz JG, Agnelli Mesquita-Ferrari R, Fernandes KPS, Deana AM, Tempestini Horliana ACR, et al. Evaluation of the Effect of Phototherapy in Patients with Mandibular Fracture on Mandibular Dynamics, Pain, Edema, and Bite Force: A Pilot Study. Photomedicine and Laser Surgery. 2018;36(1):24-30. | Title |
|  | Lauweryns I, Carels C, Marchal G, Bellon E, Hermans R, Vlietinck R. Magnetic resonance imaging of the masseter muscle: A preliminary genetic study in monozygotic and dizygotic twins. Journal of Craniofacial Genetics and Developmental Biology. 1995;15(1):26-34. | Title |
|  | Laviv A, Sadow PM, Keith DA. Pseudogout in the temporomandibular joint with imaging, arthroscopic, operative, and pathologic findings. Report of an unusual case. Journal of Oral and Maxillofacial Surgery. 2015;73(6):1106-12. | Title |
|  | Law CJ, Mehta RS. Dry versus wet and gross: Comparisons between the dry skull method and gross dissection in estimations of jaw muscle cross-sectional area and bite forces in sea otters. Journal of Morphology. 2019;280(11):1706-13. | Title |
|  | Lawler ME, Hansen GM, Williams WB, Susarla SM, Faquin WC, Troulis MJ, Kaban LB. Serial Histologic and Immunohistochemical Changes in Anterior Digastric Myocytes in Response to Distraction Osteogenesis. Journal of Oral and Maxillofacial Surgery. 2012;70(1):168-78. | Title |
|  | Le Révérend BJD, Edelson LR, Loret C. Anatomical, functional, physiological and behavioural aspects of the development of mastication in early childhood. British Journal of Nutrition. 2014;111(3):403-14. | Title |
|  | Learreta JA, Moses AJ. Cephalometric variation in patients with and without intraoral neuromuscular repositioning appliance. Journal of general orthodontics. 1999;10(2):14-21. | Title |
|  | Leboulanger N, Picard A, Roger G, Garabedian EN. Fetal rhabdomyoma of the infratemporal fossa in children. European Annals of Otorhinolaryngology, Head and Neck Diseases. 2010;127(1):30-2. | Title |
|  | Ledogar JA. Human feeding biomechanics: Intraspecific variation and evolution [Dissertation/Thesis]2015. | Title |
|  | Ledogar JA, Dechow PC, Wang Q, Gharpure PH, Gordon AD, Baab KL, et al. Human feeding biomechanics: Performance, variation, and functional constraints. PeerJ. 2016;2016(7). | Title |
|  | Lee C, Kang M, Kim T, Kim JY, Huh JK. Relationship Between Temporal and Masseter Muscle Status and Skeletal Patterns With Facial Asymmetry. Journal of Oral and Maxillofacial Surgery. 2023;81(9):S84-S5. | Abstract |
|  | Lee DH, Yu HS. Masseter muscle changes following orthognathic surgery: a long-term three-dimensional computed tomography follow-up. Angle Orthod. 2012;82(5):792-8. | Title |
|  | Lee EJ, Hwang HJ, Byeon HK, Park HS, Choi HS. A low grade fibromyxoid sarcoma originating from the masseter muscle: A case report. Journal of Medical Case Reports. 2015;9(1). | Title |
|  | Lee HH, Kim ST, Lee KJ, Baik HS. Effect of a second injection of botulinum toxin on lower facial contouring, as evaluated using 3-dimensional laser scanning. Dermatologic Surgery. 2015;41(4):439-44. | Title |
|  | Lee JH, Kim SM, Kim MJ. Three-dimensional analysis of airway anatomy in patients with mandibular anterior arch reconstruction. International Journal of Oral and Maxillofacial Surgery. 2017;46:31-2. | Title |
|  | Lee JK, Lim SC. Intramuscular hemangiomas of the mylohyoid and sternocleidomastoid muscle. Auris Nasus Larynx. 2005;32(3):323-7. | Title |
|  | Lee JS, Kang SH. Direct transparotid approach via a modified mini-preauricular incision for open reduction and internal fixation of subcondylar fractures. Journal of the Korean Association of Oral and Maxillofacial Surgeons. 2021;47(4):327-34. | Title |
|  | Lee JY, Kim DJ, Lee SG, Chung JW. A longitudinal study on the osteoarthritic change of the temporomandibular joint based on 1-year follow-up computed tomography. Journal of Cranio-Maxillofacial Surgery. 2012;40(8):e223-e8. | Title |
|  | Lee LA, Karabina A, Broadwell LJ, Leinwand LA. The ancient sarcomeric myosins found in specialized muscles. Skeletal Muscle. 2019;9(1). | Title |
|  | Lee PH, Chen JJ, Tsou YA. A recurrent sialolipoma of the parotid gland: A case report. Oncology Letters. 2014;7(6):1981-3. | Title |
|  | Lee SH, Koh KS, Song WC. Asymmetric Protrusion of the Midface in Young Adults. Journal of Craniofacial Surgery. 2018;29(8):2353-7. | Title |
|  | Lee WB, Hwang DS, Kim UK. Sequential treatment from mandibulectomy to reconstruction on mandibular oral cancer - Case review I: Mandibular ramus and angle lesion of primary intraosseous squamous cell carcinoma. Journal of the Korean Association of Oral and Maxillofacial Surgeons. 2021;47(2):120-7. | Title |
|  | Lee YK, Moon HJ. Reciprocal influence of masticatory apparatus, craniofacial structure and whole body homeostasis. Medical Hypotheses. 2012;79(6):761-6. | Title |
|  | Lehman H, Nitzan DW. Response to the letter: Limited mouth opening of unknown cause cured by diagnostic coronoidectomy: A new clinical entity? British Journal of Oral and Maxillofacial Surgery. 2015;53(5):477. | Title |
|  | Leiggener CS, Erni S, Gallo LM. Novel approach to the study of jaw kinematics in an alloplastic TMJ reconstruction. International Journal of Oral and Maxillofacial Surgery. 2012;41(9):1041-5. | Title |
|  | Leiser Y, Peled M, Braun R, Abu-El Naaj I. Treatment of low subcondylar fractures - A 5-year retrospective study. International Journal of Oral and Maxillofacial Surgery. 2013;42(6):716-20. | Title |
|  | Leiser Y, Shilo D, Wolff A, Rachmiel A. Functional Reconstruction in Mandibular Avulsion Injuries. J Craniofac Surg. 2016;27(8):2113-6. | Title |
|  | Lekroengsin B, Tachiki C, Takaki T, Nishii Y. Relationship between Changes in Condylar Morphology and Masticatory Muscle Volume after Skeletal Class II Surgery. Journal of Clinical Medicine. 2023;12(14). | Title |
|  | Lenguas L, Alarcón JA, Venancio F, Kassem M, Martín C. Surface electromyographic evaluation of jaw muscles in children with unilateral crossbite and lateral shift in the early mixed dentition. Sexual dimorphism. Medicina Oral, Patologia Oral y Cirugia Bucal. 2012;17(6):e1096-e102. | Title |
|  | Leonard KC, Boettcher ML, Dickinson E, Herrel A, Hartstone-Rose A. The Ontogeny of Masticatory Muscle Architecture in Microcebus murinus. FASEB Journal. 2019;33(SUPPL 1):615.6. | Title |
|  | Lerman MD. A revised view of the dynamics, physiology, and treatment of occlusion: A new paradigm. Cranio-the Journal of Craniomandibular & Sleep Practice. 2004;22(1):50-63. | Title |
|  | Leung DK, Hägg U. An electromyographic investigation of the first six months of progressive mandibular advancement of the Herbst appliance in adolescents. Angle Orthod. 2001;71(3):177-84. | Title |
|  | Levenson E, Morgan W. A case of an intramuscular hemangioma of the muscles of mastication. Journal of Investigative Medicine. 2022;70(2):597-8. | Title |
|  | Lewis K, Collyer J, Coombes D. The use of CT Navigation in the removal of foreign bodies in the head and neck region. British Journal of Oral and Maxillofacial Surgery. 2012;50:S4. | Title |
|  | Li B. Numerical simulation of mandibular distraction osteogenesis and malformed microtia auditory rehabilitation. 2011. | Title |
|  | Li C, Yang C, Qiu W, Qiu Y, Jiang Q, Chen M. Myositis ossificans of the masticatory muscle monitored over three generations: A case report and review of the literature. Experimental and Therapeutic Medicine. 2020;19(4):2622-6. | Title |
|  | Li DZ, Zhao Y, Xin JB, Yan M, Li ZJ, Hu SM. Application of fence locator in CT-guided radiofrequency thermal coagulation for trigeminal neuralgia. Journal of Interventional Radiology (China). 2016;25(8):686-8. | Title |
|  | Li HC, Li DM, Zhang ZY, Lü CS, Liu YF, Zhang J, Gui L. 3-dimensional CT cephalometry before and after mandibular angle osteotomy and its clinical significance. Zhonghua zheng xing wai ke za zhi = Zhonghua zhengxing waike zazhi = Chinese journal of plastic surgery. 2008;24(3):199-202. | Title |
|  | Li HT, Cui CJ, Lu SL, He KY. Study on the association of ultrasonographic thickness and electromyographic activity of masseter muscle in young females with different vertical craniofacial morphology. Shanghai Kou Qiang Yi Xue. 2008;17(5):529-34. | Included |
|  | Li J, Chen J, Zheng G, Liao G, Fu Z, Li J, et al. Digital subtraction angiography-guided percutaneous sclerotherapy of venous malformations with pingyangmycin and/or absolute ethanol in the maxillofacial region. Journal of Oral and Maxillofacial Surgery. 2010;68(9):2258-66. | Title |
|  | Li X, Feng X, Li J, Bao X, Xu J, Lin J. Can Botulinum Toxin-A Contribute to Reconstructing the Physiological Homeostasis of the Masticatory Complex in Short-faced Patients during Occlusal Therapy? A Prospective Pilot Study. Toxins. 2022;14(6). | Title |
|  | Li Y, Li H, Lai Q, Xue R, Zhu K, Deng Y. Finite element analysis of 3D-printed personalized titanium plates for mandibular angle fracture. Computer Methods in Biomechanics and Biomedical Engineering. 2023;26(1):78-89. | Title |
|  | Li Y, Wu P, Liu S, Tang M, Yu S, Kikkawa DO, Lu W. Finite Element Analysis of 2- and 3-Point Internal Fixation Methods for the Treatment of Zygomaticomaxillary Complex Fracture. J Craniofac Surg. 2020;31(8):2208-12. | Title |
|  | Li Y, Wu P, Ma Y, Tang M, Zeng X, Tang Y, et al. Stability evaluation of different internal fixation methods for zygomaticomaxillary complex fractures by finite element biomechanical analysis. Chinese Journal of Experimental Ophthalmology. 2020;38(11):916-22. | Title |
|  | Lieb G. Untersuchungen über die Variationen im Aufbau des Gesichtsschädels und ihren korrelativen Zusammenhang mit der individuellen Form des Kauorgans bei Gebißfehlbildungen. Fortschritte der Kieferorthopädie. 1966;27(3):276-326. | Title |
|  | Lim D, Beitzel F, Lynch G, Woods MG. Myosin heavy chain isoform composition of human masseter muscle from subjects with different mandibular plane angles. Australian orthodontic journal. 2006;22(2):105-14. | Abstract |
|  | Lim M, Nascimento TD, Kim DJ, Ellingrod VL, DaSilva AF. Aberrant Brain Signal Variability and COMT Genotype in Chronic TMD Patients. J Dent Res. 2021;100(7):714-22. | Title |
|  | Lim S, Baek HJ, Kang YH. A Case Report of Primary Extranodal Diffuse Large B-Cell Lymphoma Involving the Masseter Muscle: Histological-Radiological Correlation. Current Medical Imaging. 2023;19(7):788-94. | Title |
|  | Limonta E, Arienti C, Rampichini S, Venturelli M, Emiliano CÈ, Veicsteinas A, Esposito F. Effects of two different self-adapted occlusal splints on electromyographic and force parameters during elbow flexors isometric contraction. Journal of Strength and Conditioning Research. 2018;32(1):230-6. | Title |
|  | Lin CS, Liu LK, Lee WJ, Peng LN, Lin CP, Lee SY, Chen LK. Low masseter muscle mass is associated with frailty in community-dwelling older adults: I-Lan Longitudinal Aging Study. Experimental Gerontology. 2022;163. | Title |
|  | Lin CS, Wu CY, Wang DH, Lin HH, Lo KC, Lo WL, et al. Brain signatures associated with swallowing efficiency in older people. Experimental Gerontology. 2019;115:1-8. | Title |
|  | Lin S, Guo R, Liu K, Mi H, Wang M, Fu H, Li R. Clinical analysis of 11 patients with neuroendocrine carcinoma in maxillofacial region. Chinese Journal of Stomatology. 2023;58(2):151-7. | Title |
|  | Linderholm H, Lindqvist B, Ringqvist M, Wennström A. Isometric bite force in children and its relation to body build and general muscle force. Acta Odontologica Scandinavica. 1971;29(5):563-8. | Title |
|  | Lindsey CA, English JD. Orthodontic treatment and masticatory muscle exercises to correct a Class I open bite in an adult patient. American Journal of Orthodontics and Dentofacial Orthopedics. 2003;124(1):91-8. | Title |
|  | Lindström I, Protto S, Khan N, Väärämäki S, Oksala N, Hernesniemi J. Statin use, development of sarcopenia, and long-term survival after endovascular aortic repair. Journal of Vascular Surgery. 2021;74(5):1651-+. | Title |
|  | Linsen SS, Reich RH, Teschke M. Pressure pain threshold and oral health-related quality of life implications of patients with alloplastic temporomandibular joint replacement - A prospective study. Journal of Oral and Maxillofacial Surgery. 2012;70(11):2531-42. | Title |
|  | Linsen SS, Schön A, Mercuri LG, Teschke M. How Does a Unilateral Temporomandibular Joint Replacement Affect Bilateral Masseter and Temporalis Muscle Activity?-A Prospective Study. Journal of Oral and Maxillofacial Surgery. 2021;79(2):314-23. | Title |
|  | Liokatis P, Tzortzinis G, Gerasimidis S, Smolka W. Application of the lambda plate on condylar fractures: Finite element evaluation of the fixation rigidity for different fracture patterns and plate placements. Injury. 2022;53(4):1345-52. | Title |
|  | Lione R, Franchi L, Noviello A, Bollero P, Fanucci E, Cozza P. Three-dimensional evaluation of masseter muscle in different vertical facial patterns: a cross-sectional study in growing children. Ultrason Imaging. 2013;35(4):307-17. | Included |
|  | Lione R, Kiliaridis S, Noviello A, Franchi L, Antonarakis GS, Cozza P. Evaluation of masseter muscles in relation to treatment with removable bite-blocks in dolichofacial growing subjects: a prospective controlled study. American journal of orthodontics and dentofacial orthopedics. 2017;151(6):1058‐64. | Abstract |
|  | Lipphaus A, Witzel U. Three-dimensional finite element analysis of the dural folds and the human skull under head acceleration. Anatomical Record. 2021;304(2):384-92. | Title |
|  | Litt RA. Relapse after total mandibular advancement: A possible solution. Angle Orthodontist. 1978;48(4):262-73. | Title |
|  | Liu EY, Sommer M, Fan B, Ng B, Mastick J, Shepherd JA. 3D optical models for predicting osteopenia, sarcopenia, and obesity status in women. Journal of Clinical Densitometry. 2018;21(1):33-4. | Title |
|  | Liu J, Jin ZL, Li Q. Effect of occlusal hypofunction and its recovery on the three-dimensional architecture of mandibular alveolar bone in growing rats. Journal of Surgical Research. 2015;193(1):229-36. | Title |
|  | Liu X, Cai HX, Cao PY, Feng Y, Jiang HH, Liu L, et al. TLR4 contributes to the damage of cartilage and subchondral bone in discectomy-induced TMJOA mice. Journal of Cellular and Molecular Medicine. 2020;24(19):11489-99. | Title |
|  | Liu YF, Wang YL, Zuo YP, Sun Q, Wei J, Zhao LX. Structural changes of the temporomandibular joint in adolescents with skeletal class III malocclusions after maxillary protraction: An X-ray measurement analysis. Chinese Journal of Tissue Engineering Research. 2021;25(8):1154-9. | Title |
|  | Liu YH, Ma YX, Hu J, Gao GD, Wu YK, Zhang ZY. Features of facioscapulohumeral muscular dystrophy in oral and maxillofacial region and MRI analysis of facial muscles. Zhonghua kou qiang yi xue za zhi = Zhonghua kouqiang yixue zazhi = Chinese journal of stomatology. 2016;51(12):739-45. | Title |
|  | Liu YH, Yang XJ, Gao XH, Li Y. [Magnetic resonance imaging assessment of the lateral pterygoid muscle in Class III malocclusion subjects]. Zhonghua kou qiang yi xue za zhi = Zhonghua kouqiang yixue zazhi = Chinese journal of stomatology. 2012;47(1):6-9. | Abstract |
|  | Liu Z, Shu J, Zhang Y, Fan Y. The Biomechanical Effects of Sagittal Split Ramus Osteotomy on Temporomandibular Joint. Computer Methods in Biomechanics and Biomedical Engineering. 2018;21(11):617-24. | Title |
|  | Liu ZF, Liu TC, Cai JH, Wu GF, Wang GY, Wang Y, et al. Quantitative magnetic resonance imaging assessment of brain injury after successful cardiopulmonary resuscitation in a rat model of asphyxia cardiac arrest. Brain Imaging and Behavior. 2022;16(1):270-80. | Title |
|  | Liu ZF, Wang DH, Sun XC, Wang JJ, Hu L, Li H, Dai PD. The site of origin and expansive routes of juvenile nasopharyngeal angiofibroma (JNA). International Journal of Pediatric Otorhinolaryngology. 2011;75(9):1088-92. | Title |
|  | Liu ZJ, Yamagata K, Kuroe K, Suenaga S, Noikura T, Ito G. Morphological and positional assessments of TMJ components and lateral pterygoid muscle in relation to symptoms and occlusion of patients with temporomandibular disorders. Journal of Oral Rehabilitation. 2000;27(10):860-74. | Title |
|  | Lo DS, Oliveira LR, Gilio A. A rare case of mandibular osteomyelitis of odontogenic origin in a healthy adolescent. Pediatrics. 2020;146(1):395-7. | Title |
|  | Lo LJ, Mardini S, Chen YR. Volumetric change of the muscles of mastication following resection of mandibular angles: A long-term follow-up. Annals of Plastic Surgery. 2005;54(6):615-21. | Title |
|  | Lobbezoo F, Drangsholt M, Peck C, Sato H, Kopp S, Svensson P, editors. Topical review: New insights into the pathology and diagnosis of disorders of the temporomandibular joint. Journal of Orofacial Pain; 2004. | Title |
|  | Lobbezoo F, Naeije M. Bruxism is mainly regulated centrally, not peripherally. Journal of Oral Rehabilitation. 2001;28(12):1085-91. | Title |
|  | Lombardo D, Coqueugniot H, Colard T. Ontogeny of human cranial vault microstructure. American Journal of Physical Anthropology. 2019;168:145-6. | Title |
|  | Lopes M, Fontao L, Santos C, Alves JE, Coelho A, Peixoto C, et al. Extraocular muscles thickening: The hidden rarity. European Journal of Neurology. 2015;22:530. | Title |
|  | Lorenzini G, Picciotti M, Di Vece L, Pepponi E, Brindisi L, Vessio V, et al. Cervical necrotizing fasciitis of odontogenic origin involving the temporal region - A case report. Journal of Cranio-Maxillofacial Surgery. 2011;39(8):570-3. | Title |
|  | Loukas M, Kapos T, Louis Jr RG, Wartman C, Jones A, Hallner B. Gross anatomical, CT and MRI analyses of the buccal fat pad with special emphasis on volumetric variations. Surgical and Radiologic Anatomy. 2006;28(3):254-60. | Title |
|  | Loureiro RM, Collin J, Sumi DV, Araújo LC, Murakoshi RW, Gomes RLE, Daniel MM. Postoperative CT findings of orthognathic surgery and its complications: A guide for radiologists. Journal of Neuroradiology. 2022;49(1):17-32. | Title |
|  | Lous I, Olesen J. Evaluation of pericranial tenderness and oral function in patients with common migraine, muscle contraction headache and 'combination headache'. Pain. 1982;12(4):385-93. | Title |
|  | Lovald ST, Wagner JD, Baack B. Biomechanical Optimization of Bone Plates Used in Rigid Fixation of Mandibular Fractures. Journal of Oral and Maxillofacial Surgery. 2009;67(5):973-85. | Title |
|  | Lowe AA. Correlations between orofacial muscle activity and craniofacial morphology in a sample of control and anterior open-bite subjects. Am J Orthod. 1980;78(1):89-98. | Full text  Other outcomes |
|  | Lowe AA, Takada K. Associations between anterior temporal, masseter, and orbicularis oris muscle activity and craniofacial morphology in children. Am J Orthod. 1984;86(4):319-30. | Full text  Other outcomes |
|  | Lowe AA, Takada K, Taylor LM. Muscle activity during function and its correlation with craniofacial morphology in a sample of subjects with Class II, Division 1 malocclusions. Am J Orthod. 1983;84(3):204-11. | Full text  Only Class II/1 |
|  | Lucas BD, Barbosa TD, Castelo PM, Gaviao MBD. Influence of anthropometry, TMD, and sex on molar bite force in adolescents with and without orthodontic needs. Journal of Orofacial Orthopedics-Fortschritte Der Kieferorthopadie. 2017;78(6):487-93. | Title |
|  | Lucas BL, Barbosa TS, Pereira LJ, Gavião MBD, Castelo PM. Electromyographic evaluation of masticatory muscles at rest and maximal intercuspal positions of the mandible in children with sleep bruxism. European Archives of Paediatric Dentistry. 2014;15(4):269-74. | Title |
|  | Luderman LN, Michaels MT, Levic DS, Knapik EW. Zebrafish Erc1b mediates motor innervation and organization of craniofacial muscles in control of jaw movement. Developmental Dynamics. 2023;252(1):104-23. | Title |
|  | Lüdinghausen M, Kageyama I, Miura M, Aikhatib M. Morphological peculiarities of the deep infratemporal fossa in advanced age. Surgical and Radiologic Anatomy. 2006;28(3):284-92. | Title |
|  | Luhr HG. Indications for use of a microsystem for internal fixation in craniofacial surgery. J Craniofac Surg. 1990;1(1):35-52. | Title |
|  | Lyons AJ, Crichton S, Pezier T. Trismus following radiotherapy to the head and neck is likely to have distinct genotype dependent cause. Oral Oncology. 2013;49(9):932-6. | Title |
|  | Lyons MF, Aggarwal A. Relaxation rate in the assessment of masseter muscle fatigue. Journal of Oral Rehabilitation. 2001;28(2):174-9. | Title |
|  | Lyons MF, Baxendale RH. MASSETER MUSCLE-RELAXATION RATE IN VOLUNTEERS WITH A MYOGENOUS CRANIOMANDIBULAR DISORDER. Journal of Oral Rehabilitation. 1995;22(5):355-64. | Title |
|  | M.S.P.S S. Bite Force Evaluation in Patients Treated with Titanium Conventional Plating System and Titanium Locking Plating System for Mandibular Fractures [Dissertation/Thesis]2009. | Title |
|  | Ma L, Qi X, Qin J, Zhong S, Zhang B, Zhang Y, Xia H. Effects of the closing and opening muscle groups on jaw condyle biomechanics after prominent mandibular angle osteotomy. Journal of Cranio-Maxillofacial Surgery. 2013;41(5):408-11. | Title |
|  | Maby A, Guay B, Thuot F. Infantile myofibromatosis treated by mandibulectomy and staged reconstruction with submental flap and free fibula flap: A case report. Journal of Otolaryngology - Head and Neck Surgery. 2019;48(1). | Title |
|  | MacDonald JWC, Hannam AG. Relationship between occlusal contacts and jaw-closing muscle activity during tooth clenching: Part I. The Journal of Prosthetic Dentistry. 1984;52(5):718-29. | Title |
|  | Machida N, Yamada K, Takata Y, Yamada Y. Relationship between facial asymmetry and masseter reflex activity. Journal of Oral and Maxillofacial Surgery. 2003;61(3):298-303. | Full text  Other outcomes |
|  | Macho GA. Variation in enamel thickness and cusp area within human maxillary molars and its bearing on scaling techniques used for studies of enamel thickness between species. Archives of Oral Biology. 1994;39(9):783-92. | Title |
|  | Macho GA, Spears IR. Effects of loading on the biochemical behavior of molars of <i>Homo, Pan</i>, and <i>Pongo</i>. American Journal of Physical Anthropology. 1999;109(2):211-27. | Title |
|  | MacIntosh RB, Khan F, Waligora BM. Chondrosarcoma of the temporomandibular disc: Behavior over a 28-year observation period. Journal of Oral and Maxillofacial Surgery. 2015;73(3):465-74. | Title |
|  | Mackool RJ, Hopper RA, Grayson BH, Holliday R, McCarthy JG. Volumetric change of the medial pterygoid following distraction osteogenesis of the mandible: An example of the associated soft-tissue changes. Plastic and Reconstructive Surgery. 2003;111(6):1804-7. | Title |
|  | Madsen H. What's new on the dental scene? Browsing through the dental literature. Journal of Orofacial Orthopedics. 2003;64(1):1-5. | Title |
|  | Maeda A, Soejima K, Ogura M, Ohmure H, Sugihara K, Miyawaki S. Orthodontic treatment combined with mandibular distraction osteogenesis and changes in stomatognathic function. Angle Orthod. 2008;78(6):1125-32. | Title |
|  | Maezawa H, Hirata M, Yoshida K. Neurophysiological Basis of Deep Brain Stimulation and Botulinum Neurotoxin Injection for Treating Oromandibular Dystonia. Toxins. 2022;14(11). | Title |
|  | Maffei C, Garcia P, De Biase NG, De Souza Camargo E, Vianna-Lara MS, Grégio AMT, Azevedo-Alanis LR. Orthodontic intervention combined with myofunctional therapy increases electromyographic activity of masticatory muscles in patients with skeletal unilateral posterior crossbite. Acta Odontologica Scandinavica. 2014;72(4):298-303. | Title |
|  | Magalhaes IB, Pereira LJ, Marques LS, Gameiro GH. The influence of malocclusion on masticatory performance <i>A systematic review</i>. Angle Orthodontist. 2010;80(5):981-7. | Title |
|  | Mahathi N, Azariah E, Ravindran C. Finite element analysis comparison of plate designs in managing fractures involving the mental foramen. Craniomaxillofacial Trauma and Reconstruction. 2013;6(2):93-8. | Title |
|  | Mahony D. Refining occlusion with muscle balance to enhance long-term orthodontic stability. International journal of orthodontics (Milwaukee, Wis). 2004;15(4):11-6. | Title |
|  | Maj G. Development of class II, division 2 malocclusion from mixed dentition to permanent dentition. Bulletin de l"Academie de chirurgie dentaire. 1981(27):71-6. | Title |
|  | Makhlynets N, Prots H, Pantus A, Ozhogan Z, Plaviuk L. THE EXISTENCE OF A FUNCTIONAL MATRIX IN THE DEVELOPMENT OF THE FACIAL SKELETON IN CHILDREN. Georgian Med News. 2023(334):125-32. | Title |
|  | Makhlynets NP, Ozhogan ZR, Soldatyuk VM, Pyuryk MV. IMPROVING THE QUALITY OF DIAGNOSIS WITH MAXILLOMANDIBULAR ANOMALIES IN THE BACKGROUND OF CHRONIC HABITS. World of Medicine and Biology. 2022;81(3):104-8. | Title |
|  | Maki K, Inou N, Takanishi A, Miller AJ. Computer-assisted simulations in orthodontic diagnosis and the application of a new cone beam X-ray computed tomography. Orthod Craniofac Res. 2003;6 Suppl 1:95-101; discussion 79-82. | Title |
|  | Maki K, Inou N, Takanishi A, Miller AJ. Modeling of structure, quality, and function in the orthodontic patient. Orthodontics and Craniofacial Research. 2003;6(SUPPL1):52-8. | Title |
|  | Maki K, Miller AJ, Okano T, Hatcher D, Yamaguchi T, Hobayashi H, Shibasaki Y. Cortical bone mineral density in asymmetrical mandibles: a three-dimensional quantitative computed tomography study. European Journal of Orthodontics. 2001;23(3):217-32. | Full text  Other outcomes |
|  | Maki K, Miller AJ, Okano T, Shibasaki Y. A three-dimensional, quantitative computed tomographic study of changes in distribution of bone mineralization in the developing human mandible. Archives of Oral Biology. 2001;46(7):667-78. | Title |
|  | Maloul A, Regev E, Whyne CM, Beek M, Fialkov JA. In vitro quantification of strain patterns in the craniofacial skeleton due to masseter and temporalis activities. Journal of Craniofacial Surgery. 2012;23(5):1529-34. | Title |
|  | Mandel L. Intramuscular hemangioma with phleboliths or choristoma? [2]. Journal of Oral and Maxillofacial Surgery. 2001;59(12):1511-2. | Title |
|  | Manfredini D. Etiopathogenesis of disk displacement of the temporomandibular joint: A review of the mechanisms. Indian Journal of Dental Research. 2009;20(2):212-21. | Title |
|  | Mangal U, Park JH, Lim SH, Choi SK, Sung JH, Chae JM. Control of the occlusal plane in orthognathic surgery. AJO-DO Clinical Companion. 2021;1(1):43-54. | Title |
|  | Mangilli LD, Sassi FC, Sernik RA, Tanaka C, Andrade CR. Electromyographic and ultrasonographic characterization of masticatory function in individuals with normal occlusion. J Soc Bras Fonoaudiol. 2012;24(3):211-7. | Abstract |
|  | Mani V, George A, Keshava PY, Puthanveedu RK. Pterygoid plate disjunction: Minimally invasive treatment for internal derangement of the temporomandibular joint. Asian Journal of Oral and Maxillofacial Surgery. 2005;17(4):247-55. | Title |
|  | Manns A, Rojas V, Van Diest N, Rojas D, Sobral C. Comparative study of molar and incisor bite forces regarding deciduous, mixed, and definitive dentition. Cranio-the Journal of Craniomandibular & Sleep Practice. 2022;40(4):373-80. | Title |
|  | Manns A, Valdivieso C, Rojas V, Valdés C, Ramírez V. Comparison of clinical and electromyographic rest vertical dimensions in dolichofacial and brachyfacial young adults: A cross-sectional study. Journal of Prosthetic Dentistry. 2018;120(4):513-9. | Title |
|  | Manoj M, Mathew L, Natarajan S, Yellapurkar S, Shetty S, Denny C, Dahal S. Morphometric anlaysis of mandibular coronoid, condyle and sigmoid shape using panoromic view for personal identification in south Indian population. Journal of Clinical Imaging Science. 2022;12. | Title |
|  | Manrikyan GE, Vardanyan IF, Markaryan MM, Manrikyan ME, Badeyan EH, Manukyan AH, et al. Association between the Obstructive Sleep Apnea and Cephalometric Parameters in Teenagers. Journal of Clinical Medicine. 2023;12(21). | Title |
|  | Manson JD. Bone morphology and bone loss in periodontal disease. Journal of Clinical Periodontology. 1976;3(1):14-22. | Title |
|  | Manzano D, Silván A, Saez J, Moreno JC. Myositis ossificans of the temporalis muscle. Case report. Medicina Oral, Patologia Oral y Cirugia Bucal. 2007;12(4):220-3. | Title |
|  | Marchetti C, Bianchi A, Mazzoni S, Cipriani R, Campobassi A. Oromandibular reconstruction using a fibula osteocutaneous free flap: Four different "preplating" techniques. Plastic and Reconstructive Surgery. 2006;118(3):643-51. | Title |
|  | Marewski M, Petto C, Schneider M, Harzer W. Genetic response in masseter muscle after orthognathic surgery in comparison with healthy controls – A Microarray study. Journal of Cranio-Maxillofacial Surgery. 2017;45(4):547-51. | Title |
|  | Marini I, Alessandri Bonetti G, Bortolotti F, Bartolucci ML, Gatto MR, Michelotti A. Effects of experimental insoles on body posture, mandibular kinematics and masticatory muscles activity. A pilot study in healthy volunteers. Journal of Electromyography and Kinesiology. 2015;25(3):531-9. | Title |
|  | Marini I, Gatto MR, Bartolucci ML, Bortolotti F, Alessandri Bonetti G, Michelotti A. Effects of experimental occlusal interference on body posture: An optoelectronic stereophotogrammetric analysis. Journal of Oral Rehabilitation. 2013;40(7):509-18. | Title |
|  | Marito P, Hasegawa Y, Tamaki K, Maria MTS, Yoshimoto T, Kusunoki H, et al. The Association of Dietary Intake, Oral Health, and Blood Pressure in Older Adults: A Cross-Sectional Observational Study. Nutrients. 2022;14(6). | Title |
|  | Markov NM, Ivanov VV, Krechina EK, Pogabalo IV, Rumshiskaya AD, Rozhnova EV. [Relationship between masticatory muscles activity and motor cortex activation during treatment of patients with distal malocclusion]. Stomatologiia (Mosk). 2019;98(3):71-9. | Title |
|  | Markwardt J, Klemm E, Aust D, Lauer G. [Exsudativ proliferative synovialitis of the temporomandibular joint]. Laryngorhinootologie. 2007;86(10):728-31. | Title |
|  | Marotti J, Heger S, Tinschert J, Tortamano P, Chuembou F, Radermacher K, Wolfart S. Recent advances of ultrasound imaging in dentistry - a review of the literature. Oral Surgery Oral Medicine Oral Pathology Oral Radiology. 2013;115(6):819-32. | Title |
|  | Marques HB, Richter FF, Heck L, Xavier LL, de Campos D. Biomechanical potential of the temporal muscle in brachyfacial and dolichofacial subjects: a study on dry mandibles. Orthodontics & craniofacial research. 2016;19(3):162-8. | Title |
|  | Marquezin MCS, Andrade AdS, Rossi Md, Gameiro GH, Gavião MBD, Castelo PM. Avaliação do dimorfismo sexual e da relação entre as características craniofaciais, dos arcos dentários e do músculo masseter na fase de dentição mista. Revista CEFAC. 2014;16(4):1231-8. | Full text  Only Class II |
|  | Marquezin MCS, Gaviao MBD, Alonso M, Ramirez-Sotelo LR, Haiter-Neto F, Castelo PM. Relationship between orofacial function, dentofacial morphology, and bite force in young subjects. Oral Diseases. 2014;20(6):567-73. | Full text  Other outcomes |
|  | Marquezin MCS, Kobayashi FY, Montes ABM, Gavião MBD, Castelo PM. Assessment of masticatory performance, bite force, orthodontic treatment need and orofacial dysfunction in children and adolescents. Archives of Oral Biology. 2013;58(3):286-92. | Abstract |
|  | Marshall SD, Kruger K, Franciscus RG, Southard TE. Development of the mandibular curve of spee and maxillary compensating curve: A finite element model. Plos One. 2019;14(12). | Title |
|  | Martín C, Palma JC, Alamán JM, Lopez-Quiñones JM, Alarcón JA. Longitudinal evaluation of sEMG of masticatory muscles and kinematics of mandible changes in children treated for unilateral cross-bite. Journal of Electromyography and Kinesiology. 2012;22(4):620-8. | Title |
|  | Martin-Fernandez E, Gonzalez-Gonzalez I, Dellanos-Lanchares H, Mauvezin-Quevedo MA, Brizuela-Velasco A, Alvarez-Arenal A. Mandibular flexure and peri-implant bone stress distribution on an implant-supported fixed full-Arch mandibular prosthesis: 3D finite element analysis. BioMed Research International. 2018;2018. | Title |
|  | Martínez Aparicio C, Jaeaeskelaeinen SK, García Carricondo A, Espín Galvez F. Trismus and neuropathic pain caused by a lesion of the mandibular branch of the trigeminal nerve after local anesthesia. Clinical Neurophysiology. 2014;125:S214. | Title |
|  | Martini M, Wiedemeyer V, Heim N, Messing-Jünger M, Linsen S. Bite force and electromyography evaluation after cranioplasty in patients with craniosynostosis. Oral Surgery, Oral Medicine, Oral Pathology and Oral Radiology. 2017;124(6):e267-e75. | Title |
|  | Marushko T, Yakovenko L, Kiselova N, Holubovska Y, Kulchytska YE. Multidisciplinary approach to JIA diagnosis in pediatric patients. Pediatric Rheumatology. 2021;19(SUPPL 1). | Title |
|  | Mascarenhas S, Tuffin JR, Hassan I. Tuberculous submasseteric abscess: case report. British Journal of Oral and Maxillofacial Surgery. 2009;47(7):566-8. | Title |
|  | Masci C, Ciarrocchi I, Spadaro A, Necozione S, Marci MC, Monaco A. Does orthodontic treatment provide a real functional improvement? A case control study. BMC Oral Health. 2013;13(1). | Title |
|  | Mason AG, Scott BJJ, Van Der Glas HW, Linden RWA, Cadden SW. Remote noxious stimuli modulate jaw reflexes evoked by activation of periodontal ligament mechanoreceptors in man. Experimental Physiology. 2002;87(6):699-706. | Title |
|  | Massarelli O, Gobbi R, Raho MT, Tullio A. Three-dimensional primary reconstruction of anterior mouth floor and ventral tongue using the 'trilobed' buccinator myomucosal island flap. International Journal of Oral and Maxillofacial Surgery. 2008;37(10):917-22. | Title |
|  | Mastroianni D, Woods MG. 3D-CT assessment of mandibular widths in young subjects with different underlying vertical facial patterns. Journal of the World Federation of Orthodontists. 2019;8(2):78-86. | Included |
|  | Masunaga M, Tanaka H, Nakago T. High construction bite activator treatment of anterior crossbite in mixed dentition. Nihon Kyosei Shika Gakkai zasshi = The journal of Japan Orthodontic Society. 1989;48(1):66-78. | Title |
|  | Matarese G, Isola G, Alibrandi A, Lo Gullo A, Bagnato G, Cordasco G, Perillo L. Occlusal and MRI characterizations in systemic sclerosis patients: A prospective study from Southern Italian cohort. Joint Bone Spine. 2016;83(1):57-62. | Title |
|  | Matic DB, Yazdani A, Wells RG, Lee TY, Gan BS. The effects of masseter muscle paralysis on facial bone growth. Journal of Surgical Research. 2007;139(2):243-52. | Title |
|  | Matsumoto R, Ioi H, Goto TK, Hara A, Nakata S, Nakasima A, Counts AL. Relationship between the unilateral TMJ osteoarthritis/osteoarthrosis, mandibular asymmetry and the EMG activity of the masticatory muscles: a retrospective study. J Oral Rehabil. 2010;37(2):85-92. | Title |
|  | Matsumoto S, Morinushi T, Ogura T. Time dependent changes of variables associated with malocclusion in patients with Duchenne muscular dystrophy. The Journal of clinical pediatric dentistry. 2002;27(1):53-61. | Title |
|  | Matsuyuki T, Kitahara T, Nakashima A. Developmental changes in craniofacial morphology in subjects with Duchenne muscular dystrophy. European Journal of Orthodontics. 2006;28(1):42-50. | Title |
|  | Matt LB, McGee SW. Metastatic colon adenocarcinoma presenting as left preauricular pain. Journal of Gastrointestinal Cancer. 2012;43(SUPPL. 1):S251-S3. | Title |
|  | Mavreas D, Melsen B. Changes in the mechanical advantage of the masseter and temporal muscles following surgical correction of mandibular prognathism. Int J Adult Orthodon Orthognath Surg. 1997;12(3):215-25. | Title |
|  | Mavropoulos A, Ammann P, Bresin A, Kiliaridis S. Masticatory demands induce region-specific changes in mandibular bone density in growing rats. Angle Orthodontist. 2005;75(4):625-30. | Title |
|  | Mavropoulos A, Bresin A, Kiliaridis S. Morphometric analysis of the mandible in growing rats with different masticatory functional demands: adaptation to an upper posterior bite block. European Journal of Oral Sciences. 2004;112(3):259-66. | Title |
|  | Mavropoulos A, Kiliaridis S, Bresin A, Ammann P. Effect of different masticatory functional and mechanical demands on the structural adaptation of the mandibular alveolar bone in young growing rats. Bone. 2004;35(1):191-7. | Title |
|  | Mavropoulos A, Ödman A, Ammann P, Kiliaridis S. Rehabilitation of masticatory function improves the alveolar bone architecture of the mandible in adult rats. Bone. 2010;47(3):687-92. | Title |
|  | Mayne RJ, van der Poel C, Woods MG, Lynch GS. Skeletal effects of the alteration of masseter muscle function. Australian Orthodontic Journal. 2015;31(2):184-94. | Abstract |
|  | Mays S. Mandibular morphology in two archaeological human skeletal samples from northwest Europe with different masticatory regimes. Homo-Journal of Comparative Human Biology. 2015;66(3):203-15. | Title |
|  | Mays SA. Loss of molar occlusion and mandibular morphology in adults in an ancient human population consuming a coarse diet. American Journal of Physical Anthropology. 2013;152(3):383-92. | Title |
|  | Mazza D, Marini M, Impara L, Cassetta M, Scarpato P, Barchetti F, Di Paolo C. Anatomic examination of the upper head of the lateral pterygoid muscle using magnetic resonance imaging and clinical data. Journal of Craniofacial Surgery. 2009;20(5):1508-11. | Title |
|  | Mazzone N, Matteini C, Incisivo V, Belli E. Temporomandibular joint disorders and maxillomandibular malformations: role of condylar "repositionin" plate. J Craniofac Surg. 2009;20(3):909-15. | Title |
|  | McCollum MA. Palatal thickening and facial form in Paranthropus: Examination of alternative developmental models. American Journal of Physical Anthropology. 1997;103(3):375-92. | Title |
|  | McGoldrick DM, Alsabbagh AY, Shaikh M, Pettit L, Bhatia SK. Masseter muscle defined sarcopenia and survival in head and neck cancer patients. British Journal of Oral & Maxillofacial Surgery. 2022;60(4):454-8. | Title |
|  | McNamara DC. Occlusal adjustment for a physiologically balanced occlusion. The Journal of Prosthetic Dentistry. 1977;38(3):284-93. | Title |
|  | McNamara JA, Lione R, Franchi L, Angelieri F, Cevidanes LHS, Darendeliler MA, Cozza P. The role of rapid maxillary expansion in the promotion of oral and general health. Progress in Orthodontics. 2015;16(1):1-7. | Title |
|  | McNamee A, Robertson T, Sounness B, O'Gorman P. FDG PET/CT of metabolic myopathy with posttreatment follow-up. Clinical Nuclear Medicine. 2018;43(9):e316-e8. | Title |
|  | Mehanna P, Bertram A, Wilson I. Calcified facial haematoma. New Zealand Medical Journal. 2009;122(1306):84-5. | Title |
|  | Melis M. Dr. Melis Comments on Chakfa, et al.'s article in the October 2002 issue of CRANIO. Cranio - Journal of Craniomandibular and Sleep Practice. 2003;21(2):86. | Title |
|  | Melke GSD, Costa ALF, Lopes S, Fuziy A, Ferreira-Santos RI. Three-dimensional lateral pterygoid muscle volume: MRI analyses with insertion patterns correlation. Annals of Anatomy-Anatomischer Anzeiger. 2016;208:9-18. | Abstract |
|  | Melugin MB, Oyen OJ, Indresano AT. The effect of rim mandibulectomy configuration and residual segment size on postoperative fracture risk: An in vitro study. Journal of Oral and Maxillofacial Surgery. 2001;59(4):409-13. | Title |
|  | Mendes RA, Upton LG. Management of dystonia of the lateral pterygoid muscle with botulinum toxin A. British Journal of Oral and Maxillofacial Surgery. 2009;47(6):481-3. | Title |
|  | Menegaz RA, Baier DB, Metzger KA, Herring SW, Brainerd EL. XROMM analysis of tooth occlusion and temporomandibular joint kinematics during feeding in juvenile miniature pigs. Journal of Experimental Biology. 2015;218(16):2573-84. | Title |
|  | Menéndez L, Bernal V, Novellino P, Perez SI. Effect of bite force and diet composition on craniofacial diversification of Southern South American human populations. American Journal of Physical Anthropology. 2014;155(1):114-27. | Title |
|  | Merema BBJ, Sieswerda JJ, Spijkervet FKL, Kraeima J, Witjes MJH. A Contemporary Approach to Non-Invasive 3D Determination of Individual Masticatory Muscle Forces: A Proof of Concept. Journal of Personalized Medicine. 2022;12(8). | Title |
|  | Merigue LF, Conti ACDCF, Oltramari-Navarro PVP, Navarro RDL, de Almeida MR. Tomographic evaluation of the temporomandibular joint in malocclusion subjects: condylar morphology and position. Brazilian Oral Research. 2016;30(1). | Title |
|  | Mescollotto FF, Pelai EB, de Castro EM, Pires PF, Ferreira TS, Bigaton DR. Influence of smartphone use on the median frequency of mastigatory and trapezius muscles in women - a Pilot study. Journal of Bodywork and Movement Therapies. 2020;24(2):69-73. | Title |
|  | Mesnard M, Ramos A, Ballu A, Morlier J, Cid M, Simoes JA. Biomechanical analysis comparing natural and alloplastic temporomandibular joint replacement using a finite element model. Journal of Oral and Maxillofacial Surgery. 2011;69(4):1008-17. | Title |
|  | Messere A, Tschakovsky M, Seddone S, Lulli G, Franco W, Maffiodo D, et al. Hyper-Oxygenation Attenuates the Rapid Vasodilatory Response to Muscle Contraction and Compression. Frontiers in Physiology. 2018;9. | Title |
|  | Messina G, Amato A, Rizzo F, Dominguez LJ, Iovane A, Barbagallo M, Proia P. The Association between Masticatory Muscles Activation and Foot Pressure Distribution in Older Female Adults: A Cross-Sectional Study. International Journal of Environmental Research and Public Health. 2023;20(6). | Title |
|  | Meter Grabovac M, Dediol E, Biočić J, Lešin A, Macan D, Perić B. HYPERPLASIA OF THE MANDIBULAR CORONOID PROCESSES - A CASE REPORT. Acta Stomatologica Croatica. 2022;56(4):433-4. | Title |
|  | Meyer C, Kahn JL, Lambert A, Boutemy P, Wilk A. Development of a static simulator of the mandible. Journal of Cranio-Maxillofacial Surgery. 2000;28(5):278-86. | Title |
|  | Meziane M, Boulaadas M, Eabdenbitsen A, Dib N, Essakalli L, Kzadri M. Intramasseteric hemangioma operated by intraoral approach. Revue de Stomatologie et de Chirurgie Maxillo-Faciale. 2010;111(3):168-71. | Title |
|  | Michelotti A. An interview with Ambrosina Michelotti. Dental Press Journal of Orthodontics. 2018;23(2):22-9. | Title |
|  | Michelotti A, Iodice G. The role of orthodontics in temporomandibular disorders. Journal of Oral Rehabilitation. 2010;37(6):411-29. | Title |
|  | Michelotti A, Rongo R, Valentino R, D’Antò V, Bucci R, Danzi G, Cioffi I. Evaluation of masticatory muscle activity in patients with unilateral posterior crossbite before and after rapid maxillary expansion. European Journal of Orthodontics. 2019;41(1):46-53. | Title |
|  | Mijiritsky E, Shacham M, Meilik Y, Dekel-Steinkeller M. Clinical Influence of Mandibular Flexure on Oral Rehabilitation: Narrative Review. International Journal of Environmental Research and Public Health. 2022;19(24). | Title |
|  | Milhomem AC, de Souza Jorge IM, da Costa EL, Vinaud MC, de Souza Lino R. Polymethyl methacrylate (PMMA) in the treatment of a case of hemifacial microsomia. Aesthetic Surgery Journal Open Forum. 2020;2(1). | Title |
|  | Mills JRE. Die Behandlung der Klasse II/1 in England - 1. Grundsätzliche Betrachtungen. Fortschritte der Kieferorthopädie. 1969;30(1):72-81. | Title |
|  | Min HJ, Kim KS. Primary Non-Hodgkin's lymphoma originating from the masseter muscle. Bangladesh Journal of Medical Science. 2021;20(1):181-6. | Title |
|  | Min L, Lai G, Xin L. Changes in Masseter Muscle Following Curved Ostectomy of the Prominent Mandibular Angle: An Initial Study With Real-Time 3D Ultrasonograpy. Journal of Oral and Maxillofacial Surgery. 2008;66(12):2434-43. | Title |
|  | Minami I, Akhter R, Albersen I, Burger C, Whittle T, Lobbezoo F, et al. Masseter motor unit recruitment is altered in experimental jaw muscle pain. Journal of Dental Research. 2013;92(2):143-8. | Title |
|  | Miralles R, Berger B, Bull R, Manns A, Carvajal R. Influence of the activator on electromyographic activity of mandibular elevator muscles. American Journal of Orthodontics and Dentofacial Orthopedics. 1988;94(2):97-103. | Title |
|  | Miralles R, Hevia R, Contreras L, Carvajal R, Bull R, Manns A. Patterns of electromyographic activity in subjects with different skeletal facial types. Angle Orthodontist. 1991;61(4):277-84. | Full text  Other outcomes |
|  | Miranda-Viana M, Moreira GM, de Souza LM, Nejaim Y, Haiter-Neto F, Freitas DQ. Tridimensional assessment of the mandibular angle in patients with different skeletal patterns by cone-beam computed tomography scans: a retrospective study. Bmc Oral Health. 2023;23(1). | Title |
|  | Mirzoyev MS, Gapharov KО, Khushvakhtov DI, Shakirov MN, Jonibekova RN, Yulchiev RI. Functional state of mastication muscles in patients during treatment of lower jaw defects with implants. International Journal of Pharmaceutical Research. 2020;12(1):2510-7. | Title |
|  | Mitsuyama A, Takahashi T, Ueno T. Effects of teeth clenching on the soleus H reflex during lower limb muscle fatigue. Journal of Prosthodontic Research. 2017;61(2):202-9. | Title |
|  | Mittal A, Das D, Iyer N, Nagaraj J, Gupta M. Masseter cysticercosis - A rare case diagnosed on ultrasound. Dentomaxillofacial Radiology. 2008;37(2):113-6. | Title |
|  | Miyajima K, Imamura S, Fuwa Y, Nakamura S, Nagahara K, Tsuchiya T, et al. Posterior bite raising effects on a primary anterior crossbite case. The Journal of clinical pediatric dentistry. 1995;19(2):131-4. | Title |
|  | Miyamoto K, Ishizuka Y, Tanne K. Changes in masseter muscle activity during orthodontic treatment evaluated by a 24-hour EMG system. Angle Orthodontist. 1996;66(3):223-8. | Title |
|  | Miyamoto K, Ishizuka Y, Ueda HM, Saifuddin M, Shikata N, Tanne K. Masseter muscle activity during the whole day in children and young adults. Journal of Oral Rehabilitation. 1999;26(11):858-64. | Title |
|  | Miyamoto K, Yamada K, Ishizuka Y, Morimoto N, Tanne K. Masseter muscle activity during the whole day in young adults. American journal of orthodontics and dentofacial orthopedics : official publication of the American Association of Orthodontists, its constituent societies, and the American Board of Orthodontics. 1996;110(4):394-8. | Title |
|  | Miyazaki M, Yonemitsu I, Takei M, Kure-Hattori I, Ono T. The imbalance of masticatory muscle activity affects the asymmetric growth of condylar cartilage and subchondral bone in rats. Archives of Oral Biology. 2016;63:22-31. | Title |
|  | Mizen KD, Loukota RA, Addante RR. Mass in the Masseter Muscle. Journal of Oral and Maxillofacial Surgery. 2004;62(5):607-10. | Title |
|  | Mizuno S, Matsunaga S, Kasahara N, Kasahara M, Shimoo Y, Abe S, et al. Effect of the Correction of Bilateral Differences in Masseter Muscle Functional Pressure on the Mandible of Growing Rats. Journal of Functional Biomaterials. 2023;14(8). | Title |
|  | Mizutani S, Matsuzaki H, Iyota K, Tani A, Oku S, Tabuchi H, et al. Changes of Oral and Physical Functions in Older Japanese Who Participated in Keyboard Harmonica and Exercise Classes during COVID-19-Related Movement Restrictions. International Journal of Environmental Research and Public Health. 2023;20(4). | Title |
|  | Moawad HA, Sinanan AC, Lewis MP, Hunt NP. Grouping patients for masseter muscle genotype-phenotype studies. Angle Orthod. 2012;82(2):261-6. | Abstract |
|  | Moayedi M, Krishnamoorthy G, He PY, Agur A, Weissman-Fogel I, Tenenbaum HC, et al. Structural abnormalities in the temporalis musculo-aponeurotic complex in chronic muscular temporomandibular disorders. Pain. 2020;161(8):1787-97. | Title |
|  | Moazen M, Curtis N, Evans SE, O'Higgins P, Fagan MJ. Combined finite element and multibody dynamics analysis of biting in a <i>Uromastyx hardwickii</i> lizard skull. Journal of Anatomy. 2008;213(5):499-508. | Title |
|  | Mohl ND. Head posture and its role in occlusion. New York State Dental Journal. 1976;42(1):17-23. | Title |
|  | Mohlin BO, Derweduwen K, Pilley R, Kingdon A, Shaw WC, Kenealy P. Malocclusion and temporomandibular disorder: A comparison of adolescents with moderate to severe dysfunction with those without signs and symptoms of temporomandibular disorder and their further development to 30 years of age. Angle Orthodontist. 2004;74(3):319-27. | Title |
|  | Mokhtar EA, Rattan V, Rai S, Jolly SS, Lal V. Analysis of maximum bite force and chewing efficiency in unilateral temporomandibular joint ankylosis cases treated with buccal fat pad interpositional arthroplasty. British Journal of Oral and Maxillofacial Surgery. 2022;60(3):313-9. | Title |
|  | Møller E, Bakke M. Morphological and functional malocclusion. L" Orthodontie francaise. 1986;57 Pt 2:477-500. | Title |
|  | MÖLler E, Sheikholeslam A, Lous I. Response of elevator activity during mastication to treatment of functional disorders. European Journal of Oral Sciences. 1984;92(1):64-83. | Title |
|  | Moltoni G, D'Arco F, Rossi-Espadnet MC, James G, Hayward R. Observations on the growth of temporalis muscle: A 3D CT imaging study. Journal of Anatomy. 2021;238(5):1218-24. | Title |
|  | Mommaerts MY. On the reinsertion of the lateral pterygoid tendon in total temporomandibular joint replacement surgery. Journal of Cranio-Maxillofacial Surgery. 2019;47(12):1913-7. | Title |
|  | Monaco A, Spadaro A, Cattaneo R, Giannoni M. Effects of myogenous facial pain on muscle activity of head and neck. International Journal of Oral and Maxillofacial Surgery. 2010;39(8):767-73. | Title |
|  | Monemi M, Eriksson PO, Eriksson A, Thornell LE. Adverse changes in fibre type composition of the human masseter versus biceps brachii muscle during aging. Journal of the Neurological Sciences. 1998;154(1):35-48. | Title |
|  | Montag NS. Temporomandibular joint heterotopic ossification and myositis ossificans of facial musculature following traumatic brain injury: A rare case. PM and R. 2018;10(9):S60-S1. | Title |
|  | Monteiro AA, Kopp S. Reproducibility of estimation of blood flow in the human masseter muscle from measurements of 133xe clearance. Acta Odontologica Scandinavica. 1989;47(5):329-36. | Title |
|  | Moon HJ, Lee YK. The relationship between dental occlusion/temporomandibular joint status and general body health: Part 1. Dental occlusion and TMJ status exert an influence on general body health. Journal of Alternative and Complementary Medicine. 2011;17(11):995-1000. | Title |
|  | Moreira CVA, Serra AVP, Silva LOR, Fernandes ACF, de Azevedo RA. Total bilateral TMJ reconstruction for pain and dysfunction: Case report. International Journal of Surgery Case Reports. 2018;42:138-44. | Title |
|  | Moreno Hay I, Sanchez T, Ardizone I, Aneiros F, Celemin A. Electromyographic comparisons between clenching, swallowing and chewing in jaw muscles with varying occlusal parameters. Medicina Oral, Patologia Oral y Cirugia Bucal. 2008;13(3):207-13. | Title |
|  | Moreno-Hay I, Okeson JP. Does altering the occlusal vertical dimension produce temporomandibular disorders? A literature review. Journal of Oral Rehabilitation. 2015;42(11):875-82. | Title |
|  | Morimitsu T, Nokubi T, Nagashima T, Yoshida M, Ikebe K, Okuno Y. Relationship between orofaciocranial morphologic factors and electromyographic activities of the masticatory muscles. Nihon Ago Kansetsu Gakkai Zasshi. 1989;1(1):162-71. | Full text  Other outcomes |
|  | Morita K, Tsuka H, Kimura H, Mori T, Yoshikawa M, Yoshida M, et al. Oral function and vertical jump height among healthy older people in Japan. Community Dent Health. 2019;36(4):275-9. | Title |
|  | Moriya Y, Tuchida K, Moriya Y, Sawada T, Koga J, Sato J, et al. The influence of craniofacial form on bite force and EMG activity of masticatory muscles. VIII-1. Bite force of complete denture wearers. J Oral Sci. 1999;41(1):19-27. | Title |
|  | Mork AL. Evolutionary Morphology of the Masticatory Apparatus in Tree Gouging Marmosets [Dissertation/Thesis]2012. | Title |
|  | Mormina E, Granata F, Gaeta M, Longo M, Calamuneri A, Arrigo A, et al. Microstructural investigation of masticatory muscles: a pre- and post-treatment diffusion tensor imaging study in a bruxism case. Dentomaxillofacial Radiology. 2018;47(5). | Title |
|  | Morneburg TR, Döhla S, Wichmann M, Pröschel PA. Afferent sensory mechanisms involved in jaw gape-related muscle activation in unilateral biting. Clinical Oral Investigations. 2014;18(3):883-90. | Title |
|  | Moroi A, Ishihara Y, Sotobori M, Iguchi R, Kosaka A, Ikawa H, et al. Changes in occlusal function after orthognathic surgery in mandibular prognathism with and without asymmetry. International Journal of Oral and Maxillofacial Surgery. 2015;44(8):971-6. | Title |
|  | Morris PJR, Cox PG, Cobb SNF. The biomechanical significance of the elongated rodent incisor root in the mandible during incision. Scientific Reports. 2022;12(1). | Title |
|  | Moss JP. An investigation of the muscle activity of patients with Class II Division 2 malocclusion and the changes during treatment. Transactions European Orthodontic Society. 1975:87-101. | Title |
|  | Moss JP. A cephalometric and electromyographic investigation of patients treated for the correction of mandibular prognathism by mandibular surgery only. International journal of orthodontics. 1985;23(1-2):10-9. | Title |
|  | Moss ML, Simon MR. Growth of the human mandibular angular process: A functional cranial analysis. American Journal of Physical Anthropology. 1968;28(2):127-38. | Title |
|  | Motoyoshi M, Shimazaki T, Hosoi K, Wada M, Namura S. Stresses on the cervical column associated with vertical occlusal alteration. European Journal of Orthodontics. 2003;25(2):135-8. | Title |
|  | Motoyoshi M, Shimazaki T, Sugai T, Namura S. Biomechanical influences of head posture on occlusion: An experimental study using finite element analysis. European Journal of Orthodontics. 2002;24(4):319-26. | Title |
|  | Mücke T, Löffel A, Kanatas A, Karnezi S, Rana M, Fichter A, et al. Botulinum toxin as a therapeutic agent to prevent relapse in deep bite patients. Journal of Cranio-Maxillofacial Surgery. 2016;44(5):584-9. | Title |
|  | Muftuoglu O, Akturk ES, Eren H, Gorurgoz C, Karasu HA, Orhan K, et al. Long-term evaluation of masseter muscle activity, dimensions, and elasticity after orthognathic surgery in skeletal class III patients. Clin Oral Investig. 2023;27(7):3855-61. | Title |
|  | Muller K, Chen J, Hoi U, Hansen L. Trigeminal nerve neuromuscular choristoma versus rhabdomyoma. Journal of Neuropathology and Experimental Neurology. 2013;72(6):591. | Title |
|  | Mulliken JB, Le MN. A craniofacial glossary. Journal of Craniofacial Surgery. 2008;19(3):705-12. | Title |
|  | Mundt T, Mack F, Schwahn C, Bernhardt O, Kocher T, John U, Biffar R. Gender differences in associations between occlusal support and signs of temporomandibular disorders: results of the population-based Study of Health in Pomerania (SHIP). International Journal of Prosthodontics. 2005;18(3):232-9. | Title |
|  | Munhoz WC, Hsing WT. Interrelations between orthostatic postural deviations and subjects' age, sex, malocclusion, and specific signs and symptoms of functional pathologies of the temporomandibular system: A preliminary correlation and regression study. Cranio - Journal of Craniomandibular and Sleep Practice. 2014;32(3):175-86. | Title |
|  | Murakami K, Yamamoto K, Sugiura T, Horita S, Matsusue Y, Kirita T. Computed Tomography–Based 3-Dimensional Finite Element Analyses of Various Types of Plates Placed for a Virtually Reduced Unilateral Condylar Fracture of the Mandible of a Patient. Journal of Oral and Maxillofacial Surgery. 2017;75(6):1239.e1-.e11. | Title |
|  | Murakami K, Yamamoto K, Sugiura T, Yamanaka Y, Kirita T. Changes in Mandibular Movement and Occlusal Condition After Conservative Treatment for Condylar Fractures. Journal of Oral and Maxillofacial Surgery. 2009;67(1):83-91. | Title |
|  | Murakami K, Yamamoto K, Tsuyuki M, Sugiura T, Tsutsumi S, Kirita T. Theoretical efficacy of preventive measures for pathologic fracture after surgical removal of mandibular lesions based on a three-dimensional finite element analysis. Journal of Oral and Maxillofacial Surgery. 2014;72(4):833.e1-.e18. | Title |
|  | Murakami M, Hirano H, Watanabe Y, Sakai K, Kim H, Katakura A. Relationship between chewing ability and sarcopenia in Japanese community-dwelling older adults. Geriatrics and Gerontology International. 2015;15(8):1007-12. | Title |
|  | Murakami M, Iijima K, Watanabe Y, Tanaka T, Iwasa Y, Edahiro A, et al. Development of a simple method to measure masseter muscle mass. Gerodontology. 2020;37(4):383-8. | Title |
|  | Muralee Mohan C, Thakral A, Bhat SK. Primary extranodal non-Hodgkin's lymphoma involving masseter and buccinator muscles. International Journal of Oral and Maxillofacial Surgery. 2012;41(11):1393-6. | Title |
|  | Muraoka H, Kaneda T, Ito K, Hirahara N, Kondo T, Tokunaga S. Quantitative analysis of masticatory muscle changes by Eichner index using diffusion-weighted imaging. Oral Radiology. 2023;39(2):437-45. | Title |
|  | Murray GM, Peck CC. Orofacial pain and jaw muscle activity: A new model. Journal of Orofacial Pain. 2007;21(4):263-78. | Title |
|  | Musto F, Rosati R, Sforza C, Toma M, Dellavia C. Standardised surface electromyography allows effective submental muscles assessment. Journal of Electromyography and Kinesiology. 2017;34:1-5. | Title |
|  | Nagahara K, Miyajima K, Tanida K, Yamada A, Nakamura S, Iizuka T. Some considerations about maintenance of the mandibular position for adult patient with TMJ disorder. Aichi Gakuin Dent Sci. 1990;3:17-25. | Title |
|  | Nagar Y, Arensburg B. Bilateral aplasia of the condyles in a 1,400-year-old mandible from Israel. American Journal of Physical Anthropology. 2000;111(1):135-9. | Title |
|  | Nagayama K, Suenaga S, Nagata J, Takada H, Majima HJ, Miyawaki S. Clinical significance of magnetization transfer contrast imaging for edematous changes in masticatory muscle. Journal of Computer Assisted Tomography. 2010;34(2):233-41. | Title |
|  | Nagayama K, Tomonari H, Kitashima F, Miyawaki S. Extraction treatment of a Class II division 2 malocclusion with mandibular posterior discrepancy and changes in stomatognathic function. Angle Orthodontist. 2015;85(2):314-21. | Title |
|  | Nagpal R, Georgi G, Knauth S, Schmid-Herrmann C, Muschol N, Braulke T, et al. Early enzyme replacement therapy prevents dental and craniofacial abnormalities in a mouse model of mucopolysaccharidosis type VI. Frontiers in Physiology. 2022;13. | Title |
|  | Nagy AD, Simhofer H. Mandibular condylectomy and meniscectomy for the treatment of septic temporomandibular joint arthritis in a horse. Veterinary Surgery. 2006;35(7):663-8. | Title |
|  | Naik K, Shetty P, Hegde P. Adenoid cystic carcinoma of buccal mucosa with extensive hyalinization: A unique case report. Annals of Tropical Medicine and Public Health. 2013;6(5):571-4. | Title |
|  | Nair R, Deguchi TS, Li X, Katashiba S, Chan YH. Quantitative analysis of the maxilla and the mandible in hyper- and hypodivergent skeletal class II pattern. Orthodontics & Craniofacial Research. 2009;12(1):9-13. | Title |
|  | Nakajima K, Yamaguchi T, Maki K. Surgical orthodontic treatment for a patient with advanced periodontal disease: evaluation with electromyography and 3-dimensional cone-beam computed tomography. Am J Orthod Dentofacial Orthop. 2009;136(3):450-9. | Title |
|  | Nakajima S, Osato S. Association of gonial angle with morphology and bone mineral content of the body of the adult human mandible with complete permanent dentition. Annals of Anatomy-Anatomischer Anzeiger. 2013;195(6):533-8. | Title |
|  | Nakajima T, Kajikawa Y, Tokiwa N, Hanada K. Stability of the mandible after surgical correction of skeletal Class III malocclusion in 50 patients. Journal of Oral Surgery. 1979;37(1):21-5. | Title |
|  | Nakakawaji K, Kodachi K, Sakamoto T, Harazaki M, Isshiki Y. Correlation between facial patterns and function of the masticatory muscles in girls and women. The Bulletin of Tokyo Dental College. 2002;43(2):51-9. | Full text  Other outcomes |
|  | Nakamura A, Zeredo JL, Utsumi D, Fujishita A, Koga Y, Yoshida N. Influence of malocclusion on the development of masticatory function and mandibular growth. Angle Orthodontist. 2013;83(5):749-57. | Abstract |
|  | Nakamura M, Imaoka M, Tazaki F, Nakao H, Hida M, Kono R, et al. Association between Bone-Related Physiological Substances and Oral Function in Community-Dwelling Older People. International Journal of Environmental Research and Public Health. 2022;19(17). | Title |
|  | Nakamura N, Fujihara H, Kawaguchi K, Yamada H, Nakayama R, Yasukawa M, et al. Possible Action of Olaparib for Preventing Invasion of Oral Squamous Cell Carcinoma In Vitro and In Vivo. International Journal of Molecular Sciences. 2022;23(5). | Title |
|  | Nakamura S, Okochi K, Murata Y, Shibuya H, Kurabayashi T. [18F]Fluorodeoxyglucose-PET/CT differentiation between physiological and pathological accumulations in head and neck. Nuclear Medicine Communications. 2009;30(7):498-503. | Title |
|  | Nakamura T, Yoshida Y, Churei H, Aizawa J, Hirohata K, Ohmi T, et al. The effect of teeth clenching on dynamic balance at jump-landing: A pilot study. Journal of Applied Biomechanics. 2017;33(3):211-5. | Title |
|  | Nakamura YN, Iwamoto H, Etoh T, Shiotsuka Y, Yamaguchi T, Ono Y, et al. Three-dimensional observation of connective tissue of bovine <i>Masseter</i> muscle under concentrate -: and roughage-fed conditions by using immunohistochemical/confocal laser-scanning microscopic methods. Journal of Food Science. 2007;72(6):E375-E81. | Title |
|  | Nakano H, Maki K, Shibasaki Y, Miller AJ. Three-dimensional changes in the condyle during development of an asymmetrical mandible in a rat: A microcomputed tomography study. American Journal of Orthodontics and Dentofacial Orthopedics. 2004;126(4):410-20. | Title |
|  | Nakashima A, Yamada T, Sugiyama G, Mizunoya W, Nakano H, Yasuda K, et al. Masseter Muscle Properties Differ between the Left and Right Sides in Mandibular Class III Patients with Asymmetry. Journal of Hard Tissue Biology. 2020;29(1):25-30. | Abstract |
|  | Nakata S, Mizuno M, Koyano K, Nakayama E, Watanabe M, Murakami T. Functional masticatory evaluation in hemifacial microsomia. Eur J Orthod. 1995;17(4):273-80. | Title |
|  | Nakata Y, Ueda HM, Kato M, Tabe H, Shikata-Wakisaka N, Matsumoto E, et al. Changes in stomatognathic function induced by orthognathic surgery in patients with mandibular prognathism. J Oral Maxillofac Surg. 2007;65(3):444-51. | Title |
|  | Nakayama M, Ariji Y, Nishiyama W, Ariji E. Evaluation of the masseter muscle elasticity with the use of acoustic coupling agents as references in strain sonoelastography. Dentomaxillofacial Radiology. 2015;44(3). | Title |
|  | Nalamliang N, Thongudomporn U. Effects of class II intermaxillary elastics on masticatory muscle activity balance, occlusal contact area and masticatory performance: A multicenter randomised controlled trial. Journal of Oral Rehabilitation. 2023;50(2):131-9. | Title |
|  | Narayanan CD, Prakash P, Dhanasekaran CK. Intramuscular hemangioma of the masseter muscle: A case report. Cases Journal. 2009;2(5). | Title |
|  | Nardi C, Vignoli C, Pietragalla M, Tonelli P, Calistri L, Franchi L, et al. Imaging of mandibular fractures: a pictorial review. Insights into Imaging. 2020;11(1). | Title |
|  | Narsinh P, Lin YK. Idiopathic myositis ossificans of the lateral pterygoid muscle in the paediatric patient: A case report. Advances in Oral and Maxillofacial Surgery. 2021;1. | Title |
|  | Naser-Ud-Din S, Sampson WJ, Dreyer CW, Thoirs K. Ultrasound measurements of the masseter muscle as predictors of cephalometric indices in orthodontics: A pilot study. Ultrasound in Medicine and Biology. 2010;36(9):1412-21. | Included |
|  | Naser-Ud-Din S, Sowman PF, Sampson WJ, Dreyer CW, Türker KS. Masseter length determines muscle spindle reflex excitability during jaw-closing movements. American Journal of Orthodontics and Dentofacial Orthopedics. 2011;139(4):e305-e13. | Full text  Other outcomes |
|  | Naser-Ud-Din S, Thoirs K, Sampson WJ. Ultrasonography, lateral cephalometry and 3D imaging of the human masseter muscle. Orthodontics & Craniofacial Research. 2011;14(1):33-43. | Included |
|  | Nash PG, MacEfield VG, Klineberg IJ, Gustin SM, Murray GM, Henderson LA. Bilateral activation of the trigeminothalamic tract by acute orofacial cutaneous and muscle pain in humans. Pain. 2010;151(2):384-93. | Title |
|  | Nashida Y, Yamakado K, Kumamoto T, Suga S, Takaki H, Hori H, et al. Radiofrequency ablation used for the treatment of frequently recurrent rhabdomyosarcoma in the masticator space in a 10-year-old girl. Journal of Pediatric Hematology/Oncology. 2007;29(9):640-2. | Title |
|  | Nasr WF, Elsheikh E, El-Anwar MW, Sweed AH, Bessar A, Ezzeldin N. Two- versus Three-Point Internal Fixation of Displaced Zygomaticomaxillary Complex Fractures. Craniomaxillofacial trauma and reconstruction. 2018;11(4):256‐64. | Title |
|  | Navarrete AL, Rafferty KL, Liu ZJ, Ye WM, Greenlee GM, Herring SW. Botulinum neurotoxin type A in the masseter muscle: Effects on incisor eruption in rabbits. American Journal of Orthodontics and Dentofacial Orthopedics. 2013;143(4):499-506. | Title |
|  | Navic P, Inthasan C, Chaimongkhol T, Mahakkanukrauh P. Facial reconstruction using 3-D computerized method: A scoping review of Methods, current Status, and future developments. Legal Medicine. 2023;62. | Title |
|  | Nayak SS, Arun S, Taranath Kamath A, Jaladhigere Lakshmanagowda B, Dubey E, Koshy J. The Influence of the Mandibular Chin Angle on the Occurrence of Mandibular Condylar Fracture: A Retrospective Study. Scientific World Journal. 2021;2021. | Title |
|  | Nct. Evaluation of Muscle Activity, Bite Force and Salivary Cortisol in Children With Bruxism. https://clinicaltrialsgov/show/NCT02757261. 2016. | Title |
|  | Nct. Satisfaction and Adaptation After Elevation of Vertical Dimension of Occlusion Using Overlay Denture or Fixed Crowns. https://clinicaltrialsgov/show/NCT03579134. 2018. | Title |
|  | Nct. Evaluation of Patient Satisfaction Using Autogenous Inlay (Sandwich) Eminoplasty Versus Patient Specific Poly Ether-ether Ketone (PEEK) Onlay Implant. https://clinicaltrialsgov/show/NCT03994692. 2019. | Title |
|  | Nct. Effect of Buccal Fat Pad and Platelet Rich Fibrin After Ultrasonic Hydrodynamic Maxillary Sinus Membrane Elevation. https://clinicaltrialsgov/show/NCT04123860. 2019. | Title |
|  | Neaux D, Blanc B, Ortiz K, Locatelli Y, Laurens F, Baly I, et al. How Changes in Functional Demands Associated with Captivity Affect the Skull Shape of a Wild Boar (<i>Sus scrofa</i>). Evolutionary Biology. 2021;48(1):27-40. | Title |
|  | Neff CW, Kydd WL. The open bite: physiology and occlusion. Angle Orthodontist. 1966;36(4):351-7. | Title |
|  | Negishi S, Sato K, Kasai K. The Effects of Chewing Exercises on Masticatory Function after Surgical Orthodontic Treatment. Applied Sciences-Basel. 2021;11(18). | Title |
|  | Nemeth P. Adaptation to chronic lengthening of the rat masseter and temporalis muscles [Dissertation/Thesis]1988. | Title |
|  | Nesvick CL, Perry A, Graffeo CS, Nguyen QBD, Raghunathan A, Jentoft ME, et al. Primary intracranial gliosarcoma with extensive invasion of the skull base, brain parenchyma, orbit, and muscles of mastication. Journal of Neurological Surgery Part B: Skull Base. 2017;78. | Title |
|  | Newton JP, McClure IJ, Cowpe JG, Delday MI, Maltin CA. Masseteric hypertrophy?: Preliminary report. British Journal of Oral and Maxillofacial Surgery. 1999;37(5):405-8. | Title |
|  | Ng HP, Liu J, Huang S, Ong SH, Foong KWC, Goh PS, Nowinski WL. An improved shape determinative slice determination method for patient-specific modeling of facial anatomical structure. International Journal of Computer Assisted Radiology and Surgery. 2008;3(3-4):221-30. | Title |
|  | Ngan PW, Hagg U, Yiu C, Wei SH. Treatment response and long-term dentofacial adaptations to maxillary expansion and protraction. Seminars in orthodontics. 1997;3(4):255‐64. | Title |
|  | Ngan PW, Yiu C, Hagg U, Wei SHY, Bowley J. Masticatory muscle pain before, during, and after treatment with orthopedic protraction headgear: A pilot study. Angle Orthodontist. 1997;67(6):433-8. | Title |
|  | Ngom, P.I., et al., Masseter muscle thickness in relation to craniofacial morphology. International Orthodontics, 2008. 6(3): p. 251-267. | Included |
|  | Nguyen A, Balaban JP, Azizi E, Talmadge RJ, Lappin AK. Fatigue resistant jaw muscles facilitate long-lasting courtship behaviour in the southern alligator lizard (<i>Elgaria multicarinata</i>). Proceedings of the Royal Society B-Biological Sciences. 2020;287(1935). | Title |
|  | Nguyen MS, Saag M, Jagomägi T, Nguyen QH, Voog-Oras Ü. The impact of occlusal support on temporomandibular disorders: a literature review. Proceedings of Singapore Healthcare. 2022;31. | Title |
|  | Nguyen TK. A Systematic Review of Cephalometric Normative Data in Children [Dissertation/Thesis]2022. | Title |
|  | Nguyen TQ, Lewis JH. Sumatriptan-associated ischemic colitis: Case report and review of the literature and FAERS. Drug Safety. 2014;37(2):109-21. | Title |
|  | Nicholson EK, Stock SR, Hamrick MW, Ravosa MJ. Biomineralization and adaptive plasticity of the temporomandibular joint in myostatin knockout mice. Archives of Oral Biology. 2006;51(1):37-49. | Title |
|  | Nickel JC, Weber AL, Covington Riddle P, Liu Y, Liu H, Iwasaki LR. Mechanobehaviour in dolichofacial and brachyfacial adolescents. Orthod Craniofac Res. 2017;20 Suppl 1:139-44. | Abstract |
|  | Nickel JC, Yao P, Spalding PM, Iwasaki LR. Validated numerical modeling of the effects of combined orthodontic and orthognathic surgical treatment on TMJ loads and muscle forces. American Journal of Orthodontics and Dentofacial Orthopedics. 2002;121(1):73-83. | Title |
|  | Nicolai P, Villaret AB, Farina D, Nadeau S, Yakirevitch A, Berlucchi M, Galtelli C. Endoscopic surgery for juvenile angiofibroma: A critical review of indications after 46 cases. American Journal of Rhinology and Allergy. 2010;24(2):e67-e72. | Title |
|  | Nicot R, Raoul G, Vieira AR, Ferri J, Sciote JJ. ACTN3 genotype influences masseter muscle characteristics and self-reported bruxism. Oral Diseases. 2023;29(1):232-44. | Title |
|  | Nielsen HJ, Bakke M, Blixencrone-Møller T. Functional and orthodontic treatment of a patient with an open bite craniomandibular disorder. Tandlaegebladet. 1991;95(18):877-81. | Title |
|  | Nielsen PK, Andersen LL, Olsen HB, Rosendal L, Sjogaard G, Sogaard K. EFFECT OF PHYSICAL TRAINING ON PAIN SENSITIVITY AND TRAPEZIUS MUSCLE MORPHOLOGY. Muscle & Nerve. 2010;41(6):836-44. | Title |
|  | Nielsen PK, Jensen BR, Darvann T, Jorgensen K, Bakke M. Quantitative ultrasound tissue characterization in shoulder and thigh muscles - a new approach. Bmc Musculoskeletal Disorders. 2006;7. | Title |
|  | Nieto MJ, Castillo JLd, Burgueño M, Alonso E, Guiñales J, Moreiras Á. Derivación de pacientes con síndrome de disfunción temporomandibular desde Atención Primaria. Rev esp cir oral maxilofac. 2018;40(3):112-9. | Title |
|  | Niide J. Dentofacial and masticatory function characteristics in anterior open bite in children and adults. Nihon Kyosei Shika Gakkai zasshi = The journal of Japan Orthodontic Society. 1986;45(1):38-47. | Full text  Other outcomes |
|  | Niide J, Fushima K, Miyagawa Y, Oda H, Shiratori M, Akimoto S, Suzuki Y. Various characteristics of dento-facial morphology and masticatory muscle function in children with anterior open-bite. Kanagawa shigaku The Journal of the Kanagawa Odontological Society. 1986;20(4):509-16. | Title |
|  | Nikitin VN, Nyashin YI, Tverier VM. The temporomandibular joint reaction and procedure of the occlusion correction. Series on Biomechanics. 2019;33(2):3-11. | Title |
|  | Nikitin VN, Tverier VM, Geletin PN. Distribution of the masticatory muscle forces of the dentofacial system during the creation of the bite force of the jaws at changing the number of considered muscles. Series on Biomechanics. 2023;37(3):38-42. | Title |
|  | Nikkuni Y, Nishiyama H, Hayashi T. Clinical significance of T2 mapping MRI for the evaluation of masseter muscle pain in patients with temporomandibular joint disorders. Oral Radiology. 2013;29(1):50-5. | Title |
|  | Nilesh K, Dharamsi R, Patil P, Mate P. Management of unilateral idiopathic masseter muscle hypertrophy with botulinum toxin type A. BMJ Case Reports. 2021;14(1). | Title |
|  | Nishi SE, Rahman NA, Basri R, Alam MK, Noor NFM, Zainal SA, Husein A. Surface Electromyography (sEMG) Activity of Masticatory Muscle (Masseter and Temporalis) with Three Different Types of Orthodontic Bracket. BioMed Research International. 2021;2021. | Title |
|  | Nishi T, Ohta M, Takano T, Ogami K, Ueda T, Sakurai K. Oral function is associated with the body and muscle mass indices of middle-aged dental patients. Clinical and Experimental Dental Research. 2022;8(1):217-24. | Title |
|  | Nishi TM, Yamashita S, Hirakawa YN, Katsuki NE, Tago M, Yamashita SI. Proliferative fasciitis/myositis involving the facial muscles including the masseter muscle: A rare cause of trismus. American Journal of Case Reports. 2019;20:1411-7. | Title |
|  | Nishida M, Inoue M, Yanai A, Matsumoto T. Malignant granular cell tumor of the masseter muscle: Case report. Journal of Oral and Maxillofacial Surgery. 2000;58(3):345-8. | Title |
|  | Nishizawa S, Ichinohe T, Kaneko Y. Tissue Blood Flow Reductions Induced by Remifentanil in Rabbits and the Effect of Naloxone and Phentolamine on These Changes. Journal of Oral and Maxillofacial Surgery. 2012;70(4):797-802. | Title |
|  | Nisi M, Izzetti R, Gabriele M, Pucci A. Oral intramuscular myxoma: case report and brief review of the literature. Oral Surg Oral Med Oral Pathol Oral Radiol. 2021;131(2):e52-e8. | Title |
|  | Nitzan DW. The process of lubrication impairment and its involvement in temporomandibular joint dics displacement. A theoretical concept. Journal of Oral and Maxillofacial Surgery. 2001;59(1):36-45. | Title |
|  | Niwlikar KB, Khare V, Nathani R, Bhayade SS, Shewale A. Bone mapping for mini-implant placement with various facial growth patterns using three dimensional volumetric tomography. Journal of Clinical and Diagnostic Research. 2018;12(12):13-8. | Title |
|  | Noback ML, Harvati K. Covariation in the Human Masticatory Apparatus. Anatomical Record-Advances in Integrative Anatomy and Evolutionary Biology. 2015;298(1):64-84. | Title |
|  | Noback ML, Harvati K. The contribution of subsistence to global human cranial variation. Journal of Human Evolution. 2015;80:34-50. | Title |
|  | Nogueira LM, Grotto IW, de Souza Moraes S, Sato FRL, Marchiori ÉC. Severe Pain and Limited Mouth Opening Due to TMJ Chondromatosis: a Case Report. SN Comprehensive Clinical Medicine. 2021;3(5):1227-32. | Title |
|  | Nokar S, Naini RB. The Effect of Superstructure Design on Stress Distribution in Peri-Implant Bone During Mandibular Flexure. International Journal of Oral & Maxillofacial Implants. 2010;25(1):31-7. | Title |
|  | Nørholt S, Pedersen T, Herlin T. Mandibular distraction osteogenesis in 23 patients with juvenile idiopathic arthritis. Journal of Oral and Maxillofacial Surgery. 2011;69(9):e-21. | Title |
|  | Noro T, Tanne K, Sakuda M. Orthodontic forces exerted by activators with varying construction bite heights. American Journal of Orthodontics and Dentofacial Orthopedics. 1994;105(2):169-79. | Title |
|  | Noviello A, Lione R, Da Ros V, Fanucci E, Cozza P. Analysis of correlation between vertical facial dimension and masseter muscle in growing patients. Dental Cadmos. 2015;83(7):446-55. | Included |
|  | Novruzov ZH, Aliyeva RG. The impact of activators used in treatment of distal malocclusions on chewing muscles. Azerbaijan Medical Journal. 2016(2):161-6. | Title |
|  | Nowair IM, Essa EF, Eid MK. A comparative evaluation of retromandibular versus Modified Risdon approach in surgical treatment of condylar fracture. Journal of Cranio-Maxillofacial Surgery. 2022;50(3):237-45. | Title |
|  | Nowak Z, Chęciński M, Nitecka-Buchta A, Bulanda S, Ilczuk-Rypuła D, Postek-Stefańska L, Baron S. Intramuscular injections and dry needling within masticatory muscles in management of myofascial pain. Systematic review of clinical trials. International Journal of Environmental Research and Public Health. 2021;18(18). | Title |
|  | Nowinski WL, Chua BC, Johnson A, Qian G, Poh LE, Yi SHW, et al. Three-dimensional interactive and stereotactic atlas of head muscles and glands correlated with cranial nerves and surface and sectional neuroanatomy. Journal of Neuroscience Methods. 2013;215(1):12-8. | Title |
|  | NuÑO‐Licona A, Cavazos E, Angeles‐Medina F. Electromyographic changes resulting from orthodontic correction of class III malocclusion. International Journal of Paediatric Dentistry. 1993;3(2):71-6. | Title |
|  | Nusrath MA, Kendall CH, Avery CM. Metastatic uterine leiomyosarcoma masquerading as a primary lesion of the masseter muscle. International Journal of Oral and Maxillofacial Surgery. 2006;35(5):466-8. | Title |
|  | O'Connor CF, Franciscus RG, Holton NE. Bite force production capability and efficiency in neandertals and modern humans. American Journal of Physical Anthropology. 2005;127(2):129-51. | Title |
|  | O'Gorman C, Kassardjian C, Baria M, Weikamp J, Van Alfen N, Boon A. Ultrasound is a sensitive technique to detect fasciculations of cranial muscles in amyotrophic lateral sclerosis (ALS). Neurology. 2016;86(16). | Title |
|  | O'Ryan F, Epker BN. Surgical orthodontics and the temporomandibular joint. II. Mandibular advancement via modified sagittal split ramus osteotomies. American Journal of Orthodontics. 1983;83(5):418-27. | Title |
|  | O'Ryan F, Epker BN. Surgical orthodontics and the temporomandibular joint. I. Superior repositioning of the maxilla. American Journal of Orthodontics. 1983;83(5):408-17. | Title |
|  | Obayashi N, Ariji Y, Goto M, Izumi M, Naitoh M, Kurita K, et al. CT analyses of the location of the maxillary third molar in relation to panoramic radiographic appearance. Oral Radiology. 2009;25(2):108-17. | Title |
|  | Ochoa-Escudero M, Juliano AF. Unilateral hypoplasia with contralateral hypertrophy of anterior belly of digastric muscle: a case report. Surgical and Radiologic Anatomy. 2016;38(8):973-4. | Title |
|  | Odajima N, Matsunaga T, Furukawa T, Tsukagoshi H. [Distribution of skeletal muscle involvement in myotonic dystrophy--a computed tomographic study]. Rinsho Shinkeigaku. 1990;30(7):707-12. | Title |
|  | Ödman A, Bresin A, Kiliaridis S. The effect of retraining hypofunctional jaw muscles on the transverse skull dimensions of adult rats. Acta Odontologica Scandinavica. 2019;77(3):184-8. | Title |
|  | Ödman A, Mavropoulos A, Kiliaridis S. Do masticatory functional changes influence the mandibular morphology in adult rats. Archives of Oral Biology. 2008;53(12):1149-54. | Title |
|  | Ödman AM, Hunt NP, Moawad HAM, Sinanan ACM, Kiliaridis S, Lewis MP. Molecular changes in detrained & retrained adult jaw muscle. European Journal of Orthodontics. 2013;35(5):659-63. | Title |
|  | Ogalde L A, Cárdenas A H, González N J, Montoya S H. Frecuencia de pseudoprognatismo y actividad del músculo masetero en pacientes fisurados. Rev méd Chile. 2000;128(9):1005-10. | Title |
|  | Ogasawara N, Kano F, Hashimoto N, Mori H, Liu Y, Xia L, et al. Factors secreted from dental pulp stem cells show multifaceted benefits for treating experimental temporomandibular joint osteoarthritis. Osteoarthritis and Cartilage. 2020;28(6):831-41. | Title |
|  | Ogawa A, Ishizaki A, Asami T, Kwon H, Fujii K, Kasama K, et al. Effectiveness of a mouth rinsing function test for evaluating the oral function of children. Pediatric Dental Journal. 2017;27(2):85-93. | Title |
|  | Ogawa T, Koyano K, Suetsugu T. The relationship between inclination of the occlusal plane and jaw closing path. Journal of Prosthetic Dentistry. 1996;76(6):576-80. | Title |
|  | Ogihara K, Nakahara R, Koyanagi S, Suda M. Treatment of a Brodie bite by lower lateral expansion: A case report and fourth year follow-up. Journal of Clinical Pediatric Dentistry. 1998;23(1):17-22. | Title |
|  | Ogino H. The influence of playing the clarinet on the dentomaxillofacial morphology and function. Ōu Daigaku shigakushi. 1990;17(2):131-54. | Title |
|  | Ogiya A, Takahashi K, Sato M, Kubo Y, Nishikawa N, Kikutani M, et al. Metastatic breast carcinoma of the abdominal wall muscle: a case report. Breast Cancer. 2015;22(2):206-9. | Title |
|  | Ögütcen-Toller M, Juniper RP. The development of the human lateral pterygoid muscle and the temporomandibular joint and related structures: A three-dimensional approach. Early Human Development. 1994;39(1):57-68. | Title |
|  | Ohara Y, Hirano H, Watanabe Y, Edahiro A, Sato E, Shinkai S, et al. Masseter muscle tension and chewing ability in older persons. Geriatrics and Gerontology International. 2013;13(2):372-7. | Title |
|  | Ohba S, Tasaki H, Tobita T, Minamizato T, Kawasaki T, Motooka N, et al. Assessment of skeletal stability of intraoral vertical ramus osteotomy with one-day maxillary-mandibular fixation followed by early jaw exercise. Journal of Cranio-Maxillofacial Surgery. 2013;41(7):586-92. | Title |
|  | Ohira A, Ono Y, Yano N, Takagi Y. The effect of chewing exercise in preschool children on maximum bite force and masticatory performance. International journal of paediatric dentistry. 2012;22(2):146‐53. | Title |
|  | Ohkura K, Harada K, Morishima S, Enomoto S. Changes in bite force and occlusal contact area after orthognathic surgery for correction of mandibular prognathism. Oral Surgery Oral Medicine Oral Pathology Oral Radiology and Endodontics. 2001;91(2):141-5. | Title |
|  | Ohmure H, Miyawaki S, Nagata J, Ikeda K, Yamasaki K, Al-Kalaly A. Influence of forward head posture on condylar position. Journal of Oral Rehabilitation. 2008;35(11):795-800. | Title |
|  | Ohnuki Y, Kawai N, Tanaka E, Langenbach GEJ, Tanne K, Saeki Y. Effects of increased occlusal vertical dimension on daily activity and myosin heavy chain composition in rat jaw muscle. Archives of Oral Biology. 2009;54(8):783-9. | Title |
|  | Ohrbach R. Disability assessment in temporomandibular disorders and masticatory system rehabilitation. Journal of Oral Rehabilitation. 2010;37(6):452-80. | Title |
|  | Ohshima A, Ariji Y, Gotoh M, Izumi M, Naitoh M, Kurita K, et al. Computed tomographic anatomy of the mandibular first and second molars and their surrounding structures in the spread of odontogenic infection. Oral Radiology. 2009;25(2):99-107. | Title |
|  | Okamoto K, Tanikawa C, Takada K. Hemodynamic Changes in the Masseter and Superior Orbicularis Oris Muscles before and after Exercise Load: A Comparison between Young Adult Women and Middle-Aged to Old Adult Women. International Journal of Dentistry. 2022;2022. | Title |
|  | Okamura E, Ikeda K, Mano-Usui F, Kawashima S, Kondo A, Inagaki N. Augmentation of Growth Hormone by Chewing in Females. Nutrients. 2023;15(16). | Title |
|  | Oki K, Ogino Y, Takamoto Y, Imai M, Takemura Y, Ayukawa Y, Koyano K. The significance of posterior occlusal support of teeth and removable prostheses in oral functions and standing motion. International Journal of Environmental Research and Public Health. 2021;18(13). | Title |
|  | Okui T, Ibaragi S, Kawai H, Sasaki A. Solitary Fibrous Tumor Arising in the Buccal Space. Case Reports in Medicine. 2019;2019. | Title |
|  | Okuno T, Suzuki H, Inoue A, Kusukawa J. Restricted Mandibular Movement Attributed to Ossification of Mandibular Depressors and Medial Pterygoid Muscles in Patients With Fibrodysplasia Ossificans Progressiva: A Report of 3 Cases. Journal of Oral and Maxillofacial Surgery. 2017;75(9):1891-8. | Title |
|  | Øland J, Jensen J, Melsen B. Factors of importance for the functional outcome in orthognathic surgery patients: A prospective study of 118 patients. Journal of Oral and Maxillofacial Surgery. 2010;68(9):2221-31. | Title |
|  | Olbrisch C, Santander P, Moser N, Klenke D, Meyer-Marcotty P, Quast A. Three-dimensional mandibular characteristics in skeletal malocclusion A cross-sectional study. Journal of Orofacial Orthopedics-Fortschritte Der Kieferorthopadie. 2022. | Abstract |
|  | Olivares HG, Pérez AG, Saucedo FL, Medina FÁ. Electromyographic changes in the masseter and temporalis muscles before and after orthognathic surgery in patients with mandibular prognathism. Journal of Craniofacial Surgery. 2019;30(5):1539-43. | Title |
|  | Oliveira LR, Borges LS, Sarmet M, Kagiyama K, Silva BO, Picinato-Pirola M, et al. Anatomical, behavioural and physiological analyses of craniofacial development by cineradiographic imaging in marmosets. Journal of Oral Rehabilitation. 2022;49(7):701-11. | Title |
|  | Oliwa A, Hocking C, Hamilton MJ, McLean J, Cumming S, Ballantyne B, et al. Masseter muscle volume as a disease marker in adult-onset myotonic dystrophy type 1. Neuromuscular Disorders. 2022;32(11-12):893-902. | Title |
|  | Olsen-Bergem H, Bjørnland T. A cohort study of patients with juvenile idiopathic arthritis and arthritis of the temporomandibular joint: Outcome of arthrocentesis with and without the use of steroids. International Journal of Oral and Maxillofacial Surgery. 2014;43(8):990-5. | Title |
|  | Olson R. Biomechanics of the Mammalian Tongue: Kinematic Analysis of Tongue Movements and Deformations during Feeding and Drinking [Dissertation/Thesis]2020. | Title |
|  | Onimaru M, Takahashi M, Shimazaki A, Kimura H, Inou N, Maki K. Verification of mechanical load generated by functional orthodontic appliances. Journal of Biomechanics. 2020;113. | Title |
|  | Ono S. Pre-treatment and post-treatment mandibular movements of patients with reversed occlusion. Nichidai koku kagaku = Nihon University journal of oral science. 1984;10(4):327-42. | Title |
|  | Oo LT, Miyamoto JJ, Takada JI, Cheng SWE, Yoshizawa H, Moriyama K. Three-dimensional characteristics of temporomandibular joint morphology and condylar movement in patients with mandibular asymmetry. Progress in Orthodontics. 2022;23(1). | Title |
|  | Oo LT, Miyamoto JJ, Takada JI, Moriyama K. Correlation between the position of the glenoid fossa and condylar translational movement in skeletal Class III mandibular asymmetry patients. European Journal of Orthodontics. 2022;44(3):294-302. | Title |
|  | Orabona GD, Abbate V, Maffia F, Sani L, Romano A, Maglitto F, et al. Bilateral coronoid hyperplasia: A case report of an intraoral endoscopically assisted coronoidectomy with CAD/CAM cutting guides. Oral and Maxillofacial Surgery Cases. 2022;8(3). | Title |
|  | Ord RA, Warburton G, Caccamese JF. Osteochondroma of the condyle: review of 8 cases. International Journal of Oral and Maxillofacial Surgery. 2010;39(6):523-8. | Title |
|  | Orhan K, Uyanik LO, Erkmen E, Kilinc Y. Unusually severe limitation of the jaw attributable to fibrodysplasia ossificans progressiva: a case report with cone-beam computed tomography findings. Oral Surg Oral Med Oral Pathol Oral Radiol. 2012;113(3):404-9. | Title |
|  | Orhan K, Yazici G, Kolsuz ME, Kafa N, Bayrakdar IS, Çelik Ö. An Artificial Intelligence Hypothetical Approach for Masseter Muscle Segmentation on Ultrasonography in Patients With Bruxism. Journal of Advanced Oral Research. 2021;12(2):206-13. | Title |
|  | Orsbon CP, Gidmark NJ, Ross CF. Dynamic Musculoskeletal Functional Morphology: Integrating diceCT and XROMM. Anatomical Record-Advances in Integrative Anatomy and Evolutionary Biology. 2018;301(2):378-406. | Title |
|  | Ortu E, Pietropaoli D, Adib F, Masci C, Giannoni M, Monaco A. Electromyographic evaluation in children orthodontically treated for skeletal Class II malocclusion: Comparison of two treatment techniques. Cranio - Journal of Craniomandibular Practice. 2019;37(2):129-35. | Title |
|  | Ortún-Terrazas J, Cegoñino J, del Palomar AP. In silico approach towards neuro-occlusal rehabilitation for the early correction of asymmetrical development in a unilateral crossbite patient. International Journal for Numerical Methods in Biomedical Engineering. 2023. | Title |
|  | Osawa K, Iwai T, Sugiyama S, Kitajima H, Baba J, Oguri S, et al. Peripheral osteoma arising from the lateral surface of the mandibular ramus. Journal of Oral and Maxillofacial Surgery Medicine and Pathology. 2018;30(3):278-80. | Title |
|  | Oshima T, Kuno H, Sekiya K, Tomita H, Kobayashi T, Kusumoto M. A case of medial pterygoid muscle metastasis of lung cancer presenting with trismus. International Cancer Conference Journal. 2019;8(4):153-6. | Title |
|  | Oshima T, Ogura M, Kikuchi T, Hori Y, Mugikura S, Higano S, et al. Involvement of pterygoid venous plexus in patulous eustachian tube symptoms. Acta Oto-Laryngologica. 2007;127(7):693-9. | Title |
|  | Österlund C, Thornell LE, Eriksson PO. Differences in fibre type composition between human masseter and biceps muscles in young and adults reveal unique masseter fibre type growth pattern. Anatomical Record. 2011;294(7):1158-69. | Title |
|  | Ota Y, Aoki T, Karakida K, Otsuru M, Kurabayashi H, Sasaki M, et al. Determination of deep surgical margin based on anatomical architecture for local control of squamous cell carcinoma of the buccal mucosa. Oral Oncology. 2009;45(7):605-9. | Title |
|  | Oukhai K, Maricic N, Schneider M, Harzer W, Tausche E. Developmental myosin heavy chain mRNA in masseter after orthognathic surgery: A preliminary study. Journal of Cranio-Maxillofacial Surgery. 2011;39(6):401-6. | Title |
|  | Owczarek JE, Lion KM, Radwan-Oczko M. Manifestation of stress and anxiety in the stomatognathic system of undergraduate dentistry students. Journal of International Medical Research. 2020;48(2). | Title |
|  | Owsley JQ, Agarwal CA. Safely Navigating Around the Facial Nerve in Three Dimensions. Clinics in Plastic Surgery. 2008;35(4):469-77. | Title |
|  | Oyen OJ, Tsay TP. A BIOMECHANICAL ANALYSIS OF CRANIOFACIAL FORM AND BITE FORCE. American Journal of Orthodontics and Dentofacial Orthopedics. 1991;99(4):298-309. | Title |
|  | Oymak Y, Okur S, Karapinar TH, Ay Y, Demir K, Gurcinar M, et al. Effectivenes of magnetic resonance imaging for measuring of pituitary iron overload in patients with thalassemia. Haematologica. 2015;100:600. | Title |
|  | Özdemir D, Polat NT, Polat S. Lipiodol UF retention in dental sialography. British Journal of Radiology. 2004;77(924):1040-1. | Title |
|  | Ozdemir R, Baran CN, Karagoz MA, Dogan S. Place of sagittal split osteotomy in mandibular surgery. Journal of Craniofacial Surgery. 2009;20(2):349-55. | Title |
|  | Ozdiler O, Orhan K, Cesur E, Köklü A, Algin O. Evaluation of temporomandibular joint, masticatory muscle, and brain cortex activity in patients treated by removable functional appliances: A prospective fMRI study. Dentomaxillofacial Radiology. 2019;48(7). | Title |
|  | Özkan NC, Ozkan F. The relationship of temporomandibular disorders with headaches: a retrospective analysis. Agri. 2011;23(1):13-7. | Title |
|  | Özsürekci C, Güngör E, Çalişkan H, Ayçiçek GŞ, Halil MG. Chewing function, sarcopenia and malnutrition in elderly patients. European Geriatric Medicine. 2019;10:S248. | Title |
|  | Özsürekci C, Kara M, Güngör AE, Ayçiçek G, Çalışkan H, Doğu BB, et al. Relationship between chewing ability and malnutrition, sarcopenia, and frailty in older adults. Nutr Clin Pract. 2022;37(6):1409-17. | Title |
|  | Pachnicz D, Ramos A. Mandibular condyle displacements after orthognathic surgery-an overview of quantitative studies. Quantitative Imaging in Medicine and Surgery. 2021;11(4):1628-50. | Title |
|  | Pachnicz D, Strózyk P. A Biomechanical Analysis of Muscle Force Changes After Bilateral Sagittal Split Osteotomy. Frontiers in Physiology. 2021;12. | Title |
|  | Pachnicz D, Strózyk P, Grygier D. Changes in muscle length and orientation after orthognathic surgeries using a bilateral sagittal split osteotomy as an example. Acta of Bioengineering and Biomechanics. 2021;23(4):127-35. | Title |
|  | Pacino GA, Redondo LM, Cocuzza S, Maniaci A, da Mosto MC, Boscolo-Rizzo P, et al. Primary hemangiopericytoma of the infratemporal fossa. Journal of Biological Regulators and Homeostatic Agents. 2020;34(2):691-5. | Title |
|  | Padmavati R, Muthuraman S, Kannan MS. Twin Block Appliance - A Review. International Journal of Life Science and Pharma Research. 2021:139-43. | Title |
|  | Padwa BL, Dentino K, Robson CD, Woo SB, Kurek K, Resnick CM. Pediatric Chronic Nonbacterial Osteomyelitis of the Jaw: Clinical, Radiographic, and Histopathologic Features. Journal of Oral and Maxillofacial Surgery. 2016;74(12):2393-402. | Title |
|  | Paglio AE, Bradley AP, Tubbs RS, Loukas M, Kozlowski PB, Dilandro AC, et al. Morphometric analysis of temporomandibular joint elements. Journal of Cranio-Maxillofacial Surgery. 2018;46(1):63-6. | Title |
|  | Pagonidis K, Raissaki M, Gourtsoyiannis N. Proliferative myositis - Value of imaging. Journal of Computer Assisted Tomography. 2005;29(1):108-11. | Title |
|  | Pai I, Chevretton EB, Haikel S, Sandison A, Connor S, Siddiqui A. Proceedings of the 154th Semon Club, 26 November 2018, ENT Department, Guy's and St Thomas' NHS Foundation Trust, London, UK. Journal of Laryngology and Otology. 2019;133(11):1-15. | Title |
|  | Pakdel A, Fialkov J, Whyne CM. High resolution bone material property assignment yields robust subject specific finite element models of complex thin bone structures. Journal of Biomechanics. 2016;49(9):1454-60. | Title |
|  | Pakdel AR, Whyne CM, Fialkov JA. Structural biomechanics of the craniomaxillofacial skeleton under maximal masticatory loading: Inferences and critical analysis based on a validated computational model. Journal of Plastic, Reconstructive and Aesthetic Surgery. 2017;70(6):842-50. | Title |
|  | Palmqvist P, Martínez-Navarro B, Pérez-Claros JA, Torregrosa V, Figueirido B, Jiménez-Arenas JM, et al. The giant hyena <i>Pachycrocuta brevirostris</i>: Modelling the bone-cracking behavior of an extinct carnivore. Quaternary International. 2011;243(1):61-79. | Title |
|  | Pan JJ, Ng WT, Zong JF, Chan LLK, O'Sullivan B, Lin SJ, et al. Proposal for the 8th edition of the AJCC/UICC staging system for nasopharyngeal cancer in the era of intensity-modulated radiotherapy. Cancer. 2016;122(4):546-58. | Title |
|  | Pan Y, Wang Y, Li G, Chen S, Xu T. Validity and reliability of masseter muscles segmentation from the transverse sections of Cone-Beam CT scans compared with MRI scans. International Journal of Computer Assisted Radiology and Surgery. 2022;17(4):751-9. | Abstract |
|  | Pancherz H. Long-term effects of activator (Andresen appliance) treatment. A clinical, biometric, cephalometric roentgenographic and functional analysis. Odontologisk revy Supplement. 1976;35:1-70. | Title |
|  | Pancherz H. Relapse after activator treatment. A biometric, cephalometric, and electromyographic study of subjects with and without relapse of overjet. American Journal of Orthodontics. 1977;72(5):499-512. | Title |
|  | Pancherz H. The Herbst appliance--its biologic effects and clinical use. Am J Orthod. 1985;87(1):1-20. | Title |
|  | Pancherz H, Winnberg A, Westesson PL. Masticatory muscle activity and hyoid bone behavior during cyclic jaw movements in man. A synchronized electromyographic and videofluorographic study. American Journal of Orthodontics. 1986;89(2):122-31. | Title |
|  | Pantoja LLQ, Lustosa M, Yamaguti PM, Rosa LS, Leite AF, Figueiredo PTS, et al. Pamidronate Therapy Increases Trabecular Bone Complexity of Mandibular Condyles in Individuals with Osteogenesis Imperfecta. Calcified Tissue International. 2022;110(3):303-12. | Title |
|  | Paolo CDI, Papi MDP, Falisi G, Pompa G, Santilli V, Polimeni A, Fiorini A. Subjects with temporomandibular joint disc displacement and body posture assessment via rasterstereography: A pilot case-control study. European Review for Medical and Pharmacological Sciences. 2020;24(17):8703-12. | Title |
|  | Paoloni V, Lione R, Farisco F, Halazonetis DJ, Franchi L, Cozza P. Morphometric covariation between palatal shape and skeletal pattern in Class II growing subjects. European Journal of Orthodontics. 2017;39(4):371-6. | Title |
|  | Papagiannis A, Halazonetis DJ. Shape variation and covariation of upper and lower dental arches of an orthodontic population. European Journal of Orthodontics. 2016;38(2):202-11. | Title |
|  | Paphangkorakit J, Osborn JW. Effect of jaw opening on the direction and magnitude of human incisal bite forces. Journal of Dental Research. 1997;76(1):561-7. | Title |
|  | Papuc SM, Hackmann K, Andrieux J, Vincent-Delorme C, Budişteanu M, Arghir A, et al. Microduplications of 3p26.3p26.2 containing CRBN gene in patients with intellectual disability and behavior abnormalities. European Journal of Medical Genetics. 2015;58(5):319-23. | Title |
|  | Park JC, Shin HS, Cha JY, Park JT. A three-dimensional finite element analysis of the relationship between masticatory performance and skeletal malocclusion. Journal of Periodontal and Implant Science. 2015;45(1):8-13. | Abstract |
|  | Park JW, Song HH, Roh HS, Kim YK, Lee JY. Correlation between clinical diagnosis based on RDC/TMD and MRI findings of TMJ internal derangement. International Journal of Oral and Maxillofacial Surgery. 2012;41(1):103-8. | Title |
|  | Park KM, Choi E, Kwak EJ, Kim S, Park W, Jeong JS, Kim KD. The relationship between masseter muscle thickness measured by ultrasonography and facial profile in young Korean adults. Imaging Science in Dentistry. 2018;48(3):213-21. | Full text  No skeletal patterns |
|  | Park MK, Cho SM, Yun KI, Park JU. Change in bite force and electromyographic activity of masticatory muscle in accordance with change of occlusal plane. Journal of Oral and Maxillofacial Surgery. 2012;70(8):1960-7. | Abstract |
|  | Park SB, Kim YI, Hwang DS, Lee JY. Midfacial soft-tissue changes after mandibular setback surgery with or without paranasal augmentation: Cone-beam computed tomography (CBCT) volume superimposition. Journal of Cranio-Maxillofacial Surgery. 2013;41(2):119-23. | Title |
|  | Park W, Kim BC, Yu HS, Yi CK, Lee SH. Architectural characteristics of the normal and deformity mandible revealed by three-dimensional functional unit analysis. Clinical Oral Investigations. 2010;14(6):691-8. | Title |
|  | Parks LR, Buschang PH, Alexander RA, Dechow P, Rossouw PE. Masticatory exercise as an adjunctive treatment for hyperdivergent patients. Angle Orthodontist. 2007;77(3):457-62. | Title |
|  | Parmenter MD, Nelson JP, Weigel SE, Gray MM, Payseur BA, Vinyard CJ. Masticatory Apparatus Performance and Functional Morphology in the Extremely Large Mice from Gough Island. Anatomical Record-Advances in Integrative Anatomy and Evolutionary Biology. 2020;303(1):167-79. | Title |
|  | Paschetta C, de Azevedo S, Castillo L, Martínez-Abadías N, Hernández M, Lieberman DE, González-José R. The influence of masticatory loading on craniofacial morphology: A test case across technological transitions in the Ohio valley. Am J Phys Anthropol. 2010;141(2):297-314. | Abstract |
|  | Paschetta C, González-José R. Cambios de forma y tamaño en el cráneo humano. Runa. 2022;43(2):87-116. | Title |
|  | Pastana SdG, Costa SdM, Chiappetta ALdML. Análise da mastigação em indivíduos que apresentam mordida cruzada unilateral na faixa-etária de 07 a 12 anos. Revista CEFAC. 2007;9(3):351-7. | Title |
|  | Patel AA, Pobre TT, Weiss L, Marzloff G. Laser treatment of trismus after gunshot wound to face: A case report. PM and R. 2014;6(9):S232. | Title |
|  | Patel B, Benninger B. Clinical anatomy of the Vazirani-Akinosi method for pain relief with and without an associated concussion. Clinical Anatomy. 2011;24(8):1033. | Title |
|  | Patel HP, Moseley HC, Noar JH. Cephalometric determinants of successful functional appliance therapy. Angle Orthodontist. 2002;72(5):410-7. | Title |
|  | Patel M, Scott N, Newlands C. Case of tuberculosis of the temporomandibular joint. British Journal of Oral and Maxillofacial Surgery. 2012;50(1):e1-e3. | Title |
|  | Patel R, McClay J, Veltkamp D, Booth T. Chronic Recurrent Multifocal Osteomyelitis (CRMO) of mandible in the pediatric population: Spectrum of imaging findings with clinicopathological correlation. Pediatric Radiology. 2013;43:S308-S9. | Title |
|  | Paterson T, McMahon JD, Wales C, Fogg QA. Anterolateral corridor approach to extended maxillectomy. British Journal of Oral and Maxillofacial Surgery. 2011;49:S11. | Title |
|  | Patini R, Gallenzi P, Lione R, Cozza P, Cordaro M. Ultrasonographic Evaluation of The Effects of Orthodontic or Functional Orthopaedic Treatment on Masseter Muscles: A Systematic Review and Meta-Analysis. Medicina (Kaunas). 2019;55(6). | Title |
|  | Pawlaczyk-Kamieńska T, Kulczyk T, Pawlaczyk-Wróblewska E, Borysewicz-Lewicka M, Niedziela M. Limited mandibular movements as a consequence of unilateral or asymmetrical temporomandibular joint involvement in juvenile idiopathic arthritis patients. Journal of Clinical Medicine. 2020;9(8):1-12. | Title |
|  | Peacock ZS, Chapman PH, Gupta R, Kaban LB. Replication of ancient Egyptian osteotomies of the facial skeleton: Insights into the mummification process. International Journal of Oral and Maxillofacial Surgery. 2011;40(11):1301-6. | Title |
|  | Peck CC. Biomechanics of occlusion - implications for oral rehabilitation. Journal of Oral Rehabilitation. 2016;43(3):205-14. | Title |
|  | Peck CC, Wirianski A, Murray GM. Jaw motor plasticity in health and disease. Computer Methods in Biomechanics and Biomedical Engineering. 2010;13(4):455-8. | Title |
|  | Pedrazzi ME. Treating the open bite. J Gen Orthod. 1997;8(1):5-16. | Title |
|  | Pedro JRS, Pérez BC, Ñíguez BF, Vicente VVS. Arteriovenous malformations of the temporalis muscle: A comprehensive review. Operative Neurosurgery. 2018;14(4):325-40. | Title |
|  | Pedullà E, Meli GA, Garufi A, Cascone P, Mandalà ML, Deodato L, Palazzo G. Morphometric evaluation of the temporomandibular joint and the masticatory spaces: the role of high-definition MRI. Minerva Stomatol. 2009;58(4):127-43. | Title |
|  | Pehlivan UA, Somay E, Yilmaz B, Besen AA, Mertsoylu H, Selek U, Topkan E. Pretreatment Masseter Muscle Volume Predicts Survival in Locally Advanced Nasopharyngeal Carcinoma Patients Treated with Concurrent Chemoradiotherapy. Journal of Clinical Medicine. 2023;12(21). | Title |
|  | Peng CY, Lu MY. Efficacy of hyaluronic acid injection in superior joint space for the treatment of temporomandibular disorder in Taiwan. International Journal of Oral and Maxillofacial Surgery. 2017;46:362. | Title |
|  | Peng S, Behbahani M, Sharma S, Speck S, Wadhwani NR, Rastatter JC, Alden TD. Pediatric benign triton tumor of trigeminal nerve: a case report and literature review. Child's Nervous System. 2022;38(11):2055-61. | Title |
|  | Pepicelli A, Woods M, Briggs C. The mandibular muscles and their importance in orthodontics: A contemporary review. American Journal of Orthodontics and Dentofacial Orthopedics. 2005;128(6):774-80. | Full text  Review |
|  | Pepper T, Falla L, Brennan PA. Soft tissue giant cell tumour of low malignant potential arising in the masseter-A rare entity in the head and neck. British Journal of Oral and Maxillofacial Surgery. 2010;48(2):149-51. | Title |
|  | Pereira JR, Bachesk AB, Pierri RAG, Iwaki Filho L. Myositis Ossificans Traumatica of the Temporal Muscle Treated by Bilateral Coronoidectomy and Aggressive Physiotherapy: A Case Report. Craniomaxillofacial Trauma and Reconstruction Open. 2021;6. | Title |
|  | Pereira LJ, Duarte Gaviao MB, Van Der Bilt A. Influence of oral characteristics and food products on masticatory function. Acta Odontologica Scandinavica. 2006;64(4):193-201. | Title |
|  | Pereira LJ, Gavião MB, Bonjardim LR, Castelo PM, van der Bilt A. Muscle thickness, bite force, and craniofacial dimensions in adolescents with signs and symptoms of temporomandibular dysfunction. Eur J Orthod. 2007;29(1):72-8. | Title |
|  | Perez PI, Hendershot K, Teixeira JC, Hohman MH, Adidharma L, Moody M, et al. Analysis of Cephalometric Points in Male and Female Mandibles: An Application to Gender-Affirming Facial Surgery. J Craniofac Surg. 2023;34(4):1278-82. | Title |
|  | Perez-Flecha F, Sánchez-Jáuregui E, Villegas D, García-Serrano G, Sagüillo K, Page I, et al. Our experience with modified Risdon approach for subcondylar mandibular fractures and review of literature. International Journal of Oral and Maxillofacial Surgery. 2015;44:e123. | Title |
|  | Perinetti G, Türp JC, Primožič J, Di Lenarda R, Contardo L. Associations between the masticatory system and muscle activity of other body districts. A meta-analysis of surface electromyography studies. Journal of Electromyography and Kinesiology. 2011;21(6):877-84. | Title |
|  | Perry JMG, Kay RF, Vizcaíno SF, Bargo MS. Tooth Root Size, Chewing Muscle Leverage, and the Biology of <i>Homunculus patagonicus</i> (Primates) from the late early Miocene of Patagonia. Ameghiniana. 2010;47(3):355-71. | Title |
|  | Perry JMG, Prufrock KA. Muscle Functional Morphology in Paleobiology: The Past, Present, and Future of "Paleomyology". Anatomical Record-Advances in Integrative Anatomy and Evolutionary Biology. 2018;301(3):538-55. | Title |
|  | Peterson A, Benninger B. Clinical anatomy of the Gow-Gates local anesthesia technique. Clinical Anatomy. 2011;24(8):1033-4. | Title |
|  | Peterson TM, Rugh JD, McIver JE. Mandibular rest position in subjects with high and low mandibular plane angles. American Journal of Orthodontics. 1983;83(4):318-20. | Title |
|  | Petrović D, Vujkov S, Petronijević B, Šarčev I, Stojanac I. Examination of the bioelectrical activity of the masticatory muscles during Angle's Class II division 2 therapy with an activator. Vojnosanit Pregl. 2014;71(12):1116-22. | Title |
|  | Pfeiffer JP, Grobéty D. The Class II malocclusion: Differential diagnosis and clinical application of activators, extraoral traction, and fixed appliances. American Journal of Orthodontics. 1975;68(5):499-544. | Title |
|  | Philips C, Terrie L, Thorrez L. Decellularized skeletal muscle: A versatile biomaterial in tissue engineering and regenerative medicine. Biomaterials. 2022;283. | Title |
|  | Phillips V, Ferreira J. Benefits of incorporating point of care ultrasound in the anaesthetic management of a dog requiring surgical correction of traumatic zygomatic fracture causing pseudoankylosis and severe dental malocclusion. Veterinary Record Case Reports. 2022;10(2). | Title |
|  | Piancino MG, Falla D, Merlo A, Vallelonga T, De Biase C, Dalessandri D, Debernardi C. Effects of therapy on masseter activity and chewing kinematics in patients with unilateral posterior crossbite. Archives of Oral Biology. 2016;67:61-7. | Title |
|  | Piancino MG, Farina D, Talpone F, Merlo A, Bracco P. Muscular activation during reverse and non-reverse chewing cycles in unilateral posterior crossbite. European Journal of Oral Sciences. 2009;117(2):122-8. | Title |
|  | Piancino MG, Isola G, Merlo A, Dalessandri D, Debernardi C, Bracco P. Chewing pattern and muscular activation in open bite patients. Journal of Electromyography and Kinesiology. 2012;22(2):273-9. | Title |
|  | Piancino MG, Tortarolo A, Di Benedetto L, Crincoli V, Falla D. Chewing Patterns and Muscular Activation in Deep Bite Malocclusion. Journal of Clinical Medicine. 2022;11(6). | Title |
|  | Piancino MG, Vallelonga T, Debernardi C, Bracco P. Deep bite: A case report with chewing pattern and electromyographic activity before and after therapy with function generating bite. European Journal of Paediatric Dentistry. 2013;14(2):156-9. | Title |
|  | Picchioni P, Soli P, Pirini D. Treatment of orofacial musculature imbalance. Mondo ortodontico. 1990;15(1):71-90. | Title |
|  | Piccolo L, Pichiecchio A, Alfonsi E, Foli A, Mariotto S, Piccolo G, et al. Magnetic resonance imaging evidence of posterior column degeneration in a patient with sensory ganglionopathy following low-dose bortezomib and thalidomide treatment for multiple myeloma. Journal of the Peripheral Nervous System. 2017;22:S30. | Title |
|  | Pidcock FS, Wise JM, Christensen JR. Treatment of severe post-traumatic bruxism with botulinum toxin-A: Case report. Journal of Oral and Maxillofacial Surgery. 2002;60(1):115-7. | Title |
|  | Pihut M, Gala A, Obuchowicz R, Chmura K. Influence of Ultrasound Examination on Diagnosis and Treatment of Temporomandibular Disorders. Journal of Clinical Medicine. 2022;11(5). | Title |
|  | Pihut M, Gornicki M, Orczykowska M, Zarzecka E, Ryniewicz W, Gala A. The application of radiofrequency waves in supportive treatment of temporomandibular disorders. Pain research & management. 2020;2020:6195601. | Title |
|  | Pilley JR, Mohlin B, Shaw WC, Kingdon A. A survey of craniomandibular disorders in 500 19-year-olds. European Journal of Orthodontics. 1997;19(1):57-70. | Title |
|  | Pinares Toledo J, Marileo Zagal R, Bruce Castillo L, Villanueva Conejeros R. Is the buccal compartment a masticatory space extension or an anatomic space in itself? Evidence based on medical images and human cadaver dissection. Oral Radiology. 2018;34(1):49-55. | Title |
|  | Pinheiro M, Alves JL. The feasibility of a custom-made endoprosthesis in mandibular reconstruction: Implant design and finite element analysis. Journal of Cranio-Maxillofacial Surgery. 2015;43(10):2116-28. | Title |
|  | Pirelli P, Pirelli M, Giancotti A, Mampieri G. Stomatognathic system rehabilitation: “the gnatological pre-treatment”. Journal of Biological Regulators and Homeostatic Agents. 2022;36:29-40. | Title |
|  | Pirttiniemi P. Normal and increased functional asymmetries in the craniofacial area. Acta Odontologica Scandinavica. 1998;56(6):342-5. | Title |
|  | Pissulin CNA, Bérzin F, de Faria Negrão Júnior R, Oliveira ASB, Pissulin FDM. Electromyography of the temporalis and masseter muscles in children with right unilateral crossbite. Acta Scientiarum - Health Sciences. 2010;32(2):199-204. | Title |
|  | Pitman KT, Bell D. Chondrolipoma of head and neck: Case report and review of literature. Head and Neck. 2013;35(1):E18-E20. | Title |
|  | Plenier V, Mercier J, Delaire J. Sagittal osteotomy of the mandibular ramus. Advantages of Dal Pont's second osteotomy design. Annales de Chirurgie Plastique et Esthetique. 1985;30(3):263-7. | Title |
|  | Plesh O, Meyerhoff DJ, Weiner MW. PHOSPHORUS MAGNETIC-RESONANCE SPECTROSCOPY OF HUMAN MASSETER MUSCLE. Journal of Dental Research. 1995;74(1):338-44. | Abstract |
|  | Pokhojaev A, Avni H, Sella-Tunis T, Sarig R, May H. Changes in human mandibular shape during the Terminal Pleistocene-Holocene Levant. Scientific Reports. 2019;9. | Title |
|  | Poll LW, Koch JA, Vom Dahl S, Sarbia M, Niederau C, Häussinger D, Mödder U. Type I Gaucher disease: Extraosseous extension of skeletal disease. Skeletal Radiology. 2000;29(1):15-21. | Title |
|  | Pontes HAR, Pontes FSC, E Silva BTC, Kato AMB, De Freitas Silva BS. Congenital infantile fibromatosis of the cheek: Report of a rare case and differential diagnosis. International Journal of Oral and Maxillofacial Surgery. 2011;40(11):1309-13. | Title |
|  | Ponvel K, Panneerselvam E, Balasubramanian S, Krishna Kumar Raja VB. Evaluation of labial versus labio-inferior lines of osteosynthesis using 3D miniplate for fractures of anterior mandible: A finite element analysis with a pilot clinical trial. Chinese Journal of Traumatology - English Edition. 2019;22(5):261-9. | Title |
|  | Popovic KS, Kocar M. Imaging findings in bisphosphonate-induced osteonecrosis of the jaws. Radiology and Oncology. 2010;44(4):215-9. | Title |
|  | Porro LB, Ross CF, Iriarte-Diaz J, O'Reilly JC, Evans SE, Fagan MJ. <i>In vivo</i> cranial bone strain and bite force in the agamid lizard <i>Uromastyx geyri</i>. Journal of Experimental Biology. 2014;217(11):1983-92. | Title |
|  | Portelli M, Matarese G, Militi A, Nucera R, Triolo G, Cordasco G. Myotonic dystrophy and craniofacial morphology: clinical and instrumental study. European Journal of Paediatric Dentistry. 2009;10(1):19-22. | Title |
|  | Postnikov MA, Trunin DA, Nesterov AM, Sadykov MI, Potapov VP, Gabdrafikov RR, Pankratova NV. Use of occlusal digital splint for treating the patients with temporomandibular joint dysfunction and planning orthopedic treatment. Russian Open Medical Journal. 2020;9(2). | Title |
|  | Poza MN, Connolly N, Isgren C, Talbot A. <i>Fusobacterium necrophorum</i> and <i>Actinomyces</i> spp. masseter muscle abscessation in an adult alpaca. Veterinary Record Case Reports. 2023;11(1). | Title |
|  | Prabhu NT, Munshi AK. Measurement of masseter and temporalis muscle thickness using ultrasonographic technique. The Journal of clinical pediatric dentistry. 1994;19(1):41-4. | Full text  No ceph. categorization |
|  | Prabhu R, Mandel L. Simultaneous Bilateral Hypertrophies of the Parotid Gland and Masseter Muscle: Case Report. Journal of Oral and Maxillofacial Surgery. 2017;75(1):149-52. | Title |
|  | Prabu NP, Balakrishnan K, Vigneswaran T, Dakir A, Saravanakumar B. Local spread of advanced buccal mucosa cancer - A review with 8 patients. Research Journal of Pharmaceutical, Biological and Chemical Sciences. 2017;8(4):758-64. | Title |
|  | Prado DGA, Berretin-Felix G, Migliorucci RR, Bueno MRS, Rosa RR, Polizel M, et al. Effects of orofacial myofunctional therapy on masticatory function in individuals submitted to orthognathic surgery: A randomized trial. Journal of Applied Oral Science. 2018;26. | Title |
|  | Prado FB, Freire AR, Rossi AC, Caria PHF. Finite element analysis of the three support pillars in human craniofacial skeleton. Journal of Biomechanics. 2012;45:S179. | Title |
|  | Prado FB, Freire AR, Rossi AC, Ledogar JA, Smith AL, Dechow PC, et al. Review of <i>In Vivo</i> Bone Strain Studies and Finite Element Models of the Zygomatic Complex in Humans and Nonhuman Primates: Implications for Clinical Research and Practice. Anatomical Record-Advances in Integrative Anatomy and Evolutionary Biology. 2016;299(12):1753-78. | Title |
|  | Prajapati VK, Pulikkotil SJ, Nath S. Myocysticercosis of the masseter muscle. Journal of the College of Physicians and Surgeons Pakistan. 2019;29(2):196. | Title |
|  | Prakash S, Shamim SA, Singh TP, Tripathi M, Kumar R, Bal CS. Role of FDG PET-CT in the evaluation of extraskeletal involvement in plasmacytoma. Indian Journal of Nuclear Medicine. 2022;37(5):S37. | Title |
|  | Prates LdS, Gois M, Berwig LC, Blanco-Dutra AP, Busanello-Stella AR, Silva AMTd. Avaliação clínica e eletromiográfica da mastigação nos diferentes padrões de crescimento facial. Revista CEFAC. 2016;18(1):104-12. | Full text  Other outcomes |
|  | Preuschoft H, Witzel U. A biomechanical approach to craniofacial shape in primates, using FESA. Annals of Anatomy. 2004;186(5-6):397-404. | Title |
|  | Preuschoft H, Witzel U. Functional structure of the skull in hominoidea. Folia Primatologica. 2004;75(4):219-52. | Title |
|  | Preuschoft H, Witzel U. Functional shape of the skull in vertebrates: Which forces determine skull morphology in lower primates and ancestral synapsids? Anatomical Record Part a-Discoveries in Molecular Cellular and Evolutionary Biology. 2005;283A(2):402-13. | Title |
|  | Proctor AD, DeVincenzo JP. Masseter muscle position relative to dentofacial form. Angle Orthodontist. 1970;40(1):37-44. | Full text  Other methods |
|  | Proffit WR, Fields HW. Occlusal Forces in Normal- and Long-face Children. Journal of Dental Research. 1983;62(5):571-4. | Title |
|  | Proffit WR, Turvey TA, Fields HW, Phillips C. The effect of orthognathic surgery on occlusal force. Journal of Oral and Maxillofacial Surgery. 1989;47(5):457-63. | Title |
|  | Prufrock K. Ontogeny of the Masticatory System in Strepsirrhines [Dissertation/Thesis]2020. | Title |
|  | Pu Z, Zhang Y, Yang J, Liu T, Zhang Y, Yang Q, et al. Mandibular angle ostectomy for chinese women: Approaches and extent determined by cephalometric analysis. Journal of Craniofacial Surgery. 2009;20(1):105-10. | Title |
|  | Pullinger AG, Seligman DA. Overbite and overjet characteristics of refined diagnostic groups of temporomandibular disorder patients. American Journal of Orthodontics and Dentofacial Orthopedics. 1991;100(5):401-15. | Title |
|  | Punde P, Nilesh K, Patil P. Management of Chronic Recurrent TMS Dislocation; Evaluation of Functional Outcomes and MRI Findings of As theorocenses and ABI into Joint space. European Journal of Molecular and Clinical Medicine. 2022;9(7):4829-50. | Title |
|  | Qi K, Guo SX, Xu Y, Deng Q, Liu L, Li B, Wang MQ. An investigation of the simultaneously recorded occlusal contact and surface electromyographic activity of jaw-closing muscles for patients with temporomandibular disorders and a scissors-bite relationship. Journal of Electromyography and Kinesiology. 2016;28:114-22. | Title |
|  | Quiudini PR, Pozza DH, Pinto AD, de Arruda MF, Guimaraes AS. Differences in bite force between dolichofacial and brachyfacial individuals: Side of mastication, gender, weight and height. Journal of Prosthodontic Research. 2017;61(3):283-9. | Abstract |
|  | R.Naik A. Ultrasonographic Assessment of Fibrotic Bands, Muscle Thickness and Vascularity in Oral Submucous Fibrosis [Dissertation/Thesis]2018. | Title |
|  | Raadsheer MC, Kiliaridis S, Van Eijden TM, Van Ginkel FC, Prahl-Andersen B. Masseter muscle thickness in growing individuals and its relation to facial morphology. Arch Oral Biol. 1996;41(4):323-32. | Full text  No skeletal patterns |
|  | Raadsheer MC, van Eijden T, van Ginkel FC, Prahl-Andersen B. Contribution of jaw muscle size and craniofacial morphology to human bite force magnitude. Journal of Dental Research. 1999;78(1):31-42. | Full text  Other outcomes |
|  | Raadsheer MC, van Eijden T, van Ginkel FC, Prahl-Andersen B. Human jaw muscle strength and size in relation to limb muscle strength and size. European Journal of Oral Sciences. 2004;112(5):398-405. | Abstract |
|  | Raadsheer MC, Van Eijden TM, Van Spronsen PH, Van Ginkel FC, Kiliaridis S, Prahl-Andersen B. A comparison of human masseter muscle thickness measured by ultrasonography and magnetic resonance imaging. Arch Oral Biol. 1994;39(12):1079-84. | Abstract |
|  | Rafferty KL, Herring SW, Artese F. Three-dimensional loading and growth of the zygomatic arch. Journal of Experimental Biology. 2000;203(14):2093-104. | Title |
|  | Rafferty KL, Liu ZJ, Ye WM, Navarrete AL, Nguyen TT, Salamati A, Herring SW. Botulinum toxin in masticatory muscles: Short- and long-term effects on muscle, bone, and craniofacial function in adult rabbits. Bone. 2012;50(3):651-62. | Title |
|  | Ragbir M, Mohammed F, Dunaway DJ, Chippindale AJ, Latimer J, McLean NR. Prediction of the position of the intraparotid portion of the facial nerve on MRI and CT. British Journal of Plastic Surgery. 2002;55(5):376-9. | Title |
|  | Rahemi H, Nigam N, Wakeling JM. Regionalizing muscle activity causes changes to the magnitude and direction of the force from whole muscles-a modeling study. Frontiers in Physiology. 2014;5. | Title |
|  | Rai A, Sahu S, Hoogar M. Myositis ossificans of masseter muscle: A rare case report. Virchows Archiv. 2020;477(SUPPL 1):S321. | Title |
|  | Rai S, Nandy K, Bhatt S, Patel D, Mithi M, Rathod P. Surgical outcomes of T4b oral cancers: assessment of prognostic factors and a need to re-evaluate the current staging system. International Journal of Oral and Maxillofacial Surgery. 2023;52(2):143-51. | Title |
|  | Rajamoorthy SN, Hemavathy. Botulinum Toxin-A injections into Facial Muscles for the treatment of Temporomandibular Disorders and Bruxism: A Systematic Review. Journal of Population Therapeutics and Clinical Pharmacology. 2023;30(16):e229-e40. | Title |
|  | Rajaram PC, Naidu ME, Rao SP. M-Mode echomyography - A new technique for the functional assessment of striated muscles. Indian Journal of Radiology and Imaging. 2002;12(4):537-41. | Title |
|  | Ralli M, di Stadio A, Greco A, Altissimi G, Mazzei F, Turchetta R, et al. Development of progressive hearing loss and tinnitus in a patient with myasthenia gravis: an overlooked comorbidity? Hearing, Balance and Communication. 2017;15(4):260-6. | Title |
|  | Rando C, Hillson S, Antoine D. Changes in mandibular dimensions during the mediaeval to post-mediaeval transition in London: A possible response to decreased masticatory load. Archives of Oral Biology. 2014;59(1):73-81. | Title |
|  | Rani S, Ravi MS. Masseter muscle thickness in different skeletal morphology: an ultrasonographic study. Indian J Dent Res. 2010;21(3):402-7. | Included |
|  | Raoul G, Rowlerson A, Sciote J, Codaccioni E, Stevens L, Maurage CA, et al. Masseter myosin heavy chain composition varies with mandibular asymmetry. J Craniofac Surg. 2011;22(3):1093-8. | Abstract |
|  | Raparia K, Lin JW, Donovan D, Vrabec JT, Zhai Q, Ayala AA, Ro JY. Chondroblastoma-like chondroma of soft tissue: Report of the first case in the base of skull. Annals of Diagnostic Pathology. 2013;17(3):298-301. | Title |
|  | Raphael KG, Tadinada A, Bradshaw JM, Janal MN, Sirois DA, Chan KC, Lurie AG. Osteopenic consequences of botulinum toxin injections in the masticatory muscles: a pilot study. J Oral Rehabil. 2014;41(8):555-63. | Title |
|  | Rasheed SA, Prabhu NT, Munshi AK. Electromyographic and ultrasonographic observations of masseter and anterior temporalis muscles in children. Journal of Clinical Pediatric Dentistry. 1996;20(2):127-32. | Full text  No skeletal patterns |
|  | Rashid A, Roatta S. Differential control of blood flow in masseter and biceps brachii muscles during stress. Archives of Oral Biology. 2022;141. | Title |
|  | Ratansi R, Fabbroni G, Kanatas A. Myositis in the head and neck: challenges in diagnosis and management. British Journal of Oral and Maxillofacial Surgery. 2017;55(7):722-3. | Title |
|  | Rauso R, Colella G, Franco R, Ronchi A, Chirico F. Ossified Carcinoma Ex Pleomorphic Adenoma in accessory lobe of parotid gland: Complexity in clinical, imaging and histologic diagnosis and minimally invasive surgery. Oral Oncology. 2019;92:95-8. | Title |
|  | Raustia AM, Oikarinen KS. Changes in electric activity of masseter and temporal muscles after mandibular sagittal split osteotomy. International Journal of Oral and Maxillofacial Surgery. 1994;23(3):180-4. | Title |
|  | Ravi Kiran BS, Mehta S, Chaudhary R, Breh R. Cysticercosis involving the masseter muscle: A rare anatomical location. Advances in Oral and Maxillofacial Surgery. 2023;11. | Title |
|  | Ravikumar PAT, Dinesh SPS. Identifying cortical plate inclination as a parameter to re-assess transverse discrepancies in untreated adults with different vertical facial heights-A retrospective CBCT study. Orthodontics & Craniofacial Research. 2022;25(2):219-25. | Title |
|  | Ravosa MJ, Klopp EB, Pinchoff J, Stock SR, Hamrick MW. Plasticity of mandibular biomineralization in myostatin-deficient mice. Journal of Morphology. 2007;268(3):275-82. | Title |
|  | Ravosa MJ, López EK, Menegaz RA, Stock SR, Stack MS, Hamrick MW. Using "Mighty Mouse" to understand masticatory plasticity:: myostatin-deficient mice and musculoskeletal function. Integrative and Comparative Biology. 2008;48(3):345-59. | Title |
|  | Ravosa MJ, Ross CF, Williams SH, Costley DB. Allometry of Masticatory Loading Parameters in Mammals. Anatomical Record-Advances in Integrative Anatomy and Evolutionary Biology. 2010;293(4):557-71. | Title |
|  | Raymond JL. Occlusal justification of early treatment of Class III malocclusions. L' Orthodontie française. 2006;77(2):207-12. | Title |
|  | Reddi SP, Morales MJ, Addante RR. Solitary lesion in the masseter muscle. Journal of Oral and Maxillofacial Surgery. 2001;59(1):71-5. | Title |
|  | Reddy GTP. "Measurement of Maximum Bite Force in Different Individuals with Varying Occlusal Themes”-An in Vivo Study [Dissertation/Thesis]2010. | Title |
|  | Reey RW, Eastwood A. The passive activator: Case selection, treatment response, and corrective mechanics. American Journal of Orthodontics. 1978;73(4):378-409. | Title |
|  | Regalo SCH, Lucas BD, Díaz-Serrano KV, Frota NPR, Regalo IH, Nassar MSP, et al. Analysis of the stomatognathic system of children according orthodontic treatment needs. Journal of Orofacial Orthopedics-Fortschritte Der Kieferorthopadie. 2018;79(1):39-47. | Title |
|  | Regalo SCH, Santds CM, Vitti M, Regalo CA, de Vasconcelos PB, Mestriner W, et al. Evaluation of molar and incisor bite force in indigenous compared with white population in Brazil. Archives of Oral Biology. 2008;53(3):282-6. | Title |
|  | Regalo SCH, Vitti M, Semprini M, Rosa LB, Martinez FHRM, Santos CM, Hallak JEC. Electromyographic analysis of the masseter and temporal muscles in oralized deaf individuals. Electromyography and Clinical Neurophysiology. 2006;46(4):217-22. | Title |
|  | Regulski PA, Zielinski J, Szopinski KT. Temporomandibular Disk Dislocation Impacts the Stomatognathic System: Comparative Study Based on Biexponential Quantitative T2 Maps. Journal of Clinical Medicine. 2022;11(6). | Title |
|  | Reicheneder C, Proff P, Baumert U, Gedrange T. Comparison of maximum mouth-opening capacity and condylar path length in adults and children during the growth period. Annals of Anatomy-Anatomischer Anzeiger. 2008;190(4):344-50. | Title |
|  | Reiser PJ, Bicer S, Chen Q, Zhu L, Quan N. Masticatory ('superfast') myosin heavy chain and embryonic/atrial myosin light chain 1 in rodent jaw-closing muscles. Journal of Experimental Biology. 2009;212(16):2511-9. | Title |
|  | Reiser V, Alterman M, Shlomi B, Issakov J, Dagan Y, Kleinman S, et al. Oral intravascular fasciitis: a rare maxillofacial lesion. Oral Surg Oral Med Oral Pathol Oral Radiol. 2012;114(2):e40-4. | Title |
|  | Reiter AM, Schwarz T. Computed tomographic appearance of masticatory myositis in dogs: 7 cases (1999-2006). Javma-Journal of the American Veterinary Medical Association. 2007;231(6):924-30. | Title |
|  | Remijn L, Groen BE, Speyer R, van Limbeek J, Vermaire JA, van den Engel-Hoek L, Nijhuis-van der Sanden MWG. Can mastication in children with cerebral palsy be analyzed by clinical observation, dynamic ultrasound and 3D kinematics? Journal of Electromyography and Kinesiology. 2017;32:22-9. | Title |
|  | Remy F, Godio-Raboutet Y, Verna E, Gorincour G, Bonnaure P, Adalian P, et al. Characterization of the perinatal mandible growth pattern: preliminary results. Surgical and Radiologic Anatomy. 2018;40(6):667-79. | Title |
|  | Renner E, Thatcher G. Combined Gap and Interpositional Arthroplasty Utilizing Three-Dimensional Printed Model in a Dog with Temporomandibular Joint Ankylosis and Pseudoankylosis. Journal of Veterinary Dentistry. 2022;39(3):284-9. | Title |
|  | Reynolds AK, Nickel JC, Liu Y, Leeper DK, Riffel KM, Liu H, Iwasaki LR. Sex differences in jaw muscle duty factors during exercise in two environments: A pilot study. Journal of Electromyography and Kinesiology. 2016;30:15-22. | Title |
|  | Rezaeitabar Y, Ulusoy I. Automatic 3D segmentation of individual facial muscles using unlabeled prior information. Int J Comput Assist Radiol Surg. 2012;7(1):35-41. | Abstract |
|  | Ribeiro MC, Regalo SCH, Pepato AO, Siéssere S, de Souza LG, Sverzut CE, Trivellato AE. Bite force, electromyography, and mandible mobility during the 6-month period after surgical treatment for isolated fractures of the zygomatico-orbital complex. Oral Surgery Oral Medicine Oral Pathology Oral Radiology and Endodontology. 2011;111(4):E1-E7. | Title |
|  | Rich ME. Masseter muscle bite force in first bicuspid and collapsed occlusion cases. International journal of orthodontics (Milwaukee, Wis). 2012;23(2):29-33. | Title |
|  | Ricketts RM. Bioprogressive therapy as an answer to orthodontic needs Part II. American Journal of Orthodontics. 1976;70(4):359-97. | Title |
|  | Riddle PC, Nickel JC, Liu Y, Gonzalez YM, Gallo LM, Conley RS, et al. Mechanobehavior and mandibular ramus length in different facial phenotypes. Angle Orthod. 2020;90(6):866-72. | Full text  Other outcomes |
|  | Riedel F, Sadick H, Maurer JT, Hörmann K. A case of a slowly growing cheek tumor. HNO. 2000;48(10):782-3. | Title |
|  | Riedel M, Saurenmann T, Horber D, Zucol Fröhlich F, Zivkovic V. Thrombophlebitis of a superficial temporal vein as an extracranial complication of bacterial sinusitis: A case report and review of literature. Swiss Medical Weekly. 2023;153:31S. | Title |
|  | Righetti MA, Palinkas M, Gonçalves LMN, Taube OS, Verri ED, Esposto DS, et al. The impact of osteoarthritis on stomatognathic system function. Osteoporosis International. 2020;31(SUPPL 1):S207. | Title |
|  | Rikimaru H, Kikuchi M, Itoh M, Tashiro M, Watanabe M. Mapping energy metabolism in jaw and tongue muscles during chewing. Journal of Dental Research. 2001;80(9):1849-53. | Title |
|  | Ringkob TP, Swartz DR, Greaser ML. Light microscopy and image analysis of thin filament lengths utilizing dual probes on beef, chicken, and rabbit myofibrils. Journal of Animal Science. 2004;82(5):1445-53. | Title |
|  | Ringqvist M. Fibre sizes of human masseter muscle in relation to bite force. Journal of the Neurological Sciences. 1973;19(3):297-305. | Title |
|  | Ringqvist M. Size and distribution of histochemical fibre types in masseter muscle of adults with different states of occlusion. Journal of the Neurological Sciences. 1974;22(4):429-38. | Title |
|  | Ringqvist M. Fiber types in human masticatory muscles. Relation to function. European Journal of Oral Sciences. 1974;82(4):333-55. | Title |
|  | Ro JY. Bite force measurement in awake rats: A behavioral model for persistent orofacial muscle pain and hyperalgesia. Journal of Orofacial Pain. 2005;19(2):159-67. | Title |
|  | Ro JY, Capra NF. Assessing mechanical sensitivity of masseter muscle in lightly anesthetized rats: A model for craniofacial muscle hyperalgesia. Neuroscience Research. 2006;56(1):119-23. | Title |
|  | Roatta S, Mohammed M, Turturici M, Milano L, Passatore M. A model for investigating the control of muscle blood flow: the masseteric artery in conscious rabbits. Physiological Measurement. 2010;31(9):N71-N7. | Title |
|  | Robert R, Legent F, Rogez JM, Menier Y, Heloury Y, Patra P, Leborgne J. The infratemporal fossa: A trial clarification. Surgical and Radiologic Anatomy. 1989;11(4):307-11. | Title |
|  | Roberta C, Pasquale P, Giacomo DR, Massimo M, Stefano V, Rosa V, et al. Mandibular coronoid process tumor resembling a mandibular condyle: A case report. Oral and Maxillofacial Surgery Cases. 2019;5(1). | Title |
|  | Roberts Shannon L, Li Z, Fattah A, McKee N, Agur AM. Pectoralis minor transfer for facial reanimation: Architectural study of donor and recipient muscles. Clinical Anatomy. 2016;29(1):95-6. | Title |
|  | Rodrigues da Silva MAM, Issa JPM, Vitti M, Rodrigues da Silva AM, Semprini M, Regalo SCH. Electromyographical analysis of the masseter muscle in dentulous and partially toothless patients with temporomandibular joint disorders. Electromyography and Clinical Neurophysiology. 2006;46(5):263-8. | Title |
|  | Rodrigues HG, Hautier L, Evans AR. Convergent Traits in Mammals Associated with Divergent Behaviors: the Case of the Continuous Dental Replacement in Rock-Wallabies and African Mole-Rats. Journal of Mammalian Evolution. 2017;24(3):261-74. | Title |
[truncated: 148,479 more chars]
